# Supplementary material for: Deletion of wfs1 Impairs Oligodendrocyte Precursor Cells Dorsal Distribution and Myelination Through the wfs1-hmgcs1 Axis in Zebrafish
Source: Biology (Basel). 2026 Aug 16;15(16):1402. doi: 10.3390/biology15161402 (PMC13510151; doi:10.3390/biology15161402)
Supplement: Supplementary file 1 [file biology-15-01402-s001.zip › File S1-Transcriptome sequencing data and differentially expressed genes (DEGs) in wfs1-- zebrafish at 5 dpf/1.GO/wfs1KOvsWT/GSEA/wfs1KO/gsea_report_for_wfs1KO_1700447271245.html]

Report for wfs1KO 1700447271245 [GSEA]


| GS   follow link to MSigDB | GS DETAILS | SIZE | ES | NES | NOM p-val | FDR q-val | FWER p-val | RANK AT MAX | LEADING EDGE || 1 | MANNOSIDASE\_ACTIVITY(GO:0015923) | Details ... | 31 | 0.67 | 2.52 | 0.000 | 0.011 | 0.000 | 1043 | tags=16%, list=3%, signal=17% |
| 2 | MANNOSYL\_OLIGOSACCHARIDE\_MANNOSIDASE\_ACTIVITY(GO:0015924) | Details ... | 28 | 0.68 | 2.43 | 0.000 | 0.011 | 0.011 | 1043 | tags=18%, list=3%, signal=18% |
| 3 | ALPHA\_MANNOSIDASE\_ACTIVITY(GO:0004559) | Details ... | 30 | 0.68 | 2.42 | 0.000 | 0.011 | 0.011 | 1043 | tags=17%, list=3%, signal=17% |
| 4 | ESCRT\_COMPLEX(GO:0036452) | Details ... | 26 | 0.51 | 2.33 | 0.000 | 0.011 | 0.011 | 2871 | tags=15%, list=9%, signal=17% |
| 5 | COTRANSLATIONAL\_PROTEIN\_TARGETING\_TO\_MEMBRANE(GO:0006613) | Details ... | 17 | 0.68 | 2.23 | 0.000 | 0.013 | 0.023 | 717 | tags=18%, list=2%, signal=18% |
| 6 | PROTEIN\_LOCALIZATION\_TO\_ENDOPLASMIC\_RETICULUM(GO:0070972) | Details ... | 37 | 0.43 | 2.23 | 0.000 | 0.013 | 0.023 | 543 | tags=8%, list=2%, signal=8% |
| 7 | RNA\_CAP\_BINDING(GO:0000339) | Details ... | 16 | 0.64 | 2.22 | 0.000 | 0.013 | 0.023 | 220 | tags=13%, list=1%, signal=13% |
| 8 | SRP\_DEPENDENT\_COTRANSLATIONAL\_PROTEIN\_TARGETING\_TO\_MEMBRANE(GO:0006614) | Details ... | 16 | 0.62 | 2.21 | 0.000 | 0.014 | 0.034 | 441 | tags=13%, list=1%, signal=13% |
| 9 | PROTEIN\_TARGETING\_TO\_ER(GO:0045047) | Details ... | 25 | 0.50 | 2.15 | 0.000 | 0.024 | 0.097 | 441 | tags=8%, list=1%, signal=8% |
| 10 | ESTABLISHMENT\_OF\_PROTEIN\_LOCALIZATION\_TO\_ENDOPLASMIC\_RETICULUM(GO:0072599) | Details ... | 25 | 0.50 | 2.15 | 0.000 | 0.021 | 0.097 | 441 | tags=8%, list=1%, signal=8% |
| 11 | RNA\_LOCALIZATION(GO:0006403) | Details ... | 94 | 0.34 | 2.10 | 0.000 | 0.030 | 0.110 | 1628 | tags=5%, list=5%, signal=6% |
| 12 | AMINOACYL\_TRNA\_EDITING\_ACTIVITY(GO:0002161) | Details ... | 25 | 0.64 | 2.07 | 0.000 | 0.038 | 0.156 | 3874 | tags=28%, list=12%, signal=32% |
| 13 | PROTEIN\_EXPORT\_FROM\_NUCLEUS(GO:0006611) | Details ... | 54 | 0.40 | 2.07 | 0.000 | 0.036 | 0.156 | 3131 | tags=11%, list=10%, signal=12% |
| 14 | CONDENSED\_CHROMOSOME(GO:0000793) | Details ... | 54 | 0.49 | 2.03 | 0.000 | 0.046 | 0.164 | 2204 | tags=13%, list=7%, signal=14% |
| 15 | DICARBOXYLIC\_ACID\_METABOLIC\_PROCESS(GO:0043648) | Details ... | 44 | 0.54 | 2.02 | 0.000 | 0.044 | 0.164 | 2628 | tags=20%, list=8%, signal=22% |
| 16 | CELLULAR\_MODIFIED\_AMINO\_ACID\_CATABOLIC\_PROCESS(GO:0042219) | Details ... | 19 | 0.73 | 2.02 | 0.000 | 0.043 | 0.164 | 3023 | tags=37%, list=9%, signal=41% |
| 17 | MITOCHONDRIAL\_LARGE\_RIBOSOMAL\_SUBUNIT(GO:0005762) | Details ... | 36 | 0.50 | 1.99 | 0.000 | 0.059 | 0.235 | 1842 | tags=8%, list=6%, signal=9% |
| 18 | ORGANELLAR\_LARGE\_RIBOSOMAL\_SUBUNIT(GO:0000315) | Details ... | 36 | 0.50 | 1.99 | 0.000 | 0.056 | 0.235 | 1842 | tags=8%, list=6%, signal=9% |
| 19 | EXTRINSIC\_COMPONENT\_OF\_ORGANELLE\_MEMBRANE(GO:0031312) | Details ... | 19 | 0.61 | 1.97 | 0.000 | 0.057 | 0.243 | 2823 | tags=21%, list=9%, signal=23% |
| 20 | NUCLEIC\_ACID\_TRANSPORT(GO:0050657) | Details ... | 84 | 0.33 | 1.95 | 0.000 | 0.075 | 0.274 | 1628 | tags=5%, list=5%, signal=5% |
| 21 | RNA\_TRANSPORT(GO:0050658) | Details ... | 84 | 0.33 | 1.95 | 0.000 | 0.071 | 0.274 | 1628 | tags=5%, list=5%, signal=5% |
| 22 | ESTABLISHMENT\_OF\_RNA\_LOCALIZATION(GO:0051236) | Details ... | 84 | 0.33 | 1.95 | 0.000 | 0.068 | 0.274 | 1628 | tags=5%, list=5%, signal=5% |
| 23 | NUCLEAR\_EXPORT(GO:0051168) | Details ... | 65 | 0.36 | 1.94 | 0.000 | 0.068 | 0.274 | 3131 | tags=9%, list=10%, signal=10% |
| 24 | TRANSLATION\_INITIATION\_FACTOR\_ACTIVITY(GO:0003743) | Details ... | 58 | 0.40 | 1.94 | 0.000 | 0.066 | 0.274 | 2870 | tags=10%, list=9%, signal=11% |
| 25 | SERINE\_FAMILY\_AMINO\_ACID\_BIOSYNTHETIC\_PROCESS(GO:0009070) | Details ... | 16 | 0.58 | 1.93 | 0.000 | 0.064 | 0.274 | 3798 | tags=31%, list=12%, signal=35% |
| 26 | ORGANIC\_HYDROXY\_COMPOUND\_CATABOLIC\_PROCESS(GO:1901616) | Details ... | 39 | 0.54 | 1.92 | 0.000 | 0.071 | 0.287 | 4470 | tags=31%, list=14%, signal=36% |
| 27 | NEUTRAL\_AMINO\_ACID\_TRANSPORT(GO:0015804) | Details ... | 22 | 0.58 | 1.91 | 0.000 | 0.074 | 0.313 | 4693 | tags=27%, list=15%, signal=32% |
| 28 | SNAP\_RECEPTOR\_ACTIVITY(GO:0005484) | Details ... | 45 | 0.53 | 1.90 | 0.000 | 0.077 | 0.340 | 2411 | tags=20%, list=8%, signal=22% |
| 29 | METALLOCARBOXYPEPTIDASE\_ACTIVITY(GO:0004181) | Details ... | 20 | 0.66 | 1.89 | 0.000 | 0.080 | 0.350 | 1609 | tags=30%, list=5%, signal=32% |
| 30 | RETINYL\_PALMITATE\_ESTERASE\_ACTIVITY(GO:0050253) | Details ... | 16 | 0.77 | 1.88 | 0.000 | 0.086 | 0.414 | 1157 | tags=31%, list=4%, signal=32% |
| 31 | SNRNA\_3'\_END\_PROCESSING(GO:0034472) | Details ... | 18 | 0.57 | 1.87 | 0.000 | 0.092 | 0.444 | 3814 | tags=17%, list=12%, signal=19% |
| 32 | CARBON\_NITROGEN\_LYASE\_ACTIVITY(GO:0016840) | Details ... | 15 | 0.59 | 1.87 | 0.000 | 0.092 | 0.463 | 4161 | tags=33%, list=13%, signal=38% |
| 33 | CYCLIN\_DEPENDENT\_PROTEIN\_SERINE\_THREONINE\_KINASE\_REGULATOR\_ACTIVITY(GO:0016538) | Details ... | 49 | 0.43 | 1.87 | 0.000 | 0.091 | 0.475 | 4869 | tags=22%, list=15%, signal=26% |
| 34 | RIBONUCLEOPROTEIN\_COMPLEX\_EXPORT\_FROM\_NUCLEUS(GO:0071426) | Details ... | 46 | 0.40 | 1.85 | 0.000 | 0.100 | 0.525 | 3131 | tags=9%, list=10%, signal=10% |
| 35 | OXIDOREDUCTASE\_ACTIVITY\_\_ACTING\_ON\_THE\_CH\_NH\_GROUP\_OF\_DONORS(GO:0016645) | Details ... | 30 | 0.58 | 1.85 | 0.000 | 0.103 | 0.563 | 5446 | tags=33%, list=17%, signal=40% |
| 36 | SNARE\_COMPLEX(GO:0031201) | Details ... | 62 | 0.45 | 1.84 | 0.000 | 0.103 | 0.563 | 3434 | tags=18%, list=11%, signal=20% |
| 37 | NUCLEOBASE\_CONTAINING\_COMPOUND\_TRANSPORT(GO:0015931) | Details ... | 108 | 0.26 | 1.83 | 0.000 | 0.111 | 0.589 | 3681 | tags=6%, list=12%, signal=7% |
| 38 | CYSTEINE\_TYPE\_ENDOPEPTIDASE\_ACTIVITY\_INVOLVED\_IN\_EXECUTION\_PHASE\_OF\_APOPTOSIS(GO:0097200) | Details ... | 16 | 0.63 | 1.83 | 0.000 | 0.117 | 0.604 | 3826 | tags=38%, list=12%, signal=43% |
| 39 | NUCLEOCYTOPLASMIC\_TRANSPORT(GO:0006913) | Details ... | 106 | 0.31 | 1.82 | 0.000 | 0.118 | 0.604 | 3131 | tags=8%, list=10%, signal=9% |
| 40 | NUCLEAR\_TRANSPORT(GO:0051169) | Details ... | 106 | 0.31 | 1.82 | 0.000 | 0.115 | 0.604 | 3131 | tags=8%, list=10%, signal=9% |
| 41 | REGULATION\_OF\_PEPTIDYL\_TYROSINE\_PHOSPHORYLATION(GO:0050730) | Details ... | 46 | 0.61 | 1.82 | 0.000 | 0.115 | 0.604 | 4330 | tags=37%, list=14%, signal=43% |
| 42 | RIBONUCLEOPROTEIN\_COMPLEX\_LOCALIZATION(GO:0071166) | Details ... | 47 | 0.39 | 1.82 | 0.026 | 0.113 | 0.604 | 3131 | tags=9%, list=10%, signal=9% |
| 43 | DNA\_DEPENDENT\_ATPASE\_ACTIVITY(GO:0008094) | Details ... | 43 | 0.56 | 1.82 | 0.000 | 0.114 | 0.604 | 1739 | tags=16%, list=5%, signal=17% |
| 44 | COFACTOR\_TRANSPORT(GO:0051181) | Details ... | 26 | 0.54 | 1.81 | 0.000 | 0.114 | 0.620 | 2401 | tags=23%, list=8%, signal=25% |
| 45 | FOLIC\_ACID\_CONTAINING\_COMPOUND\_METABOLIC\_PROCESS(GO:0006760) | Details ... | 17 | 0.60 | 1.80 | 0.000 | 0.126 | 0.654 | 4740 | tags=24%, list=15%, signal=28% |
| 46 | RNA\_EXPORT\_FROM\_NUCLEUS(GO:0006405) | Details ... | 55 | 0.37 | 1.80 | 0.026 | 0.130 | 0.671 | 1628 | tags=5%, list=5%, signal=6% |
| 47 | GOLGI\_ORGANIZATION(GO:0007030) | Details ... | 61 | 0.35 | 1.80 | 0.000 | 0.128 | 0.671 | 3924 | tags=16%, list=12%, signal=19% |
| 48 | RECIPROCAL\_MEIOTIC\_RECOMBINATION(GO:0007131) | Details ... | 27 | 0.69 | 1.80 | 0.000 | 0.129 | 0.671 | 1739 | tags=26%, list=5%, signal=27% |
| 49 | CONDENSED\_NUCLEAR\_CHROMOSOME(GO:0000794) | Details ... | 20 | 0.63 | 1.80 | 0.000 | 0.128 | 0.671 | 1297 | tags=20%, list=4%, signal=21% |
| 50 | SYNAPTIC\_VESICLE\_RECYCLING(GO:0036465) | Details ... | 38 | 0.58 | 1.79 | 0.000 | 0.128 | 0.682 | 2483 | tags=16%, list=8%, signal=17% |
| 51 | VACUOLAR\_TRANSPORT(GO:0007034) |  | 87 | 0.34 | 1.78 | 0.000 | 0.134 | 0.693 | 2336 | tags=9%, list=7%, signal=10% |
| 52 | REGULATION\_OF\_PROTEIN\_DEPHOSPHORYLATION(GO:0035304) |  | 26 | 0.51 | 1.78 | 0.000 | 0.136 | 0.710 | 5099 | tags=23%, list=16%, signal=27% |
| 53 | INNER\_MITOCHONDRIAL\_MEMBRANE\_ORGANIZATION(GO:0007007) |  | 23 | 0.47 | 1.77 | 0.026 | 0.139 | 0.710 | 3447 | tags=22%, list=11%, signal=24% |
| 54 | SERINE\_FAMILY\_AMINO\_ACID\_METABOLIC\_PROCESS(GO:0009069) |  | 36 | 0.46 | 1.77 | 0.000 | 0.139 | 0.740 | 3798 | tags=28%, list=12%, signal=31% |
| 55 | EXORIBONUCLEASE\_COMPLEX(GO:1905354) |  | 15 | 0.62 | 1.77 | 0.000 | 0.138 | 0.740 | 1460 | tags=20%, list=5%, signal=21% |
| 56 | TETRAHYDROFOLATE\_METABOLIC\_PROCESS(GO:0046653) |  | 15 | 0.63 | 1.77 | 0.000 | 0.139 | 0.740 | 4740 | tags=27%, list=15%, signal=31% |
| 57 | HOMOLOGOUS\_RECOMBINATION(GO:0035825) |  | 28 | 0.64 | 1.77 | 0.000 | 0.139 | 0.740 | 1739 | tags=25%, list=5%, signal=26% |
| 58 | PROTEIN\_TARGETING\_TO\_MEMBRANE(GO:0006612) |  | 62 | 0.42 | 1.76 | 0.000 | 0.139 | 0.740 | 7167 | tags=27%, list=22%, signal=35% |
| 59 | ACTIVATION\_OF\_CYSTEINE\_TYPE\_ENDOPEPTIDASE\_ACTIVITY\_INVOLVED\_IN\_APOPTOTIC\_PROCESS(GO:0006919) |  | 24 | 0.58 | 1.76 | 0.000 | 0.141 | 0.740 | 7045 | tags=46%, list=22%, signal=59% |
| 60 | OXIDOREDUCTASE\_ACTIVITY\_\_ACTING\_ON\_THE\_CH\_NH\_GROUP\_OF\_DONORS\_\_NAD\_OR\_NADP\_AS\_ACCEPTOR(GO:0016646) |  | 18 | 0.74 | 1.76 | 0.000 | 0.143 | 0.759 | 5012 | tags=44%, list=16%, signal=53% |
| 61 | COPPER\_ION\_BINDING(GO:0005507) |  | 38 | 0.49 | 1.76 | 0.000 | 0.141 | 0.759 | 4415 | tags=29%, list=14%, signal=34% |
| 62 | SYNAPTIC\_VESICLE\_ENDOCYTOSIS(GO:0048488) |  | 33 | 0.59 | 1.75 | 0.000 | 0.144 | 0.759 | 2483 | tags=15%, list=8%, signal=16% |
| 63 | REGULATION\_OF\_DEPHOSPHORYLATION(GO:0035303) |  | 35 | 0.41 | 1.75 | 0.000 | 0.151 | 0.777 | 5099 | tags=20%, list=16%, signal=24% |
| 64 | ANCHORING\_JUNCTION(GO:0070161) |  | 147 | 0.29 | 1.75 | 0.000 | 0.149 | 0.777 | 3862 | tags=12%, list=12%, signal=13% |
| 65 | CHROMATIN\_REMODELING(GO:0006338) |  | 85 | 0.35 | 1.74 | 0.000 | 0.149 | 0.777 | 941 | tags=5%, list=3%, signal=5% |
| 66 | NEUTRAL\_AMINO\_ACID\_TRANSMEMBRANE\_TRANSPORTER\_ACTIVITY(GO:0015175) |  | 24 | 0.55 | 1.74 | 0.000 | 0.154 | 0.797 | 4693 | tags=25%, list=15%, signal=29% |
| 67 | PROTEIN\_TARGETING\_TO\_VACUOLE(GO:0006623) |  | 22 | 0.62 | 1.74 | 0.000 | 0.154 | 0.797 | 2336 | tags=18%, list=7%, signal=20% |
| 68 | LATE\_ENDOSOME\_TO\_VACUOLE\_TRANSPORT(GO:0045324) |  | 23 | 0.38 | 1.74 | 0.000 | 0.154 | 0.797 | 2217 | tags=9%, list=7%, signal=9% |
| 69 | PHOSPHATIDYLINOSITOL\_PHOSPHORYLATION(GO:0046854) |  | 46 | 0.44 | 1.74 | 0.000 | 0.152 | 0.797 | 5065 | tags=30%, list=16%, signal=36% |
| 70 | ADHERENS\_JUNCTION(GO:0005912) |  | 142 | 0.30 | 1.73 | 0.000 | 0.153 | 0.809 | 3862 | tags=12%, list=12%, signal=14% |
| 71 | MULTIVESICULAR\_BODY(GO:0005771) |  | 17 | 0.55 | 1.73 | 0.000 | 0.157 | 0.827 | 3375 | tags=24%, list=11%, signal=26% |
| 72 | MICROVILLUS(GO:0005902) |  | 29 | 0.49 | 1.73 | 0.000 | 0.159 | 0.843 | 4479 | tags=28%, list=14%, signal=32% |
| 73 | OXIDOREDUCTASE\_COMPLEX(GO:1990204) |  | 74 | 0.40 | 1.72 | 0.025 | 0.159 | 0.843 | 2011 | tags=8%, list=6%, signal=9% |
| 74 | MEIOSIS\_I(GO:0007127) |  | 46 | 0.50 | 1.72 | 0.000 | 0.162 | 0.843 | 1739 | tags=17%, list=5%, signal=18% |
| 75 | ESTABLISHMENT\_OF\_PROTEIN\_LOCALIZATION\_TO\_MEMBRANE(GO:0090150) |  | 113 | 0.37 | 1.72 | 0.000 | 0.165 | 0.843 | 4261 | tags=18%, list=13%, signal=20% |
| 76 | TRANSLATION\_FACTOR\_ACTIVITY\_\_RNA\_BINDING(GO:0008135) |  | 98 | 0.32 | 1.71 | 0.000 | 0.169 | 0.843 | 2870 | tags=10%, list=9%, signal=11% |
| 77 | OXIDOREDUCTASE\_ACTIVITY\_\_ACTING\_ON\_THE\_CH\_CH\_GROUP\_OF\_DONORS\_\_NAD\_OR\_NADP\_AS\_ACCEPTOR(GO:0016628) |  | 24 | 0.57 | 1.71 | 0.000 | 0.169 | 0.843 | 5884 | tags=38%, list=18%, signal=46% |
| 78 | PEPTIDYL\_TYROSINE\_MODIFICATION(GO:0018212) |  | 57 | 0.53 | 1.71 | 0.000 | 0.172 | 0.843 | 4330 | tags=30%, list=14%, signal=34% |
| 79 | ENDOPLASMIC\_RETICULUM\_UNFOLDED\_PROTEIN\_RESPONSE(GO:0030968) |  | 33 | 0.42 | 1.71 | 0.000 | 0.170 | 0.843 | 2045 | tags=12%, list=6%, signal=13% |
| 80 | CEREBELLAR\_CORTEX\_DEVELOPMENT(GO:0021695) |  | 17 | 0.59 | 1.71 | 0.000 | 0.175 | 0.853 | 7640 | tags=41%, list=24%, signal=54% |
| 81 | DIOXYGENASE\_ACTIVITY(GO:0051213) |  | 92 | 0.37 | 1.70 | 0.000 | 0.178 | 0.853 | 5876 | tags=25%, list=18%, signal=31% |
| 82 | PEPTIDYL\_TYROSINE\_PHOSPHORYLATION(GO:0018108) |  | 54 | 0.55 | 1.70 | 0.000 | 0.178 | 0.853 | 4330 | tags=31%, list=14%, signal=36% |
| 83 | MEMBRANE\_ORGANIZATION(GO:0061024) |  | 367 | 0.28 | 1.70 | 0.000 | 0.179 | 0.853 | 4662 | tags=14%, list=15%, signal=17% |
| 84 | PROTEIN\_HOMOTETRAMERIZATION(GO:0051289) |  | 20 | 0.49 | 1.70 | 0.031 | 0.182 | 0.866 | 6718 | tags=40%, list=21%, signal=51% |
| 85 | PROTEIN\_KINASE\_COMPLEX(GO:1902911) |  | 65 | 0.30 | 1.69 | 0.000 | 0.184 | 0.881 | 4196 | tags=15%, list=13%, signal=18% |
| 86 | ISOPRENOID\_BIOSYNTHETIC\_PROCESS(GO:0008299) |  | 27 | 0.39 | 1.69 | 0.000 | 0.183 | 0.881 | 3032 | tags=15%, list=10%, signal=16% |
| 87 | REGULATION\_OF\_CELL\_CELL\_ADHESION(GO:0022407) |  | 90 | 0.35 | 1.69 | 0.000 | 0.185 | 0.881 | 3781 | tags=20%, list=12%, signal=23% |
| 88 | POSITIVE\_REGULATION\_OF\_PEPTIDYL\_TYROSINE\_PHOSPHORYLATION(GO:0050731) |  | 35 | 0.60 | 1.69 | 0.000 | 0.183 | 0.881 | 4330 | tags=40%, list=14%, signal=46% |
| 89 | NEGATIVE\_REGULATION\_OF\_HYDROLASE\_ACTIVITY(GO:0051346) |  | 114 | 0.40 | 1.69 | 0.000 | 0.182 | 0.881 | 3751 | tags=24%, list=12%, signal=27% |
| 90 | TRANSFERASE\_ACTIVITY\_\_TRANSFERRING\_AMINO\_ACYL\_GROUPS(GO:0016755) |  | 32 | 0.47 | 1.69 | 0.000 | 0.188 | 0.881 | 4114 | tags=25%, list=13%, signal=29% |
| 91 | HYDROLASE\_ACTIVITY\_\_HYDROLYZING\_O\_GLYCOSYL\_COMPOUNDS(GO:0004553) |  | 110 | 0.38 | 1.69 | 0.000 | 0.186 | 0.881 | 3858 | tags=15%, list=12%, signal=18% |
| 92 | NEGATIVE\_REGULATION\_OF\_ENDOPEPTIDASE\_ACTIVITY(GO:0010951) |  | 50 | 0.54 | 1.68 | 0.000 | 0.190 | 0.894 | 3550 | tags=34%, list=11%, signal=38% |
| 93 | PORPHYRIN\_CONTAINING\_COMPOUND\_METABOLIC\_PROCESS(GO:0006778) |  | 29 | 0.52 | 1.67 | 0.036 | 0.202 | 0.912 | 4639 | tags=24%, list=15%, signal=28% |
| 94 | HEME\_METABOLIC\_PROCESS(GO:0042168) |  | 29 | 0.52 | 1.67 | 0.036 | 0.200 | 0.912 | 4639 | tags=24%, list=15%, signal=28% |
| 95 | REGULATION\_OF\_ENDOPEPTIDASE\_ACTIVITY(GO:0052548) |  | 95 | 0.46 | 1.67 | 0.000 | 0.198 | 0.912 | 6262 | tags=38%, list=20%, signal=47% |
| 96 | REPLICATION\_FORK(GO:0005657) |  | 35 | 0.47 | 1.67 | 0.000 | 0.202 | 0.929 | 11051 | tags=54%, list=35%, signal=83% |
| 97 | CEREBELLAR\_CORTEX\_MORPHOGENESIS(GO:0021696) |  | 15 | 0.62 | 1.67 | 0.000 | 0.204 | 0.929 | 7640 | tags=47%, list=24%, signal=61% |
| 98 | REGULATION\_OF\_PEPTIDASE\_ACTIVITY(GO:0052547) |  | 132 | 0.41 | 1.67 | 0.000 | 0.204 | 0.929 | 5838 | tags=33%, list=18%, signal=41% |
| 99 | VITAMIN\_TRANSMEMBRANE\_TRANSPORTER\_ACTIVITY(GO:0090482) |  | 19 | 0.58 | 1.66 | 0.000 | 0.207 | 0.929 | 7797 | tags=42%, list=24%, signal=56% |
| 100 | CYSTEINE\_TYPE\_ENDOPEPTIDASE\_ACTIVITY\_INVOLVED\_IN\_APOPTOTIC\_PROCESS(GO:0097153) |  | 19 | 0.52 | 1.66 | 0.033 | 0.209 | 0.954 | 2287 | tags=32%, list=7%, signal=34% |
| 101 | MITOTIC\_SPINDLE(GO:0072686) |  | 42 | 0.43 | 1.66 | 0.000 | 0.208 | 0.954 | 8307 | tags=29%, list=26%, signal=39% |
| 102 | SNRNA\_PROCESSING(GO:0016180) |  | 23 | 0.46 | 1.66 | 0.036 | 0.209 | 0.954 | 6525 | tags=22%, list=20%, signal=27% |
| 103 | VESICLE\_BUDDING\_FROM\_MEMBRANE(GO:0006900) |  | 35 | 0.46 | 1.66 | 0.000 | 0.211 | 0.954 | 4817 | tags=26%, list=15%, signal=30% |
| 104 | DRUG\_CATABOLIC\_PROCESS(GO:0042737) |  | 66 | 0.41 | 1.65 | 0.000 | 0.215 | 0.954 | 6288 | tags=30%, list=20%, signal=38% |
| 105 | RESPONSE\_TO\_OXIDATIVE\_STRESS(GO:0006979) |  | 85 | 0.32 | 1.65 | 0.025 | 0.219 | 0.954 | 7071 | tags=28%, list=22%, signal=36% |
| 106 | SPHINGOLIPID\_BIOSYNTHETIC\_PROCESS(GO:0030148) |  | 65 | 0.45 | 1.65 | 0.000 | 0.217 | 0.954 | 4729 | tags=23%, list=15%, signal=27% |
| 107 | ENDOMEMBRANE\_SYSTEM\_ORGANIZATION(GO:0010256) |  | 199 | 0.28 | 1.65 | 0.000 | 0.218 | 0.954 | 4545 | tags=14%, list=14%, signal=16% |
| 108 | QUINONE\_BINDING(GO:0048038) |  | 16 | 0.55 | 1.65 | 0.020 | 0.219 | 0.954 | 3054 | tags=25%, list=10%, signal=28% |
| 109 | REGULATION\_OF\_PRODUCTION\_OF\_MOLECULAR\_MEDIATOR\_OF\_IMMUNE\_RESPONSE(GO:0002700) |  | 18 | 0.69 | 1.64 | 0.000 | 0.224 | 0.973 | 4083 | tags=50%, list=13%, signal=57% |
| 110 | VESICLE\_FUSION(GO:0006906) |  | 74 | 0.39 | 1.64 | 0.000 | 0.223 | 0.973 | 2411 | tags=14%, list=8%, signal=15% |
| 111 | VESICLE\_CYTOSKELETAL\_TRAFFICKING(GO:0099518) |  | 43 | 0.42 | 1.64 | 0.000 | 0.222 | 0.973 | 4479 | tags=19%, list=14%, signal=22% |
| 112 | VESICLE\_ORGANIZATION(GO:0016050) |  | 156 | 0.30 | 1.64 | 0.000 | 0.225 | 0.973 | 4817 | tags=15%, list=15%, signal=17% |
| 113 | VESICLE\_DOCKING(GO:0048278) |  | 49 | 0.44 | 1.64 | 0.041 | 0.232 | 0.973 | 3734 | tags=18%, list=12%, signal=21% |
| 114 | POSITIVE\_REGULATION\_OF\_PRODUCTION\_OF\_MOLECULAR\_MEDIATOR\_OF\_IMMUNE\_RESPONSE(GO:0002702) |  | 15 | 0.69 | 1.64 | 0.000 | 0.231 | 0.973 | 3950 | tags=47%, list=12%, signal=53% |
| 115 | ATPASE\_REGULATOR\_ACTIVITY(GO:0060590) |  | 25 | 0.43 | 1.63 | 0.000 | 0.234 | 0.989 | 2846 | tags=12%, list=9%, signal=13% |
| 116 | MEMBRANE\_DOCKING(GO:0022406) |  | 58 | 0.42 | 1.63 | 0.000 | 0.232 | 0.989 | 3734 | tags=19%, list=12%, signal=21% |
| 117 | ORGANELLE\_LOCALIZATION\_BY\_MEMBRANE\_TETHERING(GO:0140056) |  | 58 | 0.42 | 1.63 | 0.000 | 0.230 | 0.989 | 3734 | tags=19%, list=12%, signal=21% |
| 118 | LIPID\_DROPLET(GO:0005811) |  | 31 | 0.47 | 1.63 | 0.000 | 0.230 | 0.989 | 5442 | tags=26%, list=17%, signal=31% |
| 119 | INTRACELLULAR\_PROTEIN\_TRANSPORT(GO:0006886) |  | 517 | 0.23 | 1.63 | 0.000 | 0.230 | 0.989 | 4444 | tags=12%, list=14%, signal=14% |
| 120 | CEREBELLUM\_MORPHOGENESIS(GO:0021587) |  | 22 | 0.50 | 1.62 | 0.000 | 0.242 | 1.000 | 3519 | tags=23%, list=11%, signal=26% |
| 121 | NEGATIVE\_REGULATION\_OF\_NEURON\_DEATH(GO:1901215) |  | 26 | 0.46 | 1.62 | 0.000 | 0.240 | 1.000 | 2668 | tags=19%, list=8%, signal=21% |
| 122 | NITROGEN\_COMPOUND\_TRANSPORT(GO:0071705) |  | 982 | 0.23 | 1.62 | 0.000 | 0.239 | 1.000 | 3184 | tags=9%, list=10%, signal=10% |
| 123 | HYDROLASE\_ACTIVITY\_\_ACTING\_ON\_GLYCOSYL\_BONDS(GO:0016798) |  | 131 | 0.36 | 1.62 | 0.000 | 0.240 | 1.000 | 5390 | tags=19%, list=17%, signal=23% |
| 124 | REGULATION\_OF\_HYDROLASE\_ACTIVITY(GO:0051336) |  | 353 | 0.32 | 1.62 | 0.000 | 0.239 | 1.000 | 6793 | tags=26%, list=21%, signal=33% |
| 125 | ENDORIBONUCLEASE\_ACTIVITY(GO:0004521) |  | 47 | 0.47 | 1.62 | 0.066 | 0.245 | 1.000 | 3639 | tags=17%, list=11%, signal=19% |
| 126 | SUPEROXIDE\_METABOLIC\_PROCESS(GO:0006801) |  | 21 | 0.56 | 1.62 | 0.000 | 0.245 | 1.000 | 3991 | tags=24%, list=13%, signal=27% |
| 127 | NEUROTRANSMITTER\_BIOSYNTHETIC\_PROCESS(GO:0042136) |  | 33 | 0.47 | 1.62 | 0.026 | 0.244 | 1.000 | 1508 | tags=15%, list=5%, signal=16% |
| 128 | NEGATIVE\_REGULATION\_OF\_PROTEIN\_METABOLIC\_PROCESS(GO:0051248) |  | 326 | 0.31 | 1.61 | 0.000 | 0.245 | 1.000 | 6162 | tags=22%, list=19%, signal=27% |
| 129 | NEGATIVE\_REGULATION\_OF\_CELLULAR\_PROTEIN\_METABOLIC\_PROCESS(GO:0032269) |  | 318 | 0.32 | 1.61 | 0.000 | 0.251 | 1.000 | 6162 | tags=23%, list=19%, signal=28% |
| 130 | NUCLEAR\_CHROMOSOME(GO:0000228) |  | 148 | 0.35 | 1.61 | 0.000 | 0.250 | 1.000 | 9537 | tags=31%, list=30%, signal=44% |
| 131 | PRIMARY\_ALCOHOL\_METABOLIC\_PROCESS(GO:0034308) |  | 18 | 0.61 | 1.61 | 0.000 | 0.250 | 1.000 | 1945 | tags=22%, list=6%, signal=24% |
| 132 | AROMATIC\_AMINO\_ACID\_FAMILY\_CATABOLIC\_PROCESS(GO:0009074) |  | 16 | 0.59 | 1.61 | 0.014 | 0.249 | 1.000 | 5876 | tags=50%, list=18%, signal=61% |
| 133 | NEGATIVE\_REGULATION\_OF\_MRNA\_METABOLIC\_PROCESS(GO:1903312) |  | 23 | 0.44 | 1.61 | 0.000 | 0.247 | 1.000 | 7888 | tags=35%, list=25%, signal=46% |
| 134 | ER\_TO\_GOLGI\_TRANSPORT\_VESICLE\_MEMBRANE(GO:0012507) |  | 24 | 0.35 | 1.60 | 0.020 | 0.251 | 1.000 | 4817 | tags=21%, list=15%, signal=25% |
| 135 | CERAMIDE\_BIOSYNTHETIC\_PROCESS(GO:0046513) |  | 43 | 0.46 | 1.60 | 0.000 | 0.254 | 1.000 | 3184 | tags=14%, list=10%, signal=15% |
| 136 | NEGATIVE\_REGULATION\_OF\_PROTEOLYSIS(GO:0045861) |  | 96 | 0.41 | 1.60 | 0.000 | 0.255 | 1.000 | 4631 | tags=29%, list=15%, signal=34% |
| 137 | AEROBIC\_RESPIRATION(GO:0009060) |  | 48 | 0.36 | 1.60 | 0.000 | 0.262 | 1.000 | 3693 | tags=17%, list=12%, signal=19% |
| 138 | TRANSLATIONAL\_INITIATION(GO:0006413) |  | 83 | 0.30 | 1.59 | 0.114 | 0.266 | 1.000 | 2870 | tags=7%, list=9%, signal=8% |
| 139 | NUCLEAR\_CHROMOSOME\_PART(GO:0044454) |  | 141 | 0.35 | 1.59 | 0.000 | 0.268 | 1.000 | 9537 | tags=32%, list=30%, signal=45% |
| 140 | REGULATION\_OF\_PROTEOLYSIS(GO:0030162) |  | 193 | 0.32 | 1.59 | 0.000 | 0.268 | 1.000 | 6262 | tags=28%, list=20%, signal=35% |
| 141 | NEGATIVE\_REGULATION\_OF\_PEPTIDASE\_ACTIVITY(GO:0010466) |  | 86 | 0.43 | 1.59 | 0.000 | 0.266 | 1.000 | 4631 | tags=31%, list=15%, signal=37% |
| 142 | MEIOSIS\_I\_CELL\_CYCLE\_PROCESS(GO:0061982) |  | 52 | 0.46 | 1.59 | 0.000 | 0.265 | 1.000 | 4474 | tags=23%, list=14%, signal=27% |
| 143 | ORGANELLE\_MEMBRANE\_FUSION(GO:0090174) |  | 76 | 0.38 | 1.59 | 0.000 | 0.268 | 1.000 | 2411 | tags=13%, list=8%, signal=14% |
| 144 | TETRAPYRROLE\_METABOLIC\_PROCESS(GO:0033013) |  | 33 | 0.46 | 1.58 | 0.090 | 0.281 | 1.000 | 4639 | tags=21%, list=15%, signal=25% |
| 145 | OXIDOREDUCTASE\_ACTIVITY(GO:0016491) |  | 761 | 0.29 | 1.58 | 0.000 | 0.280 | 1.000 | 6109 | tags=22%, list=19%, signal=27% |
| 146 | ORGANIC\_ACID\_CATABOLIC\_PROCESS(GO:0016054) |  | 159 | 0.30 | 1.58 | 0.000 | 0.283 | 1.000 | 6342 | tags=24%, list=20%, signal=30% |
| 147 | CARBOXYLIC\_ACID\_CATABOLIC\_PROCESS(GO:0046395) |  | 159 | 0.30 | 1.58 | 0.000 | 0.281 | 1.000 | 6342 | tags=24%, list=20%, signal=30% |
| 148 | CEREBELLUM\_DEVELOPMENT(GO:0021549) |  | 41 | 0.39 | 1.58 | 0.034 | 0.282 | 1.000 | 3519 | tags=15%, list=11%, signal=16% |
| 149 | AMIDE\_TRANSPORT(GO:0042886) |  | 802 | 0.21 | 1.58 | 0.000 | 0.281 | 1.000 | 3184 | tags=9%, list=10%, signal=10% |
| 150 | PROTEIN\_INSERTION\_INTO\_MEMBRANE(GO:0051205) |  | 22 | 0.43 | 1.58 | 0.000 | 0.286 | 1.000 | 4026 | tags=23%, list=13%, signal=26% |
| 151 | OXIDOREDUCTASE\_ACTIVITY\_\_ACTING\_ON\_THE\_CH\_CH\_GROUP\_OF\_DONORS(GO:0016627) |  | 53 | 0.38 | 1.57 | 0.022 | 0.286 | 1.000 | 6280 | tags=28%, list=20%, signal=35% |
| 152 | GLYCOSAMINOGLYCAN\_CATABOLIC\_PROCESS(GO:0006027) |  | 15 | 0.50 | 1.57 | 0.000 | 0.287 | 1.000 | 5269 | tags=33%, list=17%, signal=40% |
| 153 | MEIOTIC\_NUCLEAR\_DIVISION(GO:0140013) |  | 58 | 0.39 | 1.57 | 0.000 | 0.288 | 1.000 | 1739 | tags=14%, list=5%, signal=15% |
| 154 | PEPTIDE\_TRANSPORT(GO:0015833) |  | 786 | 0.21 | 1.57 | 0.000 | 0.288 | 1.000 | 3184 | tags=9%, list=10%, signal=10% |
| 155 | VESICLE\_MEDIATED\_TRANSPORT\_IN\_SYNAPSE(GO:0099003) |  | 93 | 0.40 | 1.57 | 0.049 | 0.288 | 1.000 | 2932 | tags=13%, list=9%, signal=14% |
| 156 | PHOSPHOLIPASE\_ACTIVITY(GO:0004620) |  | 91 | 0.37 | 1.57 | 0.000 | 0.288 | 1.000 | 5761 | tags=20%, list=18%, signal=24% |
| 157 | PERIPHERAL\_NERVOUS\_SYSTEM\_NEURON\_AXONOGENESIS(GO:0048936) |  | 15 | 0.49 | 1.57 | 0.019 | 0.287 | 1.000 | 2065 | tags=13%, list=6%, signal=14% |
| 158 | REGULATION\_OF\_CELL\_ADHESION(GO:0030155) |  | 140 | 0.29 | 1.57 | 0.000 | 0.287 | 1.000 | 4032 | tags=16%, list=13%, signal=18% |
| 159 | ORGANIC\_SUBSTANCE\_TRANSPORT(GO:0071702) |  | 1211 | 0.23 | 1.57 | 0.000 | 0.288 | 1.000 | 4817 | tags=14%, list=15%, signal=15% |
| 160 | CARBOXYLIC\_ESTER\_HYDROLASE\_ACTIVITY(GO:0052689) |  | 108 | 0.34 | 1.57 | 0.000 | 0.287 | 1.000 | 5587 | tags=21%, list=18%, signal=26% |
| 161 | UBIQUINONE\_BIOSYNTHETIC\_PROCESS(GO:0006744) |  | 18 | 0.46 | 1.56 | 0.129 | 0.290 | 1.000 | 4737 | tags=22%, list=15%, signal=26% |
| 162 | UBIQUINONE\_METABOLIC\_PROCESS(GO:0006743) |  | 18 | 0.46 | 1.56 | 0.129 | 0.289 | 1.000 | 4737 | tags=22%, list=15%, signal=26% |
| 163 | QUINONE\_METABOLIC\_PROCESS(GO:1901661) |  | 18 | 0.46 | 1.56 | 0.129 | 0.287 | 1.000 | 4737 | tags=22%, list=15%, signal=26% |
| 164 | QUINONE\_BIOSYNTHETIC\_PROCESS(GO:1901663) |  | 18 | 0.46 | 1.56 | 0.129 | 0.285 | 1.000 | 4737 | tags=22%, list=15%, signal=26% |
| 165 | OXIDATION\_REDUCTION\_PROCESS(GO:0055114) |  | 222 | 0.27 | 1.56 | 0.000 | 0.284 | 1.000 | 4639 | tags=16%, list=15%, signal=18% |
| 166 | REGULATION\_OF\_ENDOCYTOSIS(GO:0030100) |  | 42 | 0.34 | 1.56 | 0.000 | 0.285 | 1.000 | 1030 | tags=7%, list=3%, signal=7% |
| 167 | CENTROSOME\_DUPLICATION(GO:0051298) |  | 33 | 0.47 | 1.56 | 0.055 | 0.283 | 1.000 | 5141 | tags=21%, list=16%, signal=25% |
| 168 | RECOMBINATIONAL\_REPAIR(GO:0000725) |  | 88 | 0.39 | 1.56 | 0.000 | 0.284 | 1.000 | 3853 | tags=22%, list=12%, signal=24% |
| 169 | PROTEASOME\_CORE\_COMPLEX(GO:0005839) |  | 22 | 0.54 | 1.56 | 0.039 | 0.283 | 1.000 | 3403 | tags=14%, list=11%, signal=15% |
| 170 | DEFENSE\_RESPONSE\_TO\_GRAM\_POSITIVE\_BACTERIUM(GO:0050830) |  | 23 | 0.50 | 1.56 | 0.025 | 0.285 | 1.000 | 2517 | tags=30%, list=8%, signal=33% |
| 171 | SWI\_SNF\_SUPERFAMILY\_TYPE\_COMPLEX(GO:0070603) |  | 58 | 0.31 | 1.56 | 0.021 | 0.287 | 1.000 | 9313 | tags=28%, list=29%, signal=39% |
| 172 | INTEGRIN\_BINDING(GO:0005178) |  | 42 | 0.33 | 1.56 | 0.000 | 0.286 | 1.000 | 2608 | tags=17%, list=8%, signal=18% |
| 173 | TRNA\_METHYLATION(GO:0030488) |  | 25 | 0.43 | 1.56 | 0.014 | 0.286 | 1.000 | 1840 | tags=8%, list=6%, signal=8% |
| 174 | REGULATION\_OF\_CELLULAR\_PROTEIN\_METABOLIC\_PROCESS(GO:0032268) |  | 832 | 0.27 | 1.56 | 0.000 | 0.285 | 1.000 | 4510 | tags=15%, list=14%, signal=17% |
| 175 | PLASMA\_MEMBRANE\_FUSION(GO:0045026) |  | 20 | 0.53 | 1.56 | 0.041 | 0.285 | 1.000 | 1898 | tags=20%, list=6%, signal=21% |
| 176 | NEUROTRANSMITTER\_METABOLIC\_PROCESS(GO:0042133) |  | 50 | 0.41 | 1.55 | 0.000 | 0.284 | 1.000 | 1508 | tags=12%, list=5%, signal=13% |
| 177 | TRANSLATIONAL\_ELONGATION(GO:0006414) |  | 46 | 0.35 | 1.55 | 0.025 | 0.284 | 1.000 | 2630 | tags=13%, list=8%, signal=14% |
| 178 | REGULATION\_OF\_PROTEIN\_METABOLIC\_PROCESS(GO:0051246) |  | 874 | 0.26 | 1.55 | 0.000 | 0.285 | 1.000 | 4738 | tags=15%, list=15%, signal=17% |
| 179 | DOUBLE\_STRAND\_BREAK\_REPAIR\_VIA\_HOMOLOGOUS\_RECOMBINATION(GO:0000724) |  | 87 | 0.39 | 1.55 | 0.000 | 0.288 | 1.000 | 3853 | tags=21%, list=12%, signal=23% |
| 180 | PRODUCTION\_OF\_MOLECULAR\_MEDIATOR\_OF\_IMMUNE\_RESPONSE(GO:0002440) |  | 40 | 0.53 | 1.55 | 0.000 | 0.288 | 1.000 | 4083 | tags=38%, list=13%, signal=43% |
| 181 | SMALL\_MOLECULE\_CATABOLIC\_PROCESS(GO:0044282) |  | 223 | 0.32 | 1.55 | 0.000 | 0.286 | 1.000 | 6380 | tags=25%, list=20%, signal=31% |
| 182 | PROTEIN\_TRANSPORT(GO:0015031) |  | 777 | 0.21 | 1.55 | 0.000 | 0.286 | 1.000 | 3184 | tags=9%, list=10%, signal=9% |
| 183 | ESTABLISHMENT\_OF\_PROTEIN\_LOCALIZATION\_TO\_VACUOLE(GO:0072666) |  | 38 | 0.43 | 1.55 | 0.112 | 0.285 | 1.000 | 2336 | tags=11%, list=7%, signal=11% |
| 184 | CARBOXYPEPTIDASE\_ACTIVITY(GO:0004180) |  | 31 | 0.53 | 1.55 | 0.053 | 0.284 | 1.000 | 2127 | tags=23%, list=7%, signal=24% |
| 185 | RESPONSE\_TO\_IONIZING\_RADIATION(GO:0010212) |  | 32 | 0.48 | 1.55 | 0.020 | 0.286 | 1.000 | 3800 | tags=28%, list=12%, signal=32% |
| 186 | MEMBRANE\_LIPID\_BIOSYNTHETIC\_PROCESS(GO:0046467) |  | 100 | 0.37 | 1.55 | 0.000 | 0.288 | 1.000 | 5007 | tags=20%, list=16%, signal=24% |
| 187 | MRNA\_TRANSPORT(GO:0051028) |  | 60 | 0.30 | 1.54 | 0.095 | 0.289 | 1.000 | 3681 | tags=8%, list=12%, signal=9% |
| 188 | ALPHA\_AMINO\_ACID\_CATABOLIC\_PROCESS(GO:1901606) |  | 77 | 0.35 | 1.54 | 0.000 | 0.287 | 1.000 | 2729 | tags=16%, list=9%, signal=17% |
| 189 | ALCOHOL\_METABOLIC\_PROCESS(GO:0006066) |  | 151 | 0.34 | 1.54 | 0.000 | 0.288 | 1.000 | 3533 | tags=17%, list=11%, signal=19% |
| 190 | CELLULAR\_AMINO\_ACID\_CATABOLIC\_PROCESS(GO:0009063) |  | 86 | 0.33 | 1.54 | 0.000 | 0.293 | 1.000 | 2729 | tags=14%, list=9%, signal=15% |
| 191 | AMINE\_METABOLIC\_PROCESS(GO:0009308) |  | 37 | 0.40 | 1.54 | 0.051 | 0.294 | 1.000 | 2458 | tags=19%, list=8%, signal=20% |
| 192 | ORGANIC\_ACID\_TRANSPORT(GO:0015849) |  | 145 | 0.33 | 1.54 | 0.000 | 0.299 | 1.000 | 4981 | tags=21%, list=16%, signal=25% |
| 193 | CELLULAR\_RESPONSE\_TO\_TOPOLOGICALLY\_INCORRECT\_PROTEIN(GO:0035967) |  | 59 | 0.36 | 1.53 | 0.033 | 0.299 | 1.000 | 2045 | tags=12%, list=6%, signal=13% |
| 194 | SINGLE\_STRANDED\_DNA\_BINDING(GO:0003697) |  | 62 | 0.33 | 1.53 | 0.000 | 0.300 | 1.000 | 6879 | tags=29%, list=22%, signal=37% |
| 195 | AMINOGLYCAN\_CATABOLIC\_PROCESS(GO:0006026) |  | 22 | 0.50 | 1.53 | 0.031 | 0.299 | 1.000 | 6288 | tags=41%, list=20%, signal=51% |
| 196 | NEGATIVE\_REGULATION\_OF\_MAPK\_CASCADE(GO:0043409) |  | 35 | 0.45 | 1.53 | 0.000 | 0.300 | 1.000 | 4422 | tags=29%, list=14%, signal=33% |
| 197 | CELLULAR\_RESPONSE\_TO\_UNFOLDED\_PROTEIN(GO:0034620) |  | 44 | 0.40 | 1.53 | 0.034 | 0.300 | 1.000 | 2952 | tags=16%, list=9%, signal=18% |
| 198 | REGULATION\_OF\_CATALYTIC\_ACTIVITY(GO:0050790) |  | 631 | 0.30 | 1.53 | 0.000 | 0.302 | 1.000 | 6793 | tags=24%, list=21%, signal=30% |
| 199 | MEMBRANE\_RAFT(GO:0045121) |  | 71 | 0.33 | 1.53 | 0.000 | 0.302 | 1.000 | 6042 | tags=21%, list=19%, signal=26% |
| 200 | MEMBRANE\_MICRODOMAIN(GO:0098857) |  | 71 | 0.33 | 1.53 | 0.000 | 0.300 | 1.000 | 6042 | tags=21%, list=19%, signal=26% |
| 201 | PEPTIDE\_LYSINE\_N\_ACETYLTRANSFERASE\_ACTIVITY(GO:0061733) |  | 24 | 0.48 | 1.53 | 0.064 | 0.302 | 1.000 | 3115 | tags=17%, list=10%, signal=18% |
| 202 | HYDROLASE\_ACTIVITY\_\_ACTING\_ON\_CARBON\_NITROGEN\_(BUT\_NOT\_PEPTIDE)\_BONDS\_\_IN\_CYCLIC\_AMIDINES(GO:0016814) |  | 33 | 0.40 | 1.53 | 0.000 | 0.303 | 1.000 | 8706 | tags=39%, list=27%, signal=54% |
| 203 | MEMBRANE\_REGION(GO:0098589) |  | 72 | 0.33 | 1.53 | 0.000 | 0.303 | 1.000 | 6042 | tags=21%, list=19%, signal=26% |
| 204 | TRANSLATION\_ELONGATION\_FACTOR\_ACTIVITY(GO:0003746) |  | 29 | 0.36 | 1.53 | 0.035 | 0.301 | 1.000 | 2070 | tags=14%, list=6%, signal=15% |
| 205 | MICROFILAMENT\_MOTOR\_ACTIVITY(GO:0000146) |  | 22 | 0.43 | 1.53 | 0.000 | 0.300 | 1.000 | 6871 | tags=27%, list=22%, signal=35% |
| 206 | HISTONE\_ACETYLTRANSFERASE\_ACTIVITY(GO:0004402) |  | 23 | 0.50 | 1.53 | 0.064 | 0.299 | 1.000 | 3115 | tags=17%, list=10%, signal=19% |
| 207 | COMMON\_BILE\_DUCT\_DEVELOPMENT(GO:0061009) |  | 19 | 0.46 | 1.53 | 0.000 | 0.298 | 1.000 | 3448 | tags=21%, list=11%, signal=24% |
| 208 | POSITIVE\_REGULATION\_OF\_RESPONSE\_TO\_WOUNDING(GO:1903036) |  | 17 | 0.46 | 1.52 | 0.000 | 0.300 | 1.000 | 3107 | tags=24%, list=10%, signal=26% |
| 209 | ENDOSOMAL\_TRANSPORT(GO:0016197) |  | 116 | 0.24 | 1.52 | 0.000 | 0.299 | 1.000 | 2506 | tags=9%, list=8%, signal=9% |
| 210 | DIOL\_METABOLIC\_PROCESS(GO:0034311) |  | 19 | 0.45 | 1.52 | 0.056 | 0.299 | 1.000 | 8292 | tags=42%, list=26%, signal=57% |
| 211 | CARBOXYLIC\_ACID\_TRANSPORT(GO:0046942) |  | 143 | 0.34 | 1.52 | 0.000 | 0.304 | 1.000 | 4981 | tags=22%, list=16%, signal=26% |
| 212 | NEURAL\_CREST\_CELL\_DIFFERENTIATION(GO:0014033) |  | 150 | 0.33 | 1.52 | 0.000 | 0.303 | 1.000 | 3614 | tags=12%, list=11%, signal=13% |
| 213 | OXIDOREDUCTASE\_ACTIVITY\_\_ACTING\_ON\_SINGLE\_DONORS\_WITH\_INCORPORATION\_OF\_MOLECULAR\_OXYGEN\_\_INCORPORATION\_OF\_TWO\_ATOMS\_OF\_OXYGEN(GO:0016702) |  | 36 | 0.50 | 1.52 | 0.054 | 0.302 | 1.000 | 5876 | tags=39%, list=18%, signal=48% |
| 214 | PRESYNAPSE\_ASSEMBLY(GO:0099054) |  | 23 | 0.60 | 1.52 | 0.025 | 0.301 | 1.000 | 1043 | tags=13%, list=3%, signal=13% |
| 215 | PRESYNAPSE\_ORGANIZATION(GO:0099172) |  | 23 | 0.60 | 1.52 | 0.025 | 0.300 | 1.000 | 1043 | tags=13%, list=3%, signal=13% |
| 216 | PEPTIDE\_N\_ACETYLTRANSFERASE\_ACTIVITY(GO:0034212) |  | 30 | 0.42 | 1.52 | 0.063 | 0.300 | 1.000 | 3115 | tags=13%, list=10%, signal=15% |
| 217 | REGULATION\_OF\_PROTEIN\_KINASE\_B\_SIGNALING(GO:0051896) |  | 28 | 0.48 | 1.52 | 0.000 | 0.301 | 1.000 | 5592 | tags=32%, list=18%, signal=39% |
| 218 | HINDBRAIN\_MORPHOGENESIS(GO:0021575) |  | 35 | 0.44 | 1.52 | 0.020 | 0.302 | 1.000 | 7640 | tags=34%, list=24%, signal=45% |
| 219 | ESTABLISHMENT\_OF\_PROTEIN\_LOCALIZATION(GO:0045184) |  | 804 | 0.20 | 1.52 | 0.000 | 0.302 | 1.000 | 4444 | tags=11%, list=14%, signal=13% |
| 220 | POSITIVE\_REGULATION\_OF\_DEVELOPMENTAL\_GROWTH(GO:0048639) |  | 20 | 0.44 | 1.52 | 0.054 | 0.301 | 1.000 | 3491 | tags=20%, list=11%, signal=22% |
| 221 | MEMBRANE\_FUSION(GO:0061025) |  | 96 | 0.34 | 1.51 | 0.030 | 0.307 | 1.000 | 5011 | tags=18%, list=16%, signal=21% |
| 222 | SYNAPTIC\_VESICLE\_FUSION\_TO\_PRESYNAPTIC\_ACTIVE\_ZONE\_MEMBRANE(GO:0031629) |  | 18 | 0.55 | 1.51 | 0.076 | 0.316 | 1.000 | 1898 | tags=22%, list=6%, signal=24% |
| 223 | VESICLE\_FUSION\_TO\_PLASMA\_MEMBRANE(GO:0099500) |  | 18 | 0.55 | 1.51 | 0.076 | 0.315 | 1.000 | 1898 | tags=22%, list=6%, signal=24% |
| 224 | OXIDOREDUCTASE\_ACTIVITY\_\_ACTING\_ON\_THE\_CH\_NH2\_GROUP\_OF\_DONORS(GO:0016638) |  | 24 | 0.55 | 1.51 | 0.078 | 0.317 | 1.000 | 6229 | tags=54%, list=20%, signal=67% |
| 225 | FATTY\_ACYL\_COA\_BINDING(GO:0000062) |  | 17 | 0.48 | 1.51 | 0.000 | 0.318 | 1.000 | 5292 | tags=35%, list=17%, signal=42% |
| 226 | FATTY\_ACID\_DERIVATIVE\_BINDING(GO:1901567) |  | 17 | 0.48 | 1.51 | 0.000 | 0.316 | 1.000 | 5292 | tags=35%, list=17%, signal=42% |
| 227 | NEGATIVE\_REGULATION\_OF\_NEURON\_APOPTOTIC\_PROCESS(GO:0043524) |  | 24 | 0.44 | 1.51 | 0.022 | 0.316 | 1.000 | 2398 | tags=17%, list=8%, signal=18% |
| 228 | OXIDOREDUCTASE\_ACTIVITY\_\_ACTING\_ON\_THE\_CH\_NH2\_GROUP\_OF\_DONORS\_\_OXYGEN\_AS\_ACCEPTOR(GO:0016641) |  | 20 | 0.61 | 1.50 | 0.048 | 0.325 | 1.000 | 6229 | tags=65%, list=20%, signal=81% |
| 229 | PEPTIDASE\_REGULATOR\_ACTIVITY(GO:0061134) |  | 140 | 0.36 | 1.50 | 0.048 | 0.326 | 1.000 | 4789 | tags=26%, list=15%, signal=31% |
| 230 | COLLAGEN\_FIBRIL\_ORGANIZATION(GO:0030199) |  | 28 | 0.43 | 1.50 | 0.086 | 0.329 | 1.000 | 6024 | tags=29%, list=19%, signal=35% |
| 231 | REGULATION\_OF\_JAK\_STAT\_CASCADE(GO:0046425) |  | 26 | 0.50 | 1.50 | 0.020 | 0.328 | 1.000 | 4682 | tags=31%, list=15%, signal=36% |
| 232 | REGULATION\_OF\_STAT\_CASCADE(GO:1904892) |  | 26 | 0.50 | 1.50 | 0.020 | 0.327 | 1.000 | 4682 | tags=31%, list=15%, signal=36% |
| 233 | PEPTIDASE\_INHIBITOR\_ACTIVITY(GO:0030414) |  | 133 | 0.38 | 1.50 | 0.048 | 0.330 | 1.000 | 4789 | tags=27%, list=15%, signal=32% |
| 234 | REGULATION\_OF\_RNA\_STABILITY(GO:0043487) |  | 45 | 0.35 | 1.50 | 0.016 | 0.330 | 1.000 | 10297 | tags=36%, list=32%, signal=52% |
| 235 | REGULATION\_OF\_MRNA\_STABILITY(GO:0043488) |  | 42 | 0.36 | 1.50 | 0.051 | 0.330 | 1.000 | 10297 | tags=36%, list=32%, signal=53% |
| 236 | OXIDOREDUCTASE\_ACTIVITY\_\_ACTING\_ON\_SINGLE\_DONORS\_WITH\_INCORPORATION\_OF\_MOLECULAR\_OXYGEN(GO:0016701) |  | 37 | 0.47 | 1.50 | 0.084 | 0.330 | 1.000 | 5876 | tags=38%, list=18%, signal=46% |
| 237 | MAP\_KINASE\_ACTIVITY(GO:0004707) |  | 19 | 0.51 | 1.50 | 0.000 | 0.329 | 1.000 | 6998 | tags=32%, list=22%, signal=40% |
| 238 | POSITIVE\_REGULATION\_OF\_CYSTEINE\_TYPE\_ENDOPEPTIDASE\_ACTIVITY\_INVOLVED\_IN\_APOPTOTIC\_PROCESS(GO:0043280) |  | 39 | 0.44 | 1.49 | 0.020 | 0.330 | 1.000 | 7470 | tags=41%, list=23%, signal=54% |
| 239 | PROTEIN\_INSERTION\_INTO\_ER\_MEMBRANE(GO:0045048) |  | 17 | 0.51 | 1.49 | 0.000 | 0.331 | 1.000 | 5606 | tags=29%, list=18%, signal=36% |
| 240 | REGULATION\_OF\_CYSTEINE\_TYPE\_ENDOPEPTIDASE\_ACTIVITY\_INVOLVED\_IN\_APOPTOTIC\_PROCESS(GO:0043281) |  | 49 | 0.38 | 1.49 | 0.000 | 0.330 | 1.000 | 7470 | tags=37%, list=23%, signal=48% |
| 241 | LATE\_ENDOSOME(GO:0005770) |  | 107 | 0.24 | 1.49 | 0.000 | 0.330 | 1.000 | 3659 | tags=11%, list=11%, signal=13% |
| 242 | INOSITOL\_PHOSPHATE\_METABOLIC\_PROCESS(GO:0043647) |  | 33 | 0.35 | 1.49 | 0.000 | 0.329 | 1.000 | 4736 | tags=18%, list=15%, signal=21% |
| 243 | CYCLASE\_ACTIVITY(GO:0009975) |  | 31 | 0.55 | 1.49 | 0.000 | 0.329 | 1.000 | 5190 | tags=32%, list=16%, signal=38% |
| 244 | LIPID\_PHOSPHORYLATION(GO:0046834) |  | 53 | 0.38 | 1.49 | 0.050 | 0.329 | 1.000 | 5065 | tags=32%, list=16%, signal=38% |
| 245 | PHENOL\_CONTAINING\_COMPOUND\_BIOSYNTHETIC\_PROCESS(GO:0046189) |  | 16 | 0.55 | 1.49 | 0.023 | 0.333 | 1.000 | 690 | tags=13%, list=2%, signal=13% |
| 246 | ALDO\_KETO\_REDUCTASE\_(NADP)\_ACTIVITY(GO:0004033) |  | 17 | 0.58 | 1.49 | 0.032 | 0.337 | 1.000 | 2400 | tags=24%, list=8%, signal=25% |
| 247 | VESICLE\_TRANSPORT\_ALONG\_ACTIN\_FILAMENT(GO:0030050) |  | 21 | 0.41 | 1.49 | 0.000 | 0.336 | 1.000 | 4479 | tags=19%, list=14%, signal=22% |
| 248 | ACTIN\_FILAMENT\_BASED\_TRANSPORT(GO:0099515) |  | 21 | 0.41 | 1.49 | 0.000 | 0.335 | 1.000 | 4479 | tags=19%, list=14%, signal=22% |
| 249 | RESPONSE\_TO\_UNFOLDED\_PROTEIN(GO:0006986) |  | 49 | 0.37 | 1.49 | 0.083 | 0.334 | 1.000 | 7036 | tags=31%, list=22%, signal=39% |
| 250 | ENZYME\_REGULATOR\_ACTIVITY(GO:0030234) |  | 662 | 0.24 | 1.49 | 0.000 | 0.333 | 1.000 | 5109 | tags=15%, list=16%, signal=18% |
| 251 | ORGANELLE\_FUSION(GO:0048284) |  | 92 | 0.34 | 1.48 | 0.034 | 0.333 | 1.000 | 5011 | tags=17%, list=16%, signal=21% |
| 252 | ACID\_THIOL\_LIGASE\_ACTIVITY(GO:0016878) |  | 27 | 0.45 | 1.48 | 0.030 | 0.334 | 1.000 | 7611 | tags=37%, list=24%, signal=49% |
| 253 | PHOTOPERIODISM(GO:0009648) |  | 20 | 0.53 | 1.48 | 0.113 | 0.334 | 1.000 | 10190 | tags=55%, list=32%, signal=81% |
| 254 | DNA\_RECOMBINATION(GO:0006310) |  | 158 | 0.35 | 1.48 | 0.000 | 0.335 | 1.000 | 3853 | tags=16%, list=12%, signal=19% |
| 255 | PIGMENT\_METABOLIC\_PROCESS(GO:0042440) |  | 48 | 0.37 | 1.48 | 0.037 | 0.336 | 1.000 | 2691 | tags=13%, list=8%, signal=14% |
| 256 | PROTEIN\_LOCALIZATION\_TO\_VACUOLE(GO:0072665) |  | 46 | 0.39 | 1.48 | 0.093 | 0.338 | 1.000 | 2336 | tags=9%, list=7%, signal=9% |
| 257 | PROTEIN\_LOCALIZATION\_TO\_ORGANELLE(GO:0033365) |  | 300 | 0.22 | 1.48 | 0.029 | 0.337 | 1.000 | 4426 | tags=10%, list=14%, signal=12% |
| 258 | CARTILAGE\_MORPHOGENESIS(GO:0060536) |  | 27 | 0.41 | 1.48 | 0.146 | 0.341 | 1.000 | 4905 | tags=19%, list=15%, signal=22% |
| 259 | RESPONSE\_TO\_VITAMIN(GO:0033273) |  | 15 | 0.45 | 1.47 | 0.062 | 0.344 | 1.000 | 5108 | tags=27%, list=16%, signal=32% |
| 260 | HEME\_BINDING(GO:0020037) |  | 146 | 0.36 | 1.47 | 0.032 | 0.345 | 1.000 | 6458 | tags=34%, list=20%, signal=43% |
| 261 | REGULATION\_OF\_CENTROSOME\_CYCLE(GO:0046605) |  | 17 | 0.49 | 1.47 | 0.099 | 0.346 | 1.000 | 1794 | tags=12%, list=6%, signal=12% |
| 262 | OPTIC\_NERVE\_DEVELOPMENT(GO:0021554) |  | 17 | 0.50 | 1.47 | 0.092 | 0.346 | 1.000 | 3240 | tags=18%, list=10%, signal=20% |
| 263 | REGULATION\_OF\_PHOSPHATE\_METABOLIC\_PROCESS(GO:0019220) |  | 524 | 0.29 | 1.47 | 0.000 | 0.348 | 1.000 | 4470 | tags=16%, list=14%, signal=19% |
| 264 | REGULATION\_OF\_PHOSPHORUS\_METABOLIC\_PROCESS(GO:0051174) |  | 524 | 0.29 | 1.47 | 0.000 | 0.346 | 1.000 | 4470 | tags=16%, list=14%, signal=19% |
| 265 | DEFENSE\_RESPONSE\_TO\_GRAM\_NEGATIVE\_BACTERIUM(GO:0050829) |  | 16 | 0.47 | 1.47 | 0.035 | 0.346 | 1.000 | 2244 | tags=31%, list=7%, signal=34% |
| 266 | NEGATIVE\_REGULATION\_OF\_CATALYTIC\_ACTIVITY(GO:0043086) |  | 185 | 0.31 | 1.47 | 0.000 | 0.347 | 1.000 | 3751 | tags=17%, list=12%, signal=19% |
| 267 | DEAMINASE\_ACTIVITY(GO:0019239) |  | 28 | 0.39 | 1.47 | 0.000 | 0.352 | 1.000 | 8706 | tags=43%, list=27%, signal=59% |
| 268 | POLYOL\_METABOLIC\_PROCESS(GO:0019751) |  | 68 | 0.30 | 1.47 | 0.000 | 0.353 | 1.000 | 5012 | tags=22%, list=16%, signal=26% |
| 269 | SYNAPTIC\_VESICLE\_CYCLE(GO:0099504) |  | 92 | 0.39 | 1.46 | 0.050 | 0.354 | 1.000 | 2932 | tags=12%, list=9%, signal=13% |
| 270 | NUCLEOSOME\_BINDING(GO:0031491) |  | 41 | 0.36 | 1.46 | 0.000 | 0.354 | 1.000 | 792 | tags=7%, list=2%, signal=7% |
| 271 | REGULATION\_OF\_CENTROSOME\_DUPLICATION(GO:0010824) |  | 16 | 0.50 | 1.46 | 0.127 | 0.357 | 1.000 | 1794 | tags=13%, list=6%, signal=13% |
| 272 | NEUROMAST\_DEPOSITION(GO:0048885) |  | 15 | 0.57 | 1.46 | 0.020 | 0.357 | 1.000 | 5846 | tags=40%, list=18%, signal=49% |
| 273 | ALCOHOL\_CATABOLIC\_PROCESS(GO:0046164) |  | 30 | 0.40 | 1.46 | 0.071 | 0.360 | 1.000 | 4470 | tags=23%, list=14%, signal=27% |
| 274 | TETRAPYRROLE\_BINDING(GO:0046906) |  | 154 | 0.35 | 1.46 | 0.032 | 0.359 | 1.000 | 6458 | tags=33%, list=20%, signal=41% |
| 275 | ALCOHOL\_BIOSYNTHETIC\_PROCESS(GO:0046165) |  | 66 | 0.34 | 1.46 | 0.057 | 0.357 | 1.000 | 3158 | tags=17%, list=10%, signal=18% |
| 276 | CENTROSOME(GO:0005813) |  | 179 | 0.27 | 1.46 | 0.000 | 0.357 | 1.000 | 2268 | tags=8%, list=7%, signal=9% |
| 277 | POSITIVE\_REGULATION\_OF\_CYSTEINE\_TYPE\_ENDOPEPTIDASE\_ACTIVITY(GO:2001056) |  | 40 | 0.41 | 1.46 | 0.020 | 0.356 | 1.000 | 7470 | tags=40%, list=23%, signal=52% |
| 278 | CELLULAR\_MODIFIED\_AMINO\_ACID\_METABOLIC\_PROCESS(GO:0006575) |  | 111 | 0.31 | 1.46 | 0.063 | 0.356 | 1.000 | 5188 | tags=20%, list=16%, signal=24% |
| 279 | POSITIVE\_REGULATION\_OF\_IMMUNE\_EFFECTOR\_PROCESS(GO:0002699) |  | 26 | 0.50 | 1.46 | 0.032 | 0.356 | 1.000 | 2608 | tags=23%, list=8%, signal=25% |
| 280 | LIPID\_MODIFICATION(GO:0030258) |  | 171 | 0.32 | 1.46 | 0.050 | 0.357 | 1.000 | 5065 | tags=22%, list=16%, signal=26% |
| 281 | ENDOPEPTIDASE\_INHIBITOR\_ACTIVITY(GO:0004866) |  | 124 | 0.39 | 1.46 | 0.079 | 0.356 | 1.000 | 4678 | tags=28%, list=15%, signal=33% |
| 282 | CHROMOSOME\_CONDENSATION(GO:0030261) |  | 32 | 0.38 | 1.46 | 0.000 | 0.357 | 1.000 | 792 | tags=6%, list=2%, signal=6% |
| 283 | PROTEIN\_TETRAMERIZATION(GO:0051262) |  | 33 | 0.34 | 1.46 | 0.059 | 0.357 | 1.000 | 6718 | tags=30%, list=21%, signal=38% |
| 284 | MITOCHONDRIAL\_FISSION(GO:0000266) |  | 17 | 0.47 | 1.46 | 0.164 | 0.357 | 1.000 | 10198 | tags=41%, list=32%, signal=61% |
| 285 | REGULATION\_OF\_CHROMATIN\_ORGANIZATION(GO:1902275) |  | 45 | 0.36 | 1.45 | 0.136 | 0.363 | 1.000 | 3941 | tags=13%, list=12%, signal=15% |
| 286 | ENDOPEPTIDASE\_REGULATOR\_ACTIVITY(GO:0061135) |  | 127 | 0.38 | 1.45 | 0.079 | 0.363 | 1.000 | 4678 | tags=28%, list=15%, signal=32% |
| 287 | NUCLEAR\_CHROMATIN(GO:0000790) |  | 76 | 0.34 | 1.45 | 0.130 | 0.362 | 1.000 | 9537 | tags=30%, list=30%, signal=43% |
| 288 | POLY\_PURINE\_TRACT\_BINDING(GO:0070717) |  | 16 | 0.50 | 1.45 | 0.000 | 0.367 | 1.000 | 944 | tags=13%, list=3%, signal=13% |
| 289 | PHOSPHATIDYLINOSITOL\_3\_KINASE\_COMPLEX(GO:0005942) |  | 29 | 0.43 | 1.45 | 0.031 | 0.366 | 1.000 | 4871 | tags=28%, list=15%, signal=33% |
| 290 | CYSTEINE\_TYPE\_ENDOPEPTIDASE\_INHIBITOR\_ACTIVITY(GO:0004869) |  | 26 | 0.49 | 1.45 | 0.051 | 0.367 | 1.000 | 2444 | tags=31%, list=8%, signal=33% |
| 291 | ENZYME\_INHIBITOR\_ACTIVITY(GO:0004857) |  | 223 | 0.31 | 1.45 | 0.072 | 0.367 | 1.000 | 4879 | tags=20%, list=15%, signal=24% |
| 292 | KINASE\_REGULATOR\_ACTIVITY(GO:0019207) |  | 132 | 0.30 | 1.45 | 0.000 | 0.366 | 1.000 | 4879 | tags=17%, list=15%, signal=20% |
| 293 | MESENCHYMAL\_CELL\_DEVELOPMENT(GO:0014031) |  | 137 | 0.31 | 1.45 | 0.032 | 0.367 | 1.000 | 3614 | tags=12%, list=11%, signal=14% |
| 294 | NEURAL\_CREST\_CELL\_DEVELOPMENT(GO:0014032) |  | 137 | 0.31 | 1.45 | 0.032 | 0.366 | 1.000 | 3614 | tags=12%, list=11%, signal=14% |
| 295 | STEM\_CELL\_DEVELOPMENT(GO:0048864) |  | 137 | 0.31 | 1.45 | 0.032 | 0.365 | 1.000 | 3614 | tags=12%, list=11%, signal=14% |
| 296 | REGULATION\_OF\_PHOSPHORYLATION(GO:0042325) |  | 476 | 0.30 | 1.45 | 0.000 | 0.364 | 1.000 | 4470 | tags=16%, list=14%, signal=19% |
| 297 | REGULATION\_OF\_CYSTEINE\_TYPE\_ENDOPEPTIDASE\_ACTIVITY(GO:2000116) |  | 52 | 0.35 | 1.45 | 0.020 | 0.364 | 1.000 | 7470 | tags=35%, list=23%, signal=45% |
| 298 | ORGANIC\_CYCLIC\_COMPOUND\_CATABOLIC\_PROCESS(GO:1901361) |  | 318 | 0.25 | 1.44 | 0.000 | 0.364 | 1.000 | 4621 | tags=14%, list=14%, signal=16% |
| 299 | SERINE\_TYPE\_ENDOPEPTIDASE\_INHIBITOR\_ACTIVITY(GO:0004867) |  | 82 | 0.41 | 1.44 | 0.049 | 0.363 | 1.000 | 4678 | tags=29%, list=15%, signal=34% |
| 300 | PEPTIDOGLYCAN\_MURALYTIC\_ACTIVITY(GO:0061783) |  | 21 | 0.44 | 1.44 | 0.084 | 0.363 | 1.000 | 1686 | tags=14%, list=5%, signal=15% |
| 301 | PROTEIN\_TARGETING(GO:0006605) |  | 136 | 0.30 | 1.44 | 0.000 | 0.363 | 1.000 | 4261 | tags=12%, list=13%, signal=14% |
| 302 | RESPONSE\_TO\_TOPOLOGICALLY\_INCORRECT\_PROTEIN(GO:0035966) |  | 64 | 0.33 | 1.44 | 0.100 | 0.365 | 1.000 | 2045 | tags=11%, list=6%, signal=12% |
| 303 | RESPONSE\_TO\_COLD(GO:0009409) |  | 16 | 0.41 | 1.44 | 0.089 | 0.365 | 1.000 | 1802 | tags=19%, list=6%, signal=20% |
| 304 | SNARE\_BINDING(GO:0000149) |  | 108 | 0.33 | 1.44 | 0.069 | 0.365 | 1.000 | 2932 | tags=11%, list=9%, signal=12% |
| 305 | COFACTOR\_BINDING(GO:0048037) |  | 468 | 0.25 | 1.44 | 0.032 | 0.366 | 1.000 | 6458 | tags=24%, list=20%, signal=29% |
| 306 | ORGANIC\_ACID\_TRANSMEMBRANE\_TRANSPORTER\_ACTIVITY(GO:0005342) |  | 132 | 0.32 | 1.44 | 0.000 | 0.369 | 1.000 | 4862 | tags=19%, list=15%, signal=22% |
| 307 | HEART\_LOOPING(GO:0001947) |  | 126 | 0.30 | 1.44 | 0.000 | 0.368 | 1.000 | 6941 | tags=24%, list=22%, signal=30% |
| 308 | LIPASE\_ACTIVITY(GO:0016298) |  | 103 | 0.33 | 1.44 | 0.061 | 0.369 | 1.000 | 5761 | tags=18%, list=18%, signal=22% |
| 309 | REGULATION\_OF\_BEHAVIOR(GO:0050795) |  | 20 | 0.51 | 1.44 | 0.072 | 0.371 | 1.000 | 3624 | tags=30%, list=11%, signal=34% |
| 310 | REGULATION\_OF\_PROTEIN\_MODIFICATION\_PROCESS(GO:0031399) |  | 499 | 0.27 | 1.44 | 0.000 | 0.370 | 1.000 | 5163 | tags=17%, list=16%, signal=20% |
| 311 | REGULATION\_OF\_CYTOKINE\_PRODUCTION(GO:0001817) |  | 83 | 0.37 | 1.44 | 0.032 | 0.370 | 1.000 | 4083 | tags=23%, list=13%, signal=26% |
| 312 | ENDOPLASMIC\_RETICULUM\_ORGANIZATION(GO:0007029) |  | 45 | 0.33 | 1.44 | 0.000 | 0.369 | 1.000 | 6382 | tags=24%, list=20%, signal=31% |
| 313 | CYTOPLASMIC\_STRESS\_GRANULE(GO:0010494) |  | 25 | 0.33 | 1.44 | 0.089 | 0.370 | 1.000 | 1628 | tags=8%, list=5%, signal=8% |
| 314 | COA\_LIGASE\_ACTIVITY(GO:0016405) |  | 24 | 0.47 | 1.43 | 0.030 | 0.370 | 1.000 | 7611 | tags=42%, list=24%, signal=55% |
| 315 | CARBOXYLIC\_ACID\_TRANSMEMBRANE\_TRANSPORTER\_ACTIVITY(GO:0046943) |  | 129 | 0.33 | 1.43 | 0.000 | 0.369 | 1.000 | 4862 | tags=19%, list=15%, signal=23% |
| 316 | ORGANIC\_HYDROXY\_COMPOUND\_METABOLIC\_PROCESS(GO:1901615) |  | 206 | 0.31 | 1.43 | 0.065 | 0.374 | 1.000 | 4736 | tags=18%, list=15%, signal=22% |
| 317 | VESICLE\_TARGETING(GO:0006903) |  | 19 | 0.45 | 1.43 | 0.054 | 0.373 | 1.000 | 4653 | tags=21%, list=15%, signal=25% |
| 318 | REGULATION\_OF\_DENDRITE\_DEVELOPMENT(GO:0050773) |  | 29 | 0.44 | 1.43 | 0.108 | 0.374 | 1.000 | 4687 | tags=21%, list=15%, signal=24% |
| 319 | PTERIDINE\_CONTAINING\_COMPOUND\_METABOLIC\_PROCESS(GO:0042558) |  | 26 | 0.35 | 1.43 | 0.065 | 0.373 | 1.000 | 9320 | tags=38%, list=29%, signal=54% |
| 320 | STEM\_CELL\_PROLIFERATION(GO:0072089) |  | 26 | 0.40 | 1.43 | 0.051 | 0.374 | 1.000 | 3739 | tags=15%, list=12%, signal=17% |
| 321 | PROTEIN\_SERINE\_THREONINE\_TYROSINE\_KINASE\_ACTIVITY(GO:0004712) |  | 25 | 0.36 | 1.43 | 0.053 | 0.374 | 1.000 | 11173 | tags=48%, list=35%, signal=74% |
| 322 | METALLOEXOPEPTIDASE\_ACTIVITY(GO:0008235) |  | 45 | 0.43 | 1.43 | 0.137 | 0.376 | 1.000 | 4248 | tags=20%, list=13%, signal=23% |
| 323 | HORMONE\_MEDIATED\_SIGNALING\_PATHWAY(GO:0009755) |  | 63 | 0.37 | 1.43 | 0.086 | 0.376 | 1.000 | 7182 | tags=41%, list=23%, signal=53% |
| 324 | CATALYTIC\_ACTIVITY\_\_ACTING\_ON\_A\_GLYCOPROTEIN(GO:0140103) |  | 20 | 0.43 | 1.43 | 0.097 | 0.376 | 1.000 | 3843 | tags=30%, list=12%, signal=34% |
| 325 | ORGANELLE\_FISSION(GO:0048285) |  | 197 | 0.28 | 1.43 | 0.000 | 0.376 | 1.000 | 9099 | tags=28%, list=29%, signal=40% |
| 326 | POSITIVE\_REGULATION\_OF\_ENDOPEPTIDASE\_ACTIVITY(GO:0010950) |  | 42 | 0.40 | 1.43 | 0.042 | 0.379 | 1.000 | 7470 | tags=38%, list=23%, signal=50% |
| 327 | POSITIVE\_REGULATION\_OF\_PEPTIDASE\_ACTIVITY(GO:0010952) |  | 42 | 0.40 | 1.43 | 0.042 | 0.378 | 1.000 | 7470 | tags=38%, list=23%, signal=50% |
| 328 | MACROMOLECULE\_LOCALIZATION(GO:0033036) |  | 1291 | 0.19 | 1.42 | 0.000 | 0.382 | 1.000 | 4444 | tags=11%, list=14%, signal=12% |
| 329 | ORGANIC\_ANION\_TRANSPORT(GO:0015711) |  | 216 | 0.31 | 1.42 | 0.000 | 0.381 | 1.000 | 4981 | tags=19%, list=16%, signal=23% |
| 330 | ESTABLISHMENT\_OF\_VESICLE\_LOCALIZATION(GO:0051650) |  | 158 | 0.28 | 1.42 | 0.000 | 0.384 | 1.000 | 4714 | tags=15%, list=15%, signal=17% |
| 331 | RESPONSE\_TO\_NUTRIENT(GO:0007584) |  | 27 | 0.39 | 1.42 | 0.086 | 0.388 | 1.000 | 2057 | tags=15%, list=6%, signal=16% |
| 332 | CERAMIDE\_METABOLIC\_PROCESS(GO:0006672) |  | 65 | 0.33 | 1.42 | 0.030 | 0.388 | 1.000 | 3533 | tags=14%, list=11%, signal=16% |
| 333 | REGULATION\_OF\_MOLECULAR\_FUNCTION(GO:0065009) |  | 803 | 0.25 | 1.42 | 0.000 | 0.388 | 1.000 | 6793 | tags=22%, list=21%, signal=27% |
| 334 | REGULATION\_OF\_MRNA\_METABOLIC\_PROCESS(GO:1903311) |  | 128 | 0.25 | 1.42 | 0.000 | 0.392 | 1.000 | 7521 | tags=20%, list=24%, signal=26% |
| 335 | NEGATIVE\_REGULATION\_OF\_ALPHA\_BETA\_T\_CELL\_ACTIVATION(GO:0046636) |  | 15 | 0.48 | 1.42 | 0.033 | 0.392 | 1.000 | 3387 | tags=27%, list=11%, signal=30% |
| 336 | VITAMIN\_TRANSPORT(GO:0051180) |  | 19 | 0.50 | 1.41 | 0.096 | 0.394 | 1.000 | 7797 | tags=47%, list=24%, signal=63% |
| 337 | CARBOXYLIC\_ACID\_BINDING(GO:0031406) |  | 103 | 0.31 | 1.41 | 0.016 | 0.395 | 1.000 | 6711 | tags=25%, list=21%, signal=32% |
| 338 | VENTRAL\_MIDLINE\_DEVELOPMENT(GO:0007418) |  | 15 | 0.52 | 1.41 | 0.071 | 0.396 | 1.000 | 8662 | tags=40%, list=27%, signal=55% |
| 339 | RNA\_STABILIZATION(GO:0043489) |  | 20 | 0.42 | 1.41 | 0.050 | 0.397 | 1.000 | 10234 | tags=45%, list=32%, signal=66% |
| 340 | FATTY\_ACID\_LIGASE\_ACTIVITY(GO:0015645) |  | 18 | 0.46 | 1.41 | 0.083 | 0.398 | 1.000 | 6267 | tags=33%, list=20%, signal=41% |
| 341 | PROTEOLYSIS(GO:0006508) |  | 1024 | 0.23 | 1.41 | 0.000 | 0.398 | 1.000 | 5095 | tags=15%, list=16%, signal=18% |
| 342 | REGULATION\_OF\_CYTOKINESIS(GO:0032465) |  | 30 | 0.35 | 1.41 | 0.075 | 0.403 | 1.000 | 8474 | tags=40%, list=27%, signal=54% |
| 343 | ENDOCYTOSIS(GO:0006897) |  | 285 | 0.27 | 1.41 | 0.058 | 0.402 | 1.000 | 5412 | tags=14%, list=17%, signal=17% |
| 344 | PROTEIN\_KINASE\_B\_SIGNALING(GO:0043491) |  | 30 | 0.44 | 1.41 | 0.019 | 0.401 | 1.000 | 5592 | tags=30%, list=18%, signal=36% |
| 345 | SINGLE\_STRANDED\_RNA\_BINDING(GO:0003727) |  | 40 | 0.35 | 1.41 | 0.036 | 0.402 | 1.000 | 1694 | tags=13%, list=5%, signal=13% |
| 346 | INTEGRAL\_COMPONENT\_OF\_POSTSYNAPTIC\_SPECIALIZATION\_MEMBRANE(GO:0099060) |  | 17 | 0.51 | 1.41 | 0.063 | 0.401 | 1.000 | 1043 | tags=12%, list=3%, signal=12% |
| 347 | INTRINSIC\_COMPONENT\_OF\_POSTSYNAPTIC\_SPECIALIZATION\_MEMBRANE(GO:0098948) |  | 17 | 0.51 | 1.41 | 0.063 | 0.400 | 1.000 | 1043 | tags=12%, list=3%, signal=12% |
| 348 | CENTRIOLE\_ASSEMBLY(GO:0098534) |  | 22 | 0.48 | 1.41 | 0.025 | 0.399 | 1.000 | 10360 | tags=50%, list=32%, signal=74% |
| 349 | RESPONSE\_TO\_RADIATION(GO:0009314) |  | 161 | 0.31 | 1.41 | 0.000 | 0.398 | 1.000 | 7936 | tags=27%, list=25%, signal=36% |
| 350 | PRESYNAPTIC\_MEMBRANE(GO:0042734) |  | 43 | 0.38 | 1.41 | 0.101 | 0.400 | 1.000 | 6986 | tags=23%, list=22%, signal=30% |
| 351 | ALDEHYDE\_DEHYDROGENASE\_(NAD)\_ACTIVITY(GO:0004029) |  | 15 | 0.54 | 1.40 | 0.074 | 0.403 | 1.000 | 2030 | tags=27%, list=6%, signal=28% |
| 352 | REGULATION\_OF\_PROTEIN\_PHOSPHORYLATION(GO:0001932) |  | 397 | 0.28 | 1.40 | 0.000 | 0.404 | 1.000 | 5163 | tags=18%, list=16%, signal=21% |
| 353 | N\_ACETYLTRANSFERASE\_ACTIVITY(GO:0008080) |  | 58 | 0.34 | 1.40 | 0.054 | 0.404 | 1.000 | 3790 | tags=17%, list=12%, signal=20% |
| 354 | PROTEIN\_PALMITOYLATION(GO:0018345) |  | 39 | 0.38 | 1.40 | 0.000 | 0.404 | 1.000 | 9060 | tags=41%, list=28%, signal=57% |
| 355 | PHOSPHATIDYLINOSITOL\_METABOLIC\_PROCESS(GO:0046488) |  | 127 | 0.27 | 1.40 | 0.045 | 0.403 | 1.000 | 5065 | tags=18%, list=16%, signal=21% |
| 356 | SPINAL\_CORD\_PATTERNING(GO:0021511) |  | 16 | 0.54 | 1.40 | 0.069 | 0.403 | 1.000 | 11529 | tags=75%, list=36%, signal=117% |
| 357 | TRNA\_METHYLTRANSFERASE\_ACTIVITY(GO:0008175) |  | 26 | 0.42 | 1.40 | 0.109 | 0.402 | 1.000 | 1840 | tags=8%, list=6%, signal=8% |
| 358 | POSITIVE\_REGULATION\_OF\_HYDROLASE\_ACTIVITY(GO:0051345) |  | 186 | 0.29 | 1.40 | 0.000 | 0.403 | 1.000 | 6791 | tags=24%, list=21%, signal=31% |
| 359 | PHOSPHATIDYLCHOLINE\_BINDING(GO:0031210) |  | 16 | 0.43 | 1.40 | 0.120 | 0.402 | 1.000 | 3857 | tags=25%, list=12%, signal=28% |
| 360 | QUATERNARY\_AMMONIUM\_GROUP\_BINDING(GO:0050997) |  | 16 | 0.43 | 1.40 | 0.120 | 0.401 | 1.000 | 3857 | tags=25%, list=12%, signal=28% |
| 361 | UBIQUITIN\_PROTEIN\_LIGASE\_ACTIVITY(GO:0061630) |  | 234 | 0.24 | 1.40 | 0.000 | 0.402 | 1.000 | 4951 | tags=16%, list=16%, signal=19% |
| 362 | MEMBRANE\_LIPID\_METABOLIC\_PROCESS(GO:0006643) |  | 140 | 0.30 | 1.40 | 0.000 | 0.406 | 1.000 | 5110 | tags=19%, list=16%, signal=23% |
| 363 | NEURON\_DEATH(GO:0070997) |  | 54 | 0.32 | 1.40 | 0.103 | 0.405 | 1.000 | 3376 | tags=15%, list=11%, signal=17% |
| 364 | AROMATIC\_COMPOUND\_CATABOLIC\_PROCESS(GO:0019439) |  | 291 | 0.23 | 1.40 | 0.000 | 0.405 | 1.000 | 5021 | tags=14%, list=16%, signal=17% |
| 365 | CLATHRIN\_COATED\_PIT(GO:0005905) |  | 31 | 0.41 | 1.40 | 0.111 | 0.406 | 1.000 | 5310 | tags=19%, list=17%, signal=23% |
| 366 | ENDONUCLEASE\_ACTIVITY(GO:0004519) |  | 84 | 0.35 | 1.40 | 0.066 | 0.405 | 1.000 | 5165 | tags=18%, list=16%, signal=21% |
| 367 | CYTOKINE\_PRODUCTION(GO:0001816) |  | 90 | 0.36 | 1.40 | 0.032 | 0.405 | 1.000 | 4083 | tags=22%, list=13%, signal=25% |
| 368 | ACTIN\_FILAMENT(GO:0005884) |  | 58 | 0.28 | 1.39 | 0.043 | 0.405 | 1.000 | 249 | tags=3%, list=1%, signal=3% |
| 369 | CELLULAR\_MACROMOLECULE\_LOCALIZATION(GO:0070727) |  | 818 | 0.19 | 1.39 | 0.000 | 0.404 | 1.000 | 3184 | tags=8%, list=10%, signal=9% |
| 370 | REGULATION\_OF\_CYCLIN\_DEPENDENT\_PROTEIN\_SERINE\_THREONINE\_KINASE\_ACTIVITY(GO:0000079) |  | 32 | 0.35 | 1.39 | 0.061 | 0.404 | 1.000 | 4869 | tags=19%, list=15%, signal=22% |
| 371 | REGULATION\_OF\_CYCLIN\_DEPENDENT\_PROTEIN\_KINASE\_ACTIVITY(GO:1904029) |  | 32 | 0.35 | 1.39 | 0.061 | 0.403 | 1.000 | 4869 | tags=19%, list=15%, signal=22% |
| 372 | AMINO\_ACID\_TRANSPORT(GO:0006865) |  | 68 | 0.34 | 1.39 | 0.025 | 0.402 | 1.000 | 4981 | tags=21%, list=16%, signal=24% |
| 373 | CENTRIOLE\_REPLICATION(GO:0007099) |  | 21 | 0.49 | 1.39 | 0.000 | 0.401 | 1.000 | 10360 | tags=52%, list=32%, signal=78% |
| 374 | FATTY\_ACID\_METABOLIC\_PROCESS(GO:0006631) |  | 175 | 0.33 | 1.39 | 0.054 | 0.401 | 1.000 | 7381 | tags=30%, list=23%, signal=39% |
| 375 | MAP\_KINASE\_KINASE\_ACTIVITY(GO:0004708) |  | 15 | 0.50 | 1.39 | 0.056 | 0.407 | 1.000 | 11173 | tags=67%, list=35%, signal=103% |
| 376 | ALPHA\_AMINO\_ACID\_METABOLIC\_PROCESS(GO:1901605) |  | 164 | 0.27 | 1.39 | 0.085 | 0.406 | 1.000 | 5021 | tags=20%, list=16%, signal=23% |
| 377 | PEPTIDASE\_ACTIVITY\_\_ACTING\_ON\_L\_AMINO\_ACID\_PEPTIDES(GO:0070011) |  | 539 | 0.28 | 1.39 | 0.000 | 0.408 | 1.000 | 4934 | tags=17%, list=15%, signal=20% |
| 378 | REGULATION\_OF\_PROTEIN\_TYROSINE\_KINASE\_ACTIVITY(GO:0061097) |  | 19 | 0.46 | 1.39 | 0.101 | 0.410 | 1.000 | 11448 | tags=63%, list=36%, signal=98% |
| 379 | ENDOCYTIC\_RECYCLING(GO:0032456) |  | 29 | 0.31 | 1.39 | 0.083 | 0.410 | 1.000 | 1983 | tags=10%, list=6%, signal=11% |
| 380 | NUCLEAR\_OUTER\_MEMBRANE\_ENDOPLASMIC\_RETICULUM\_MEMBRANE\_NETWORK(GO:0042175) |  | 545 | 0.25 | 1.39 | 0.031 | 0.412 | 1.000 | 6513 | tags=20%, list=20%, signal=25% |
| 381 | ENDORIBONUCLEASE\_ACTIVITY\_\_PRODUCING\_5'\_PHOSPHOMONOESTERS(GO:0016891) |  | 26 | 0.45 | 1.39 | 0.165 | 0.411 | 1.000 | 5016 | tags=19%, list=16%, signal=23% |
| 382 | PHOSPHORUS\_OXYGEN\_LYASE\_ACTIVITY(GO:0016849) |  | 28 | 0.50 | 1.39 | 0.000 | 0.411 | 1.000 | 11057 | tags=57%, list=35%, signal=87% |
| 383 | PHOSPHATIDYLINOSITOL\_BINDING(GO:0035091) |  | 158 | 0.23 | 1.39 | 0.000 | 0.412 | 1.000 | 4865 | tags=13%, list=15%, signal=16% |
| 384 | NEURON\_APOPTOTIC\_PROCESS(GO:0051402) |  | 45 | 0.33 | 1.38 | 0.026 | 0.411 | 1.000 | 3376 | tags=16%, list=11%, signal=17% |
| 385 | ENDOPEPTIDASE\_ACTIVITY(GO:0004175) |  | 378 | 0.29 | 1.38 | 0.000 | 0.411 | 1.000 | 4934 | tags=19%, list=15%, signal=22% |
| 386 | CELLULAR\_LIPID\_METABOLIC\_PROCESS(GO:0044255) |  | 614 | 0.27 | 1.38 | 0.055 | 0.411 | 1.000 | 5158 | tags=18%, list=16%, signal=21% |
| 387 | NEGATIVE\_REGULATION\_OF\_EMBRYONIC\_DEVELOPMENT(GO:0045992) |  | 15 | 0.49 | 1.38 | 0.028 | 0.410 | 1.000 | 1891 | tags=13%, list=6%, signal=14% |
| 388 | CATALYTIC\_ACTIVITY\_\_ACTING\_ON\_RNA(GO:0140098) |  | 303 | 0.24 | 1.38 | 0.051 | 0.411 | 1.000 | 3874 | tags=10%, list=12%, signal=12% |
| 389 | OMEGA\_PEPTIDASE\_ACTIVITY(GO:0008242) |  | 16 | 0.52 | 1.38 | 0.030 | 0.411 | 1.000 | 3023 | tags=25%, list=9%, signal=28% |
| 390 | PEPTIDASE\_ACTIVITY(GO:0008233) |  | 558 | 0.28 | 1.38 | 0.062 | 0.411 | 1.000 | 4963 | tags=17%, list=16%, signal=20% |
| 391 | REGULATION\_OF\_KINASE\_ACTIVITY(GO:0043549) |  | 209 | 0.30 | 1.38 | 0.000 | 0.411 | 1.000 | 7756 | tags=27%, list=24%, signal=35% |
| 392 | CARBOXYLIC\_ACID\_METABOLIC\_PROCESS(GO:0019752) |  | 564 | 0.26 | 1.38 | 0.083 | 0.410 | 1.000 | 6016 | tags=21%, list=19%, signal=25% |
| 393 | PHOSPHOLIPASE\_A2\_ACTIVITY(GO:0004623) |  | 23 | 0.45 | 1.38 | 0.121 | 0.409 | 1.000 | 1592 | tags=13%, list=5%, signal=14% |
| 394 | GOLGI\_VESICLE\_BUDDING(GO:0048194) |  | 23 | 0.42 | 1.38 | 0.111 | 0.409 | 1.000 | 4817 | tags=26%, list=15%, signal=31% |
| 395 | ENDOPLASMIC\_RETICULUM\_MEMBRANE(GO:0005789) |  | 536 | 0.25 | 1.38 | 0.064 | 0.409 | 1.000 | 6513 | tags=20%, list=20%, signal=25% |
| 396 | MEMBRANE\_BIOGENESIS(GO:0044091) |  | 17 | 0.46 | 1.38 | 0.080 | 0.412 | 1.000 | 1043 | tags=12%, list=3%, signal=12% |
| 397 | MEMBRANE\_ASSEMBLY(GO:0071709) |  | 17 | 0.46 | 1.38 | 0.080 | 0.411 | 1.000 | 1043 | tags=12%, list=3%, signal=12% |
| 398 | PHOSPHOLIPID\_SCRAMBLING(GO:0017121) |  | 22 | 0.43 | 1.38 | 0.070 | 0.410 | 1.000 | 5211 | tags=23%, list=16%, signal=27% |
| 399 | TRANSFERASE\_ACTIVITY\_\_TRANSFERRING\_ACYL\_GROUPS(GO:0016746) |  | 253 | 0.26 | 1.38 | 0.000 | 0.410 | 1.000 | 4261 | tags=15%, list=13%, signal=17% |
| 400 | LIPID\_TRANSPORT(GO:0006869) |  | 174 | 0.30 | 1.38 | 0.031 | 0.409 | 1.000 | 4608 | tags=21%, list=14%, signal=24% |
| 401 | RESPONSE\_TO\_STEROID\_HORMONE(GO:0048545) |  | 45 | 0.38 | 1.38 | 0.100 | 0.413 | 1.000 | 7020 | tags=38%, list=22%, signal=48% |
| 402 | CELLULAR\_PROTEIN\_LOCALIZATION(GO:0034613) |  | 812 | 0.19 | 1.38 | 0.000 | 0.413 | 1.000 | 3184 | tags=8%, list=10%, signal=9% |
| 403 | NEGATIVE\_REGULATION\_OF\_MRNA\_CATABOLIC\_PROCESS(GO:1902373) |  | 20 | 0.39 | 1.38 | 0.049 | 0.412 | 1.000 | 10234 | tags=45%, list=32%, signal=66% |
| 404 | PROTEIN\_KINASE\_REGULATOR\_ACTIVITY(GO:0019887) |  | 111 | 0.27 | 1.38 | 0.000 | 0.413 | 1.000 | 4879 | tags=14%, list=15%, signal=17% |
| 405 | RESPONSE\_TO\_FUNGUS(GO:0009620) |  | 28 | 0.35 | 1.38 | 0.091 | 0.412 | 1.000 | 8878 | tags=43%, list=28%, signal=59% |
| 406 | LYMPHOCYTE\_MIGRATION(GO:0072676) |  | 36 | 0.45 | 1.38 | 0.000 | 0.411 | 1.000 | 2466 | tags=22%, list=8%, signal=24% |
| 407 | SPHINGOLIPID\_METABOLIC\_PROCESS(GO:0006665) |  | 102 | 0.33 | 1.38 | 0.100 | 0.411 | 1.000 | 5110 | tags=21%, list=16%, signal=24% |
| 408 | DYNEIN\_LIGHT\_INTERMEDIATE\_CHAIN\_BINDING(GO:0051959) |  | 16 | 0.44 | 1.38 | 0.114 | 0.410 | 1.000 | 4068 | tags=19%, list=13%, signal=21% |
| 409 | FORMATION\_OF\_ANATOMICAL\_BOUNDARY(GO:0048859) |  | 15 | 0.51 | 1.37 | 0.088 | 0.412 | 1.000 | 7745 | tags=40%, list=24%, signal=53% |
| 410 | NEGATIVE\_REGULATION\_OF\_AUTOPHAGY(GO:0010507) |  | 18 | 0.35 | 1.37 | 0.020 | 0.413 | 1.000 | 2490 | tags=11%, list=8%, signal=12% |
| 411 | MRNA\_EXPORT\_FROM\_NUCLEUS(GO:0006406) |  | 32 | 0.33 | 1.37 | 0.117 | 0.413 | 1.000 | 3131 | tags=9%, list=10%, signal=10% |
| 412 | MRNA\_CONTAINING\_RIBONUCLEOPROTEIN\_COMPLEX\_EXPORT\_FROM\_NUCLEUS(GO:0071427) |  | 32 | 0.33 | 1.37 | 0.117 | 0.412 | 1.000 | 3131 | tags=9%, list=10%, signal=10% |
| 413 | SWIM\_BLADDER\_DEVELOPMENT(GO:0048794) |  | 26 | 0.44 | 1.37 | 0.065 | 0.412 | 1.000 | 8146 | tags=35%, list=26%, signal=46% |
| 414 | LATE\_ENDOSOME\_MEMBRANE(GO:0031902) |  | 59 | 0.25 | 1.37 | 0.067 | 0.413 | 1.000 | 3552 | tags=12%, list=11%, signal=13% |
| 415 | RAB\_PROTEIN\_SIGNAL\_TRANSDUCTION(GO:0032482) |  | 18 | 0.42 | 1.37 | 0.048 | 0.412 | 1.000 | 6343 | tags=33%, list=20%, signal=42% |
| 416 | MRNA\_STABILIZATION(GO:0048255) |  | 18 | 0.42 | 1.37 | 0.077 | 0.412 | 1.000 | 10234 | tags=44%, list=32%, signal=65% |
| 417 | ORGANIC\_ACID\_TRANSMEMBRANE\_TRANSPORT(GO:1903825) |  | 57 | 0.31 | 1.37 | 0.063 | 0.412 | 1.000 | 2998 | tags=12%, list=9%, signal=14% |
| 418 | CARBOXYLIC\_ACID\_TRANSMEMBRANE\_TRANSPORT(GO:1905039) |  | 57 | 0.31 | 1.37 | 0.063 | 0.411 | 1.000 | 2998 | tags=12%, list=9%, signal=14% |
| 419 | REGULATION\_OF\_IMMUNE\_EFFECTOR\_PROCESS(GO:0002697) |  | 47 | 0.38 | 1.37 | 0.069 | 0.411 | 1.000 | 4083 | tags=23%, list=13%, signal=27% |
| 420 | ESTABLISHMENT\_OF\_PROTEIN\_LOCALIZATION\_TO\_ORGANELLE(GO:0072594) |  | 177 | 0.23 | 1.37 | 0.118 | 0.412 | 1.000 | 4026 | tags=8%, list=13%, signal=10% |
| 421 | SPROUTING\_ANGIOGENESIS(GO:0002040) |  | 127 | 0.27 | 1.37 | 0.031 | 0.412 | 1.000 | 7067 | tags=21%, list=22%, signal=27% |
| 422 | CATALYTIC\_ACTIVITY\_\_ACTING\_ON\_A\_TRNA(GO:0140101) |  | 113 | 0.29 | 1.37 | 0.024 | 0.412 | 1.000 | 3874 | tags=12%, list=12%, signal=13% |
| 423 | CELL\_CELL\_ADHESION(GO:0098609) |  | 346 | 0.26 | 1.37 | 0.000 | 0.411 | 1.000 | 4208 | tags=14%, list=13%, signal=16% |
| 424 | 1\_PHOSPHATIDYLINOSITOL\_3\_KINASE\_REGULATOR\_ACTIVITY(GO:0046935) |  | 18 | 0.49 | 1.37 | 0.080 | 0.410 | 1.000 | 4871 | tags=39%, list=15%, signal=46% |
| 425 | INTRACELLULAR\_TRANSPORT(GO:0046907) |  | 884 | 0.19 | 1.37 | 0.000 | 0.411 | 1.000 | 3184 | tags=8%, list=10%, signal=8% |
| 426 | INTERMEDIATE\_FILAMENT\_BASED\_PROCESS(GO:0045103) |  | 21 | 0.43 | 1.37 | 0.135 | 0.411 | 1.000 | 10716 | tags=52%, list=34%, signal=79% |
| 427 | INTERMEDIATE\_FILAMENT\_CYTOSKELETON\_ORGANIZATION(GO:0045104) |  | 21 | 0.43 | 1.37 | 0.135 | 0.410 | 1.000 | 10716 | tags=52%, list=34%, signal=79% |
| 428 | LONG\_CHAIN\_FATTY\_ACID\_COA\_LIGASE\_ACTIVITY(GO:0004467) |  | 16 | 0.48 | 1.37 | 0.062 | 0.409 | 1.000 | 6267 | tags=38%, list=20%, signal=47% |
| 429 | AROMATIC\_AMINO\_ACID\_FAMILY\_METABOLIC\_PROCESS(GO:0009072) |  | 24 | 0.43 | 1.37 | 0.154 | 0.408 | 1.000 | 7790 | tags=46%, list=24%, signal=61% |
| 430 | OXIDOREDUCTASE\_ACTIVITY\_\_ACTING\_ON\_PAIRED\_DONORS\_\_WITH\_INCORPORATION\_OR\_REDUCTION\_OF\_MOLECULAR\_OXYGEN(GO:0016705) |  | 177 | 0.33 | 1.37 | 0.134 | 0.408 | 1.000 | 6780 | tags=31%, list=21%, signal=39% |
| 431 | VESICLE\_LOCALIZATION(GO:0051648) |  | 162 | 0.27 | 1.37 | 0.000 | 0.408 | 1.000 | 4714 | tags=14%, list=15%, signal=17% |
| 432 | POSITIVE\_REGULATION\_OF\_PHOSPHORUS\_METABOLIC\_PROCESS(GO:0010562) |  | 300 | 0.32 | 1.36 | 0.034 | 0.410 | 1.000 | 7756 | tags=29%, list=24%, signal=38% |
| 433 | POSITIVE\_REGULATION\_OF\_PHOSPHATE\_METABOLIC\_PROCESS(GO:0045937) |  | 300 | 0.32 | 1.36 | 0.034 | 0.409 | 1.000 | 7756 | tags=29%, list=24%, signal=38% |
| 434 | NEURAL\_CREST\_CELL\_MIGRATION(GO:0001755) |  | 100 | 0.33 | 1.36 | 0.000 | 0.410 | 1.000 | 3373 | tags=13%, list=11%, signal=14% |
| 435 | DIOL\_BIOSYNTHETIC\_PROCESS(GO:0034312) |  | 16 | 0.42 | 1.36 | 0.026 | 0.410 | 1.000 | 6682 | tags=31%, list=21%, signal=40% |
| 436 | POSITIVE\_REGULATION\_OF\_PROTEOLYSIS(GO:0045862) |  | 79 | 0.29 | 1.36 | 0.108 | 0.410 | 1.000 | 6262 | tags=23%, list=20%, signal=28% |
| 437 | MEIOTIC\_CELL\_CYCLE\_PROCESS(GO:1903046) |  | 74 | 0.32 | 1.36 | 0.051 | 0.413 | 1.000 | 1739 | tags=11%, list=5%, signal=11% |
| 438 | GLUCOSAMINE\_CONTAINING\_COMPOUND\_METABOLIC\_PROCESS(GO:1901071) |  | 20 | 0.49 | 1.36 | 0.059 | 0.419 | 1.000 | 7006 | tags=40%, list=22%, signal=51% |
| 439 | POSITIVE\_REGULATION\_OF\_CATALYTIC\_ACTIVITY(GO:0043085) |  | 334 | 0.28 | 1.36 | 0.000 | 0.420 | 1.000 | 7908 | tags=28%, list=25%, signal=36% |
| 440 | PROTEIN\_LOCALIZATION\_TO\_MEMBRANE(GO:0072657) |  | 220 | 0.24 | 1.36 | 0.044 | 0.423 | 1.000 | 6659 | tags=20%, list=21%, signal=25% |
| 441 | MESENCHYMAL\_CELL\_DIFFERENTIATION(GO:0048762) |  | 170 | 0.29 | 1.35 | 0.069 | 0.427 | 1.000 | 3614 | tags=11%, list=11%, signal=12% |
| 442 | CYSTEINE\_TYPE\_ENDOPEPTIDASE\_ACTIVITY(GO:0004197) |  | 102 | 0.29 | 1.35 | 0.137 | 0.429 | 1.000 | 4934 | tags=20%, list=15%, signal=23% |
| 443 | CLATHRIN\_COAT(GO:0030118) |  | 35 | 0.31 | 1.35 | 0.050 | 0.430 | 1.000 | 5189 | tags=20%, list=16%, signal=24% |
| 444 | PHOSPHATE\_ION\_TRANSPORT(GO:0006817) |  | 19 | 0.38 | 1.35 | 0.042 | 0.429 | 1.000 | 3517 | tags=16%, list=11%, signal=18% |
| 445 | ORGANONITROGEN\_COMPOUND\_CATABOLIC\_PROCESS(GO:1901565) |  | 731 | 0.20 | 1.35 | 0.000 | 0.432 | 1.000 | 5110 | tags=13%, list=16%, signal=15% |
| 446 | ENDOTHELIAL\_CELL\_DEVELOPMENT(GO:0001885) |  | 29 | 0.45 | 1.35 | 0.157 | 0.432 | 1.000 | 6618 | tags=34%, list=21%, signal=43% |
| 447 | GLYCEROPHOSPHOLIPID\_METABOLIC\_PROCESS(GO:0006650) |  | 187 | 0.27 | 1.35 | 0.080 | 0.432 | 1.000 | 5065 | tags=17%, list=16%, signal=20% |
| 448 | THIOLESTER\_HYDROLASE\_ACTIVITY(GO:0016790) |  | 44 | 0.35 | 1.35 | 0.040 | 0.431 | 1.000 | 5292 | tags=25%, list=17%, signal=30% |
| 449 | IMMUNE\_EFFECTOR\_PROCESS(GO:0002252) |  | 169 | 0.32 | 1.35 | 0.000 | 0.434 | 1.000 | 6666 | tags=30%, list=21%, signal=38% |
| 450 | SPINDLE\_POLE(GO:0000922) |  | 52 | 0.33 | 1.34 | 0.022 | 0.439 | 1.000 | 7859 | tags=33%, list=25%, signal=43% |
| 451 | ORGANIC\_ACID\_METABOLIC\_PROCESS(GO:0006082) |  | 630 | 0.25 | 1.34 | 0.131 | 0.442 | 1.000 | 6016 | tags=21%, list=19%, signal=26% |
| 452 | ENDOPLASMIC\_RETICULUM\_SUBCOMPARTMENT(GO:0098827) |  | 538 | 0.24 | 1.34 | 0.095 | 0.446 | 1.000 | 6513 | tags=20%, list=20%, signal=25% |
| 453 | REGULATION\_OF\_TRANSFERASE\_ACTIVITY(GO:0051338) |  | 232 | 0.29 | 1.34 | 0.000 | 0.445 | 1.000 | 7756 | tags=26%, list=24%, signal=34% |
| 454 | FOCAL\_ADHESION(GO:0005925) |  | 61 | 0.27 | 1.34 | 0.090 | 0.445 | 1.000 | 3862 | tags=15%, list=12%, signal=17% |
| 455 | SERINE\_TYPE\_ENDOPEPTIDASE\_ACTIVITY(GO:0004252) |  | 146 | 0.38 | 1.34 | 0.061 | 0.445 | 1.000 | 7939 | tags=36%, list=25%, signal=48% |
| 456 | POSTSYNAPTIC\_MEMBRANE\_ORGANIZATION(GO:0001941) |  | 18 | 0.50 | 1.34 | 0.122 | 0.444 | 1.000 | 1043 | tags=11%, list=3%, signal=11% |
| 457 | MICROTUBULE\_ORGANIZING\_CENTER(GO:0005815) |  | 285 | 0.24 | 1.34 | 0.000 | 0.445 | 1.000 | 7710 | tags=21%, list=24%, signal=28% |
| 458 | REGULATION\_OF\_PHOSPHATASE\_ACTIVITY(GO:0010921) |  | 22 | 0.31 | 1.34 | 0.128 | 0.446 | 1.000 | 3751 | tags=14%, list=12%, signal=15% |
| 459 | POSITIVE\_REGULATION\_OF\_JAK\_STAT\_CASCADE(GO:0046427) |  | 21 | 0.46 | 1.34 | 0.090 | 0.446 | 1.000 | 3960 | tags=24%, list=12%, signal=27% |
| 460 | POSITIVE\_REGULATION\_OF\_STAT\_CASCADE(GO:1904894) |  | 21 | 0.46 | 1.34 | 0.090 | 0.445 | 1.000 | 3960 | tags=24%, list=12%, signal=27% |
| 461 | POSITIVE\_REGULATION\_OF\_CELLULAR\_PROTEIN\_METABOLIC\_PROCESS(GO:0032270) |  | 390 | 0.26 | 1.34 | 0.000 | 0.444 | 1.000 | 6618 | tags=21%, list=21%, signal=27% |
| 462 | HETEROPHILIC\_CELL\_CELL\_ADHESION\_VIA\_PLASMA\_MEMBRANE\_CELL\_ADHESION\_MOLECULES(GO:0007157) |  | 24 | 0.47 | 1.34 | 0.116 | 0.444 | 1.000 | 3702 | tags=25%, list=12%, signal=28% |
| 463 | OXOACID\_METABOLIC\_PROCESS(GO:0043436) |  | 590 | 0.25 | 1.34 | 0.152 | 0.445 | 1.000 | 6016 | tags=20%, list=19%, signal=25% |
| 464 | SERINE\_TYPE\_PEPTIDASE\_ACTIVITY(GO:0008236) |  | 158 | 0.37 | 1.33 | 0.108 | 0.451 | 1.000 | 7939 | tags=36%, list=25%, signal=48% |
| 465 | SERINE\_HYDROLASE\_ACTIVITY(GO:0017171) |  | 158 | 0.37 | 1.33 | 0.108 | 0.450 | 1.000 | 7939 | tags=36%, list=25%, signal=48% |
| 466 | PROTEIN\_LOCALIZATION(GO:0008104) |  | 1083 | 0.17 | 1.33 | 0.000 | 0.449 | 1.000 | 3184 | tags=8%, list=10%, signal=8% |
| 467 | ANION\_TRANSPORT(GO:0006820) |  | 344 | 0.28 | 1.33 | 0.033 | 0.449 | 1.000 | 4164 | tags=16%, list=13%, signal=18% |
| 468 | UBIQUITIN\_LIKE\_PROTEIN\_LIGASE\_ACTIVITY(GO:0061659) |  | 243 | 0.23 | 1.33 | 0.000 | 0.454 | 1.000 | 4951 | tags=15%, list=16%, signal=18% |
| 469 | NUCLEAR\_SPECK(GO:0016607) |  | 85 | 0.24 | 1.33 | 0.079 | 0.454 | 1.000 | 6954 | tags=19%, list=22%, signal=24% |
| 470 | L\_AMINO\_ACID\_TRANSMEMBRANE\_TRANSPORTER\_ACTIVITY(GO:0015179) |  | 46 | 0.33 | 1.33 | 0.036 | 0.454 | 1.000 | 4862 | tags=20%, list=15%, signal=23% |
| 471 | RESPONSE\_TO\_TUMOR\_NECROSIS\_FACTOR(GO:0034612) |  | 42 | 0.41 | 1.33 | 0.000 | 0.455 | 1.000 | 2466 | tags=19%, list=8%, signal=21% |
| 472 | METENCEPHALON\_DEVELOPMENT(GO:0022037) |  | 57 | 0.29 | 1.33 | 0.108 | 0.456 | 1.000 | 3519 | tags=11%, list=11%, signal=12% |
| 473 | LEUKOCYTE\_CELL\_CELL\_ADHESION(GO:0007159) |  | 54 | 0.37 | 1.33 | 0.039 | 0.456 | 1.000 | 3523 | tags=28%, list=11%, signal=31% |
| 474 | DEFENSE\_RESPONSE\_TO\_VIRUS(GO:0051607) |  | 57 | 0.41 | 1.33 | 0.014 | 0.458 | 1.000 | 9294 | tags=42%, list=29%, signal=59% |
| 475 | POSITIVE\_REGULATION\_OF\_PHOSPHORYLATION(GO:0042327) |  | 290 | 0.32 | 1.33 | 0.035 | 0.457 | 1.000 | 7756 | tags=29%, list=24%, signal=38% |
| 476 | NEGATIVE\_REGULATION\_OF\_SMOOTHENED\_SIGNALING\_PATHWAY(GO:0045879) |  | 22 | 0.38 | 1.33 | 0.074 | 0.459 | 1.000 | 6244 | tags=23%, list=20%, signal=28% |
| 477 | STEM\_CELL\_DIFFERENTIATION(GO:0048863) |  | 247 | 0.24 | 1.33 | 0.068 | 0.461 | 1.000 | 3614 | tags=10%, list=11%, signal=11% |
| 478 | MITOCHONDRIAL\_RESPIRATORY\_CHAIN\_COMPLEX\_I(GO:0005747) |  | 36 | 0.37 | 1.33 | 0.207 | 0.460 | 1.000 | 1769 | tags=6%, list=6%, signal=6% |
| 479 | NADH\_DEHYDROGENASE\_COMPLEX(GO:0030964) |  | 36 | 0.37 | 1.33 | 0.207 | 0.459 | 1.000 | 1769 | tags=6%, list=6%, signal=6% |
| 480 | RESPIRATORY\_CHAIN\_COMPLEX\_I(GO:0045271) |  | 36 | 0.37 | 1.33 | 0.207 | 0.458 | 1.000 | 1769 | tags=6%, list=6%, signal=6% |
| 481 | MONOCARBOXYLIC\_ACID\_TRANSPORT(GO:0015718) |  | 64 | 0.33 | 1.32 | 0.114 | 0.460 | 1.000 | 4164 | tags=22%, list=13%, signal=25% |
| 482 | CARBOHYDRATE\_TRANSMEMBRANE\_TRANSPORTER\_ACTIVITY(GO:0015144) |  | 29 | 0.41 | 1.32 | 0.103 | 0.459 | 1.000 | 3397 | tags=21%, list=11%, signal=23% |
| 483 | POSITIVE\_REGULATION\_OF\_PROTEIN\_METABOLIC\_PROCESS(GO:0051247) |  | 411 | 0.25 | 1.32 | 0.000 | 0.459 | 1.000 | 7105 | tags=22%, list=22%, signal=28% |
| 484 | RESPONSE\_TO\_STRESS(GO:0006950) |  | 1478 | 0.20 | 1.32 | 0.000 | 0.458 | 1.000 | 4462 | tags=14%, list=14%, signal=15% |
| 485 | EMBRYONIC\_HEART\_TUBE\_MORPHOGENESIS(GO:0003143) |  | 140 | 0.27 | 1.32 | 0.030 | 0.458 | 1.000 | 6941 | tags=22%, list=22%, signal=28% |
| 486 | ALPHA\_BETA\_T\_CELL\_ACTIVATION(GO:0046631) |  | 24 | 0.38 | 1.32 | 0.123 | 0.458 | 1.000 | 3387 | tags=17%, list=11%, signal=19% |
| 487 | OSSIFICATION(GO:0001503) |  | 90 | 0.28 | 1.32 | 0.175 | 0.458 | 1.000 | 6845 | tags=23%, list=21%, signal=30% |
| 488 | NUCLEOTIDE\_SUGAR\_BIOSYNTHETIC\_PROCESS(GO:0009226) |  | 18 | 0.46 | 1.32 | 0.133 | 0.457 | 1.000 | 9831 | tags=61%, list=31%, signal=88% |
| 489 | EPITHELIAL\_TO\_MESENCHYMAL\_TRANSITION(GO:0001837) |  | 33 | 0.33 | 1.32 | 0.061 | 0.460 | 1.000 | 1891 | tags=6%, list=6%, signal=6% |
| 490 | RESPONSE\_TO\_ORGANIC\_CYCLIC\_COMPOUND(GO:0014070) |  | 116 | 0.32 | 1.32 | 0.089 | 0.459 | 1.000 | 5036 | tags=19%, list=16%, signal=22% |
| 491 | HISTONE\_DEACETYLASE\_BINDING(GO:0042826) |  | 18 | 0.48 | 1.32 | 0.163 | 0.460 | 1.000 | 10665 | tags=67%, list=33%, signal=100% |
| 492 | REGULATION\_OF\_ALPHA\_BETA\_T\_CELL\_ACTIVATION(GO:0046634) |  | 17 | 0.46 | 1.32 | 0.066 | 0.460 | 1.000 | 3387 | tags=24%, list=11%, signal=26% |
| 493 | REPLICATION\_FORK\_PROCESSING(GO:0031297) |  | 15 | 0.46 | 1.32 | 0.096 | 0.461 | 1.000 | 2730 | tags=27%, list=9%, signal=29% |
| 494 | ENDOTHELIUM\_DEVELOPMENT(GO:0003158) |  | 59 | 0.32 | 1.32 | 0.128 | 0.461 | 1.000 | 6618 | tags=24%, list=21%, signal=30% |
| 495 | EXOCRINE\_PANCREAS\_DEVELOPMENT(GO:0031017) |  | 48 | 0.31 | 1.32 | 0.149 | 0.466 | 1.000 | 3529 | tags=13%, list=11%, signal=14% |
| 496 | EXOCRINE\_SYSTEM\_DEVELOPMENT(GO:0035272) |  | 48 | 0.31 | 1.32 | 0.149 | 0.465 | 1.000 | 3529 | tags=13%, list=11%, signal=14% |
| 497 | NEGATIVE\_REGULATION\_OF\_NEURON\_PROJECTION\_DEVELOPMENT(GO:0010977) |  | 48 | 0.42 | 1.32 | 0.147 | 0.465 | 1.000 | 3433 | tags=15%, list=11%, signal=16% |
| 498 | GLUTAMINE\_FAMILY\_AMINO\_ACID\_CATABOLIC\_PROCESS(GO:0009065) |  | 22 | 0.38 | 1.32 | 0.112 | 0.466 | 1.000 | 6229 | tags=27%, list=20%, signal=34% |
| 499 | PYRIMIDINE\_NUCLEOSIDE\_METABOLIC\_PROCESS(GO:0006213) |  | 30 | 0.35 | 1.32 | 0.036 | 0.466 | 1.000 | 4461 | tags=20%, list=14%, signal=23% |
| 500 | ACETYLTRANSFERASE\_ACTIVITY(GO:0016407) |  | 74 | 0.28 | 1.32 | 0.117 | 0.467 | 1.000 | 4174 | tags=15%, list=13%, signal=17% |
| 501 | LIPID\_LOCALIZATION(GO:0010876) |  | 192 | 0.27 | 1.32 | 0.032 | 0.466 | 1.000 | 4608 | tags=19%, list=14%, signal=22% |
| 502 | NEUROMAST\_DEVELOPMENT(GO:0048884) |  | 76 | 0.31 | 1.31 | 0.072 | 0.469 | 1.000 | 6303 | tags=22%, list=20%, signal=28% |
| 503 | REGULATION\_OF\_CILIUM\_ASSEMBLY(GO:1902017) |  | 19 | 0.36 | 1.31 | 0.189 | 0.470 | 1.000 | 262 | tags=5%, list=1%, signal=5% |
| 504 | NEGATIVE\_REGULATION\_OF\_TRANSLATION(GO:0017148) |  | 96 | 0.31 | 1.31 | 0.113 | 0.469 | 1.000 | 6119 | tags=20%, list=19%, signal=24% |
| 505 | GOLGI\_VESICLE\_TRANSPORT(GO:0048193) |  | 167 | 0.21 | 1.31 | 0.022 | 0.469 | 1.000 | 8016 | tags=25%, list=25%, signal=33% |
| 506 | HISTONE\_DEMETHYLASE\_ACTIVITY(GO:0032452) |  | 25 | 0.36 | 1.31 | 0.249 | 0.469 | 1.000 | 6545 | tags=20%, list=21%, signal=25% |
| 507 | GLYCEROLIPID\_METABOLIC\_PROCESS(GO:0046486) |  | 232 | 0.26 | 1.31 | 0.094 | 0.472 | 1.000 | 5065 | tags=18%, list=16%, signal=21% |
| 508 | CELL\_SURFACE(GO:0009986) |  | 254 | 0.28 | 1.31 | 0.029 | 0.472 | 1.000 | 3950 | tags=19%, list=12%, signal=22% |
| 509 | DNA\_REPAIR(GO:0006281) |  | 311 | 0.25 | 1.31 | 0.091 | 0.473 | 1.000 | 3924 | tags=13%, list=12%, signal=15% |
| 510 | POSITIVE\_REGULATION\_OF\_REPRODUCTIVE\_PROCESS(GO:2000243) |  | 22 | 0.45 | 1.31 | 0.062 | 0.472 | 1.000 | 6892 | tags=41%, list=22%, signal=52% |
| 511 | ANTIGEN\_PROCESSING\_AND\_PRESENTATION(GO:0019882) |  | 25 | 0.41 | 1.31 | 0.100 | 0.472 | 1.000 | 2107 | tags=24%, list=7%, signal=26% |
| 512 | REGULATION\_OF\_CARTILAGE\_DEVELOPMENT(GO:0061035) |  | 21 | 0.34 | 1.31 | 0.143 | 0.472 | 1.000 | 262 | tags=5%, list=1%, signal=5% |
| 513 | CYTOKINE\_BINDING(GO:0019955) |  | 76 | 0.33 | 1.31 | 0.077 | 0.475 | 1.000 | 4744 | tags=22%, list=15%, signal=26% |
| 514 | ORGANELLE\_LOCALIZATION(GO:0051640) |  | 306 | 0.21 | 1.31 | 0.035 | 0.476 | 1.000 | 5079 | tags=12%, list=16%, signal=14% |
| 515 | NUCLEAR\_DIVISION(GO:0000280) |  | 177 | 0.26 | 1.31 | 0.077 | 0.476 | 1.000 | 8998 | tags=28%, list=28%, signal=38% |
| 516 | PERIPHERAL\_NERVOUS\_SYSTEM\_NEURON\_DIFFERENTIATION(GO:0048934) |  | 21 | 0.37 | 1.30 | 0.216 | 0.480 | 1.000 | 2065 | tags=10%, list=6%, signal=10% |
| 517 | PERIPHERAL\_NERVOUS\_SYSTEM\_NEURON\_DEVELOPMENT(GO:0048935) |  | 21 | 0.37 | 1.30 | 0.216 | 0.479 | 1.000 | 2065 | tags=10%, list=6%, signal=10% |
| 518 | SERINE\_THREONINE\_PROTEIN\_KINASE\_COMPLEX(GO:1902554) |  | 53 | 0.24 | 1.30 | 0.000 | 0.479 | 1.000 | 4932 | tags=17%, list=15%, signal=20% |
| 519 | ACTIN\_CYTOSKELETON\_REORGANIZATION(GO:0031532) |  | 28 | 0.37 | 1.30 | 0.168 | 0.479 | 1.000 | 3565 | tags=18%, list=11%, signal=20% |
| 520 | CELL\_SUBSTRATE\_ADHERENS\_JUNCTION(GO:0005924) |  | 62 | 0.26 | 1.30 | 0.128 | 0.481 | 1.000 | 3862 | tags=15%, list=12%, signal=16% |
| 521 | NEGATIVE\_REGULATION\_OF\_GENE\_EXPRESSION\_\_EPIGENETIC(GO:0045814) |  | 17 | 0.42 | 1.30 | 0.193 | 0.480 | 1.000 | 3229 | tags=12%, list=10%, signal=13% |
| 522 | NEGATIVE\_REGULATION\_OF\_PHOSPHORUS\_METABOLIC\_PROCESS(GO:0010563) |  | 125 | 0.26 | 1.30 | 0.099 | 0.481 | 1.000 | 6847 | tags=22%, list=21%, signal=27% |
| 523 | NEGATIVE\_REGULATION\_OF\_PHOSPHATE\_METABOLIC\_PROCESS(GO:0045936) |  | 125 | 0.26 | 1.30 | 0.099 | 0.480 | 1.000 | 6847 | tags=22%, list=21%, signal=27% |
| 524 | TRANSITION\_METAL\_ION\_BINDING(GO:0046914) |  | 949 | 0.22 | 1.30 | 0.000 | 0.482 | 1.000 | 6729 | tags=21%, list=21%, signal=25% |
| 525 | CELL\_ADHESION(GO:0007155) |  | 658 | 0.23 | 1.30 | 0.029 | 0.484 | 1.000 | 4108 | tags=13%, list=13%, signal=15% |
| 526 | BIOLOGICAL\_ADHESION(GO:0022610) |  | 658 | 0.23 | 1.30 | 0.029 | 0.483 | 1.000 | 4108 | tags=13%, list=13%, signal=15% |
| 527 | ORGANIC\_SUBSTANCE\_CATABOLIC\_PROCESS(GO:1901575) |  | 1140 | 0.20 | 1.30 | 0.016 | 0.485 | 1.000 | 5110 | tags=13%, list=16%, signal=15% |
| 528 | LIPID\_METABOLIC\_PROCESS(GO:0006629) |  | 784 | 0.26 | 1.30 | 0.138 | 0.485 | 1.000 | 5110 | tags=18%, list=16%, signal=20% |
| 529 | RETROGRADE\_TRANSPORT\_\_ENDOSOME\_TO\_GOLGI(GO:0042147) |  | 39 | 0.29 | 1.30 | 0.145 | 0.484 | 1.000 | 5282 | tags=18%, list=17%, signal=21% |
| 530 | CARBOHYDRATE\_DERIVATIVE\_CATABOLIC\_PROCESS(GO:1901136) |  | 98 | 0.31 | 1.30 | 0.068 | 0.485 | 1.000 | 6288 | tags=26%, list=20%, signal=32% |
| 531 | ORGANONITROGEN\_COMPOUND\_METABOLIC\_PROCESS(GO:1901564) |  | 4445 | 0.18 | 1.30 | 0.000 | 0.488 | 1.000 | 5115 | tags=13%, list=16%, signal=13% |
| 532 | DNA\_REPLICATION(GO:0006260) |  | 129 | 0.27 | 1.30 | 0.090 | 0.487 | 1.000 | 9643 | tags=34%, list=30%, signal=49% |
| 533 | NUCLEAR\_REPLICATION\_FORK(GO:0043596) |  | 21 | 0.38 | 1.29 | 0.188 | 0.493 | 1.000 | 11051 | tags=48%, list=35%, signal=73% |
| 534 | VASCULAR\_ENDOTHELIAL\_GROWTH\_FACTOR\_RECEPTOR\_SIGNALING\_PATHWAY(GO:0048010) |  | 29 | 0.40 | 1.29 | 0.090 | 0.498 | 1.000 | 13133 | tags=66%, list=41%, signal=111% |
| 535 | POSITIVE\_REGULATION\_OF\_PROTEIN\_MODIFICATION\_PROCESS(GO:0031401) |  | 284 | 0.28 | 1.29 | 0.068 | 0.498 | 1.000 | 7003 | tags=24%, list=22%, signal=30% |
| 536 | POSITIVE\_REGULATION\_OF\_MULTI\_ORGANISM\_PROCESS(GO:0043902) |  | 21 | 0.44 | 1.29 | 0.089 | 0.498 | 1.000 | 4988 | tags=29%, list=16%, signal=34% |
| 537 | RESPONSE\_TO\_ENDOPLASMIC\_RETICULUM\_STRESS(GO:0034976) |  | 114 | 0.23 | 1.29 | 0.143 | 0.497 | 1.000 | 2243 | tags=7%, list=7%, signal=8% |
| 538 | CELL\_ADHESION\_MOLECULE\_BINDING(GO:0050839) |  | 108 | 0.24 | 1.29 | 0.135 | 0.497 | 1.000 | 3689 | tags=11%, list=12%, signal=13% |
| 539 | PEPTIDE\_TRANSMEMBRANE\_TRANSPORTER\_ACTIVITY(GO:1904680) |  | 18 | 0.49 | 1.29 | 0.202 | 0.496 | 1.000 | 9478 | tags=50%, list=30%, signal=71% |
| 540 | REGULATION\_OF\_MRNA\_PROCESSING(GO:0050684) |  | 85 | 0.24 | 1.29 | 0.186 | 0.499 | 1.000 | 7521 | tags=19%, list=24%, signal=25% |
| 541 | REGULATION\_OF\_MRNA\_CATABOLIC\_PROCESS(GO:0061013) |  | 54 | 0.27 | 1.29 | 0.160 | 0.498 | 1.000 | 7888 | tags=24%, list=25%, signal=32% |
| 542 | RESPONSE\_TO\_CYTOKINE(GO:0034097) |  | 161 | 0.32 | 1.29 | 0.000 | 0.497 | 1.000 | 5154 | tags=25%, list=16%, signal=29% |
| 543 | INTRINSIC\_COMPONENT\_OF\_POSTSYNAPTIC\_MEMBRANE(GO:0098936) |  | 18 | 0.45 | 1.29 | 0.127 | 0.497 | 1.000 | 1043 | tags=11%, list=3%, signal=11% |
| 544 | INTEGRAL\_COMPONENT\_OF\_POSTSYNAPTIC\_MEMBRANE(GO:0099055) |  | 18 | 0.45 | 1.29 | 0.127 | 0.496 | 1.000 | 1043 | tags=11%, list=3%, signal=11% |
| 545 | MEIOTIC\_CELL\_CYCLE(GO:0051321) |  | 103 | 0.30 | 1.29 | 0.065 | 0.500 | 1.000 | 10059 | tags=39%, list=32%, signal=57% |
| 546 | CENTRIOLE(GO:0005814) |  | 49 | 0.33 | 1.29 | 0.171 | 0.499 | 1.000 | 2268 | tags=12%, list=7%, signal=13% |
| 547 | POSITIVE\_REGULATION\_OF\_CELLULAR\_AMIDE\_METABOLIC\_PROCESS(GO:0034250) |  | 40 | 0.30 | 1.29 | 0.178 | 0.499 | 1.000 | 54 | tags=3%, list=0%, signal=3% |
| 548 | DITERPENOID\_METABOLIC\_PROCESS(GO:0016101) |  | 36 | 0.39 | 1.28 | 0.198 | 0.503 | 1.000 | 3636 | tags=22%, list=11%, signal=25% |
| 549 | RESPONSE\_TO\_ESTROGEN(GO:0043627) |  | 29 | 0.48 | 1.28 | 0.191 | 0.503 | 1.000 | 7380 | tags=45%, list=23%, signal=58% |
| 550 | REGULATION\_OF\_CELL\_CYCLE(GO:0051726) |  | 377 | 0.23 | 1.28 | 0.051 | 0.502 | 1.000 | 7442 | tags=24%, list=23%, signal=31% |
| 551 | REGULATION\_OF\_LIPID\_BIOSYNTHETIC\_PROCESS(GO:0046890) |  | 35 | 0.27 | 1.28 | 0.168 | 0.502 | 1.000 | 1051 | tags=6%, list=3%, signal=6% |
| 552 | TRANSCRIPTION\_ELONGATION\_FACTOR\_COMPLEX(GO:0008023) |  | 37 | 0.34 | 1.28 | 0.254 | 0.502 | 1.000 | 594 | tags=5%, list=2%, signal=6% |
| 553 | REGULATION\_OF\_MULTI\_ORGANISM\_PROCESS(GO:0043900) |  | 40 | 0.35 | 1.28 | 0.069 | 0.502 | 1.000 | 4988 | tags=20%, list=16%, signal=24% |
| 554 | CATALYTIC\_ACTIVITY\_\_ACTING\_ON\_A\_PROTEIN(GO:0140096) |  | 2142 | 0.20 | 1.28 | 0.000 | 0.501 | 1.000 | 7649 | tags=22%, list=24%, signal=27% |
| 555 | LIGAND\_GATED\_CALCIUM\_CHANNEL\_ACTIVITY(GO:0099604) |  | 17 | 0.49 | 1.28 | 0.179 | 0.501 | 1.000 | 11736 | tags=65%, list=37%, signal=102% |
| 556 | REGULATION\_OF\_PROTEIN\_COMPLEX\_DISASSEMBLY(GO:0043244) |  | 68 | 0.24 | 1.28 | 0.092 | 0.501 | 1.000 | 3342 | tags=10%, list=10%, signal=11% |
| 557 | NEUROMAST\_HAIR\_CELL\_DEVELOPMENT(GO:0035675) |  | 15 | 0.43 | 1.28 | 0.100 | 0.501 | 1.000 | 9719 | tags=47%, list=30%, signal=67% |
| 558 | MODIFIED\_AMINO\_ACID\_BINDING(GO:0072341) |  | 34 | 0.39 | 1.28 | 0.185 | 0.502 | 1.000 | 2276 | tags=12%, list=7%, signal=13% |
| 559 | REGULATION\_OF\_REPRODUCTIVE\_PROCESS(GO:2000241) |  | 36 | 0.41 | 1.28 | 0.158 | 0.501 | 1.000 | 4988 | tags=28%, list=16%, signal=33% |
| 560 | RETINOID\_METABOLIC\_PROCESS(GO:0001523) |  | 35 | 0.39 | 1.28 | 0.198 | 0.500 | 1.000 | 3636 | tags=23%, list=11%, signal=26% |
| 561 | NEGATIVE\_REGULATION\_OF\_MOLECULAR\_FUNCTION(GO:0044092) |  | 218 | 0.26 | 1.28 | 0.140 | 0.500 | 1.000 | 6847 | tags=25%, list=21%, signal=31% |
| 562 | NCRNA\_CATABOLIC\_PROCESS(GO:0034661) |  | 18 | 0.41 | 1.28 | 0.231 | 0.500 | 1.000 | 1460 | tags=11%, list=5%, signal=12% |
| 563 | EXOPEPTIDASE\_ACTIVITY(GO:0008238) |  | 96 | 0.34 | 1.28 | 0.204 | 0.499 | 1.000 | 4279 | tags=18%, list=13%, signal=20% |
| 564 | RNA\_POLYADENYLATION(GO:0043631) |  | 21 | 0.34 | 1.28 | 0.135 | 0.499 | 1.000 | 944 | tags=5%, list=3%, signal=5% |
| 565 | NEGATIVE\_REGULATION\_OF\_CELLULAR\_AMIDE\_METABOLIC\_PROCESS(GO:0034249) |  | 99 | 0.30 | 1.28 | 0.112 | 0.498 | 1.000 | 6119 | tags=19%, list=19%, signal=24% |
| 566 | NEGATIVE\_REGULATION\_OF\_RNA\_CATABOLIC\_PROCESS(GO:1902369) |  | 23 | 0.34 | 1.28 | 0.132 | 0.499 | 1.000 | 10234 | tags=43%, list=32%, signal=64% |
| 567 | NEUREXIN\_FAMILY\_PROTEIN\_BINDING(GO:0042043) |  | 15 | 0.49 | 1.28 | 0.188 | 0.499 | 1.000 | 7548 | tags=40%, list=24%, signal=52% |
| 568 | CELLULAR\_RESPONSE\_TO\_STRESS(GO:0033554) |  | 762 | 0.19 | 1.28 | 0.025 | 0.499 | 1.000 | 4196 | tags=11%, list=13%, signal=13% |
| 569 | MONOCARBOXYLIC\_ACID\_TRANSMEMBRANE\_TRANSPORTER\_ACTIVITY(GO:0008028) |  | 50 | 0.35 | 1.28 | 0.136 | 0.500 | 1.000 | 3749 | tags=18%, list=12%, signal=20% |
| 570 | AMINO\_ACID\_TRANSMEMBRANE\_TRANSPORTER\_ACTIVITY(GO:0015171) |  | 73 | 0.29 | 1.28 | 0.050 | 0.500 | 1.000 | 5262 | tags=16%, list=16%, signal=20% |
| 571 | RESPONSE\_TO\_LIPID(GO:0033993) |  | 140 | 0.27 | 1.28 | 0.037 | 0.500 | 1.000 | 7381 | tags=30%, list=23%, signal=39% |
| 572 | LIGASE\_ACTIVITY\_\_FORMING\_CARBON\_SULFUR\_BONDS(GO:0016877) |  | 36 | 0.31 | 1.28 | 0.170 | 0.500 | 1.000 | 7611 | tags=31%, list=24%, signal=40% |
| 573 | POSITIVE\_REGULATION\_OF\_GROWTH(GO:0045927) |  | 26 | 0.35 | 1.28 | 0.115 | 0.500 | 1.000 | 3491 | tags=15%, list=11%, signal=17% |
| 574 | PHOSPHATIDYLINOSITOL\_3\_KINASE\_REGULATOR\_ACTIVITY(GO:0035014) |  | 20 | 0.45 | 1.28 | 0.143 | 0.499 | 1.000 | 4871 | tags=35%, list=15%, signal=41% |
| 575 | ER\_TO\_GOLGI\_VESICLE\_MEDIATED\_TRANSPORT(GO:0006888) |  | 83 | 0.22 | 1.28 | 0.101 | 0.500 | 1.000 | 8455 | tags=23%, list=27%, signal=31% |
| 576 | CHEMOKINE\_MEDIATED\_SIGNALING\_PATHWAY(GO:0070098) |  | 31 | 0.44 | 1.27 | 0.138 | 0.501 | 1.000 | 5055 | tags=32%, list=16%, signal=38% |
| 577 | REGULATION\_OF\_BONE\_MINERALIZATION(GO:0030500) |  | 20 | 0.36 | 1.27 | 0.146 | 0.500 | 1.000 | 6409 | tags=35%, list=20%, signal=44% |
| 578 | REGULATION\_OF\_BIOMINERAL\_TISSUE\_DEVELOPMENT(GO:0070167) |  | 20 | 0.36 | 1.27 | 0.146 | 0.499 | 1.000 | 6409 | tags=35%, list=20%, signal=44% |
| 579 | REGULATION\_OF\_SYNAPSE\_ASSEMBLY(GO:0051963) |  | 18 | 0.49 | 1.27 | 0.164 | 0.500 | 1.000 | 953 | tags=11%, list=3%, signal=11% |
| 580 | PURINE\_CONTAINING\_COMPOUND\_CATABOLIC\_PROCESS(GO:0072523) |  | 30 | 0.40 | 1.27 | 0.082 | 0.500 | 1.000 | 9805 | tags=47%, list=31%, signal=67% |
| 581 | NEGATIVE\_REGULATION\_OF\_INTRACELLULAR\_SIGNAL\_TRANSDUCTION(GO:1902532) |  | 135 | 0.24 | 1.27 | 0.000 | 0.500 | 1.000 | 4682 | tags=18%, list=15%, signal=21% |
| 582 | SECRETORY\_GRANULE(GO:0030141) |  | 54 | 0.36 | 1.27 | 0.178 | 0.501 | 1.000 | 3842 | tags=17%, list=12%, signal=19% |
| 583 | DOUBLE\_STRAND\_BREAK\_REPAIR(GO:0006302) |  | 142 | 0.27 | 1.27 | 0.105 | 0.501 | 1.000 | 3853 | tags=18%, list=12%, signal=20% |
| 584 | INTEGRAL\_COMPONENT\_OF\_GOLGI\_MEMBRANE(GO:0030173) |  | 48 | 0.31 | 1.27 | 0.102 | 0.502 | 1.000 | 10478 | tags=44%, list=33%, signal=65% |
| 585 | INTRINSIC\_COMPONENT\_OF\_GOLGI\_MEMBRANE(GO:0031228) |  | 48 | 0.31 | 1.27 | 0.102 | 0.501 | 1.000 | 10478 | tags=44%, list=33%, signal=65% |
| 586 | AMINO\_SUGAR\_METABOLIC\_PROCESS(GO:0006040) |  | 32 | 0.40 | 1.27 | 0.165 | 0.501 | 1.000 | 7570 | tags=41%, list=24%, signal=53% |
| 587 | EXTRACELLULAR\_REGION\_PART(GO:0044421) |  | 957 | 0.26 | 1.27 | 0.078 | 0.500 | 1.000 | 4503 | tags=17%, list=14%, signal=19% |
| 588 | NEGATIVE\_REGULATION\_OF\_MONONUCLEAR\_CELL\_PROLIFERATION(GO:0032945) |  | 20 | 0.46 | 1.27 | 0.085 | 0.500 | 1.000 | 3387 | tags=30%, list=11%, signal=34% |
| 589 | NEGATIVE\_REGULATION\_OF\_LYMPHOCYTE\_PROLIFERATION(GO:0050672) |  | 20 | 0.46 | 1.27 | 0.085 | 0.499 | 1.000 | 3387 | tags=30%, list=11%, signal=34% |
| 590 | NEGATIVE\_REGULATION\_OF\_LEUKOCYTE\_PROLIFERATION(GO:0070664) |  | 20 | 0.46 | 1.27 | 0.085 | 0.498 | 1.000 | 3387 | tags=30%, list=11%, signal=34% |
| 591 | REGULATION\_OF\_MAPK\_CASCADE(GO:0043408) |  | 184 | 0.29 | 1.27 | 0.000 | 0.498 | 1.000 | 4422 | tags=20%, list=14%, signal=23% |
| 592 | RNA\_HELICASE\_ACTIVITY(GO:0003724) |  | 59 | 0.25 | 1.27 | 0.185 | 0.497 | 1.000 | 5797 | tags=15%, list=18%, signal=19% |
| 593 | REGULATION\_OF\_CYTOKINE\_MEDIATED\_SIGNALING\_PATHWAY(GO:0001959) |  | 16 | 0.44 | 1.27 | 0.100 | 0.496 | 1.000 | 5421 | tags=38%, list=17%, signal=45% |
| 594 | REGULATION\_OF\_RESPONSE\_TO\_CYTOKINE\_STIMULUS(GO:0060759) |  | 16 | 0.44 | 1.27 | 0.100 | 0.496 | 1.000 | 5421 | tags=38%, list=17%, signal=45% |
| 595 | OXIDOREDUCTASE\_ACTIVITY\_\_ACTING\_ON\_THE\_ALDEHYDE\_OR\_OXO\_GROUP\_OF\_DONORS(GO:0016903) |  | 37 | 0.31 | 1.27 | 0.232 | 0.496 | 1.000 | 5134 | tags=24%, list=16%, signal=29% |
| 596 | RNA\_3'\_END\_PROCESSING(GO:0031123) |  | 64 | 0.25 | 1.27 | 0.221 | 0.496 | 1.000 | 1460 | tags=5%, list=5%, signal=5% |
| 597 | GLUTAMINE\_FAMILY\_AMINO\_ACID\_BIOSYNTHETIC\_PROCESS(GO:0009084) |  | 18 | 0.42 | 1.27 | 0.208 | 0.495 | 1.000 | 6095 | tags=22%, list=19%, signal=27% |
| 598 | INTRINSIC\_COMPONENT\_OF\_SYNAPTIC\_MEMBRANE(GO:0099240) |  | 23 | 0.40 | 1.27 | 0.109 | 0.496 | 1.000 | 1043 | tags=9%, list=3%, signal=9% |
| 599 | INTEGRAL\_COMPONENT\_OF\_SYNAPTIC\_MEMBRANE(GO:0099699) |  | 23 | 0.40 | 1.27 | 0.109 | 0.495 | 1.000 | 1043 | tags=9%, list=3%, signal=9% |
| 600 | NEGATIVE\_REGULATION\_OF\_PHOSPHORYLATION(GO:0042326) |  | 97 | 0.26 | 1.27 | 0.091 | 0.494 | 1.000 | 5144 | tags=16%, list=16%, signal=20% |
| 601 | POSITIVE\_REGULATION\_OF\_PROTEIN\_PHOSPHORYLATION(GO:0001934) |  | 239 | 0.29 | 1.27 | 0.066 | 0.496 | 1.000 | 7105 | tags=26%, list=22%, signal=33% |
| 602 | CELL\_CYCLE\_PROCESS(GO:0022402) |  | 487 | 0.20 | 1.27 | 0.101 | 0.496 | 1.000 | 7495 | tags=20%, list=24%, signal=25% |
| 603 | DEFENSE\_RESPONSE\_TO\_OTHER\_ORGANISM(GO:0098542) |  | 141 | 0.29 | 1.27 | 0.000 | 0.496 | 1.000 | 6543 | tags=28%, list=21%, signal=35% |
| 604 | CELLULAR\_RESPONSE\_TO\_RADIATION(GO:0071478) |  | 57 | 0.35 | 1.27 | 0.175 | 0.497 | 1.000 | 5941 | tags=25%, list=19%, signal=30% |
| 605 | REGULATION\_OF\_LEUKOCYTE\_CELL\_CELL\_ADHESION(GO:1903037) |  | 49 | 0.36 | 1.27 | 0.163 | 0.497 | 1.000 | 3523 | tags=27%, list=11%, signal=30% |
| 606 | CELL\_CYCLE(GO:0007049) |  | 826 | 0.20 | 1.27 | 0.041 | 0.496 | 1.000 | 7495 | tags=21%, list=24%, signal=27% |
| 607 | AMIDE\_TRANSMEMBRANE\_TRANSPORTER\_ACTIVITY(GO:0042887) |  | 35 | 0.35 | 1.27 | 0.171 | 0.497 | 1.000 | 9688 | tags=43%, list=30%, signal=61% |
| 608 | CELLULAR\_AMINE\_METABOLIC\_PROCESS(GO:0044106) |  | 35 | 0.33 | 1.27 | 0.172 | 0.497 | 1.000 | 2458 | tags=14%, list=8%, signal=15% |
| 609 | PROTEIN\_TRANSPORTER\_ACTIVITY(GO:0008565) |  | 21 | 0.38 | 1.27 | 0.227 | 0.497 | 1.000 | 7424 | tags=33%, list=23%, signal=43% |
| 610 | CARBOHYDRATE\_DERIVATIVE\_TRANSPORT(GO:1901264) |  | 33 | 0.34 | 1.26 | 0.195 | 0.498 | 1.000 | 9887 | tags=36%, list=31%, signal=53% |
| 611 | NEGATIVE\_REGULATION\_OF\_CELL\_PROJECTION\_ORGANIZATION(GO:0031345) |  | 51 | 0.40 | 1.26 | 0.178 | 0.498 | 1.000 | 1084 | tags=10%, list=3%, signal=10% |
| 612 | ISOPRENOID\_METABOLIC\_PROCESS(GO:0006720) |  | 56 | 0.32 | 1.26 | 0.246 | 0.498 | 1.000 | 3636 | tags=16%, list=11%, signal=18% |
| 613 | PYRIMIDINE\_RIBONUCLEOSIDE\_METABOLIC\_PROCESS(GO:0046131) |  | 28 | 0.34 | 1.26 | 0.118 | 0.499 | 1.000 | 4461 | tags=18%, list=14%, signal=21% |
| 614 | ACTIN\_BASED\_CELL\_PROJECTION(GO:0098858) |  | 70 | 0.24 | 1.26 | 0.060 | 0.499 | 1.000 | 4479 | tags=16%, list=14%, signal=18% |
| 615 | TRNA\_BINDING(GO:0000049) |  | 40 | 0.25 | 1.26 | 0.101 | 0.499 | 1.000 | 5385 | tags=18%, list=17%, signal=21% |
| 616 | ORGANIC\_ANION\_TRANSMEMBRANE\_TRANSPORTER\_ACTIVITY(GO:0008514) |  | 177 | 0.28 | 1.26 | 0.082 | 0.499 | 1.000 | 3829 | tags=15%, list=12%, signal=17% |
| 617 | PEPTIDYL\_AMINO\_ACID\_MODIFICATION(GO:0018193) |  | 481 | 0.20 | 1.26 | 0.062 | 0.501 | 1.000 | 4532 | tags=11%, list=14%, signal=13% |
| 618 | REGULATION\_OF\_INFLAMMATORY\_RESPONSE(GO:0050727) |  | 45 | 0.30 | 1.26 | 0.083 | 0.502 | 1.000 | 2608 | tags=18%, list=8%, signal=19% |
| 619 | POSITIVE\_REGULATION\_OF\_CYTOKINE\_PRODUCTION(GO:0001819) |  | 46 | 0.36 | 1.26 | 0.170 | 0.502 | 1.000 | 3950 | tags=22%, list=12%, signal=25% |
| 620 | CHROMOSOME(GO:0005694) |  | 508 | 0.21 | 1.26 | 0.090 | 0.502 | 1.000 | 4256 | tags=10%, list=13%, signal=12% |
| 621 | ESTABLISHMENT\_OF\_PROTEIN\_LOCALIZATION\_TO\_PLASMA\_MEMBRANE(GO:0061951) |  | 22 | 0.37 | 1.26 | 0.100 | 0.502 | 1.000 | 3579 | tags=23%, list=11%, signal=26% |
| 622 | COPII\_COATED\_VESICLE\_BUDDING(GO:0090114) |  | 17 | 0.41 | 1.26 | 0.174 | 0.502 | 1.000 | 4817 | tags=24%, list=15%, signal=28% |
| 623 | MRNA\_POLYADENYLATION(GO:0006378) |  | 19 | 0.36 | 1.26 | 0.217 | 0.504 | 1.000 | 944 | tags=5%, list=3%, signal=5% |
| 624 | TERPENOID\_METABOLIC\_PROCESS(GO:0006721) |  | 40 | 0.36 | 1.26 | 0.254 | 0.504 | 1.000 | 3636 | tags=20%, list=11%, signal=23% |
| 625 | UBIQUITIN\_PROTEIN\_TRANSFERASE\_ACTIVITY(GO:0004842) |  | 367 | 0.20 | 1.26 | 0.000 | 0.506 | 1.000 | 4951 | tags=13%, list=16%, signal=15% |
| 626 | NEGATIVE\_REGULATION\_OF\_NERVOUS\_SYSTEM\_DEVELOPMENT(GO:0051961) |  | 76 | 0.35 | 1.26 | 0.165 | 0.508 | 1.000 | 1084 | tags=8%, list=3%, signal=8% |
| 627 | CLATHRIN\_COATED\_VESICLE(GO:0030136) |  | 58 | 0.27 | 1.25 | 0.115 | 0.509 | 1.000 | 5433 | tags=17%, list=17%, signal=21% |
| 628 | CHEMOKINE\_ACTIVITY(GO:0008009) |  | 37 | 0.41 | 1.25 | 0.127 | 0.509 | 1.000 | 5055 | tags=30%, list=16%, signal=35% |
| 629 | CHEMOKINE\_RECEPTOR\_BINDING(GO:0042379) |  | 37 | 0.41 | 1.25 | 0.127 | 0.508 | 1.000 | 5055 | tags=30%, list=16%, signal=35% |
| 630 | MONOCARBOXYLIC\_ACID\_METABOLIC\_PROCESS(GO:0032787) |  | 280 | 0.27 | 1.25 | 0.219 | 0.508 | 1.000 | 5158 | tags=19%, list=16%, signal=22% |
| 631 | NEGATIVE\_REGULATION\_OF\_CELLULAR\_METABOLIC\_PROCESS(GO:0031324) |  | 757 | 0.20 | 1.25 | 0.118 | 0.510 | 1.000 | 7127 | tags=19%, list=22%, signal=24% |
| 632 | REGULATION\_OF\_CELLULAR\_AMIDE\_METABOLIC\_PROCESS(GO:0034248) |  | 182 | 0.25 | 1.25 | 0.131 | 0.511 | 1.000 | 6119 | tags=14%, list=19%, signal=18% |
| 633 | MICROTUBULE\_POLYMERIZATION(GO:0046785) |  | 23 | 0.30 | 1.25 | 0.195 | 0.511 | 1.000 | 5141 | tags=22%, list=16%, signal=26% |
| 634 | LATERAL\_LINE\_DEVELOPMENT(GO:0048882) |  | 110 | 0.24 | 1.25 | 0.061 | 0.512 | 1.000 | 3433 | tags=12%, list=11%, signal=13% |
| 635 | PROTEIN\_PHOSPHATASE\_REGULATOR\_ACTIVITY(GO:0019888) |  | 68 | 0.24 | 1.25 | 0.091 | 0.512 | 1.000 | 3968 | tags=13%, list=12%, signal=15% |
| 636 | MRNA\_CATABOLIC\_PROCESS(GO:0006402) |  | 122 | 0.21 | 1.25 | 0.164 | 0.511 | 1.000 | 1460 | tags=5%, list=5%, signal=5% |
| 637 | ATPASE\_COMPLEX(GO:1904949) |  | 67 | 0.22 | 1.25 | 0.118 | 0.510 | 1.000 | 9313 | tags=25%, list=29%, signal=36% |
| 638 | ATPASE\_ACTIVITY\_\_COUPLED(GO:0042623) |  | 187 | 0.20 | 1.25 | 0.031 | 0.510 | 1.000 | 6884 | tags=20%, list=22%, signal=26% |
| 639 | SMALL\_MOLECULE\_METABOLIC\_PROCESS(GO:0044281) |  | 1237 | 0.23 | 1.25 | 0.181 | 0.512 | 1.000 | 5190 | tags=16%, list=16%, signal=19% |
| 640 | CYTOKINE\_ACTIVITY(GO:0005125) |  | 127 | 0.33 | 1.25 | 0.068 | 0.512 | 1.000 | 7163 | tags=29%, list=22%, signal=37% |
| 641 | REGULATION\_OF\_NEUROTRANSMITTER\_LEVELS(GO:0001505) |  | 129 | 0.31 | 1.25 | 0.202 | 0.511 | 1.000 | 1898 | tags=9%, list=6%, signal=9% |
| 642 | NEGATIVE\_REGULATION\_OF\_NEURON\_DIFFERENTIATION(GO:0045665) |  | 55 | 0.38 | 1.25 | 0.214 | 0.512 | 1.000 | 1084 | tags=9%, list=3%, signal=9% |
| 643 | COGNITION(GO:0050890) |  | 16 | 0.39 | 1.25 | 0.179 | 0.512 | 1.000 | 4795 | tags=19%, list=15%, signal=22% |
| 644 | HORMONE\_BINDING(GO:0042562) |  | 51 | 0.42 | 1.25 | 0.173 | 0.512 | 1.000 | 6481 | tags=39%, list=20%, signal=49% |
| 645 | ENERGY\_DERIVATION\_BY\_OXIDATION\_OF\_ORGANIC\_COMPOUNDS(GO:0015980) |  | 136 | 0.25 | 1.25 | 0.171 | 0.512 | 1.000 | 3693 | tags=12%, list=12%, signal=13% |
| 646 | CYTOKINE\_MEDIATED\_SIGNALING\_PATHWAY(GO:0019221) |  | 131 | 0.32 | 1.25 | 0.085 | 0.513 | 1.000 | 6504 | tags=31%, list=20%, signal=38% |
| 647 | PROTEIN\_METABOLIC\_PROCESS(GO:0019538) |  | 3666 | 0.17 | 1.25 | 0.000 | 0.513 | 1.000 | 5058 | tags=12%, list=16%, signal=13% |
| 648 | CYTOKINE\_RECEPTOR\_ACTIVITY(GO:0004896) |  | 85 | 0.31 | 1.25 | 0.129 | 0.513 | 1.000 | 4814 | tags=22%, list=15%, signal=26% |
| 649 | REGULATION\_OF\_CIRCADIAN\_RHYTHM(GO:0042752) |  | 34 | 0.38 | 1.25 | 0.203 | 0.513 | 1.000 | 9634 | tags=47%, list=30%, signal=67% |
| 650 | CATABOLIC\_PROCESS(GO:0009056) |  | 1394 | 0.18 | 1.25 | 0.038 | 0.513 | 1.000 | 5110 | tags=13%, list=16%, signal=15% |
| 651 | NEGATIVE\_REGULATION\_OF\_PROTEIN\_PHOSPHORYLATION(GO:0001933) |  | 90 | 0.25 | 1.25 | 0.125 | 0.514 | 1.000 | 5144 | tags=17%, list=16%, signal=20% |
| 652 | HEART\_MORPHOGENESIS(GO:0003007) |  | 227 | 0.25 | 1.25 | 0.105 | 0.513 | 1.000 | 6941 | tags=20%, list=22%, signal=26% |
| 653 | GLUTATHIONE\_METABOLIC\_PROCESS(GO:0006749) |  | 40 | 0.32 | 1.25 | 0.183 | 0.513 | 1.000 | 6138 | tags=23%, list=19%, signal=28% |
| 654 | CAVEOLA(GO:0005901) |  | 32 | 0.29 | 1.24 | 0.116 | 0.515 | 1.000 | 4120 | tags=16%, list=13%, signal=18% |
| 655 | PLASMA\_MEMBRANE\_RAFT(GO:0044853) |  | 32 | 0.29 | 1.24 | 0.116 | 0.514 | 1.000 | 4120 | tags=16%, list=13%, signal=18% |
| 656 | ENDOPLASMIC\_RETICULUM\_PART(GO:0044432) |  | 593 | 0.23 | 1.24 | 0.295 | 0.514 | 1.000 | 4817 | tags=15%, list=15%, signal=18% |
| 657 | DETERMINATION\_OF\_HEART\_LEFT\_RIGHT\_ASYMMETRY(GO:0061371) |  | 177 | 0.24 | 1.24 | 0.119 | 0.514 | 1.000 | 6941 | tags=22%, list=22%, signal=28% |
| 658 | CELL\_REDOX\_HOMEOSTASIS(GO:0045454) |  | 17 | 0.45 | 1.24 | 0.264 | 0.514 | 1.000 | 7445 | tags=35%, list=23%, signal=46% |
| 659 | NEGATIVE\_REGULATION\_OF\_LEUKOCYTE\_ACTIVATION(GO:0002695) |  | 34 | 0.35 | 1.24 | 0.167 | 0.513 | 1.000 | 3387 | tags=24%, list=11%, signal=26% |
| 660 | NEGATIVE\_REGULATION\_OF\_CELL\_ACTIVATION(GO:0050866) |  | 34 | 0.35 | 1.24 | 0.167 | 0.513 | 1.000 | 3387 | tags=24%, list=11%, signal=26% |
| 661 | SWI\_SNF\_COMPLEX(GO:0016514) |  | 20 | 0.43 | 1.24 | 0.310 | 0.512 | 1.000 | 2781 | tags=10%, list=9%, signal=11% |
| 662 | CELLULAR\_MODIFIED\_AMINO\_ACID\_BIOSYNTHETIC\_PROCESS(GO:0042398) |  | 24 | 0.36 | 1.24 | 0.190 | 0.512 | 1.000 | 1508 | tags=13%, list=5%, signal=13% |
| 663 | MANGANESE\_ION\_BINDING(GO:0030145) |  | 25 | 0.34 | 1.24 | 0.182 | 0.512 | 1.000 | 5009 | tags=24%, list=16%, signal=28% |
| 664 | PURINE\_NUCLEOTIDE\_CATABOLIC\_PROCESS(GO:0006195) |  | 20 | 0.43 | 1.24 | 0.253 | 0.513 | 1.000 | 9805 | tags=45%, list=31%, signal=65% |
| 665 | ADAPTIVE\_IMMUNE\_RESPONSE(GO:0002250) |  | 61 | 0.31 | 1.24 | 0.079 | 0.513 | 1.000 | 5842 | tags=31%, list=18%, signal=38% |
| 666 | FATTY\_ACID\_CATABOLIC\_PROCESS(GO:0009062) |  | 51 | 0.33 | 1.24 | 0.237 | 0.515 | 1.000 | 6664 | tags=29%, list=21%, signal=37% |
| 667 | SINGLE\_FERTILIZATION(GO:0007338) |  | 40 | 0.42 | 1.24 | 0.176 | 0.516 | 1.000 | 5268 | tags=30%, list=17%, signal=36% |
| 668 | GALACTOSYLTRANSFERASE\_ACTIVITY(GO:0008378) |  | 20 | 0.44 | 1.24 | 0.286 | 0.517 | 1.000 | 5115 | tags=30%, list=16%, signal=36% |
| 669 | FERTILIZATION(GO:0009566) |  | 42 | 0.43 | 1.24 | 0.115 | 0.516 | 1.000 | 5268 | tags=31%, list=17%, signal=37% |
| 670 | FIN\_REGENERATION(GO:0031101) |  | 84 | 0.26 | 1.24 | 0.194 | 0.518 | 1.000 | 9306 | tags=31%, list=29%, signal=44% |
| 671 | RESPONSE\_TO\_ESTRADIOL(GO:0032355) |  | 22 | 0.49 | 1.24 | 0.168 | 0.518 | 1.000 | 7020 | tags=45%, list=22%, signal=58% |
| 672 | CHROMOSOMAL\_PART(GO:0044427) |  | 442 | 0.21 | 1.24 | 0.129 | 0.517 | 1.000 | 4256 | tags=11%, list=13%, signal=12% |
| 673 | ALPHA\_AMINO\_ACID\_BIOSYNTHETIC\_PROCESS(GO:1901607) |  | 57 | 0.30 | 1.24 | 0.171 | 0.518 | 1.000 | 4000 | tags=16%, list=13%, signal=18% |
| 674 | ENTRAINMENT\_OF\_CIRCADIAN\_CLOCK(GO:0009649) |  | 15 | 0.56 | 1.24 | 0.155 | 0.517 | 1.000 | 10190 | tags=67%, list=32%, signal=98% |
| 675 | ATPASE\_ACTIVITY\_\_COUPLED\_TO\_TRANSMEMBRANE\_MOVEMENT\_OF\_IONS\_\_PHOSPHORYLATIVE\_MECHANISM(GO:0015662) |  | 24 | 0.34 | 1.24 | 0.288 | 0.517 | 1.000 | 6471 | tags=29%, list=20%, signal=37% |
| 676 | NEGATIVE\_REGULATION\_OF\_DEFENSE\_RESPONSE(GO:0031348) |  | 27 | 0.35 | 1.24 | 0.124 | 0.517 | 1.000 | 5421 | tags=26%, list=17%, signal=31% |
| 677 | REGULATION\_OF\_DOUBLE\_STRAND\_BREAK\_REPAIR\_VIA\_HOMOLOGOUS\_RECOMBINATION(GO:0010569) |  | 15 | 0.49 | 1.24 | 0.076 | 0.518 | 1.000 | 11831 | tags=53%, list=37%, signal=85% |
| 678 | NEGATIVE\_REGULATION\_OF\_T\_CELL\_PROLIFERATION(GO:0042130) |  | 18 | 0.47 | 1.24 | 0.159 | 0.518 | 1.000 | 3387 | tags=33%, list=11%, signal=37% |
| 679 | DNA\_INTEGRATION(GO:0015074) |  | 18 | 0.48 | 1.24 | 0.200 | 0.517 | 1.000 | 2186 | tags=56%, list=7%, signal=60% |
| 680 | REGULATION\_OF\_MICROTUBULE\_BASED\_PROCESS(GO:0032886) |  | 97 | 0.23 | 1.24 | 0.088 | 0.517 | 1.000 | 9789 | tags=26%, list=31%, signal=37% |
| 681 | CYTOKINETIC\_PROCESS(GO:0032506) |  | 17 | 0.37 | 1.24 | 0.183 | 0.517 | 1.000 | 402 | tags=6%, list=1%, signal=6% |
| 682 | LYMPHOCYTE\_MEDIATED\_IMMUNITY(GO:0002449) |  | 38 | 0.39 | 1.23 | 0.202 | 0.517 | 1.000 | 5842 | tags=37%, list=18%, signal=45% |
| 683 | NEGATIVE\_REGULATION\_OF\_PROTEIN\_MODIFICATION\_PROCESS(GO:0031400) |  | 127 | 0.22 | 1.23 | 0.101 | 0.517 | 1.000 | 5144 | tags=15%, list=16%, signal=18% |
| 684 | ORGANIC\_ACID\_BINDING(GO:0043177) |  | 115 | 0.26 | 1.23 | 0.160 | 0.517 | 1.000 | 6711 | tags=24%, list=21%, signal=31% |
| 685 | LEUKOCYTE\_MEDIATED\_IMMUNITY(GO:0002443) |  | 49 | 0.37 | 1.23 | 0.209 | 0.517 | 1.000 | 6666 | tags=37%, list=21%, signal=46% |
| 686 | IMMUNOGLOBULIN\_MEDIATED\_IMMUNE\_RESPONSE(GO:0016064) |  | 33 | 0.40 | 1.23 | 0.143 | 0.518 | 1.000 | 1578 | tags=21%, list=5%, signal=22% |
| 687 | B\_CELL\_MEDIATED\_IMMUNITY(GO:0019724) |  | 33 | 0.40 | 1.23 | 0.143 | 0.517 | 1.000 | 1578 | tags=21%, list=5%, signal=22% |
| 688 | NEGATIVE\_REGULATION\_OF\_METABOLIC\_PROCESS(GO:0009892) |  | 868 | 0.18 | 1.23 | 0.089 | 0.517 | 1.000 | 7127 | tags=19%, list=22%, signal=23% |
| 689 | EXTRACELLULAR\_SPACE(GO:0005615) |  | 837 | 0.25 | 1.23 | 0.165 | 0.517 | 1.000 | 4503 | tags=17%, list=14%, signal=19% |
| 690 | CELLULAR\_RESPONSE\_TO\_DNA\_DAMAGE\_STIMULUS(GO:0006974) |  | 408 | 0.22 | 1.23 | 0.122 | 0.518 | 1.000 | 3924 | tags=12%, list=12%, signal=13% |
| 691 | GERM\_CELL\_MIGRATION(GO:0008354) |  | 15 | 0.42 | 1.23 | 0.165 | 0.519 | 1.000 | 1239 | tags=13%, list=4%, signal=14% |
| 692 | T\_CELL\_ACTIVATION(GO:0042110) |  | 103 | 0.28 | 1.23 | 0.170 | 0.521 | 1.000 | 2107 | tags=13%, list=7%, signal=13% |
| 693 | MYELINATION\_IN\_PERIPHERAL\_NERVOUS\_SYSTEM(GO:0022011) |  | 17 | 0.36 | 1.23 | 0.155 | 0.521 | 1.000 | 8021 | tags=29%, list=25%, signal=39% |
| 694 | SEMICIRCULAR\_CANAL\_DEVELOPMENT(GO:0060872) |  | 25 | 0.35 | 1.23 | 0.213 | 0.527 | 1.000 | 5867 | tags=16%, list=18%, signal=20% |
| 695 | NEGATIVE\_REGULATION\_OF\_LYMPHOCYTE\_ACTIVATION(GO:0051250) |  | 32 | 0.35 | 1.23 | 0.140 | 0.527 | 1.000 | 3387 | tags=25%, list=11%, signal=28% |
| 696 | EXTERNAL\_SIDE\_OF\_PLASMA\_MEMBRANE(GO:0009897) |  | 135 | 0.28 | 1.23 | 0.079 | 0.527 | 1.000 | 3950 | tags=23%, list=12%, signal=26% |
| 697 | CELL\_SUBSTRATE\_JUNCTION(GO:0030055) |  | 65 | 0.24 | 1.23 | 0.124 | 0.527 | 1.000 | 3862 | tags=14%, list=12%, signal=16% |
| 698 | SYMBIONT\_PROCESS(GO:0044403) |  | 19 | 0.38 | 1.22 | 0.182 | 0.530 | 1.000 | 5116 | tags=21%, list=16%, signal=25% |
| 699 | HYDROLASE\_ACTIVITY(GO:0016787) |  | 2296 | 0.20 | 1.22 | 0.055 | 0.530 | 1.000 | 5116 | tags=15%, list=16%, signal=16% |
| 700 | DNA\_POLYMERASE\_ACTIVITY(GO:0034061) |  | 20 | 0.28 | 1.22 | 0.167 | 0.529 | 1.000 | 5630 | tags=25%, list=18%, signal=30% |
| 701 | BONE\_MINERALIZATION(GO:0030282) |  | 39 | 0.28 | 1.22 | 0.234 | 0.529 | 1.000 | 6409 | tags=23%, list=20%, signal=29% |
| 702 | CUL4\_RING\_E3\_UBIQUITIN\_LIGASE\_COMPLEX(GO:0080008) |  | 20 | 0.34 | 1.22 | 0.185 | 0.529 | 1.000 | 5540 | tags=25%, list=17%, signal=30% |
| 703 | ACTIN\_FILAMENT\_BUNDLE(GO:0032432) |  | 38 | 0.28 | 1.22 | 0.186 | 0.530 | 1.000 | 4805 | tags=16%, list=15%, signal=19% |
| 704 | PROTEIN\_DOMAIN\_SPECIFIC\_BINDING(GO:0019904) |  | 103 | 0.21 | 1.22 | 0.100 | 0.529 | 1.000 | 7493 | tags=20%, list=23%, signal=27% |
| 705 | VESICLE\_MEDIATED\_TRANSPORT(GO:0016192) |  | 829 | 0.19 | 1.22 | 0.080 | 0.529 | 1.000 | 3000 | tags=7%, list=9%, signal=8% |
| 706 | CLATHRIN\_BINDING(GO:0030276) |  | 58 | 0.31 | 1.22 | 0.241 | 0.531 | 1.000 | 4795 | tags=17%, list=15%, signal=20% |
| 707 | MONOOXYGENASE\_ACTIVITY(GO:0004497) |  | 127 | 0.33 | 1.22 | 0.200 | 0.530 | 1.000 | 6660 | tags=31%, list=21%, signal=40% |
| 708 | VESICLE\_TRANSPORT\_ALONG\_MICROTUBULE(GO:0047496) |  | 19 | 0.40 | 1.22 | 0.234 | 0.530 | 1.000 | 3299 | tags=16%, list=10%, signal=18% |
| 709 | PHAGOCYTOSIS(GO:0006909) |  | 69 | 0.27 | 1.22 | 0.176 | 0.530 | 1.000 | 5592 | tags=16%, list=18%, signal=19% |
| 710 | POSITIVE\_REGULATION\_OF\_MOLECULAR\_FUNCTION(GO:0044093) |  | 402 | 0.24 | 1.22 | 0.050 | 0.531 | 1.000 | 6791 | tags=22%, list=21%, signal=28% |
| 711 | MOLECULAR\_FUNCTION\_REGULATOR(GO:0098772) |  | 1234 | 0.21 | 1.22 | 0.000 | 0.531 | 1.000 | 5109 | tags=14%, list=16%, signal=17% |
| 712 | MESENCHYME\_DEVELOPMENT(GO:0060485) |  | 222 | 0.24 | 1.22 | 0.122 | 0.530 | 1.000 | 4227 | tags=11%, list=13%, signal=13% |
| 713 | CYCLIN\_DEPENDENT\_PROTEIN\_KINASE\_HOLOENZYME\_COMPLEX(GO:0000307) |  | 30 | 0.27 | 1.22 | 0.144 | 0.531 | 1.000 | 3739 | tags=13%, list=12%, signal=15% |
| 714 | PROTEIN\_CYSTEINE\_S\_PALMITOYLTRANSFERASE\_ACTIVITY(GO:0019706) |  | 30 | 0.37 | 1.22 | 0.109 | 0.530 | 1.000 | 8160 | tags=40%, list=26%, signal=54% |
| 715 | PROTEIN\_CYSTEINE\_S\_ACYLTRANSFERASE\_ACTIVITY(GO:0019707) |  | 30 | 0.37 | 1.22 | 0.109 | 0.530 | 1.000 | 8160 | tags=40%, list=26%, signal=54% |
| 716 | POSITIVE\_REGULATION\_OF\_CELL\_ADHESION(GO:0045785) |  | 49 | 0.30 | 1.22 | 0.122 | 0.529 | 1.000 | 4032 | tags=22%, list=13%, signal=26% |
| 717 | PROTEIN\_TYROSINE\_KINASE\_BINDING(GO:1990782) |  | 33 | 0.37 | 1.22 | 0.063 | 0.528 | 1.000 | 10275 | tags=42%, list=32%, signal=63% |
| 718 | NEGATIVE\_REGULATION\_OF\_NITROGEN\_COMPOUND\_METABOLIC\_PROCESS(GO:0051172) |  | 723 | 0.19 | 1.22 | 0.118 | 0.528 | 1.000 | 7127 | tags=19%, list=22%, signal=24% |
| 719 | SECRETORY\_VESICLE(GO:0099503) |  | 174 | 0.27 | 1.22 | 0.121 | 0.528 | 1.000 | 2249 | tags=9%, list=7%, signal=9% |
| 720 | ACTIVATION\_OF\_GTPASE\_ACTIVITY(GO:0090630) |  | 63 | 0.30 | 1.22 | 0.269 | 0.527 | 1.000 | 7836 | tags=29%, list=25%, signal=38% |
| 721 | ACROSOME\_REACTION(GO:0007340) |  | 19 | 0.48 | 1.22 | 0.113 | 0.527 | 1.000 | 5268 | tags=37%, list=17%, signal=44% |
| 722 | MONOCARBOXYLIC\_ACID\_BINDING(GO:0033293) |  | 27 | 0.40 | 1.22 | 0.078 | 0.527 | 1.000 | 7483 | tags=33%, list=23%, signal=44% |
| 723 | METALLOPEPTIDASE\_ACTIVITY(GO:0008237) |  | 168 | 0.26 | 1.22 | 0.189 | 0.527 | 1.000 | 2941 | tags=12%, list=9%, signal=13% |
| 724 | CELLULAR\_RESPONSE\_TO\_ESTROGEN\_STIMULUS(GO:0071391) |  | 25 | 0.47 | 1.22 | 0.233 | 0.534 | 1.000 | 7380 | tags=44%, list=23%, signal=57% |
| 725 | MICROTUBULE\_CYTOSKELETON(GO:0015630) |  | 576 | 0.20 | 1.22 | 0.182 | 0.535 | 1.000 | 7710 | tags=20%, list=24%, signal=26% |
| 726 | POSTTRANSCRIPTIONAL\_REGULATION\_OF\_GENE\_EXPRESSION(GO:0010608) |  | 212 | 0.22 | 1.21 | 0.160 | 0.536 | 1.000 | 6291 | tags=14%, list=20%, signal=18% |
| 727 | EPITHELIAL\_CELL\_PROLIFERATION(GO:0050673) |  | 55 | 0.28 | 1.21 | 0.231 | 0.538 | 1.000 | 5864 | tags=18%, list=18%, signal=22% |
| 728 | LIPID\_BIOSYNTHETIC\_PROCESS(GO:0008610) |  | 335 | 0.25 | 1.21 | 0.240 | 0.537 | 1.000 | 5158 | tags=17%, list=16%, signal=20% |
| 729 | ANTIBIOTIC\_METABOLIC\_PROCESS(GO:0016999) |  | 75 | 0.24 | 1.21 | 0.130 | 0.538 | 1.000 | 5171 | tags=17%, list=16%, signal=21% |
| 730 | DNA\_DAMAGE\_CHECKPOINT(GO:0000077) |  | 38 | 0.30 | 1.21 | 0.164 | 0.538 | 1.000 | 1253 | tags=8%, list=4%, signal=8% |
| 731 | NBAF\_COMPLEX(GO:0071565) |  | 15 | 0.44 | 1.21 | 0.223 | 0.542 | 1.000 | 6063 | tags=27%, list=19%, signal=33% |
| 732 | CELLULAR\_RESPONSE\_TO\_CYTOKINE\_STIMULUS(GO:0071345) |  | 138 | 0.30 | 1.21 | 0.085 | 0.542 | 1.000 | 5154 | tags=25%, list=16%, signal=29% |
| 733 | MAPK\_CASCADE(GO:0000165) |  | 217 | 0.27 | 1.21 | 0.126 | 0.543 | 1.000 | 4476 | tags=18%, list=14%, signal=20% |
| 734 | SIGNAL\_TRANSDUCTION\_BY\_PROTEIN\_PHOSPHORYLATION(GO:0023014) |  | 217 | 0.27 | 1.21 | 0.126 | 0.543 | 1.000 | 4476 | tags=18%, list=14%, signal=20% |
| 735 | PHOSPHORYLATION(GO:0016310) |  | 1320 | 0.19 | 1.21 | 0.000 | 0.542 | 1.000 | 7328 | tags=21%, list=23%, signal=26% |
| 736 | ADAPTIVE\_IMMUNE\_RESPONSE\_BASED\_ON\_SOMATIC\_RECOMBINATION\_OF\_IMMUNE\_RECEPTORS\_BUILT\_FROM\_IMMUNOGLOBULIN\_SUPERFAMILY\_DOMAINS(GO:0002460) |  | 45 | 0.33 | 1.21 | 0.177 | 0.542 | 1.000 | 5273 | tags=29%, list=17%, signal=35% |
| 737 | SMALL\_MOLECULE\_BIOSYNTHETIC\_PROCESS(GO:0044283) |  | 408 | 0.23 | 1.21 | 0.233 | 0.542 | 1.000 | 5292 | tags=18%, list=17%, signal=21% |
| 738 | ORGANOPHOSPHATE\_ESTER\_TRANSPORT(GO:0015748) |  | 53 | 0.28 | 1.21 | 0.208 | 0.543 | 1.000 | 8613 | tags=36%, list=27%, signal=49% |
| 739 | ESTABLISHMENT\_OF\_LOCALIZATION\_IN\_CELL(GO:0051649) |  | 1029 | 0.17 | 1.21 | 0.116 | 0.543 | 1.000 | 4535 | tags=10%, list=14%, signal=11% |
| 740 | NUCLEAR\_PORE(GO:0005643) |  | 42 | 0.24 | 1.21 | 0.229 | 0.543 | 1.000 | 1448 | tags=5%, list=5%, signal=5% |
| 741 | AMINO\_ACID\_TRANSMEMBRANE\_TRANSPORT(GO:0003333) |  | 39 | 0.31 | 1.21 | 0.130 | 0.545 | 1.000 | 2998 | tags=13%, list=9%, signal=14% |
| 742 | NEGATIVE\_REGULATION\_OF\_MACROMOLECULE\_METABOLIC\_PROCESS(GO:0010605) |  | 818 | 0.18 | 1.21 | 0.118 | 0.544 | 1.000 | 7127 | tags=18%, list=22%, signal=23% |
| 743 | POSITIVE\_REGULATION\_OF\_KINASE\_ACTIVITY(GO:0033674) |  | 111 | 0.31 | 1.21 | 0.229 | 0.544 | 1.000 | 7756 | tags=30%, list=24%, signal=39% |
| 744 | EXTRACELLULAR\_REGION(GO:0005576) |  | 1324 | 0.25 | 1.21 | 0.165 | 0.543 | 1.000 | 6654 | tags=23%, list=21%, signal=28% |
| 745 | LYMPHOCYTE\_ACTIVATION(GO:0046649) |  | 133 | 0.26 | 1.21 | 0.106 | 0.543 | 1.000 | 2107 | tags=11%, list=7%, signal=12% |
| 746 | REGULATION\_OF\_GENE\_EXPRESSION\_\_EPIGENETIC(GO:0040029) |  | 90 | 0.28 | 1.21 | 0.221 | 0.544 | 1.000 | 6084 | tags=19%, list=19%, signal=23% |
| 747 | RNA\_BINDING(GO:0003723) |  | 892 | 0.16 | 1.20 | 0.100 | 0.546 | 1.000 | 5473 | tags=10%, list=17%, signal=12% |
| 748 | CELLULAR\_AMIDE\_METABOLIC\_PROCESS(GO:0043603) |  | 634 | 0.20 | 1.20 | 0.165 | 0.545 | 1.000 | 3184 | tags=7%, list=10%, signal=8% |
| 749 | REGULATION\_OF\_NEURON\_APOPTOTIC\_PROCESS(GO:0043523) |  | 35 | 0.29 | 1.20 | 0.145 | 0.547 | 1.000 | 2398 | tags=11%, list=8%, signal=12% |
| 750 | IMMUNE\_SYSTEM\_PROCESS(GO:0002376) |  | 1074 | 0.21 | 1.20 | 0.000 | 0.549 | 1.000 | 5168 | tags=17%, list=16%, signal=19% |
| 751 | REGULATION\_OF\_POSTSYNAPTIC\_MEMBRANE\_POTENTIAL(GO:0060078) |  | 19 | 0.52 | 1.20 | 0.351 | 0.548 | 1.000 | 9274 | tags=37%, list=29%, signal=52% |
| 752 | SOLUTE:SODIUM\_SYMPORTER\_ACTIVITY(GO:0015370) |  | 53 | 0.37 | 1.20 | 0.217 | 0.548 | 1.000 | 6310 | tags=25%, list=20%, signal=31% |
| 753 | NUCLEAR\_CHROMOSOME\_SEGREGATION(GO:0098813) |  | 126 | 0.26 | 1.20 | 0.195 | 0.547 | 1.000 | 8939 | tags=27%, list=28%, signal=37% |
| 754 | UBIQUITIN\_LIKE\_PROTEIN\_TRANSFERASE\_ACTIVITY(GO:0019787) |  | 384 | 0.18 | 1.20 | 0.035 | 0.548 | 1.000 | 4951 | tags=13%, list=16%, signal=15% |
| 755 | ESTABLISHMENT\_OF\_ORGANELLE\_LOCALIZATION(GO:0051656) |  | 237 | 0.21 | 1.20 | 0.198 | 0.547 | 1.000 | 4714 | tags=11%, list=15%, signal=13% |
| 756 | OOGENESIS(GO:0048477) |  | 52 | 0.34 | 1.20 | 0.115 | 0.547 | 1.000 | 9734 | tags=35%, list=31%, signal=50% |
| 757 | NON\_MEMBRANE\_SPANNING\_PROTEIN\_TYROSINE\_KINASE\_ACTIVITY(GO:0004715) |  | 44 | 0.34 | 1.20 | 0.262 | 0.547 | 1.000 | 8638 | tags=39%, list=27%, signal=53% |
| 758 | HISTONE\_BINDING(GO:0042393) |  | 126 | 0.24 | 1.20 | 0.261 | 0.546 | 1.000 | 3427 | tags=7%, list=11%, signal=8% |
| 759 | ASPARTIC\_TYPE\_ENDOPEPTIDASE\_ACTIVITY(GO:0004190) |  | 15 | 0.47 | 1.20 | 0.277 | 0.546 | 1.000 | 11594 | tags=47%, list=36%, signal=73% |
| 760 | GLYCOSYL\_COMPOUND\_CATABOLIC\_PROCESS(GO:1901658) |  | 18 | 0.46 | 1.20 | 0.233 | 0.550 | 1.000 | 1678 | tags=17%, list=5%, signal=18% |
| 761 | CELLULAR\_RESPONSE\_TO\_OXIDATIVE\_STRESS(GO:0034599) |  | 42 | 0.26 | 1.20 | 0.257 | 0.550 | 1.000 | 4069 | tags=17%, list=13%, signal=19% |
| 762 | RESPONSE\_TO\_ORGANIC\_SUBSTANCE(GO:0010033) |  | 897 | 0.20 | 1.20 | 0.037 | 0.550 | 1.000 | 5194 | tags=15%, list=16%, signal=18% |
| 763 | SENSORY\_SYSTEM\_DEVELOPMENT(GO:0048880) |  | 125 | 0.22 | 1.20 | 0.061 | 0.549 | 1.000 | 8170 | tags=26%, list=26%, signal=35% |
| 764 | LATERAL\_LINE\_SYSTEM\_DEVELOPMENT(GO:0048925) |  | 125 | 0.22 | 1.20 | 0.061 | 0.548 | 1.000 | 8170 | tags=26%, list=26%, signal=35% |
| 765 | POSITIVE\_REGULATION\_OF\_TRANSFERASE\_ACTIVITY(GO:0051347) |  | 128 | 0.29 | 1.20 | 0.159 | 0.548 | 1.000 | 7756 | tags=29%, list=24%, signal=38% |
| 766 | GERM\_PLASM(GO:0060293) |  | 24 | 0.42 | 1.20 | 0.226 | 0.550 | 1.000 | 6084 | tags=29%, list=19%, signal=36% |
| 767 | NADP\_BINDING(GO:0050661) |  | 32 | 0.30 | 1.20 | 0.185 | 0.551 | 1.000 | 6079 | tags=25%, list=19%, signal=31% |
| 768 | SYNTAXIN\_BINDING(GO:0019905) |  | 55 | 0.31 | 1.20 | 0.219 | 0.551 | 1.000 | 2932 | tags=9%, list=9%, signal=10% |
| 769 | MICROTUBULE\_ORGANIZING\_CENTER\_PART(GO:0044450) |  | 69 | 0.29 | 1.19 | 0.298 | 0.553 | 1.000 | 2268 | tags=10%, list=7%, signal=11% |
| 770 | MYELOID\_CELL\_DIFFERENTIATION(GO:0030099) |  | 203 | 0.21 | 1.19 | 0.117 | 0.554 | 1.000 | 6937 | tags=20%, list=22%, signal=26% |
| 771 | LIPID\_CATABOLIC\_PROCESS(GO:0016042) |  | 177 | 0.27 | 1.19 | 0.277 | 0.554 | 1.000 | 5110 | tags=18%, list=16%, signal=21% |
| 772 | RESPONSE\_TO\_BIOTIC\_STIMULUS(GO:0009607) |  | 255 | 0.26 | 1.19 | 0.053 | 0.555 | 1.000 | 6543 | tags=27%, list=21%, signal=33% |
| 773 | RESPONSE\_TO\_OTHER\_ORGANISM(GO:0051707) |  | 251 | 0.26 | 1.19 | 0.024 | 0.554 | 1.000 | 7314 | tags=30%, list=23%, signal=38% |
| 774 | RESPONSE\_TO\_EXTERNAL\_BIOTIC\_STIMULUS(GO:0043207) |  | 251 | 0.26 | 1.19 | 0.024 | 0.554 | 1.000 | 7314 | tags=30%, list=23%, signal=38% |
| 775 | EPHRIN\_RECEPTOR\_ACTIVITY(GO:0005003) |  | 15 | 0.53 | 1.19 | 0.294 | 0.555 | 1.000 | 3307 | tags=20%, list=10%, signal=22% |
| 776 | PHOSPHORUS\_METABOLIC\_PROCESS(GO:0006793) |  | 1956 | 0.18 | 1.19 | 0.028 | 0.554 | 1.000 | 7328 | tags=20%, list=23%, signal=25% |
| 777 | REGULATION\_OF\_STRESS\_ACTIVATED\_MAPK\_CASCADE(GO:0032872) |  | 37 | 0.34 | 1.19 | 0.256 | 0.555 | 1.000 | 4176 | tags=24%, list=13%, signal=28% |
| 778 | REGULATION\_OF\_STRESS\_ACTIVATED\_PROTEIN\_KINASE\_SIGNALING\_CASCADE(GO:0070302) |  | 37 | 0.34 | 1.19 | 0.256 | 0.554 | 1.000 | 4176 | tags=24%, list=13%, signal=28% |
| 779 | INTERSPECIES\_INTERACTION\_BETWEEN\_ORGANISMS(GO:0044419) |  | 24 | 0.36 | 1.19 | 0.136 | 0.554 | 1.000 | 5116 | tags=21%, list=16%, signal=25% |
| 780 | PHENOL\_CONTAINING\_COMPOUND\_METABOLIC\_PROCESS(GO:0018958) |  | 33 | 0.39 | 1.19 | 0.369 | 0.555 | 1.000 | 690 | tags=9%, list=2%, signal=9% |
| 781 | PYRUVATE\_METABOLIC\_PROCESS(GO:0006090) |  | 53 | 0.33 | 1.19 | 0.266 | 0.555 | 1.000 | 4480 | tags=19%, list=14%, signal=22% |
| 782 | HETEROCYCLE\_CATABOLIC\_PROCESS(GO:0046700) |  | 289 | 0.20 | 1.19 | 0.113 | 0.555 | 1.000 | 6380 | tags=17%, list=20%, signal=21% |
| 783 | ROUGH\_ENDOPLASMIC\_RETICULUM(GO:0005791) |  | 22 | 0.30 | 1.19 | 0.220 | 0.555 | 1.000 | 8031 | tags=23%, list=25%, signal=30% |
| 784 | PROTEIN\_PHOSPHORYLATION(GO:0006468) |  | 995 | 0.21 | 1.19 | 0.068 | 0.555 | 1.000 | 7328 | tags=22%, list=23%, signal=27% |
| 785 | REGULATION\_OF\_MICROTUBULE\_CYTOSKELETON\_ORGANIZATION(GO:0070507) |  | 88 | 0.23 | 1.19 | 0.122 | 0.555 | 1.000 | 3632 | tags=8%, list=11%, signal=9% |
| 786 | POSITIVE\_CHEMOTAXIS(GO:0050918) |  | 16 | 0.43 | 1.19 | 0.317 | 0.555 | 1.000 | 9357 | tags=56%, list=29%, signal=80% |
| 787 | EXECUTION\_PHASE\_OF\_APOPTOSIS(GO:0097194) |  | 23 | 0.35 | 1.19 | 0.193 | 0.555 | 1.000 | 5211 | tags=26%, list=16%, signal=31% |
| 788 | NEGATIVE\_REGULATION\_OF\_MITOTIC\_CELL\_CYCLE(GO:0045930) |  | 85 | 0.25 | 1.19 | 0.251 | 0.557 | 1.000 | 1650 | tags=7%, list=5%, signal=7% |
| 789 | DEFENSE\_RESPONSE\_TO\_BACTERIUM(GO:0042742) |  | 84 | 0.27 | 1.19 | 0.026 | 0.558 | 1.000 | 3534 | tags=15%, list=11%, signal=17% |
| 790 | PROTEIN\_LOCALIZATION\_TO\_CELL\_PERIPHERY(GO:1990778) |  | 104 | 0.23 | 1.19 | 0.151 | 0.557 | 1.000 | 5859 | tags=21%, list=18%, signal=26% |
| 791 | RIBONUCLEASE\_ACTIVITY(GO:0004540) |  | 77 | 0.29 | 1.19 | 0.240 | 0.558 | 1.000 | 3639 | tags=13%, list=11%, signal=15% |
| 792 | MONOCARBOXYLIC\_ACID\_CATABOLIC\_PROCESS(GO:0072329) |  | 63 | 0.28 | 1.19 | 0.363 | 0.559 | 1.000 | 4565 | tags=17%, list=14%, signal=20% |
| 793 | PROTEOGLYCAN\_BIOSYNTHETIC\_PROCESS(GO:0030166) |  | 49 | 0.30 | 1.19 | 0.254 | 0.559 | 1.000 | 7006 | tags=24%, list=22%, signal=31% |
| 794 | ORGANIC\_HYDROXY\_COMPOUND\_BIOSYNTHETIC\_PROCESS(GO:1901617) |  | 99 | 0.27 | 1.19 | 0.143 | 0.559 | 1.000 | 3158 | tags=12%, list=10%, signal=13% |
| 795 | CELLULAR\_PROTEIN\_METABOLIC\_PROCESS(GO:0044267) |  | 3154 | 0.16 | 1.18 | 0.068 | 0.559 | 1.000 | 5069 | tags=12%, list=16%, signal=12% |
| 796 | CELLULAR\_AMINO\_ACID\_METABOLIC\_PROCESS(GO:0006520) |  | 226 | 0.22 | 1.18 | 0.185 | 0.559 | 1.000 | 5080 | tags=17%, list=16%, signal=20% |
| 797 | RESPONSE\_TO\_XENOBIOTIC\_STIMULUS(GO:0009410) |  | 103 | 0.30 | 1.18 | 0.282 | 0.559 | 1.000 | 6399 | tags=29%, list=20%, signal=36% |
| 798 | CYSTEINE\_TYPE\_PEPTIDASE\_ACTIVITY(GO:0008234) |  | 177 | 0.21 | 1.18 | 0.206 | 0.558 | 1.000 | 5095 | tags=15%, list=16%, signal=17% |
| 799 | P\_GRANULE(GO:0043186) |  | 23 | 0.42 | 1.18 | 0.226 | 0.558 | 1.000 | 6084 | tags=30%, list=19%, signal=38% |
| 800 | TRANSFERASE\_ACTIVITY\_\_TRANSFERRING\_ACYL\_GROUPS\_OTHER\_THAN\_AMINO\_ACYL\_GROUPS(GO:0016747) |  | 202 | 0.23 | 1.18 | 0.222 | 0.561 | 1.000 | 4261 | tags=14%, list=13%, signal=16% |
| 801 | EMBRYONIC\_HEART\_TUBE\_DEVELOPMENT(GO:0035050) |  | 166 | 0.23 | 1.18 | 0.153 | 0.562 | 1.000 | 6941 | tags=20%, list=22%, signal=26% |
| 802 | FATTY\_ACID\_BIOSYNTHETIC\_PROCESS(GO:0006633) |  | 82 | 0.29 | 1.18 | 0.197 | 0.561 | 1.000 | 5292 | tags=23%, list=17%, signal=28% |
| 803 | REGULATION\_OF\_NEURON\_DEATH(GO:1901214) |  | 43 | 0.28 | 1.18 | 0.265 | 0.561 | 1.000 | 2668 | tags=12%, list=8%, signal=13% |
| 804 | REGULATION\_OF\_CELL\_DIVISION(GO:0051302) |  | 55 | 0.27 | 1.18 | 0.295 | 0.562 | 1.000 | 7185 | tags=27%, list=23%, signal=35% |
| 805 | EXTRINSIC\_COMPONENT\_OF\_MEMBRANE(GO:0019898) |  | 180 | 0.24 | 1.18 | 0.150 | 0.563 | 1.000 | 8475 | tags=27%, list=27%, signal=36% |
| 806 | DRUG\_METABOLIC\_PROCESS(GO:0017144) |  | 358 | 0.20 | 1.18 | 0.073 | 0.567 | 1.000 | 4489 | tags=13%, list=14%, signal=14% |
| 807 | CELLULAR\_BIOGENIC\_AMINE\_METABOLIC\_PROCESS(GO:0006576) |  | 32 | 0.34 | 1.18 | 0.289 | 0.567 | 1.000 | 2458 | tags=16%, list=8%, signal=17% |
| 808 | DEPHOSPHORYLATION(GO:0016311) |  | 247 | 0.19 | 1.18 | 0.133 | 0.567 | 1.000 | 5099 | tags=14%, list=16%, signal=17% |
| 809 | BASAL\_TRANSCRIPTION\_MACHINERY\_BINDING(GO:0001098) |  | 25 | 0.24 | 1.18 | 0.211 | 0.567 | 1.000 | 7061 | tags=24%, list=22%, signal=31% |
| 810 | BASAL\_RNA\_POLYMERASE\_II\_TRANSCRIPTION\_MACHINERY\_BINDING(GO:0001099) |  | 25 | 0.24 | 1.18 | 0.211 | 0.566 | 1.000 | 7061 | tags=24%, list=22%, signal=31% |
| 811 | TRIGLYCERIDE\_BIOSYNTHETIC\_PROCESS(GO:0019432) |  | 17 | 0.34 | 1.18 | 0.201 | 0.567 | 1.000 | 7304 | tags=29%, list=23%, signal=38% |
| 812 | CHROMOSOME\_SEGREGATION(GO:0007059) |  | 169 | 0.25 | 1.18 | 0.238 | 0.566 | 1.000 | 8939 | tags=25%, list=28%, signal=34% |
| 813 | MAINTENANCE\_OF\_CELL\_NUMBER(GO:0098727) |  | 22 | 0.37 | 1.18 | 0.211 | 0.566 | 1.000 | 4227 | tags=14%, list=13%, signal=16% |
| 814 | REGULATION\_OF\_LEUKOCYTE\_ACTIVATION(GO:0002694) |  | 82 | 0.30 | 1.18 | 0.179 | 0.565 | 1.000 | 2083 | tags=13%, list=7%, signal=14% |
| 815 | REGULATION\_OF\_CELL\_ACTIVATION(GO:0050865) |  | 84 | 0.30 | 1.18 | 0.238 | 0.565 | 1.000 | 2083 | tags=13%, list=7%, signal=14% |
| 816 | LIPID\_TRANSPORTER\_ACTIVITY(GO:0005319) |  | 92 | 0.27 | 1.18 | 0.256 | 0.565 | 1.000 | 4379 | tags=17%, list=14%, signal=20% |
| 817 | DNA\_METABOLIC\_PROCESS(GO:0006259) |  | 497 | 0.21 | 1.18 | 0.171 | 0.564 | 1.000 | 3924 | tags=12%, list=12%, signal=13% |
| 818 | RUFFLE\_ORGANIZATION(GO:0031529) |  | 15 | 0.39 | 1.18 | 0.201 | 0.564 | 1.000 | 10479 | tags=53%, list=33%, signal=79% |
| 819 | COPII\_COATED\_ER\_TO\_GOLGI\_TRANSPORT\_VESICLE(GO:0030134) |  | 48 | 0.24 | 1.18 | 0.272 | 0.564 | 1.000 | 2145 | tags=8%, list=7%, signal=9% |
| 820 | ENDOTHELIAL\_CELL\_PROLIFERATION(GO:0001935) |  | 35 | 0.29 | 1.18 | 0.214 | 0.565 | 1.000 | 6618 | tags=20%, list=21%, signal=25% |
| 821 | VESICLE\_MEDIATED\_TRANSPORT\_TO\_THE\_PLASMA\_MEMBRANE(GO:0098876) |  | 34 | 0.29 | 1.18 | 0.200 | 0.564 | 1.000 | 3734 | tags=18%, list=12%, signal=20% |
| 822 | CYTOPLASMIC\_DYNEIN\_COMPLEX(GO:0005868) |  | 16 | 0.46 | 1.18 | 0.270 | 0.564 | 1.000 | 8 | tags=6%, list=0%, signal=6% |
| 823 | RESPONSE\_TO\_YEAST(GO:0001878) |  | 24 | 0.35 | 1.17 | 0.341 | 0.564 | 1.000 | 8878 | tags=42%, list=28%, signal=58% |
| 824 | MEMBRANE\_PROTEIN\_COMPLEX(GO:0098796) |  | 879 | 0.21 | 1.17 | 0.176 | 0.564 | 1.000 | 4444 | tags=11%, list=14%, signal=13% |
| 825 | REPRODUCTIVE\_PROCESS(GO:0022414) |  | 272 | 0.24 | 1.17 | 0.030 | 0.563 | 1.000 | 5026 | tags=16%, list=16%, signal=19% |
| 826 | CAMERA\_TYPE\_EYE\_PHOTORECEPTOR\_CELL\_DIFFERENTIATION(GO:0060219) |  | 25 | 0.40 | 1.17 | 0.242 | 0.563 | 1.000 | 4111 | tags=16%, list=13%, signal=18% |
| 827 | EPIBOLY(GO:0090504) |  | 71 | 0.26 | 1.17 | 0.213 | 0.564 | 1.000 | 3364 | tags=10%, list=11%, signal=11% |
| 828 | GROWTH\_FACTOR\_ACTIVITY(GO:0008083) |  | 120 | 0.28 | 1.17 | 0.187 | 0.565 | 1.000 | 10271 | tags=37%, list=32%, signal=54% |
| 829 | FILAMENTOUS\_ACTIN(GO:0031941) |  | 20 | 0.31 | 1.17 | 0.217 | 0.565 | 1.000 | 249 | tags=5%, list=1%, signal=5% |
| 830 | ORGANELLAR\_RIBOSOME(GO:0000313) |  | 58 | 0.27 | 1.17 | 0.308 | 0.565 | 1.000 | 2521 | tags=7%, list=8%, signal=7% |
| 831 | MITOCHONDRIAL\_RIBOSOME(GO:0005761) |  | 58 | 0.27 | 1.17 | 0.308 | 0.564 | 1.000 | 2521 | tags=7%, list=8%, signal=7% |
| 832 | PROTEIN\_LOCALIZATION\_TO\_CHROMOSOME(GO:0034502) |  | 22 | 0.34 | 1.17 | 0.278 | 0.564 | 1.000 | 1006 | tags=9%, list=3%, signal=9% |
| 833 | COFACTOR\_METABOLIC\_PROCESS(GO:0051186) |  | 330 | 0.23 | 1.17 | 0.174 | 0.563 | 1.000 | 5876 | tags=20%, list=18%, signal=25% |
| 834 | ANION\_BINDING(GO:0043168) |  | 2521 | 0.17 | 1.17 | 0.114 | 0.563 | 1.000 | 7468 | tags=21%, list=23%, signal=25% |
| 835 | POSITIVE\_REGULATION\_OF\_CELLULAR\_METABOLIC\_PROCESS(GO:0031325) |  | 990 | 0.20 | 1.17 | 0.132 | 0.564 | 1.000 | 7125 | tags=20%, list=22%, signal=25% |
| 836 | PHOSPHATE\_CONTAINING\_COMPOUND\_METABOLIC\_PROCESS(GO:0006796) |  | 1891 | 0.18 | 1.17 | 0.028 | 0.564 | 1.000 | 7328 | tags=20%, list=23%, signal=24% |
| 837 | RESPONSE\_TO\_HORMONE(GO:0009725) |  | 222 | 0.25 | 1.17 | 0.157 | 0.563 | 1.000 | 7182 | tags=32%, list=23%, signal=41% |
| 838 | REGULATION\_OF\_MEMBRANE\_LIPID\_DISTRIBUTION(GO:0097035) |  | 43 | 0.30 | 1.17 | 0.226 | 0.563 | 1.000 | 5211 | tags=21%, list=16%, signal=25% |
| 839 | RHOMBOMERE\_MORPHOGENESIS(GO:0021593) |  | 18 | 0.41 | 1.17 | 0.407 | 0.563 | 1.000 | 7745 | tags=33%, list=24%, signal=44% |
| 840 | REGULATION\_OF\_LEUKOCYTE\_MIGRATION(GO:0002685) |  | 50 | 0.26 | 1.17 | 0.191 | 0.564 | 1.000 | 2083 | tags=8%, list=7%, signal=9% |
| 841 | NEGATIVE\_REGULATION\_OF\_CELLULAR\_PROCESS(GO:0048523) |  | 1634 | 0.19 | 1.17 | 0.105 | 0.566 | 1.000 | 4516 | tags=12%, list=14%, signal=13% |
| 842 | PEPTIDYL\_CYSTEINE\_MODIFICATION(GO:0018198) |  | 30 | 0.32 | 1.17 | 0.181 | 0.566 | 1.000 | 7167 | tags=33%, list=22%, signal=43% |
| 843 | PROTEIN\_MONOUBIQUITINATION(GO:0006513) |  | 29 | 0.33 | 1.17 | 0.272 | 0.566 | 1.000 | 365 | tags=3%, list=1%, signal=3% |
| 844 | POLE\_PLASM(GO:0045495) |  | 25 | 0.41 | 1.17 | 0.265 | 0.566 | 1.000 | 6084 | tags=28%, list=19%, signal=35% |
| 845 | REPRODUCTION(GO:0000003) |  | 276 | 0.24 | 1.17 | 0.051 | 0.565 | 1.000 | 5268 | tags=16%, list=17%, signal=19% |
| 846 | REACTIVE\_OXYGEN\_SPECIES\_METABOLIC\_PROCESS(GO:0072593) |  | 57 | 0.28 | 1.17 | 0.208 | 0.565 | 1.000 | 5171 | tags=19%, list=16%, signal=23% |
| 847 | CELLULAR\_CATABOLIC\_PROCESS(GO:0044248) |  | 1254 | 0.17 | 1.17 | 0.123 | 0.565 | 1.000 | 5110 | tags=13%, list=16%, signal=14% |
| 848 | REGULATION\_OF\_LYMPHOCYTE\_ACTIVATION(GO:0051249) |  | 73 | 0.31 | 1.17 | 0.280 | 0.566 | 1.000 | 2083 | tags=15%, list=7%, signal=16% |
| 849 | CHROMOSOME\_ORGANIZATION(GO:0051276) |  | 608 | 0.18 | 1.17 | 0.346 | 0.567 | 1.000 | 4778 | tags=10%, list=15%, signal=11% |
| 850 | MITOTIC\_DNA\_DAMAGE\_CHECKPOINT(GO:0044773) |  | 24 | 0.34 | 1.17 | 0.210 | 0.567 | 1.000 | 1006 | tags=8%, list=3%, signal=9% |
| 851 | ZINC\_ION\_BINDING(GO:0008270) |  | 708 | 0.19 | 1.17 | 0.117 | 0.566 | 1.000 | 6729 | tags=18%, list=21%, signal=22% |
| 852 | NUCLEOCYTOPLASMIC\_CARRIER\_ACTIVITY(GO:0140142) |  | 21 | 0.31 | 1.17 | 0.272 | 0.566 | 1.000 | 2896 | tags=14%, list=9%, signal=16% |
| 853 | COFACTOR\_BIOSYNTHETIC\_PROCESS(GO:0051188) |  | 166 | 0.25 | 1.17 | 0.188 | 0.566 | 1.000 | 5108 | tags=18%, list=16%, signal=21% |
| 854 | NEGATIVE\_REGULATION\_OF\_NOTCH\_SIGNALING\_PATHWAY(GO:0045746) |  | 24 | 0.33 | 1.16 | 0.252 | 0.567 | 1.000 | 7242 | tags=25%, list=23%, signal=32% |
| 855 | NEGATIVE\_REGULATION\_OF\_T\_CELL\_ACTIVATION(GO:0050868) |  | 29 | 0.34 | 1.16 | 0.228 | 0.568 | 1.000 | 3387 | tags=24%, list=11%, signal=27% |
| 856 | NEGATIVE\_REGULATION\_OF\_LEUKOCYTE\_CELL\_CELL\_ADHESION(GO:1903038) |  | 29 | 0.34 | 1.16 | 0.228 | 0.568 | 1.000 | 3387 | tags=24%, list=11%, signal=27% |
| 857 | IMMUNE\_SYSTEM\_DEVELOPMENT(GO:0002520) |  | 519 | 0.18 | 1.16 | 0.238 | 0.567 | 1.000 | 6937 | tags=18%, list=22%, signal=23% |
| 858 | CHROMATIN\_ORGANIZATION(GO:0006325) |  | 418 | 0.19 | 1.16 | 0.269 | 0.567 | 1.000 | 4750 | tags=10%, list=15%, signal=12% |
| 859 | PROTEIN\_LOCALIZATION\_TO\_CYTOSKELETON(GO:0044380) |  | 16 | 0.35 | 1.16 | 0.256 | 0.566 | 1.000 | 1259 | tags=13%, list=4%, signal=13% |
| 860 | CARBOXY\_LYASE\_ACTIVITY(GO:0016831) |  | 30 | 0.39 | 1.16 | 0.276 | 0.566 | 1.000 | 10096 | tags=47%, list=32%, signal=68% |
| 861 | NOTCH\_BINDING(GO:0005112) |  | 19 | 0.38 | 1.16 | 0.349 | 0.568 | 1.000 | 6138 | tags=26%, list=19%, signal=33% |
| 862 | ENDOPLASMIC\_RETICULUM\_EXIT\_SITE(GO:0070971) |  | 16 | 0.37 | 1.16 | 0.225 | 0.570 | 1.000 | 4817 | tags=25%, list=15%, signal=29% |
| 863 | ORGANELLE\_SUBCOMPARTMENT(GO:0031984) |  | 903 | 0.19 | 1.16 | 0.242 | 0.569 | 1.000 | 6673 | tags=18%, list=21%, signal=23% |
| 864 | CAMP\_METABOLIC\_PROCESS(GO:0046058) |  | 31 | 0.35 | 1.16 | 0.261 | 0.570 | 1.000 | 6847 | tags=29%, list=21%, signal=37% |
| 865 | RHYTHMIC\_PROCESS(GO:0048511) |  | 115 | 0.26 | 1.16 | 0.232 | 0.569 | 1.000 | 7043 | tags=24%, list=22%, signal=31% |
| 866 | DETECTION\_OF\_VISIBLE\_LIGHT(GO:0009584) |  | 16 | 0.50 | 1.16 | 0.270 | 0.569 | 1.000 | 7043 | tags=25%, list=22%, signal=32% |
| 867 | POSITIVE\_REGULATION\_OF\_INTRACELLULAR\_SIGNAL\_TRANSDUCTION(GO:1902533) |  | 241 | 0.24 | 1.16 | 0.067 | 0.571 | 1.000 | 4398 | tags=17%, list=14%, signal=19% |
| 868 | POSITIVE\_REGULATION\_OF\_METABOLIC\_PROCESS(GO:0009893) |  | 1057 | 0.20 | 1.16 | 0.114 | 0.571 | 1.000 | 7125 | tags=20%, list=22%, signal=25% |
| 869 | CELL\_CELL\_JUNCTION\_ORGANIZATION(GO:0045216) |  | 101 | 0.25 | 1.16 | 0.334 | 0.570 | 1.000 | 4945 | tags=15%, list=16%, signal=18% |
| 870 | CCR\_CHEMOKINE\_RECEPTOR\_BINDING(GO:0048020) |  | 22 | 0.44 | 1.16 | 0.197 | 0.570 | 1.000 | 2466 | tags=27%, list=8%, signal=30% |
| 871 | BIOMINERAL\_TISSUE\_DEVELOPMENT(GO:0031214) |  | 47 | 0.26 | 1.16 | 0.232 | 0.571 | 1.000 | 6654 | tags=23%, list=21%, signal=30% |
| 872 | SOLUTE:CATION\_SYMPORTER\_ACTIVITY(GO:0015294) |  | 73 | 0.31 | 1.16 | 0.252 | 0.570 | 1.000 | 6310 | tags=21%, list=20%, signal=26% |
| 873 | VENOUS\_BLOOD\_VESSEL\_DEVELOPMENT(GO:0060841) |  | 15 | 0.37 | 1.16 | 0.232 | 0.571 | 1.000 | 7924 | tags=33%, list=25%, signal=44% |
| 874 | ASPARTIC\_TYPE\_PEPTIDASE\_ACTIVITY(GO:0070001) |  | 16 | 0.42 | 1.16 | 0.248 | 0.571 | 1.000 | 11594 | tags=44%, list=36%, signal=69% |
| 875 | DNA\_DEPENDENT\_DNA\_REPLICATION\_MAINTENANCE\_OF\_FIDELITY(GO:0045005) |  | 20 | 0.38 | 1.16 | 0.277 | 0.570 | 1.000 | 2730 | tags=20%, list=9%, signal=22% |
| 876 | REGULATION\_OF\_T\_CELL\_ACTIVATION(GO:0050863) |  | 61 | 0.32 | 1.16 | 0.306 | 0.570 | 1.000 | 3523 | tags=21%, list=11%, signal=24% |
| 877 | IMPORT\_INTO\_CELL(GO:0098657) |  | 377 | 0.22 | 1.16 | 0.252 | 0.571 | 1.000 | 5310 | tags=14%, list=17%, signal=16% |
| 878 | PLASMA\_MEMBRANE\_ORGANIZATION(GO:0007009) |  | 65 | 0.24 | 1.16 | 0.287 | 0.570 | 1.000 | 8074 | tags=22%, list=25%, signal=29% |
| 879 | VIRAL\_PROCESS(GO:0016032) |  | 15 | 0.38 | 1.16 | 0.233 | 0.570 | 1.000 | 2871 | tags=13%, list=9%, signal=15% |
| 880 | APICAL\_JUNCTION\_COMPLEX(GO:0043296) |  | 91 | 0.24 | 1.16 | 0.322 | 0.570 | 1.000 | 6249 | tags=19%, list=20%, signal=23% |
| 881 | PYRIMIDINE\_CONTAINING\_COMPOUND\_CATABOLIC\_PROCESS(GO:0072529) |  | 21 | 0.38 | 1.16 | 0.199 | 0.570 | 1.000 | 5638 | tags=24%, list=18%, signal=29% |
| 882 | NEGATIVE\_REGULATION\_OF\_DEPHOSPHORYLATION(GO:0035305) |  | 18 | 0.36 | 1.16 | 0.277 | 0.572 | 1.000 | 5099 | tags=22%, list=16%, signal=26% |
| 883 | PROTEIN\_LOCALIZATION\_TO\_CILIUM(GO:0061512) |  | 26 | 0.34 | 1.15 | 0.332 | 0.574 | 1.000 | 3603 | tags=15%, list=11%, signal=17% |
| 884 | MOLECULAR\_CARRIER\_ACTIVITY(GO:0140104) |  | 47 | 0.31 | 1.15 | 0.297 | 0.574 | 1.000 | 3948 | tags=17%, list=12%, signal=19% |
| 885 | PALMITOYLTRANSFERASE\_ACTIVITY(GO:0016409) |  | 60 | 0.27 | 1.15 | 0.274 | 0.574 | 1.000 | 8160 | tags=28%, list=26%, signal=38% |
| 886 | LIGASE\_ACTIVITY(GO:0016874) |  | 137 | 0.21 | 1.15 | 0.193 | 0.577 | 1.000 | 6356 | tags=18%, list=20%, signal=23% |
| 887 | GLYCOSAMINOGLYCAN\_METABOLIC\_PROCESS(GO:0030203) |  | 55 | 0.30 | 1.15 | 0.230 | 0.576 | 1.000 | 7006 | tags=31%, list=22%, signal=40% |
| 888 | MEIOTIC\_CHROMOSOME\_SEGREGATION(GO:0045132) |  | 39 | 0.30 | 1.15 | 0.278 | 0.576 | 1.000 | 1297 | tags=8%, list=4%, signal=8% |
| 889 | GLUTAMINE\_FAMILY\_AMINO\_ACID\_METABOLIC\_PROCESS(GO:0009064) |  | 55 | 0.27 | 1.15 | 0.189 | 0.576 | 1.000 | 5012 | tags=20%, list=16%, signal=24% |
| 890 | REGULATION\_OF\_INTRACELLULAR\_SIGNAL\_TRANSDUCTION(GO:1902531) |  | 516 | 0.20 | 1.15 | 0.083 | 0.576 | 1.000 | 4516 | tags=14%, list=14%, signal=16% |
| 891 | MITOTIC\_CELL\_CYCLE(GO:0000278) |  | 438 | 0.19 | 1.15 | 0.207 | 0.576 | 1.000 | 7470 | tags=20%, list=23%, signal=26% |
| 892 | NEGATIVE\_REGULATION\_OF\_CELLULAR\_CATABOLIC\_PROCESS(GO:0031330) |  | 55 | 0.21 | 1.15 | 0.181 | 0.576 | 1.000 | 10234 | tags=33%, list=32%, signal=48% |
| 893 | GLYCOGEN\_METABOLIC\_PROCESS(GO:0005977) |  | 40 | 0.29 | 1.15 | 0.201 | 0.576 | 1.000 | 2515 | tags=15%, list=8%, signal=16% |
| 894 | CELLULAR\_GLUCAN\_METABOLIC\_PROCESS(GO:0006073) |  | 40 | 0.29 | 1.15 | 0.201 | 0.576 | 1.000 | 2515 | tags=15%, list=8%, signal=16% |
| 895 | GLUCAN\_METABOLIC\_PROCESS(GO:0044042) |  | 40 | 0.29 | 1.15 | 0.201 | 0.575 | 1.000 | 2515 | tags=15%, list=8%, signal=16% |
| 896 | HELICASE\_ACTIVITY(GO:0004386) |  | 129 | 0.20 | 1.15 | 0.138 | 0.574 | 1.000 | 3614 | tags=9%, list=11%, signal=10% |
| 897 | NEGATIVE\_REGULATION\_OF\_CELL\_DEATH(GO:0060548) |  | 196 | 0.23 | 1.15 | 0.112 | 0.574 | 1.000 | 9399 | tags=34%, list=29%, signal=47% |
| 898 | CELLULAR\_AMINO\_ACID\_BIOSYNTHETIC\_PROCESS(GO:0008652) |  | 61 | 0.26 | 1.15 | 0.252 | 0.574 | 1.000 | 4000 | tags=15%, list=13%, signal=17% |
| 899 | MULTI\_ORGANISM\_PROCESS(GO:0051704) |  | 443 | 0.23 | 1.15 | 0.158 | 0.576 | 1.000 | 6606 | tags=23%, list=21%, signal=29% |
| 900 | ANTIBIOTIC\_CATABOLIC\_PROCESS(GO:0017001) |  | 31 | 0.32 | 1.15 | 0.318 | 0.577 | 1.000 | 487 | tags=6%, list=2%, signal=7% |
| 901 | EXOCYTIC\_PROCESS(GO:0140029) |  | 50 | 0.31 | 1.15 | 0.244 | 0.577 | 1.000 | 5433 | tags=20%, list=17%, signal=24% |
| 902 | APOPTOTIC\_PROCESS\_INVOLVED\_IN\_DEVELOPMENT(GO:1902742) |  | 18 | 0.37 | 1.15 | 0.312 | 0.577 | 1.000 | 5211 | tags=28%, list=16%, signal=33% |
| 903 | ACYL\_COA\_METABOLIC\_PROCESS(GO:0006637) |  | 59 | 0.33 | 1.15 | 0.319 | 0.578 | 1.000 | 5876 | tags=34%, list=18%, signal=41% |
| 904 | THIOESTER\_METABOLIC\_PROCESS(GO:0035383) |  | 59 | 0.33 | 1.15 | 0.319 | 0.577 | 1.000 | 5876 | tags=34%, list=18%, signal=41% |
| 905 | CYTOSOLIC\_TRANSPORT(GO:0016482) |  | 56 | 0.25 | 1.15 | 0.200 | 0.576 | 1.000 | 2506 | tags=9%, list=8%, signal=10% |
| 906 | OXIDOREDUCTASE\_ACTIVITY\_\_ACTING\_ON\_THE\_CH\_OH\_GROUP\_OF\_DONORS\_\_NAD\_OR\_NADP\_AS\_ACCEPTOR(GO:0016616) |  | 112 | 0.28 | 1.15 | 0.205 | 0.578 | 1.000 | 2400 | tags=10%, list=8%, signal=11% |
| 907 | PHOSPHOLIPID\_METABOLIC\_PROCESS(GO:0006644) |  | 262 | 0.22 | 1.15 | 0.167 | 0.578 | 1.000 | 5065 | tags=15%, list=16%, signal=18% |
| 908 | PROTEIN\_TYROSINE\_KINASE\_ACTIVITY(GO:0004713) |  | 126 | 0.27 | 1.15 | 0.179 | 0.578 | 1.000 | 7642 | tags=27%, list=24%, signal=35% |
| 909 | AORTA\_DEVELOPMENT(GO:0035904) |  | 18 | 0.36 | 1.15 | 0.255 | 0.577 | 1.000 | 7695 | tags=33%, list=24%, signal=44% |
| 910 | BICELLULAR\_TIGHT\_JUNCTION(GO:0005923) |  | 81 | 0.26 | 1.15 | 0.249 | 0.577 | 1.000 | 6249 | tags=21%, list=20%, signal=26% |
| 911 | PROTEIN\_PHOSPHATASE\_INHIBITOR\_ACTIVITY(GO:0004864) |  | 31 | 0.27 | 1.15 | 0.275 | 0.577 | 1.000 | 5099 | tags=19%, list=16%, signal=23% |
| 912 | PHOSPHOLIPID\_TRANSPORT(GO:0015914) |  | 39 | 0.31 | 1.15 | 0.321 | 0.576 | 1.000 | 8613 | tags=41%, list=27%, signal=56% |
| 913 | GENERATION\_OF\_PRECURSOR\_METABOLITES\_AND\_ENERGY(GO:0006091) |  | 206 | 0.22 | 1.15 | 0.171 | 0.576 | 1.000 | 4639 | tags=14%, list=15%, signal=16% |
| 914 | COFACTOR\_CATABOLIC\_PROCESS(GO:0051187) |  | 39 | 0.31 | 1.14 | 0.311 | 0.576 | 1.000 | 5171 | tags=28%, list=16%, signal=34% |
| 915 | THIOESTER\_BIOSYNTHETIC\_PROCESS(GO:0035384) |  | 26 | 0.40 | 1.14 | 0.311 | 0.577 | 1.000 | 5835 | tags=35%, list=18%, signal=42% |
| 916 | ACYL\_COA\_BIOSYNTHETIC\_PROCESS(GO:0071616) |  | 26 | 0.40 | 1.14 | 0.311 | 0.576 | 1.000 | 5835 | tags=35%, list=18%, signal=42% |
| 917 | RHOMBOMERE\_DEVELOPMENT(GO:0021546) |  | 20 | 0.36 | 1.14 | 0.333 | 0.575 | 1.000 | 7745 | tags=30%, list=24%, signal=40% |
| 918 | POSITIVE\_REGULATION\_OF\_NITROGEN\_COMPOUND\_METABOLIC\_PROCESS(GO:0051173) |  | 921 | 0.19 | 1.14 | 0.183 | 0.575 | 1.000 | 7125 | tags=20%, list=22%, signal=25% |
| 919 | LARGE\_RIBOSOMAL\_SUBUNIT(GO:0015934) |  | 81 | 0.28 | 1.14 | 0.326 | 0.575 | 1.000 | 1842 | tags=5%, list=6%, signal=5% |
| 920 | ENERGY\_RESERVE\_METABOLIC\_PROCESS(GO:0006112) |  | 42 | 0.29 | 1.14 | 0.193 | 0.576 | 1.000 | 2515 | tags=14%, list=8%, signal=15% |
| 921 | DNA\_INTEGRITY\_CHECKPOINT(GO:0031570) |  | 41 | 0.28 | 1.14 | 0.239 | 0.581 | 1.000 | 1253 | tags=7%, list=4%, signal=8% |
| 922 | CLATHRIN\_DEPENDENT\_ENDOCYTOSIS(GO:0072583) |  | 29 | 0.30 | 1.14 | 0.305 | 0.581 | 1.000 | 5310 | tags=17%, list=17%, signal=21% |
| 923 | REGULATION\_OF\_MONONUCLEAR\_CELL\_PROLIFERATION(GO:0032944) |  | 38 | 0.35 | 1.14 | 0.305 | 0.581 | 1.000 | 3523 | tags=21%, list=11%, signal=24% |
| 924 | REGULATION\_OF\_LYMPHOCYTE\_PROLIFERATION(GO:0050670) |  | 38 | 0.35 | 1.14 | 0.305 | 0.580 | 1.000 | 3523 | tags=21%, list=11%, signal=24% |
| 925 | REGULATION\_OF\_LEUKOCYTE\_PROLIFERATION(GO:0070663) |  | 38 | 0.35 | 1.14 | 0.305 | 0.579 | 1.000 | 3523 | tags=21%, list=11%, signal=24% |
| 926 | CELLULAR\_RESPONSE\_TO\_TUMOR\_NECROSIS\_FACTOR(GO:0071356) |  | 36 | 0.36 | 1.14 | 0.309 | 0.579 | 1.000 | 2466 | tags=17%, list=8%, signal=18% |
| 927 | ANION\_TRANSMEMBRANE\_TRANSPORT(GO:0098656) |  | 146 | 0.26 | 1.14 | 0.286 | 0.579 | 1.000 | 3992 | tags=12%, list=13%, signal=14% |
| 928 | AMMONIUM\_ION\_BINDING(GO:0070405) |  | 35 | 0.30 | 1.14 | 0.201 | 0.578 | 1.000 | 5685 | tags=23%, list=18%, signal=28% |
| 929 | OCCLUDING\_JUNCTION(GO:0070160) |  | 82 | 0.25 | 1.14 | 0.240 | 0.579 | 1.000 | 6249 | tags=21%, list=20%, signal=26% |
| 930 | IMMUNOGLOBULIN\_PRODUCTION\_INVOLVED\_IN\_IMMUNOGLOBULIN\_MEDIATED\_IMMUNE\_RESPONSE(GO:0002381) |  | 18 | 0.41 | 1.14 | 0.251 | 0.579 | 1.000 | 1541 | tags=22%, list=5%, signal=23% |
| 931 | REGULATION\_OF\_INTRACELLULAR\_PH(GO:0051453) |  | 37 | 0.30 | 1.14 | 0.207 | 0.579 | 1.000 | 10367 | tags=43%, list=33%, signal=64% |
| 932 | CELLULAR\_RESPONSE\_TO\_XENOBIOTIC\_STIMULUS(GO:0071466) |  | 83 | 0.28 | 1.14 | 0.323 | 0.578 | 1.000 | 6399 | tags=29%, list=20%, signal=36% |
| 933 | PHOSPHATIDYLETHANOLAMINE\_METABOLIC\_PROCESS(GO:0046337) |  | 17 | 0.37 | 1.14 | 0.294 | 0.578 | 1.000 | 3426 | tags=24%, list=11%, signal=26% |
| 934 | NEGATIVE\_REGULATION\_OF\_NEUROGENESIS(GO:0050768) |  | 71 | 0.31 | 1.14 | 0.263 | 0.579 | 1.000 | 1084 | tags=7%, list=3%, signal=7% |
| 935 | ADENYL\_RIBONUCLEOTIDE\_BINDING(GO:0032559) |  | 1460 | 0.16 | 1.14 | 0.064 | 0.579 | 1.000 | 7467 | tags=20%, list=23%, signal=26% |
| 936 | AMIDE\_BIOSYNTHETIC\_PROCESS(GO:0043604) |  | 497 | 0.20 | 1.14 | 0.352 | 0.580 | 1.000 | 2421 | tags=5%, list=8%, signal=6% |
| 937 | AXO\_DENDRITIC\_TRANSPORT(GO:0008088) |  | 27 | 0.28 | 1.14 | 0.120 | 0.581 | 1.000 | 6854 | tags=30%, list=21%, signal=38% |
| 938 | REGULATION\_OF\_IMMUNE\_RESPONSE(GO:0050776) |  | 183 | 0.25 | 1.14 | 0.131 | 0.581 | 1.000 | 7485 | tags=34%, list=23%, signal=45% |
| 939 | SOLUTE:CATION\_ANTIPORTER\_ACTIVITY(GO:0015298) |  | 32 | 0.37 | 1.14 | 0.299 | 0.580 | 1.000 | 6987 | tags=38%, list=22%, signal=48% |
| 940 | RESPONSE\_TO\_BACTERIUM(GO:0009617) |  | 157 | 0.24 | 1.14 | 0.116 | 0.582 | 1.000 | 5340 | tags=20%, list=17%, signal=24% |
| 941 | NEGATIVE\_REGULATION\_OF\_BIOLOGICAL\_PROCESS(GO:0048519) |  | 1817 | 0.18 | 1.14 | 0.165 | 0.582 | 1.000 | 4516 | tags=12%, list=14%, signal=13% |
| 942 | POSITIVE\_REGULATION\_OF\_RHO\_PROTEIN\_SIGNAL\_TRANSDUCTION(GO:0035025) |  | 29 | 0.36 | 1.13 | 0.223 | 0.583 | 1.000 | 4374 | tags=24%, list=14%, signal=28% |
| 943 | HEME\_BIOSYNTHETIC\_PROCESS(GO:0006783) |  | 21 | 0.37 | 1.13 | 0.353 | 0.583 | 1.000 | 4639 | tags=14%, list=15%, signal=17% |
| 944 | PORPHYRIN\_CONTAINING\_COMPOUND\_BIOSYNTHETIC\_PROCESS(GO:0006779) |  | 21 | 0.37 | 1.13 | 0.353 | 0.582 | 1.000 | 4639 | tags=14%, list=15%, signal=17% |
| 945 | TETRAPYRROLE\_BIOSYNTHETIC\_PROCESS(GO:0033014) |  | 21 | 0.37 | 1.13 | 0.353 | 0.582 | 1.000 | 4639 | tags=14%, list=15%, signal=17% |
| 946 | TRANSCRIPTION\_ELONGATION\_FROM\_RNA\_POLYMERASE\_II\_PROMOTER(GO:0006368) |  | 29 | 0.25 | 1.13 | 0.308 | 0.582 | 1.000 | 3762 | tags=14%, list=12%, signal=16% |
| 947 | POSITIVE\_REGULATION\_OF\_APOPTOTIC\_PROCESS(GO:0043065) |  | 107 | 0.23 | 1.13 | 0.256 | 0.581 | 1.000 | 7605 | tags=29%, list=24%, signal=38% |
| 948 | POSITIVE\_REGULATION\_OF\_CELL\_DEATH(GO:0010942) |  | 107 | 0.23 | 1.13 | 0.256 | 0.580 | 1.000 | 7605 | tags=29%, list=24%, signal=38% |
| 949 | POSITIVE\_REGULATION\_OF\_PROGRAMMED\_CELL\_DEATH(GO:0043068) |  | 107 | 0.23 | 1.13 | 0.256 | 0.580 | 1.000 | 7605 | tags=29%, list=24%, signal=38% |
| 950 | ADENYL\_NUCLEOTIDE\_BINDING(GO:0030554) |  | 1468 | 0.16 | 1.13 | 0.064 | 0.581 | 1.000 | 7467 | tags=20%, list=23%, signal=25% |
| 951 | ACTIN\_FILAMENT\_CAPPING(GO:0051693) |  | 39 | 0.28 | 1.13 | 0.213 | 0.580 | 1.000 | 3342 | tags=15%, list=10%, signal=17% |
| 952 | MEMBRANE\_PROTEIN\_ECTODOMAIN\_PROTEOLYSIS(GO:0006509) |  | 17 | 0.35 | 1.13 | 0.273 | 0.581 | 1.000 | 6162 | tags=24%, list=19%, signal=29% |
| 953 | MITOCHONDRIAL\_PROTEIN\_COMPLEX(GO:0098798) |  | 203 | 0.25 | 1.13 | 0.293 | 0.581 | 1.000 | 3184 | tags=8%, list=10%, signal=9% |
| 954 | HUMORAL\_IMMUNE\_RESPONSE(GO:0006959) |  | 60 | 0.30 | 1.13 | 0.288 | 0.581 | 1.000 | 6439 | tags=35%, list=20%, signal=44% |
| 955 | DEFENSE\_RESPONSE(GO:0006952) |  | 412 | 0.21 | 1.13 | 0.021 | 0.581 | 1.000 | 7485 | tags=28%, list=23%, signal=36% |
| 956 | REGULATION\_OF\_RESPONSE\_TO\_EXTERNAL\_STIMULUS(GO:0032101) |  | 163 | 0.22 | 1.13 | 0.200 | 0.582 | 1.000 | 2608 | tags=9%, list=8%, signal=10% |
| 957 | CIRCADIAN\_RHYTHM(GO:0007623) |  | 82 | 0.27 | 1.13 | 0.314 | 0.582 | 1.000 | 9634 | tags=37%, list=30%, signal=52% |
| 958 | PROTEIN\_LOCALIZATION\_TO\_PLASMA\_MEMBRANE(GO:0072659) |  | 90 | 0.24 | 1.13 | 0.239 | 0.583 | 1.000 | 5859 | tags=23%, list=18%, signal=29% |
| 959 | KUPFFER'S\_VESICLE\_DEVELOPMENT(GO:0070121) |  | 66 | 0.23 | 1.13 | 0.231 | 0.585 | 1.000 | 4479 | tags=14%, list=14%, signal=16% |
| 960 | ATP\_BINDING(GO:0005524) |  | 1442 | 0.16 | 1.13 | 0.028 | 0.585 | 1.000 | 7467 | tags=21%, list=23%, signal=26% |
| 961 | HEMATOPOIETIC\_OR\_LYMPHOID\_ORGAN\_DEVELOPMENT(GO:0048534) |  | 508 | 0.17 | 1.13 | 0.212 | 0.584 | 1.000 | 6937 | tags=18%, list=22%, signal=23% |
| 962 | HEMOPOIESIS(GO:0030097) |  | 495 | 0.17 | 1.13 | 0.229 | 0.584 | 1.000 | 6937 | tags=18%, list=22%, signal=23% |
| 963 | TRANSFERASE\_COMPLEX\_\_TRANSFERRING\_PHOSPHORUS\_CONTAINING\_GROUPS(GO:0061695) |  | 210 | 0.18 | 1.13 | 0.207 | 0.586 | 1.000 | 4932 | tags=12%, list=15%, signal=15% |
| 964 | MONONUCLEAR\_CELL\_PROLIFERATION(GO:0032943) |  | 40 | 0.34 | 1.13 | 0.224 | 0.588 | 1.000 | 3523 | tags=20%, list=11%, signal=22% |
| 965 | LYMPHOCYTE\_PROLIFERATION(GO:0046651) |  | 40 | 0.34 | 1.13 | 0.224 | 0.587 | 1.000 | 3523 | tags=20%, list=11%, signal=22% |
| 966 | LEUKOCYTE\_PROLIFERATION(GO:0070661) |  | 40 | 0.34 | 1.13 | 0.224 | 0.587 | 1.000 | 3523 | tags=20%, list=11%, signal=22% |
| 967 | ORGANIC\_ACID:SODIUM\_SYMPORTER\_ACTIVITY(GO:0005343) |  | 22 | 0.38 | 1.13 | 0.324 | 0.590 | 1.000 | 5262 | tags=23%, list=16%, signal=27% |
| 968 | CELL\_CELL\_RECOGNITION(GO:0009988) |  | 15 | 0.44 | 1.12 | 0.214 | 0.590 | 1.000 | 8261 | tags=47%, list=26%, signal=63% |
| 969 | CAMP\_BIOSYNTHETIC\_PROCESS(GO:0006171) |  | 23 | 0.41 | 1.12 | 0.369 | 0.590 | 1.000 | 6847 | tags=35%, list=21%, signal=44% |
| 970 | FATTY\_ACID\_OXIDATION(GO:0019395) |  | 42 | 0.31 | 1.12 | 0.416 | 0.590 | 1.000 | 6664 | tags=29%, list=21%, signal=36% |
| 971 | ENDOPLASMIC\_RETICULUM(GO:0005783) |  | 863 | 0.20 | 1.12 | 0.284 | 0.589 | 1.000 | 4817 | tags=14%, list=15%, signal=16% |
| 972 | ACID\_AMINO\_ACID\_LIGASE\_ACTIVITY(GO:0016881) |  | 17 | 0.34 | 1.12 | 0.273 | 0.589 | 1.000 | 2933 | tags=18%, list=9%, signal=19% |
| 973 | NEGATIVE\_REGULATION\_OF\_INTRINSIC\_APOPTOTIC\_SIGNALING\_PATHWAY(GO:2001243) |  | 22 | 0.31 | 1.12 | 0.239 | 0.589 | 1.000 | 9328 | tags=36%, list=29%, signal=51% |
| 974 | REGULATION\_OF\_T\_CELL\_PROLIFERATION(GO:0042129) |  | 34 | 0.37 | 1.12 | 0.317 | 0.590 | 1.000 | 3523 | tags=24%, list=11%, signal=26% |
| 975 | CELLULAR\_RESPIRATION(GO:0045333) |  | 94 | 0.23 | 1.12 | 0.274 | 0.589 | 1.000 | 3094 | tags=9%, list=10%, signal=9% |
| 976 | OXIDOREDUCTASE\_ACTIVITY\_\_ACTING\_ON\_CH\_OH\_GROUP\_OF\_DONORS(GO:0016614) |  | 122 | 0.26 | 1.12 | 0.252 | 0.589 | 1.000 | 4565 | tags=15%, list=14%, signal=17% |
| 977 | DYNEIN\_INTERMEDIATE\_CHAIN\_BINDING(GO:0045505) |  | 19 | 0.37 | 1.12 | 0.190 | 0.590 | 1.000 | 2623 | tags=11%, list=8%, signal=11% |
| 978 | REGULATION\_OF\_SMOOTHENED\_SIGNALING\_PATHWAY(GO:0008589) |  | 38 | 0.25 | 1.12 | 0.315 | 0.589 | 1.000 | 6244 | tags=18%, list=20%, signal=23% |
| 979 | IMMUNOGLOBULIN\_PRODUCTION(GO:0002377) |  | 26 | 0.37 | 1.12 | 0.333 | 0.589 | 1.000 | 1596 | tags=19%, list=5%, signal=20% |
| 980 | NUCLEASE\_ACTIVITY(GO:0004518) |  | 145 | 0.24 | 1.12 | 0.313 | 0.589 | 1.000 | 4220 | tags=13%, list=13%, signal=15% |
| 981 | 4\_IRON\_\_4\_SULFUR\_CLUSTER\_BINDING(GO:0051539) |  | 39 | 0.28 | 1.12 | 0.245 | 0.589 | 1.000 | 6808 | tags=31%, list=21%, signal=39% |
| 982 | RESPONSE\_TO\_CHEMICAL(GO:0042221) |  | 1592 | 0.18 | 1.12 | 0.154 | 0.590 | 1.000 | 4873 | tags=13%, list=15%, signal=15% |
| 983 | DNA\_TEMPLATED\_TRANSCRIPTIONAL\_PREINITIATION\_COMPLEX\_ASSEMBLY(GO:0070897) |  | 15 | 0.34 | 1.12 | 0.206 | 0.590 | 1.000 | 4047 | tags=20%, list=13%, signal=23% |
| 984 | POSITIVE\_REGULATION\_OF\_CELL\_DEVELOPMENT(GO:0010720) |  | 115 | 0.25 | 1.12 | 0.300 | 0.590 | 1.000 | 3491 | tags=10%, list=11%, signal=11% |
| 985 | RESPONSE\_TO\_VIRUS(GO:0009615) |  | 78 | 0.31 | 1.12 | 0.216 | 0.589 | 1.000 | 9294 | tags=41%, list=29%, signal=58% |
| 986 | MICROTUBULE\_ORGANIZING\_CENTER\_ORGANIZATION(GO:0031023) |  | 61 | 0.31 | 1.12 | 0.363 | 0.589 | 1.000 | 6167 | tags=26%, list=19%, signal=32% |
| 987 | ALPHA\_GLUCOSIDASE\_ACTIVITY(GO:0090599) |  | 20 | 0.29 | 1.12 | 0.275 | 0.590 | 1.000 | 814 | tags=5%, list=3%, signal=5% |
| 988 | HYDROLASE\_ACTIVITY\_\_ACTING\_ON\_ESTER\_BONDS(GO:0016788) |  | 678 | 0.19 | 1.12 | 0.256 | 0.591 | 1.000 | 7212 | tags=21%, list=23%, signal=27% |
| 989 | LYASE\_ACTIVITY(GO:0016829) |  | 181 | 0.26 | 1.12 | 0.316 | 0.591 | 1.000 | 5190 | tags=19%, list=16%, signal=23% |
| 990 | T\_CELL\_PROLIFERATION(GO:0042098) |  | 36 | 0.36 | 1.12 | 0.285 | 0.590 | 1.000 | 3523 | tags=22%, list=11%, signal=25% |
| 991 | COFACTOR\_TRANSMEMBRANE\_TRANSPORTER\_ACTIVITY(GO:0051184) |  | 16 | 0.34 | 1.12 | 0.278 | 0.591 | 1.000 | 3334 | tags=19%, list=10%, signal=21% |
| 992 | REGULATION\_OF\_ACTIN\_FILAMENT\_DEPOLYMERIZATION(GO:0030834) |  | 41 | 0.27 | 1.12 | 0.254 | 0.592 | 1.000 | 3342 | tags=15%, list=10%, signal=16% |
| 993 | NEGATIVE\_REGULATION\_OF\_ACTIN\_FILAMENT\_DEPOLYMERIZATION(GO:0030835) |  | 41 | 0.27 | 1.12 | 0.254 | 0.591 | 1.000 | 3342 | tags=15%, list=10%, signal=16% |
| 994 | IMMUNE\_RESPONSE(GO:0006955) |  | 465 | 0.24 | 1.12 | 0.109 | 0.591 | 1.000 | 7328 | tags=31%, list=23%, signal=39% |
| 995 | ORGANIC\_ACID\_BIOSYNTHETIC\_PROCESS(GO:0016053) |  | 219 | 0.24 | 1.12 | 0.264 | 0.591 | 1.000 | 6095 | tags=22%, list=19%, signal=27% |
| 996 | POSITIVE\_REGULATION\_OF\_MACROMOLECULE\_METABOLIC\_PROCESS(GO:0010604) |  | 954 | 0.19 | 1.12 | 0.219 | 0.591 | 1.000 | 7125 | tags=20%, list=22%, signal=24% |
| 997 | DEMETHYLASE\_ACTIVITY(GO:0032451) |  | 32 | 0.33 | 1.12 | 0.311 | 0.592 | 1.000 | 6954 | tags=19%, list=22%, signal=24% |
| 998 | REGULATION\_OF\_TRANSCRIPTION\_FROM\_RNA\_POLYMERASE\_II\_PROMOTER\_INVOLVED\_IN\_DETERMINATION\_OF\_LEFT\_RIGHT\_SYMMETRY(GO:1900094) |  | 15 | 0.38 | 1.12 | 0.353 | 0.592 | 1.000 | 10645 | tags=53%, list=33%, signal=80% |
| 999 | RESPONSE\_TO\_ABIOTIC\_STIMULUS(GO:0009628) |  | 345 | 0.19 | 1.12 | 0.227 | 0.592 | 1.000 | 7949 | tags=24%, list=25%, signal=32% |
| 1000 | CELLULAR\_NITROGEN\_COMPOUND\_CATABOLIC\_PROCESS(GO:0044270) |  | 285 | 0.19 | 1.12 | 0.258 | 0.592 | 1.000 | 4621 | tags=12%, list=14%, signal=13% |
| 1001 | CHROMATIN\_DNA\_BINDING(GO:0031490) |  | 56 | 0.29 | 1.11 | 0.272 | 0.594 | 1.000 | 1475 | tags=9%, list=5%, signal=9% |
| 1002 | RECEPTOR\_MEDIATED\_ENDOCYTOSIS(GO:0006898) |  | 91 | 0.27 | 1.11 | 0.278 | 0.594 | 1.000 | 4795 | tags=15%, list=15%, signal=18% |
| 1003 | CELLULAR\_RESPONSE\_TO\_GLUCOSE\_STARVATION(GO:0042149) |  | 18 | 0.40 | 1.11 | 0.291 | 0.593 | 1.000 | 4510 | tags=28%, list=14%, signal=32% |
| 1004 | MATURATION\_OF\_5.8S\_RRNA(GO:0000460) |  | 30 | 0.35 | 1.11 | 0.335 | 0.595 | 1.000 | 715 | tags=7%, list=2%, signal=7% |
| 1005 | ENDOMEMBRANE\_SYSTEM(GO:0012505) |  | 2016 | 0.17 | 1.11 | 0.293 | 0.594 | 1.000 | 4817 | tags=12%, list=15%, signal=13% |
| 1006 | HEPATICOBILIARY\_SYSTEM\_DEVELOPMENT(GO:0061008) |  | 151 | 0.21 | 1.11 | 0.316 | 0.595 | 1.000 | 5983 | tags=17%, list=19%, signal=21% |
| 1007 | REGULATION\_OF\_CELL\_DEATH(GO:0010941) |  | 399 | 0.19 | 1.11 | 0.164 | 0.597 | 1.000 | 7470 | tags=23%, list=23%, signal=29% |
| 1008 | ANTIGEN\_PROCESSING\_AND\_PRESENTATION\_OF\_PEPTIDE\_ANTIGEN(GO:0048002) |  | 21 | 0.34 | 1.11 | 0.216 | 0.596 | 1.000 | 1541 | tags=19%, list=5%, signal=20% |
| 1009 | REGULATION\_OF\_BODY\_FLUID\_LEVELS(GO:0050878) |  | 75 | 0.30 | 1.11 | 0.291 | 0.596 | 1.000 | 3441 | tags=17%, list=11%, signal=19% |
| 1010 | COATED\_VESICLE(GO:0030135) |  | 118 | 0.19 | 1.11 | 0.219 | 0.596 | 1.000 | 5433 | tags=14%, list=17%, signal=17% |
| 1011 | PEPTIDE\_METABOLIC\_PROCESS(GO:0006518) |  | 518 | 0.19 | 1.11 | 0.330 | 0.597 | 1.000 | 2662 | tags=6%, list=8%, signal=6% |
| 1012 | NEGATIVE\_REGULATION\_OF\_CELL\_DEVELOPMENT(GO:0010721) |  | 78 | 0.30 | 1.11 | 0.277 | 0.597 | 1.000 | 2608 | tags=9%, list=8%, signal=10% |
| 1013 | AMEBOIDAL\_TYPE\_CELL\_MIGRATION(GO:0001667) |  | 237 | 0.24 | 1.11 | 0.349 | 0.597 | 1.000 | 5098 | tags=14%, list=16%, signal=17% |
| 1014 | CYTOPLASMIC\_VESICLE\_PART(GO:0044433) |  | 356 | 0.17 | 1.11 | 0.158 | 0.600 | 1.000 | 4443 | tags=10%, list=14%, signal=12% |
| 1015 | POSITIVE\_REGULATION\_OF\_GTPASE\_ACTIVITY(GO:0043547) |  | 126 | 0.24 | 1.11 | 0.254 | 0.600 | 1.000 | 8245 | tags=26%, list=26%, signal=35% |
| 1016 | L\_ALPHA\_AMINO\_ACID\_TRANSMEMBRANE\_TRANSPORT(GO:1902475) |  | 17 | 0.39 | 1.11 | 0.282 | 0.603 | 1.000 | 1236 | tags=12%, list=4%, signal=12% |
| 1017 | GLYCOSAMINOGLYCAN\_BIOSYNTHETIC\_PROCESS(GO:0006024) |  | 28 | 0.36 | 1.11 | 0.405 | 0.603 | 1.000 | 7006 | tags=39%, list=22%, signal=50% |
| 1018 | APOPTOTIC\_CELL\_CLEARANCE(GO:0043277) |  | 32 | 0.28 | 1.11 | 0.292 | 0.603 | 1.000 | 5211 | tags=16%, list=16%, signal=19% |
| 1019 | REGULATION\_OF\_NEUROTRANSMITTER\_SECRETION(GO:0046928) |  | 25 | 0.32 | 1.11 | 0.353 | 0.603 | 1.000 | 2932 | tags=12%, list=9%, signal=13% |
| 1020 | DRUG\_BINDING(GO:0008144) |  | 1579 | 0.16 | 1.11 | 0.191 | 0.604 | 1.000 | 7516 | tags=21%, list=24%, signal=26% |
| 1021 | DNA\_DEPENDENT\_DNA\_REPLICATION(GO:0006261) |  | 83 | 0.21 | 1.11 | 0.310 | 0.605 | 1.000 | 3464 | tags=8%, list=11%, signal=9% |
| 1022 | CARDIAC\_VENTRICLE\_MORPHOGENESIS(GO:0003208) |  | 18 | 0.41 | 1.11 | 0.385 | 0.604 | 1.000 | 3651 | tags=17%, list=11%, signal=19% |
| 1023 | CARBON\_CARBON\_LYASE\_ACTIVITY(GO:0016830) |  | 49 | 0.31 | 1.11 | 0.370 | 0.604 | 1.000 | 10520 | tags=45%, list=33%, signal=67% |
| 1024 | CHROMOSOME\_ORGANIZATION\_INVOLVED\_IN\_MEIOTIC\_CELL\_CYCLE(GO:0070192) |  | 29 | 0.32 | 1.11 | 0.258 | 0.604 | 1.000 | 11765 | tags=55%, list=37%, signal=87% |
| 1025 | POSITIVE\_REGULATION\_OF\_IMMUNE\_RESPONSE(GO:0050778) |  | 152 | 0.25 | 1.10 | 0.226 | 0.605 | 1.000 | 7485 | tags=36%, list=23%, signal=47% |
| 1026 | MITOCHONDRIAL\_MEMBRANE\_ORGANIZATION(GO:0007006) |  | 39 | 0.25 | 1.10 | 0.410 | 0.605 | 1.000 | 4475 | tags=15%, list=14%, signal=18% |
| 1027 | PROTEIN\_TRANSPORT\_WITHIN\_LIPID\_BILAYER(GO:0032594) |  | 24 | 0.35 | 1.10 | 0.329 | 0.606 | 1.000 | 4426 | tags=21%, list=14%, signal=24% |
| 1028 | REGULATION\_OF\_PROTEIN\_KINASE\_ACTIVITY(GO:0045859) |  | 136 | 0.23 | 1.10 | 0.161 | 0.606 | 1.000 | 7756 | tags=24%, list=24%, signal=32% |
| 1029 | AMMONIUM\_ION\_METABOLIC\_PROCESS(GO:0097164) |  | 84 | 0.26 | 1.10 | 0.230 | 0.607 | 1.000 | 4234 | tags=14%, list=13%, signal=16% |
| 1030 | THIGMOTAXIS(GO:0001966) |  | 17 | 0.39 | 1.10 | 0.335 | 0.608 | 1.000 | 955 | tags=12%, list=3%, signal=12% |
| 1031 | PHOSPHATASE\_INHIBITOR\_ACTIVITY(GO:0019212) |  | 33 | 0.26 | 1.10 | 0.279 | 0.608 | 1.000 | 5099 | tags=18%, list=16%, signal=22% |
| 1032 | REGULATION\_OF\_OSSIFICATION(GO:0030278) |  | 31 | 0.26 | 1.10 | 0.353 | 0.608 | 1.000 | 6409 | tags=26%, list=20%, signal=32% |
| 1033 | MYELOID\_CELL\_DEVELOPMENT(GO:0061515) |  | 69 | 0.23 | 1.10 | 0.327 | 0.608 | 1.000 | 6876 | tags=22%, list=22%, signal=28% |
| 1034 | NUCLEOSIDE\_DIPHOSPHATASE\_ACTIVITY(GO:0017110) |  | 15 | 0.44 | 1.10 | 0.431 | 0.608 | 1.000 | 9674 | tags=60%, list=30%, signal=86% |
| 1035 | PROTEIN\_ACYLATION(GO:0043543) |  | 113 | 0.23 | 1.10 | 0.249 | 0.607 | 1.000 | 9060 | tags=27%, list=28%, signal=37% |
| 1036 | MANGANESE\_ION\_TRANSPORT(GO:0006828) |  | 15 | 0.41 | 1.10 | 0.305 | 0.609 | 1.000 | 4409 | tags=20%, list=14%, signal=23% |
| 1037 | STRESS\_FIBER(GO:0001725) |  | 31 | 0.30 | 1.10 | 0.391 | 0.609 | 1.000 | 4805 | tags=16%, list=15%, signal=19% |
| 1038 | CONTRACTILE\_ACTIN\_FILAMENT\_BUNDLE(GO:0097517) |  | 31 | 0.30 | 1.10 | 0.391 | 0.609 | 1.000 | 4805 | tags=16%, list=15%, signal=19% |
| 1039 | ENDOSOMAL\_PART(GO:0044440) |  | 186 | 0.19 | 1.10 | 0.219 | 0.609 | 1.000 | 3942 | tags=10%, list=12%, signal=12% |
| 1040 | LONG\_CHAIN\_FATTY\_ACID\_METABOLIC\_PROCESS(GO:0001676) |  | 23 | 0.41 | 1.10 | 0.305 | 0.609 | 1.000 | 6866 | tags=35%, list=22%, signal=44% |
| 1041 | CARBOXYLIC\_ACID\_BIOSYNTHETIC\_PROCESS(GO:0046394) |  | 217 | 0.24 | 1.10 | 0.263 | 0.611 | 1.000 | 6095 | tags=22%, list=19%, signal=27% |
| 1042 | CELL\_JUNCTION\_ORGANIZATION(GO:0034330) |  | 112 | 0.23 | 1.10 | 0.367 | 0.610 | 1.000 | 4945 | tags=13%, list=16%, signal=16% |
| 1043 | LYSOSOMAL\_TRANSPORT(GO:0007041) |  | 42 | 0.27 | 1.10 | 0.302 | 0.613 | 1.000 | 2336 | tags=10%, list=7%, signal=10% |
| 1044 | CARDIAC\_CHAMBER\_MORPHOGENESIS(GO:0003206) |  | 28 | 0.35 | 1.10 | 0.386 | 0.615 | 1.000 | 3651 | tags=14%, list=11%, signal=16% |
| 1045 | ERYTHROCYTE\_MATURATION(GO:0043249) |  | 21 | 0.27 | 1.10 | 0.348 | 0.615 | 1.000 | 5157 | tags=24%, list=16%, signal=28% |
| 1046 | POSITIVE\_REGULATION\_OF\_CELL\_MIGRATION(GO:0030335) |  | 91 | 0.25 | 1.09 | 0.236 | 0.618 | 1.000 | 1084 | tags=5%, list=3%, signal=6% |
| 1047 | VOLTAGE\_GATED\_ANION\_CHANNEL\_ACTIVITY(GO:0008308) |  | 17 | 0.37 | 1.09 | 0.347 | 0.617 | 1.000 | 6400 | tags=29%, list=20%, signal=37% |
| 1048 | C\_C\_CHEMOKINE\_BINDING(GO:0019957) |  | 21 | 0.40 | 1.09 | 0.365 | 0.619 | 1.000 | 4577 | tags=29%, list=14%, signal=33% |
| 1049 | NCRNA\_3'\_END\_PROCESSING(GO:0043628) |  | 29 | 0.31 | 1.09 | 0.400 | 0.618 | 1.000 | 1460 | tags=7%, list=5%, signal=7% |
| 1050 | NEGATIVE\_REGULATION\_OF\_RESPONSE\_TO\_EXTERNAL\_STIMULUS(GO:0032102) |  | 70 | 0.26 | 1.09 | 0.244 | 0.618 | 1.000 | 3433 | tags=11%, list=11%, signal=13% |
| 1051 | CELL\_CELL\_JUNCTION(GO:0005911) |  | 190 | 0.21 | 1.09 | 0.345 | 0.618 | 1.000 | 7973 | tags=23%, list=25%, signal=31% |
| 1052 | TRANSMEMBRANE\_RECEPTOR\_PROTEIN\_TYROSINE\_KINASE\_ACTIVITY(GO:0004714) |  | 73 | 0.31 | 1.09 | 0.392 | 0.618 | 1.000 | 7642 | tags=27%, list=24%, signal=36% |
| 1053 | INTRACELLULAR\_PROTEIN\_TRANSMEMBRANE\_TRANSPORT(GO:0065002) |  | 46 | 0.26 | 1.09 | 0.342 | 0.618 | 1.000 | 3926 | tags=11%, list=12%, signal=12% |
| 1054 | PROTEIN\_TRANSMEMBRANE\_TRANSPORT(GO:0071806) |  | 46 | 0.26 | 1.09 | 0.342 | 0.617 | 1.000 | 3926 | tags=11%, list=12%, signal=12% |
| 1055 | TRANSCRIPTIONAL\_REPRESSOR\_ACTIVITY\_\_RNA\_POLYMERASE\_II\_TRANSCRIPTION\_REGULATORY\_REGION\_SEQUENCE\_SPECIFIC\_DNA\_BINDING(GO:0001227) |  | 47 | 0.27 | 1.09 | 0.343 | 0.618 | 1.000 | 5559 | tags=21%, list=17%, signal=26% |
| 1056 | MESODERM\_FORMATION(GO:0001707) |  | 43 | 0.29 | 1.09 | 0.331 | 0.618 | 1.000 | 9159 | tags=35%, list=29%, signal=49% |
| 1057 | POSITIVE\_REGULATION\_OF\_LEUKOCYTE\_MIGRATION(GO:0002687) |  | 24 | 0.33 | 1.09 | 0.226 | 0.618 | 1.000 | 2083 | tags=13%, list=7%, signal=13% |
| 1058 | ADHERENS\_JUNCTION\_ORGANIZATION(GO:0034332) |  | 26 | 0.30 | 1.09 | 0.294 | 0.618 | 1.000 | 9828 | tags=31%, list=31%, signal=44% |
| 1059 | SPINAL\_CORD\_DEVELOPMENT(GO:0021510) |  | 84 | 0.26 | 1.09 | 0.377 | 0.619 | 1.000 | 7626 | tags=24%, list=24%, signal=31% |
| 1060 | CALCIUM\_DEPENDENT\_PROTEIN\_KINASE\_ACTIVITY(GO:0010857) |  | 31 | 0.30 | 1.09 | 0.362 | 0.620 | 1.000 | 7206 | tags=29%, list=23%, signal=37% |
| 1061 | ATRIOVENTRICULAR\_VALVE\_MORPHOGENESIS(GO:0003181) |  | 17 | 0.29 | 1.09 | 0.307 | 0.621 | 1.000 | 3091 | tags=12%, list=10%, signal=13% |
| 1062 | CALCIUM:CATION\_ANTIPORTER\_ACTIVITY(GO:0015368) |  | 17 | 0.43 | 1.09 | 0.369 | 0.623 | 1.000 | 6987 | tags=47%, list=22%, signal=60% |
| 1063 | L\_AMINO\_ACID\_TRANSPORT(GO:0015807) |  | 23 | 0.33 | 1.09 | 0.407 | 0.623 | 1.000 | 1236 | tags=9%, list=4%, signal=9% |
| 1064 | CARDIAC\_CHAMBER\_DEVELOPMENT(GO:0003205) |  | 45 | 0.29 | 1.09 | 0.332 | 0.623 | 1.000 | 3651 | tags=13%, list=11%, signal=15% |
| 1065 | NEURAL\_CREST\_FORMATION(GO:0014029) |  | 17 | 0.27 | 1.09 | 0.330 | 0.623 | 1.000 | 1891 | tags=6%, list=6%, signal=6% |
| 1066 | BICARBONATE\_TRANSMEMBRANE\_TRANSPORTER\_ACTIVITY(GO:0015106) |  | 18 | 0.45 | 1.09 | 0.354 | 0.623 | 1.000 | 8935 | tags=50%, list=28%, signal=69% |
| 1067 | RESPONSE\_TO\_LIGHT\_STIMULUS(GO:0009416) |  | 130 | 0.24 | 1.09 | 0.362 | 0.624 | 1.000 | 9734 | tags=31%, list=31%, signal=44% |
| 1068 | CELL\_CELL\_JUNCTION\_ASSEMBLY(GO:0007043) |  | 74 | 0.26 | 1.09 | 0.395 | 0.625 | 1.000 | 4945 | tags=18%, list=16%, signal=21% |
| 1069 | SPECIFICATION\_OF\_SYMMETRY(GO:0009799) |  | 278 | 0.19 | 1.08 | 0.350 | 0.625 | 1.000 | 7470 | tags=21%, list=23%, signal=27% |
| 1070 | DETERMINATION\_OF\_BILATERAL\_SYMMETRY(GO:0009855) |  | 278 | 0.19 | 1.08 | 0.350 | 0.624 | 1.000 | 7470 | tags=21%, list=23%, signal=27% |
| 1071 | OXIDOREDUCTASE\_ACTIVITY\_\_ACTING\_ON\_PAIRED\_DONORS\_\_WITH\_INCORPORATION\_OR\_REDUCTION\_OF\_MOLECULAR\_OXYGEN\_\_REDUCED\_FLAVIN\_OR\_FLAVOPROTEIN\_AS\_ONE\_DONOR\_\_AND\_INCORPORATION\_OF\_ONE\_ATOM\_OF\_OXYGEN(GO:0016712) |  | 44 | 0.35 | 1.08 | 0.324 | 0.625 | 1.000 | 6399 | tags=39%, list=20%, signal=48% |
| 1072 | CATECHOLAMINE\_METABOLIC\_PROCESS(GO:0006584) |  | 17 | 0.44 | 1.08 | 0.415 | 0.624 | 1.000 | 4234 | tags=24%, list=13%, signal=27% |
| 1073 | CATECHOL\_CONTAINING\_COMPOUND\_METABOLIC\_PROCESS(GO:0009712) |  | 17 | 0.44 | 1.08 | 0.415 | 0.624 | 1.000 | 4234 | tags=24%, list=13%, signal=27% |
| 1074 | POLYOL\_CATABOLIC\_PROCESS(GO:0046174) |  | 20 | 0.31 | 1.08 | 0.289 | 0.624 | 1.000 | 4470 | tags=20%, list=14%, signal=23% |
| 1075 | CYCLIC\_NUCLEOTIDE\_BIOSYNTHETIC\_PROCESS(GO:0009190) |  | 45 | 0.30 | 1.08 | 0.272 | 0.625 | 1.000 | 8949 | tags=33%, list=28%, signal=46% |
| 1076 | XENOBIOTIC\_METABOLIC\_PROCESS(GO:0006805) |  | 51 | 0.32 | 1.08 | 0.313 | 0.625 | 1.000 | 6399 | tags=35%, list=20%, signal=44% |
| 1077 | POSITIVE\_REGULATION\_OF\_BLOOD\_CIRCULATION(GO:1903524) |  | 17 | 0.40 | 1.08 | 0.316 | 0.624 | 1.000 | 10070 | tags=59%, list=32%, signal=86% |
| 1078 | CYTOKINE\_RECEPTOR\_BINDING(GO:0005126) |  | 98 | 0.29 | 1.08 | 0.325 | 0.624 | 1.000 | 6757 | tags=23%, list=21%, signal=30% |
| 1079 | EPITHELIUM\_MIGRATION(GO:0090132) |  | 67 | 0.27 | 1.08 | 0.277 | 0.624 | 1.000 | 7020 | tags=22%, list=22%, signal=29% |
| 1080 | COA\_HYDROLASE\_ACTIVITY(GO:0016289) |  | 17 | 0.35 | 1.08 | 0.335 | 0.624 | 1.000 | 5292 | tags=35%, list=17%, signal=42% |
| 1081 | EARLY\_ENDOSOME(GO:0005769) |  | 125 | 0.20 | 1.08 | 0.271 | 0.624 | 1.000 | 7462 | tags=21%, list=23%, signal=27% |
| 1082 | REGULATION\_OF\_LIPID\_METABOLIC\_PROCESS(GO:0019216) |  | 70 | 0.21 | 1.08 | 0.371 | 0.624 | 1.000 | 4059 | tags=10%, list=13%, signal=11% |
| 1083 | PHOSPHATIDYLSERINE\_BINDING(GO:0001786) |  | 25 | 0.38 | 1.08 | 0.457 | 0.624 | 1.000 | 672 | tags=8%, list=2%, signal=8% |
| 1084 | FATTY\_ACID\_ELONGATION(GO:0030497) |  | 16 | 0.43 | 1.08 | 0.447 | 0.625 | 1.000 | 4729 | tags=38%, list=15%, signal=44% |
| 1085 | ACTIN\_FILAMENT\_BASED\_MOVEMENT(GO:0030048) |  | 43 | 0.27 | 1.08 | 0.388 | 0.624 | 1.000 | 10311 | tags=37%, list=32%, signal=55% |
| 1086 | FATTY\_ACID\_BETA\_OXIDATION(GO:0006635) |  | 35 | 0.29 | 1.08 | 0.384 | 0.626 | 1.000 | 4565 | tags=20%, list=14%, signal=23% |
| 1087 | XENOBIOTIC\_CATABOLIC\_PROCESS(GO:0042178) |  | 38 | 0.34 | 1.08 | 0.307 | 0.626 | 1.000 | 6399 | tags=39%, list=20%, signal=49% |
| 1088 | EXTRACELLULAR\_MATRIX(GO:0031012) |  | 223 | 0.24 | 1.08 | 0.371 | 0.628 | 1.000 | 6651 | tags=22%, list=21%, signal=28% |
| 1089 | CYCLIC\_PURINE\_NUCLEOTIDE\_METABOLIC\_PROCESS(GO:0052652) |  | 44 | 0.31 | 1.08 | 0.272 | 0.628 | 1.000 | 8949 | tags=34%, list=28%, signal=47% |
| 1090 | NEGATIVE\_REGULATION\_OF\_CELL\_DIFFERENTIATION(GO:0045596) |  | 127 | 0.24 | 1.08 | 0.341 | 0.629 | 1.000 | 3433 | tags=9%, list=11%, signal=11% |
| 1091 | TRANSPORT(GO:0006810) |  | 2759 | 0.18 | 1.08 | 0.251 | 0.629 | 1.000 | 4817 | tags=12%, list=15%, signal=13% |
| 1092 | PROTEIN\_KINASE\_C\_ACTIVITY(GO:0004697) |  | 22 | 0.32 | 1.08 | 0.387 | 0.630 | 1.000 | 6982 | tags=32%, list=22%, signal=41% |
| 1093 | CALCIUM\_DEPENDENT\_PROTEIN\_KINASE\_C\_ACTIVITY(GO:0004698) |  | 22 | 0.32 | 1.08 | 0.387 | 0.629 | 1.000 | 6982 | tags=32%, list=22%, signal=41% |
| 1094 | TRICARBOXYLIC\_ACID\_CYCLE(GO:0006099) |  | 29 | 0.29 | 1.08 | 0.304 | 0.629 | 1.000 | 3693 | tags=17%, list=12%, signal=19% |
| 1095 | LOCALIZATION(GO:0051179) |  | 3715 | 0.17 | 1.08 | 0.291 | 0.629 | 1.000 | 4817 | tags=12%, list=15%, signal=12% |
| 1096 | REGULATION\_OF\_TRANSLATION(GO:0006417) |  | 175 | 0.21 | 1.08 | 0.315 | 0.629 | 1.000 | 6119 | tags=14%, list=19%, signal=17% |
| 1097 | UBIQUITIN\_LIKE\_PROTEIN\_SPECIFIC\_PROTEASE\_ACTIVITY(GO:0019783) |  | 94 | 0.21 | 1.08 | 0.367 | 0.629 | 1.000 | 2919 | tags=7%, list=9%, signal=8% |
| 1098 | HISTONE\_METHYLATION(GO:0016571) |  | 58 | 0.21 | 1.07 | 0.329 | 0.632 | 1.000 | 9941 | tags=28%, list=31%, signal=40% |
| 1099 | CELLULAR\_LIPID\_CATABOLIC\_PROCESS(GO:0044242) |  | 121 | 0.25 | 1.07 | 0.373 | 0.633 | 1.000 | 7175 | tags=25%, list=22%, signal=32% |
| 1100 | NEGATIVE\_REGULATION\_OF\_PROTEIN\_COMPLEX\_DISASSEMBLY(GO:0043242) |  | 50 | 0.25 | 1.07 | 0.310 | 0.632 | 1.000 | 3342 | tags=12%, list=10%, signal=13% |
| 1101 | CENTROSOME\_CYCLE(GO:0007098) |  | 55 | 0.32 | 1.07 | 0.390 | 0.633 | 1.000 | 6167 | tags=27%, list=19%, signal=34% |
| 1102 | MONOCYTE\_CHEMOTAXIS(GO:0002548) |  | 23 | 0.38 | 1.07 | 0.242 | 0.633 | 1.000 | 2466 | tags=22%, list=8%, signal=24% |
| 1103 | PEPTIDE\_HORMONE\_BINDING(GO:0017046) |  | 29 | 0.34 | 1.07 | 0.300 | 0.633 | 1.000 | 10076 | tags=52%, list=32%, signal=76% |
| 1104 | LEUKOCYTE\_MIGRATION(GO:0050900) |  | 127 | 0.22 | 1.07 | 0.255 | 0.635 | 1.000 | 2466 | tags=9%, list=8%, signal=10% |
| 1105 | POSITIVE\_REGULATION\_OF\_ERK1\_AND\_ERK2\_CASCADE(GO:0070374) |  | 60 | 0.32 | 1.07 | 0.416 | 0.635 | 1.000 | 5055 | tags=23%, list=16%, signal=28% |
| 1106 | POSITIVE\_REGULATION\_OF\_RESPONSE\_TO\_EXTERNAL\_STIMULUS(GO:0032103) |  | 24 | 0.36 | 1.07 | 0.435 | 0.637 | 1.000 | 953 | tags=13%, list=3%, signal=13% |
| 1107 | CILIARY\_BASAL\_BODY(GO:0036064) |  | 66 | 0.24 | 1.07 | 0.329 | 0.638 | 1.000 | 2171 | tags=9%, list=7%, signal=10% |
| 1108 | DETERMINATION\_OF\_LEFT\_RIGHT\_SYMMETRY(GO:0007368) |  | 253 | 0.20 | 1.07 | 0.353 | 0.638 | 1.000 | 7160 | tags=20%, list=22%, signal=25% |
| 1109 | S\_ACYLTRANSFERASE\_ACTIVITY(GO:0016417) |  | 33 | 0.29 | 1.07 | 0.399 | 0.639 | 1.000 | 8160 | tags=36%, list=26%, signal=49% |
| 1110 | DNA\_PACKAGING(GO:0006323) |  | 84 | 0.26 | 1.07 | 0.399 | 0.639 | 1.000 | 941 | tags=5%, list=3%, signal=5% |
| 1111 | STRESS\_ACTIVATED\_PROTEIN\_KINASE\_SIGNALING\_CASCADE(GO:0031098) |  | 50 | 0.28 | 1.07 | 0.425 | 0.639 | 1.000 | 4176 | tags=18%, list=13%, signal=21% |
| 1112 | STRESS\_ACTIVATED\_MAPK\_CASCADE(GO:0051403) |  | 50 | 0.28 | 1.07 | 0.425 | 0.639 | 1.000 | 4176 | tags=18%, list=13%, signal=21% |
| 1113 | MACROPHAGE\_DIFFERENTIATION(GO:0030225) |  | 18 | 0.38 | 1.07 | 0.409 | 0.639 | 1.000 | 5643 | tags=28%, list=18%, signal=34% |
| 1114 | REGULATION\_OF\_PROTEIN\_SERINE\_THREONINE\_KINASE\_ACTIVITY(GO:0071900) |  | 84 | 0.24 | 1.07 | 0.259 | 0.638 | 1.000 | 4879 | tags=17%, list=15%, signal=20% |
| 1115 | ESTABLISHMENT\_OF\_LOCALIZATION(GO:0051234) |  | 2823 | 0.18 | 1.07 | 0.251 | 0.639 | 1.000 | 4817 | tags=12%, list=15%, signal=13% |
| 1116 | LYMPHOCYTE\_CHEMOTAXIS(GO:0048247) |  | 23 | 0.38 | 1.07 | 0.346 | 0.639 | 1.000 | 2466 | tags=22%, list=8%, signal=24% |
| 1117 | DYNEIN\_COMPLEX(GO:0030286) |  | 39 | 0.28 | 1.07 | 0.356 | 0.639 | 1.000 | 6675 | tags=26%, list=21%, signal=32% |
| 1118 | POSITIVE\_REGULATION\_OF\_MRNA\_METABOLIC\_PROCESS(GO:1903313) |  | 31 | 0.28 | 1.07 | 0.326 | 0.639 | 1.000 | 913 | tags=6%, list=3%, signal=7% |
| 1119 | POSITIVE\_REGULATION\_OF\_MULTICELLULAR\_ORGANISMAL\_PROCESS(GO:0051240) |  | 296 | 0.21 | 1.07 | 0.370 | 0.641 | 1.000 | 7979 | tags=24%, list=25%, signal=32% |
| 1120 | HISTONE\_LYSINE\_N\_METHYLTRANSFERASE\_ACTIVITY(GO:0018024) |  | 38 | 0.27 | 1.07 | 0.400 | 0.642 | 1.000 | 9101 | tags=21%, list=29%, signal=29% |
| 1121 | CELLULAR\_ALDEHYDE\_METABOLIC\_PROCESS(GO:0006081) |  | 48 | 0.29 | 1.07 | 0.385 | 0.642 | 1.000 | 3693 | tags=17%, list=12%, signal=19% |
| 1122 | MACROMOLECULE\_MODIFICATION(GO:0043412) |  | 2519 | 0.15 | 1.07 | 0.268 | 0.642 | 1.000 | 7143 | tags=18%, list=22%, signal=21% |
| 1123 | CELL\_SURFACE\_RECEPTOR\_SIGNALING\_PATHWAY\_INVOLVED\_IN\_HEART\_DEVELOPMENT(GO:0061311) |  | 24 | 0.33 | 1.06 | 0.367 | 0.641 | 1.000 | 9159 | tags=29%, list=29%, signal=41% |
| 1124 | CYTOSKELETAL\_PART(GO:0044430) |  | 843 | 0.16 | 1.06 | 0.267 | 0.642 | 1.000 | 4923 | tags=11%, list=15%, signal=13% |
| 1125 | RESPONSE\_TO\_CADMIUM\_ION(GO:0046686) |  | 15 | 0.31 | 1.06 | 0.372 | 0.641 | 1.000 | 1914 | tags=13%, list=6%, signal=14% |
| 1126 | RNA\_CATABOLIC\_PROCESS(GO:0006401) |  | 140 | 0.18 | 1.06 | 0.309 | 0.641 | 1.000 | 1460 | tags=4%, list=5%, signal=4% |
| 1127 | MRNA\_BINDING(GO:0003729) |  | 215 | 0.16 | 1.06 | 0.392 | 0.643 | 1.000 | 6833 | tags=13%, list=21%, signal=16% |
| 1128 | CHONDROITIN\_SULFATE\_PROTEOGLYCAN\_METABOLIC\_PROCESS(GO:0050654) |  | 24 | 0.29 | 1.06 | 0.354 | 0.643 | 1.000 | 7006 | tags=29%, list=22%, signal=37% |
| 1129 | MITOTIC\_CELL\_CYCLE\_PROCESS(GO:1903047) |  | 311 | 0.17 | 1.06 | 0.276 | 0.646 | 1.000 | 7470 | tags=18%, list=23%, signal=23% |
| 1130 | CELLULAR\_RESPONSE\_TO\_INTERLEUKIN\_1(GO:0071347) |  | 27 | 0.36 | 1.06 | 0.327 | 0.647 | 1.000 | 2466 | tags=19%, list=8%, signal=20% |
| 1131 | STRUCTURAL\_CONSTITUENT\_OF\_CYTOSKELETON(GO:0005200) |  | 34 | 0.23 | 1.06 | 0.416 | 0.647 | 1.000 | 3910 | tags=15%, list=12%, signal=17% |
| 1132 | CENTROMERE\_COMPLEX\_ASSEMBLY(GO:0034508) |  | 18 | 0.33 | 1.06 | 0.374 | 0.647 | 1.000 | 12831 | tags=56%, list=40%, signal=93% |
| 1133 | POSITIVE\_REGULATION\_OF\_MAPK\_CASCADE(GO:0043410) |  | 116 | 0.26 | 1.06 | 0.377 | 0.647 | 1.000 | 4727 | tags=19%, list=15%, signal=22% |
| 1134 | CELL\_MIGRATION\_INVOLVED\_IN\_GASTRULATION(GO:0042074) |  | 73 | 0.25 | 1.06 | 0.443 | 0.647 | 1.000 | 4398 | tags=15%, list=14%, signal=17% |
| 1135 | REGULATION\_OF\_PROGRAMMED\_CELL\_DEATH(GO:0043067) |  | 372 | 0.18 | 1.06 | 0.219 | 0.647 | 1.000 | 7470 | tags=23%, list=23%, signal=29% |
| 1136 | ERBB\_SIGNALING\_PATHWAY(GO:0038127) |  | 30 | 0.32 | 1.06 | 0.372 | 0.647 | 1.000 | 6875 | tags=33%, list=22%, signal=42% |
| 1137 | NEGATIVE\_REGULATION\_OF\_PROTEIN\_DEPOLYMERIZATION(GO:1901880) |  | 47 | 0.25 | 1.06 | 0.252 | 0.647 | 1.000 | 3342 | tags=13%, list=10%, signal=14% |
| 1138 | POSITIVE\_REGULATION\_OF\_CELLULAR\_COMPONENT\_MOVEMENT(GO:0051272) |  | 100 | 0.24 | 1.06 | 0.378 | 0.647 | 1.000 | 2608 | tags=8%, list=8%, signal=9% |
| 1139 | FATTY\_ACID\_TRANSPORT(GO:0015908) |  | 28 | 0.34 | 1.06 | 0.411 | 0.648 | 1.000 | 2057 | tags=18%, list=6%, signal=19% |
| 1140 | NEGATIVE\_REGULATION\_OF\_CATABOLIC\_PROCESS(GO:0009895) |  | 60 | 0.19 | 1.06 | 0.381 | 0.651 | 1.000 | 10234 | tags=30%, list=32%, signal=44% |
| 1141 | C\_C\_CHEMOKINE\_RECEPTOR\_ACTIVITY(GO:0016493) |  | 22 | 0.38 | 1.06 | 0.347 | 0.651 | 1.000 | 4577 | tags=27%, list=14%, signal=32% |
| 1142 | RECEPTOR\_LIGAND\_ACTIVITY(GO:0048018) |  | 358 | 0.22 | 1.06 | 0.320 | 0.652 | 1.000 | 4503 | tags=14%, list=14%, signal=16% |
| 1143 | PROTEIN\_N\_LINKED\_GLYCOSYLATION(GO:0006487) |  | 47 | 0.29 | 1.06 | 0.382 | 0.652 | 1.000 | 4078 | tags=15%, list=13%, signal=17% |
| 1144 | ANION\_TRANSMEMBRANE\_TRANSPORTER\_ACTIVITY(GO:0008509) |  | 284 | 0.22 | 1.06 | 0.387 | 0.652 | 1.000 | 4113 | tags=13%, list=13%, signal=14% |
| 1145 | GLIAL\_CELL\_DIFFERENTIATION(GO:0010001) |  | 134 | 0.22 | 1.06 | 0.366 | 0.652 | 1.000 | 8146 | tags=27%, list=26%, signal=36% |
| 1146 | CELL\_CYCLE\_CHECKPOINT(GO:0000075) |  | 68 | 0.24 | 1.06 | 0.319 | 0.651 | 1.000 | 4148 | tags=13%, list=13%, signal=15% |
| 1147 | TRANSFERASE\_ACTIVITY\_\_TRANSFERRING\_PHOSPHORUS\_CONTAINING\_GROUPS(GO:0016772) |  | 1010 | 0.15 | 1.06 | 0.361 | 0.651 | 1.000 | 7328 | tags=19%, list=23%, signal=24% |
| 1148 | GLUCONEOGENESIS(GO:0006094) |  | 25 | 0.29 | 1.06 | 0.409 | 0.652 | 1.000 | 3987 | tags=20%, list=13%, signal=23% |
| 1149 | CALCIUM\_DEPENDENT\_PROTEIN\_SERINE\_THREONINE\_KINASE\_ACTIVITY(GO:0009931) |  | 30 | 0.31 | 1.05 | 0.421 | 0.651 | 1.000 | 7206 | tags=30%, list=23%, signal=39% |
| 1150 | BICELLULAR\_TIGHT\_JUNCTION\_ASSEMBLY(GO:0070830) |  | 43 | 0.30 | 1.05 | 0.367 | 0.651 | 1.000 | 6249 | tags=26%, list=20%, signal=32% |
| 1151 | APICAL\_JUNCTION\_ASSEMBLY(GO:0043297) |  | 43 | 0.30 | 1.05 | 0.367 | 0.650 | 1.000 | 6249 | tags=26%, list=20%, signal=32% |
| 1152 | SYMPORTER\_ACTIVITY(GO:0015293) |  | 129 | 0.27 | 1.05 | 0.352 | 0.650 | 1.000 | 6372 | tags=22%, list=20%, signal=27% |
| 1153 | CHONDROITIN\_SULFATE\_PROTEOGLYCAN\_BIOSYNTHETIC\_PROCESS(GO:0050650) |  | 20 | 0.34 | 1.05 | 0.346 | 0.651 | 1.000 | 7006 | tags=30%, list=22%, signal=38% |
| 1154 | SECONDARY\_ACTIVE\_TRANSMEMBRANE\_TRANSPORTER\_ACTIVITY(GO:0015291) |  | 217 | 0.26 | 1.05 | 0.395 | 0.652 | 1.000 | 8645 | tags=30%, list=27%, signal=41% |
| 1155 | CHROMATIN\_ASSEMBLY\_OR\_DISASSEMBLY(GO:0006333) |  | 69 | 0.28 | 1.05 | 0.326 | 0.654 | 1.000 | 4012 | tags=12%, list=13%, signal=13% |
| 1156 | INTRINSIC\_COMPONENT\_OF\_ENDOPLASMIC\_RETICULUM\_MEMBRANE(GO:0031227) |  | 110 | 0.24 | 1.05 | 0.319 | 0.654 | 1.000 | 5606 | tags=20%, list=18%, signal=24% |
| 1157 | REGULATION\_OF\_APOPTOTIC\_PROCESS(GO:0042981) |  | 366 | 0.18 | 1.05 | 0.219 | 0.654 | 1.000 | 7470 | tags=23%, list=23%, signal=30% |
| 1158 | CELLULAR\_PROTEIN\_MODIFICATION\_PROCESS(GO:0006464) |  | 2385 | 0.15 | 1.05 | 0.324 | 0.654 | 1.000 | 7143 | tags=18%, list=22%, signal=21% |
| 1159 | PROTEIN\_MODIFICATION\_PROCESS(GO:0036211) |  | 2385 | 0.15 | 1.05 | 0.324 | 0.653 | 1.000 | 7143 | tags=18%, list=22%, signal=21% |
| 1160 | NEGATIVE\_REGULATION\_OF\_CYTOKINE\_PRODUCTION(GO:0001818) |  | 18 | 0.35 | 1.05 | 0.425 | 0.654 | 1.000 | 8046 | tags=33%, list=25%, signal=45% |
| 1161 | ACETYL\_COA\_METABOLIC\_PROCESS(GO:0006084) |  | 19 | 0.35 | 1.05 | 0.447 | 0.656 | 1.000 | 5876 | tags=32%, list=18%, signal=39% |
| 1162 | RESPONSE\_TO\_TOXIC\_SUBSTANCE(GO:0009636) |  | 64 | 0.22 | 1.05 | 0.322 | 0.655 | 1.000 | 10015 | tags=41%, list=31%, signal=59% |
| 1163 | NUCLEAR\_RECEPTOR\_ACTIVITY(GO:0004879) |  | 69 | 0.28 | 1.05 | 0.431 | 0.657 | 1.000 | 7223 | tags=30%, list=23%, signal=39% |
| 1164 | CELL\_JUNCTION\_ASSEMBLY(GO:0034329) |  | 93 | 0.23 | 1.05 | 0.446 | 0.659 | 1.000 | 4945 | tags=15%, list=16%, signal=18% |
| 1165 | MYOSIN\_COMPLEX(GO:0016459) |  | 58 | 0.22 | 1.05 | 0.478 | 0.660 | 1.000 | 5109 | tags=16%, list=16%, signal=18% |
| 1166 | AXONAL\_FASCICULATION(GO:0007413) |  | 16 | 0.36 | 1.05 | 0.458 | 0.660 | 1.000 | 3473 | tags=19%, list=11%, signal=21% |
| 1167 | NEURON\_PROJECTION\_FASCICULATION(GO:0106030) |  | 16 | 0.36 | 1.05 | 0.458 | 0.660 | 1.000 | 3473 | tags=19%, list=11%, signal=21% |
| 1168 | POSITIVE\_REGULATION\_OF\_CELLULAR\_PROCESS(GO:0048522) |  | 1698 | 0.17 | 1.05 | 0.369 | 0.659 | 1.000 | 4244 | tags=11%, list=13%, signal=12% |
| 1169 | DRUG\_TRANSPORT(GO:0015893) |  | 102 | 0.23 | 1.05 | 0.365 | 0.660 | 1.000 | 5171 | tags=16%, list=16%, signal=19% |
| 1170 | EPIDERMAL\_GROWTH\_FACTOR\_RECEPTOR\_SIGNALING\_PATHWAY(GO:0007173) |  | 28 | 0.30 | 1.05 | 0.408 | 0.659 | 1.000 | 6875 | tags=32%, list=22%, signal=41% |
| 1171 | CELL\_SURFACE\_RECEPTOR\_SIGNALING\_PATHWAY(GO:0007166) |  | 1378 | 0.18 | 1.05 | 0.355 | 0.659 | 1.000 | 6520 | tags=18%, list=20%, signal=22% |
| 1172 | POSTTRANSCRIPTIONAL\_GENE\_SILENCING(GO:0016441) |  | 63 | 0.27 | 1.05 | 0.409 | 0.661 | 1.000 | 6084 | tags=21%, list=19%, signal=25% |
| 1173 | POSTTRANSCRIPTIONAL\_GENE\_SILENCING\_BY\_RNA(GO:0035194) |  | 63 | 0.27 | 1.05 | 0.409 | 0.660 | 1.000 | 6084 | tags=21%, list=19%, signal=25% |
| 1174 | NEUTRAL\_LIPID\_BIOSYNTHETIC\_PROCESS(GO:0046460) |  | 18 | 0.32 | 1.05 | 0.374 | 0.660 | 1.000 | 7304 | tags=28%, list=23%, signal=36% |
| 1175 | ACYLGLYCEROL\_BIOSYNTHETIC\_PROCESS(GO:0046463) |  | 18 | 0.32 | 1.05 | 0.374 | 0.659 | 1.000 | 7304 | tags=28%, list=23%, signal=36% |
| 1176 | REGULATION\_OF\_RESPONSE\_TO\_WOUNDING(GO:1903034) |  | 41 | 0.28 | 1.04 | 0.428 | 0.660 | 1.000 | 8636 | tags=39%, list=27%, signal=53% |
| 1177 | RESPONSE\_TO\_DECREASED\_OXYGEN\_LEVELS(GO:0036293) |  | 75 | 0.23 | 1.04 | 0.444 | 0.661 | 1.000 | 4717 | tags=17%, list=15%, signal=20% |
| 1178 | CALCIUM\_ACTIVATED\_CATION\_CHANNEL\_ACTIVITY(GO:0005227) |  | 21 | 0.35 | 1.04 | 0.292 | 0.662 | 1.000 | 10285 | tags=57%, list=32%, signal=84% |
| 1179 | SPINDLE\_MICROTUBULE(GO:0005876) |  | 28 | 0.28 | 1.04 | 0.401 | 0.662 | 1.000 | 223 | tags=4%, list=1%, signal=4% |
| 1180 | CALCIUM\_MEDIATED\_SIGNALING(GO:0019722) |  | 58 | 0.29 | 1.04 | 0.313 | 0.662 | 1.000 | 7190 | tags=29%, list=23%, signal=38% |
| 1181 | GLAND\_DEVELOPMENT(GO:0048732) |  | 246 | 0.19 | 1.04 | 0.354 | 0.664 | 1.000 | 7182 | tags=20%, list=23%, signal=25% |
| 1182 | ORGANONITROGEN\_COMPOUND\_BIOSYNTHETIC\_PROCESS(GO:1901566) |  | 1176 | 0.16 | 1.04 | 0.414 | 0.667 | 1.000 | 5134 | tags=11%, list=16%, signal=13% |
| 1183 | VESICLE(GO:0031982) |  | 841 | 0.16 | 1.04 | 0.279 | 0.668 | 1.000 | 6986 | tags=17%, list=22%, signal=21% |
| 1184 | POSITIVE\_REGULATION\_OF\_T\_CELL\_ACTIVATION(GO:0050870) |  | 32 | 0.31 | 1.04 | 0.412 | 0.668 | 1.000 | 3523 | tags=25%, list=11%, signal=28% |
| 1185 | POSITIVE\_REGULATION\_OF\_LEUKOCYTE\_CELL\_CELL\_ADHESION(GO:1903039) |  | 32 | 0.31 | 1.04 | 0.412 | 0.667 | 1.000 | 3523 | tags=25%, list=11%, signal=28% |
| 1186 | CARGO\_RECEPTOR\_ACTIVITY(GO:0038024) |  | 62 | 0.28 | 1.04 | 0.391 | 0.667 | 1.000 | 5517 | tags=27%, list=17%, signal=33% |
| 1187 | TISSUE\_MIGRATION(GO:0090130) |  | 74 | 0.27 | 1.04 | 0.393 | 0.667 | 1.000 | 6633 | tags=22%, list=21%, signal=27% |
| 1188 | NEURAL\_NUCLEUS\_DEVELOPMENT(GO:0048857) |  | 26 | 0.31 | 1.04 | 0.337 | 0.667 | 1.000 | 184 | tags=4%, list=1%, signal=4% |
| 1189 | EMBRYONIC\_NEUROCRANIUM\_MORPHOGENESIS(GO:0048702) |  | 22 | 0.28 | 1.04 | 0.390 | 0.667 | 1.000 | 8426 | tags=36%, list=26%, signal=49% |
| 1190 | COENZYME\_BIOSYNTHETIC\_PROCESS(GO:0009108) |  | 140 | 0.24 | 1.04 | 0.363 | 0.667 | 1.000 | 5108 | tags=19%, list=16%, signal=23% |
| 1191 | CELLULAR\_RESPONSE\_TO\_ABIOTIC\_STIMULUS(GO:0071214) |  | 67 | 0.26 | 1.04 | 0.394 | 0.668 | 1.000 | 5941 | tags=21%, list=19%, signal=26% |
| 1192 | CELLULAR\_RESPONSE\_TO\_ENVIRONMENTAL\_STIMULUS(GO:0104004) |  | 67 | 0.26 | 1.04 | 0.394 | 0.667 | 1.000 | 5941 | tags=21%, list=19%, signal=26% |
| 1193 | DNA\_DIRECTED\_DNA\_POLYMERASE\_ACTIVITY(GO:0003887) |  | 17 | 0.27 | 1.04 | 0.282 | 0.667 | 1.000 | 5630 | tags=24%, list=18%, signal=29% |
| 1194 | GERM\_CELL\_DEVELOPMENT(GO:0007281) |  | 84 | 0.26 | 1.04 | 0.470 | 0.667 | 1.000 | 9804 | tags=35%, list=31%, signal=50% |
| 1195 | SPINDLE(GO:0005819) |  | 142 | 0.18 | 1.04 | 0.400 | 0.668 | 1.000 | 7973 | tags=21%, list=25%, signal=28% |
| 1196 | MITOTIC\_DNA\_INTEGRITY\_CHECKPOINT(GO:0044774) |  | 26 | 0.31 | 1.04 | 0.439 | 0.669 | 1.000 | 1006 | tags=8%, list=3%, signal=8% |
| 1197 | INTRACELLULAR\_RECEPTOR\_SIGNALING\_PATHWAY(GO:0030522) |  | 57 | 0.25 | 1.04 | 0.355 | 0.669 | 1.000 | 4501 | tags=18%, list=14%, signal=20% |
| 1198 | BETA\_GLUCOSIDASE\_ACTIVITY(GO:0008422) |  | 20 | 0.36 | 1.04 | 0.413 | 0.672 | 1.000 | 2333 | tags=10%, list=7%, signal=11% |
| 1199 | POSITIVE\_REGULATION\_OF\_CELL\_MOTILITY(GO:2000147) |  | 98 | 0.24 | 1.03 | 0.409 | 0.672 | 1.000 | 2608 | tags=8%, list=8%, signal=9% |
| 1200 | NEGATIVE\_REGULATION\_OF\_AXON\_EXTENSION(GO:0030517) |  | 38 | 0.33 | 1.03 | 0.524 | 0.672 | 1.000 | 2608 | tags=11%, list=8%, signal=11% |
| 1201 | POSITIVE\_REGULATION\_OF\_LEUKOCYTE\_ACTIVATION(GO:0002696) |  | 44 | 0.28 | 1.03 | 0.400 | 0.672 | 1.000 | 3950 | tags=20%, list=12%, signal=23% |
| 1202 | POSITIVE\_REGULATION\_OF\_BIOLOGICAL\_PROCESS(GO:0048518) |  | 2005 | 0.17 | 1.03 | 0.370 | 0.673 | 1.000 | 7230 | tags=20%, list=23%, signal=24% |
| 1203 | REGULATION\_OF\_IMMUNE\_SYSTEM\_PROCESS(GO:0002682) |  | 344 | 0.21 | 1.03 | 0.313 | 0.674 | 1.000 | 7206 | tags=26%, list=23%, signal=33% |
| 1204 | TRANSCRIPTION\_FACTOR\_ACTIVITY\_\_DIRECT\_LIGAND\_REGULATED\_SEQUENCE\_SPECIFIC\_DNA\_BINDING(GO:0098531) |  | 70 | 0.27 | 1.03 | 0.432 | 0.674 | 1.000 | 7223 | tags=30%, list=23%, signal=39% |
| 1205 | REGULATION\_OF\_CELL\_CYCLE\_PROCESS(GO:0010564) |  | 175 | 0.19 | 1.03 | 0.421 | 0.674 | 1.000 | 7434 | tags=24%, list=23%, signal=31% |
| 1206 | N\_ACYLTRANSFERASE\_ACTIVITY(GO:0016410) |  | 97 | 0.20 | 1.03 | 0.355 | 0.674 | 1.000 | 3790 | tags=11%, list=12%, signal=13% |
| 1207 | REGULATION\_OF\_GENERATION\_OF\_PRECURSOR\_METABOLITES\_AND\_ENERGY(GO:0043467) |  | 29 | 0.31 | 1.03 | 0.357 | 0.675 | 1.000 | 4470 | tags=28%, list=14%, signal=32% |
| 1208 | POSITIVE\_REGULATION\_OF\_NEURON\_DIFFERENTIATION(GO:0045666) |  | 77 | 0.23 | 1.03 | 0.414 | 0.674 | 1.000 | 3491 | tags=10%, list=11%, signal=12% |
| 1209 | ADENYLATE\_CYCLASE\_ACTIVATING\_G\_PROTEIN\_COUPLED\_RECEPTOR\_SIGNALING\_PATHWAY(GO:0007189) |  | 109 | 0.27 | 1.03 | 0.354 | 0.676 | 1.000 | 5401 | tags=20%, list=17%, signal=24% |
| 1210 | RESPONSE\_TO\_DRUG(GO:0042493) |  | 178 | 0.21 | 1.03 | 0.411 | 0.676 | 1.000 | 5171 | tags=16%, list=16%, signal=19% |
| 1211 | RETINAL\_GANGLION\_CELL\_AXON\_GUIDANCE(GO:0031290) |  | 44 | 0.23 | 1.03 | 0.390 | 0.677 | 1.000 | 184 | tags=2%, list=1%, signal=2% |
| 1212 | GOLGI\_TO\_PLASMA\_MEMBRANE\_TRANSPORT(GO:0006893) |  | 24 | 0.25 | 1.03 | 0.416 | 0.678 | 1.000 | 3579 | tags=17%, list=11%, signal=19% |
| 1213 | TRICARBOXYLIC\_ACID\_METABOLIC\_PROCESS(GO:0072350) |  | 32 | 0.27 | 1.03 | 0.435 | 0.678 | 1.000 | 3693 | tags=16%, list=12%, signal=18% |
| 1214 | TRANSCRIPTIONAL\_REPRESSOR\_COMPLEX(GO:0017053) |  | 21 | 0.29 | 1.03 | 0.378 | 0.679 | 1.000 | 9801 | tags=48%, list=31%, signal=69% |
| 1215 | POSITIVE\_REGULATION\_OF\_CELL\_ACTIVATION(GO:0050867) |  | 45 | 0.28 | 1.03 | 0.428 | 0.678 | 1.000 | 3950 | tags=20%, list=12%, signal=23% |
| 1216 | RESPIRATORY\_CHAIN\_COMPLEX(GO:0098803) |  | 67 | 0.27 | 1.03 | 0.363 | 0.678 | 1.000 | 1769 | tags=4%, list=6%, signal=5% |
| 1217 | CELL\_DIVISION(GO:0051301) |  | 278 | 0.16 | 1.03 | 0.374 | 0.678 | 1.000 | 6937 | tags=16%, list=22%, signal=21% |
| 1218 | NUCLEOTIDE\_EXCISION\_REPAIR(GO:0006289) |  | 32 | 0.28 | 1.03 | 0.378 | 0.679 | 1.000 | 7211 | tags=25%, list=23%, signal=32% |
| 1219 | POSITIVE\_REGULATION\_OF\_CELL\_GROWTH(GO:0030307) |  | 22 | 0.28 | 1.03 | 0.312 | 0.678 | 1.000 | 8709 | tags=32%, list=27%, signal=44% |
| 1220 | CELLULAR\_HORMONE\_METABOLIC\_PROCESS(GO:0034754) |  | 39 | 0.32 | 1.03 | 0.354 | 0.678 | 1.000 | 3032 | tags=18%, list=10%, signal=20% |
| 1221 | PHOSPHOLIPID\_DEPHOSPHORYLATION(GO:0046839) |  | 46 | 0.24 | 1.03 | 0.337 | 0.678 | 1.000 | 4918 | tags=15%, list=15%, signal=18% |
| 1222 | CITRATE\_METABOLIC\_PROCESS(GO:0006101) |  | 31 | 0.27 | 1.03 | 0.334 | 0.678 | 1.000 | 3693 | tags=16%, list=12%, signal=18% |
| 1223 | RECEPTOR\_REGULATOR\_ACTIVITY(GO:0030545) |  | 368 | 0.21 | 1.03 | 0.381 | 0.678 | 1.000 | 4503 | tags=14%, list=14%, signal=16% |
| 1224 | POSITIVE\_REGULATION\_OF\_CELL\_DIVISION(GO:0051781) |  | 25 | 0.30 | 1.03 | 0.486 | 0.678 | 1.000 | 2476 | tags=12%, list=8%, signal=13% |
| 1225 | SECOND\_MESSENGER\_MEDIATED\_SIGNALING(GO:0019932) |  | 190 | 0.24 | 1.03 | 0.388 | 0.681 | 1.000 | 4985 | tags=17%, list=16%, signal=20% |
| 1226 | NEGATIVE\_REGULATION\_OF\_APOPTOTIC\_PROCESS(GO:0043066) |  | 171 | 0.21 | 1.02 | 0.387 | 0.682 | 1.000 | 4734 | tags=15%, list=15%, signal=18% |
| 1227 | CERAMIDE\_CATABOLIC\_PROCESS(GO:0046514) |  | 17 | 0.33 | 1.02 | 0.364 | 0.682 | 1.000 | 3533 | tags=24%, list=11%, signal=26% |
| 1228 | EPITHELIAL\_TUBE\_MORPHOGENESIS(GO:0060562) |  | 242 | 0.20 | 1.02 | 0.404 | 0.682 | 1.000 | 3698 | tags=10%, list=12%, signal=11% |
| 1229 | KINASE\_ACTIVITY(GO:0016301) |  | 882 | 0.16 | 1.02 | 0.417 | 0.682 | 1.000 | 7418 | tags=20%, list=23%, signal=25% |
| 1230 | RECEPTOR\_GUANYLYL\_CYCLASE\_SIGNALING\_PATHWAY(GO:0007168) |  | 16 | 0.43 | 1.02 | 0.436 | 0.681 | 1.000 | 1232 | tags=13%, list=4%, signal=13% |
| 1231 | NEGATIVE\_REGULATION\_OF\_CELL\_MORPHOGENESIS\_INVOLVED\_IN\_DIFFERENTIATION(GO:0010771) |  | 41 | 0.32 | 1.02 | 0.456 | 0.682 | 1.000 | 2608 | tags=10%, list=8%, signal=11% |
| 1232 | GLIAL\_CELL\_DEVELOPMENT(GO:0021782) |  | 97 | 0.23 | 1.02 | 0.384 | 0.682 | 1.000 | 8021 | tags=27%, list=25%, signal=36% |
| 1233 | HEXOSE\_BIOSYNTHETIC\_PROCESS(GO:0019319) |  | 26 | 0.28 | 1.02 | 0.422 | 0.682 | 1.000 | 5125 | tags=23%, list=16%, signal=27% |
| 1234 | PROTEIN\_KINASE\_ACTIVATOR\_ACTIVITY(GO:0030295) |  | 44 | 0.26 | 1.02 | 0.395 | 0.682 | 1.000 | 10725 | tags=41%, list=34%, signal=62% |
| 1235 | KINASE\_ACTIVATOR\_ACTIVITY(GO:0019209) |  | 44 | 0.26 | 1.02 | 0.395 | 0.682 | 1.000 | 10725 | tags=41%, list=34%, signal=62% |
| 1236 | UNSATURATED\_FATTY\_ACID\_METABOLIC\_PROCESS(GO:0033559) |  | 36 | 0.34 | 1.02 | 0.445 | 0.683 | 1.000 | 4729 | tags=28%, list=15%, signal=33% |
| 1237 | ODONTOGENESIS(GO:0042476) |  | 22 | 0.29 | 1.02 | 0.456 | 0.684 | 1.000 | 10146 | tags=36%, list=32%, signal=53% |
| 1238 | SYNAPSE\_ASSEMBLY(GO:0007416) |  | 61 | 0.29 | 1.02 | 0.472 | 0.685 | 1.000 | 1043 | tags=7%, list=3%, signal=7% |
| 1239 | REGULATION\_OF\_PH(GO:0006885) |  | 42 | 0.25 | 1.02 | 0.381 | 0.689 | 1.000 | 10367 | tags=40%, list=33%, signal=60% |
| 1240 | BRANCHING\_INVOLVED\_IN\_BLOOD\_VESSEL\_MORPHOGENESIS(GO:0001569) |  | 16 | 0.32 | 1.02 | 0.392 | 0.689 | 1.000 | 233 | tags=6%, list=1%, signal=6% |
| 1241 | NEGATIVE\_REGULATION\_OF\_MULTICELLULAR\_ORGANISMAL\_PROCESS(GO:0051241) |  | 210 | 0.22 | 1.02 | 0.436 | 0.691 | 1.000 | 8046 | tags=22%, list=25%, signal=30% |
| 1242 | EPIBOLY\_INVOLVED\_IN\_GASTRULATION\_WITH\_MOUTH\_FORMING\_SECOND(GO:0055113) |  | 50 | 0.27 | 1.02 | 0.324 | 0.690 | 1.000 | 3251 | tags=8%, list=10%, signal=9% |
| 1243 | U2\_SNRNP(GO:0005686) |  | 17 | 0.30 | 1.02 | 0.330 | 0.692 | 1.000 | 22260 | tags=100%, list=70%, signal=331% |
| 1244 | CELL\_MIGRATION\_INVOLVED\_IN\_SPROUTING\_ANGIOGENESIS(GO:0002042) |  | 19 | 0.34 | 1.02 | 0.426 | 0.692 | 1.000 | 184 | tags=5%, list=1%, signal=5% |
| 1245 | NEGATIVE\_REGULATION\_OF\_PROGRAMMED\_CELL\_DEATH(GO:0043069) |  | 176 | 0.20 | 1.02 | 0.423 | 0.693 | 1.000 | 9399 | tags=32%, list=29%, signal=46% |
| 1246 | EPITHELIAL\_CELL\_MIGRATION(GO:0010631) |  | 63 | 0.27 | 1.02 | 0.388 | 0.693 | 1.000 | 7020 | tags=22%, list=22%, signal=28% |
| 1247 | THROMBOCYTE\_DIFFERENTIATION(GO:0002574) |  | 16 | 0.35 | 1.02 | 0.403 | 0.692 | 1.000 | 4284 | tags=31%, list=13%, signal=36% |
| 1248 | RESPONSE\_TO\_HYPOXIA(GO:0001666) |  | 74 | 0.22 | 1.01 | 0.406 | 0.692 | 1.000 | 4717 | tags=16%, list=15%, signal=19% |
| 1249 | PHOSPHATIDYLINOSITOL\_PHOSPHATE\_PHOSPHATASE\_ACTIVITY(GO:0052866) |  | 32 | 0.28 | 1.01 | 0.458 | 0.691 | 1.000 | 4918 | tags=16%, list=15%, signal=18% |
| 1250 | RESPONSE\_TO\_OXYGEN\_LEVELS(GO:0070482) |  | 76 | 0.22 | 1.01 | 0.483 | 0.691 | 1.000 | 4717 | tags=17%, list=15%, signal=20% |
| 1251 | DRUG\_TRANSMEMBRANE\_TRANSPORT(GO:0006855) |  | 31 | 0.28 | 1.01 | 0.444 | 0.692 | 1.000 | 1236 | tags=6%, list=4%, signal=7% |
| 1252 | VERY\_LONG\_CHAIN\_FATTY\_ACID\_BIOSYNTHETIC\_PROCESS(GO:0042761) |  | 20 | 0.38 | 1.01 | 0.514 | 0.692 | 1.000 | 4729 | tags=30%, list=15%, signal=35% |
| 1253 | VITAMIN\_BINDING(GO:0019842) |  | 111 | 0.22 | 1.01 | 0.355 | 0.692 | 1.000 | 6427 | tags=21%, list=20%, signal=26% |
| 1254 | TRANSCRIPTION\_FACTOR\_COMPLEX(GO:0005667) |  | 218 | 0.22 | 1.01 | 0.386 | 0.694 | 1.000 | 7293 | tags=23%, list=23%, signal=30% |
| 1255 | REGULATION\_OF\_NON\_CANONICAL\_WNT\_SIGNALING\_PATHWAY(GO:2000050) |  | 16 | 0.31 | 1.01 | 0.526 | 0.694 | 1.000 | 7738 | tags=31%, list=24%, signal=41% |
| 1256 | RESPIRATORY\_CHAIN\_COMPLEX\_IV(GO:0045277) |  | 19 | 0.33 | 1.01 | 0.360 | 0.693 | 1.000 | 746 | tags=5%, list=2%, signal=5% |
| 1257 | MESODERM\_MORPHOGENESIS(GO:0048332) |  | 51 | 0.26 | 1.01 | 0.382 | 0.693 | 1.000 | 10895 | tags=39%, list=34%, signal=59% |
| 1258 | CIRCADIAN\_BEHAVIOR(GO:0048512) |  | 17 | 0.32 | 1.01 | 0.374 | 0.694 | 1.000 | 3624 | tags=18%, list=11%, signal=20% |
| 1259 | RHYTHMIC\_BEHAVIOR(GO:0007622) |  | 17 | 0.32 | 1.01 | 0.374 | 0.694 | 1.000 | 3624 | tags=18%, list=11%, signal=20% |
| 1260 | PHOSPHATIDYLINOSITOL\_DEPHOSPHORYLATION(GO:0046856) |  | 30 | 0.28 | 1.01 | 0.413 | 0.695 | 1.000 | 4918 | tags=17%, list=15%, signal=20% |
| 1261 | CYCLIC\_NUCLEOTIDE\_METABOLIC\_PROCESS(GO:0009187) |  | 56 | 0.27 | 1.01 | 0.459 | 0.694 | 1.000 | 10076 | tags=34%, list=32%, signal=50% |
| 1262 | T\_CELL\_RECEPTOR\_SIGNALING\_PATHWAY(GO:0050852) |  | 29 | 0.32 | 1.01 | 0.351 | 0.694 | 1.000 | 5903 | tags=38%, list=19%, signal=47% |
| 1263 | MRNA\_CIS\_SPLICING\_\_VIA\_SPLICEOSOME(GO:0045292) |  | 15 | 0.28 | 1.01 | 0.414 | 0.694 | 1.000 | 22871 | tags=100%, list=72%, signal=353% |
| 1264 | ACTOMYOSIN(GO:0042641) |  | 33 | 0.28 | 1.01 | 0.448 | 0.694 | 1.000 | 7303 | tags=21%, list=23%, signal=27% |
| 1265 | ENGULFMENT\_OF\_APOPTOTIC\_CELL(GO:0043652) |  | 24 | 0.32 | 1.01 | 0.414 | 0.695 | 1.000 | 5211 | tags=21%, list=16%, signal=25% |
| 1266 | ENDONUCLEASE\_ACTIVITY\_\_ACTIVE\_WITH\_EITHER\_RIBO\_\_OR\_DEOXYRIBONUCLEIC\_ACIDS\_AND\_PRODUCING\_5'\_PHOSPHOMONOESTERS(GO:0016893) |  | 39 | 0.27 | 1.01 | 0.481 | 0.696 | 1.000 | 3639 | tags=10%, list=11%, signal=12% |
| 1267 | TRANSPORT\_VESICLE(GO:0030133) |  | 193 | 0.20 | 1.01 | 0.355 | 0.695 | 1.000 | 6311 | tags=17%, list=20%, signal=21% |
| 1268 | NEGATIVE\_REGULATION\_OF\_AXONOGENESIS(GO:0050771) |  | 40 | 0.33 | 1.01 | 0.457 | 0.696 | 1.000 | 2608 | tags=10%, list=8%, signal=11% |
| 1269 | RECEPTOR\_TYROSINE\_KINASE\_BINDING(GO:0030971) |  | 28 | 0.32 | 1.01 | 0.521 | 0.696 | 1.000 | 10275 | tags=39%, list=32%, signal=58% |
| 1270 | HYDROLASE\_ACTIVITY\_\_ACTING\_ON\_CARBON\_NITROGEN\_(BUT\_NOT\_PEPTIDE)\_BONDS(GO:0016810) |  | 108 | 0.20 | 1.01 | 0.473 | 0.696 | 1.000 | 5009 | tags=17%, list=16%, signal=20% |
| 1271 | FLAVIN\_ADENINE\_DINUCLEOTIDE\_BINDING(GO:0050660) |  | 82 | 0.19 | 1.01 | 0.425 | 0.697 | 1.000 | 6798 | tags=22%, list=21%, signal=28% |
| 1272 | CIRCADIAN\_REGULATION\_OF\_GENE\_EXPRESSION(GO:0032922) |  | 41 | 0.27 | 1.01 | 0.448 | 0.696 | 1.000 | 10203 | tags=41%, list=32%, signal=61% |
| 1273 | NEGATIVE\_REGULATION\_OF\_SIGNAL\_TRANSDUCTION(GO:0009968) |  | 411 | 0.18 | 1.01 | 0.380 | 0.696 | 1.000 | 4871 | tags=14%, list=15%, signal=16% |
| 1274 | REGULATION\_OF\_ERK1\_AND\_ERK2\_CASCADE(GO:0070372) |  | 71 | 0.28 | 1.01 | 0.505 | 0.699 | 1.000 | 5055 | tags=21%, list=16%, signal=25% |
| 1275 | POSITIVE\_REGULATION\_OF\_CELL\_PROJECTION\_ORGANIZATION(GO:0031346) |  | 84 | 0.24 | 1.01 | 0.375 | 0.699 | 1.000 | 3491 | tags=12%, list=11%, signal=13% |
| 1276 | COLLAGEN\_TRIMER(GO:0005581) |  | 56 | 0.33 | 1.01 | 0.444 | 0.698 | 1.000 | 7232 | tags=29%, list=23%, signal=37% |
| 1277 | PEPTIDYL\_L\_CYSTEINE\_S\_PALMITOYLATION(GO:0018230) |  | 26 | 0.29 | 1.01 | 0.388 | 0.698 | 1.000 | 8160 | tags=38%, list=26%, signal=52% |
| 1278 | PEPTIDYL\_S\_DIACYLGLYCEROL\_L\_CYSTEINE\_BIOSYNTHETIC\_PROCESS\_FROM\_PEPTIDYL\_CYSTEINE(GO:0018231) |  | 26 | 0.29 | 1.01 | 0.388 | 0.697 | 1.000 | 8160 | tags=38%, list=26%, signal=52% |
| 1279 | LIPOPROTEIN\_CATABOLIC\_PROCESS(GO:0042159) |  | 15 | 0.27 | 1.01 | 0.537 | 0.697 | 1.000 | 9963 | tags=47%, list=31%, signal=68% |
| 1280 | REGULATION\_OF\_DNA\_RECOMBINATION(GO:0000018) |  | 39 | 0.26 | 1.01 | 0.579 | 0.697 | 1.000 | 12436 | tags=46%, list=39%, signal=76% |
| 1281 | AXIS\_SPECIFICATION(GO:0009798) |  | 64 | 0.26 | 1.01 | 0.434 | 0.697 | 1.000 | 7279 | tags=20%, list=23%, signal=26% |
| 1282 | GLIOGENESIS(GO:0042063) |  | 147 | 0.20 | 1.00 | 0.419 | 0.697 | 1.000 | 8146 | tags=26%, list=26%, signal=35% |
| 1283 | REGULATION\_OF\_HEART\_CONTRACTION(GO:0008016) |  | 68 | 0.23 | 1.00 | 0.456 | 0.696 | 1.000 | 6618 | tags=25%, list=21%, signal=31% |
| 1284 | CONNECTIVE\_TISSUE\_DEVELOPMENT(GO:0061448) |  | 161 | 0.18 | 1.00 | 0.456 | 0.697 | 1.000 | 5083 | tags=12%, list=16%, signal=15% |
| 1285 | CELL\_DIFFERENTIATION\_IN\_SPINAL\_CORD(GO:0021515) |  | 56 | 0.28 | 1.00 | 0.351 | 0.698 | 1.000 | 8146 | tags=29%, list=26%, signal=38% |
| 1286 | POSTERIOR\_LATERAL\_LINE\_NEUROMAST\_PRIMORDIUM\_MIGRATION(GO:0048920) |  | 25 | 0.24 | 1.00 | 0.429 | 0.697 | 1.000 | 439 | tags=4%, list=1%, signal=4% |
| 1287 | MOTOR\_ACTIVITY(GO:0003774) |  | 130 | 0.18 | 1.00 | 0.480 | 0.697 | 1.000 | 5673 | tags=13%, list=18%, signal=16% |
| 1288 | LIVER\_DEVELOPMENT(GO:0001889) |  | 147 | 0.19 | 1.00 | 0.375 | 0.697 | 1.000 | 7182 | tags=21%, list=23%, signal=27% |
| 1289 | ANTIPORTER\_ACTIVITY(GO:0015297) |  | 70 | 0.26 | 1.00 | 0.492 | 0.697 | 1.000 | 6987 | tags=29%, list=22%, signal=37% |
| 1290 | CELL\_RECOGNITION(GO:0008037) |  | 56 | 0.24 | 1.00 | 0.406 | 0.698 | 1.000 | 5747 | tags=18%, list=18%, signal=22% |
| 1291 | DNA\_HELICASE\_ACTIVITY(GO:0003678) |  | 54 | 0.22 | 1.00 | 0.405 | 0.701 | 1.000 | 3235 | tags=9%, list=10%, signal=10% |
| 1292 | RETINOIC\_ACID\_RECEPTOR\_SIGNALING\_PATHWAY(GO:0048384) |  | 32 | 0.27 | 1.00 | 0.460 | 0.701 | 1.000 | 3876 | tags=16%, list=12%, signal=18% |
| 1293 | EPITHELIAL\_CELL\_DEVELOPMENT(GO:0002064) |  | 115 | 0.20 | 1.00 | 0.438 | 0.701 | 1.000 | 7125 | tags=21%, list=22%, signal=27% |
| 1294 | AP\_TYPE\_MEMBRANE\_COAT\_ADAPTOR\_COMPLEX(GO:0030119) |  | 26 | 0.25 | 1.00 | 0.378 | 0.703 | 1.000 | 5189 | tags=19%, list=16%, signal=23% |
| 1295 | MORPHOGENESIS\_OF\_AN\_EPITHELIAL\_SHEET(GO:0002011) |  | 94 | 0.20 | 1.00 | 0.392 | 0.707 | 1.000 | 3364 | tags=7%, list=11%, signal=8% |
| 1296 | MICROTUBULE\_PLUS\_END(GO:0035371) |  | 16 | 0.26 | 1.00 | 0.425 | 0.708 | 1.000 | 8022 | tags=31%, list=25%, signal=42% |
| 1297 | POSITIVE\_REGULATION\_OF\_NF\_KAPPAB\_TRANSCRIPTION\_FACTOR\_ACTIVITY(GO:0051092) |  | 22 | 0.30 | 1.00 | 0.396 | 0.707 | 1.000 | 6100 | tags=18%, list=19%, signal=22% |
| 1298 | ACTIVE\_TRANSMEMBRANE\_TRANSPORTER\_ACTIVITY(GO:0022804) |  | 311 | 0.22 | 1.00 | 0.427 | 0.707 | 1.000 | 7060 | tags=23%, list=22%, signal=29% |
| 1299 | BLOOD\_VESSEL\_ENDOTHELIAL\_CELL\_MIGRATION(GO:0043534) |  | 36 | 0.28 | 1.00 | 0.434 | 0.710 | 1.000 | 7020 | tags=25%, list=22%, signal=32% |
| 1300 | NUCLEAR\_TRANSCRIBED\_MRNA\_CATABOLIC\_PROCESS\_\_NONSENSE\_MEDIATED\_DECAY(GO:0000184) |  | 25 | 0.25 | 0.99 | 0.354 | 0.711 | 1.000 | 4356 | tags=12%, list=14%, signal=14% |
| 1301 | DNA\_MODIFICATION(GO:0006304) |  | 36 | 0.28 | 0.99 | 0.498 | 0.712 | 1.000 | 7555 | tags=33%, list=24%, signal=44% |
| 1302 | SODIUM\_ION\_TRANSMEMBRANE\_TRANSPORTER\_ACTIVITY(GO:0015081) |  | 117 | 0.27 | 0.99 | 0.407 | 0.712 | 1.000 | 9381 | tags=34%, list=29%, signal=48% |
| 1303 | TRANSMEMBRANE\_RECEPTOR\_PROTEIN\_KINASE\_ACTIVITY(GO:0019199) |  | 93 | 0.26 | 0.99 | 0.455 | 0.712 | 1.000 | 7642 | tags=23%, list=24%, signal=30% |
| 1304 | REGULATION\_OF\_MACROMOLECULE\_METABOLIC\_PROCESS(GO:0060255) |  | 3362 | 0.16 | 0.99 | 0.432 | 0.713 | 1.000 | 7127 | tags=18%, list=22%, signal=21% |
| 1305 | FATTY\_ACID\_SYNTHASE\_ACTIVITY(GO:0004312) |  | 19 | 0.40 | 0.99 | 0.502 | 0.712 | 1.000 | 5158 | tags=32%, list=16%, signal=38% |
| 1306 | PHOSPHOTRANSFERASE\_ACTIVITY\_\_FOR\_OTHER\_SUBSTITUTED\_PHOSPHATE\_GROUPS(GO:0016780) |  | 20 | 0.30 | 0.99 | 0.477 | 0.712 | 1.000 | 6479 | tags=20%, list=20%, signal=25% |
| 1307 | VASCULAR\_ENDOTHELIAL\_GROWTH\_FACTOR\_SIGNALING\_PATHWAY(GO:0038084) |  | 19 | 0.33 | 0.99 | 0.439 | 0.712 | 1.000 | 11941 | tags=47%, list=37%, signal=76% |
| 1308 | CAMP\_MEDIATED\_SIGNALING(GO:0019933) |  | 121 | 0.25 | 0.99 | 0.382 | 0.711 | 1.000 | 5401 | tags=19%, list=17%, signal=23% |
| 1309 | POSITIVE\_REGULATION\_OF\_LOCOMOTION(GO:0040017) |  | 102 | 0.23 | 0.99 | 0.448 | 0.711 | 1.000 | 2608 | tags=8%, list=8%, signal=9% |
| 1310 | REGULATION\_OF\_MAP\_KINASE\_ACTIVITY(GO:0043405) |  | 31 | 0.29 | 0.99 | 0.432 | 0.711 | 1.000 | 3482 | tags=19%, list=11%, signal=22% |
| 1311 | SPLICEOSOMAL\_COMPLEX\_ASSEMBLY(GO:0000245) |  | 34 | 0.23 | 0.99 | 0.535 | 0.711 | 1.000 | 24639 | tags=100%, list=77%, signal=439% |
| 1312 | ERK1\_AND\_ERK2\_CASCADE(GO:0070371) |  | 73 | 0.27 | 0.99 | 0.518 | 0.711 | 1.000 | 5055 | tags=21%, list=16%, signal=24% |
| 1313 | ACYL\_COA\_HYDROLASE\_ACTIVITY(GO:0047617) |  | 15 | 0.35 | 0.99 | 0.422 | 0.711 | 1.000 | 5292 | tags=33%, list=17%, signal=40% |
| 1314 | FAD\_BINDING(GO:0071949) |  | 38 | 0.26 | 0.99 | 0.529 | 0.712 | 1.000 | 10190 | tags=45%, list=32%, signal=66% |
| 1315 | CARBOHYDRATE\_METABOLIC\_PROCESS(GO:0005975) |  | 353 | 0.19 | 0.99 | 0.419 | 0.712 | 1.000 | 7047 | tags=22%, list=22%, signal=28% |
| 1316 | IRON\_ION\_BINDING(GO:0005506) |  | 169 | 0.23 | 0.99 | 0.354 | 0.714 | 1.000 | 7421 | tags=32%, list=23%, signal=41% |
| 1317 | PROTEIN\_CHROMOPHORE\_LINKAGE(GO:0018298) |  | 43 | 0.31 | 0.99 | 0.487 | 0.714 | 1.000 | 5274 | tags=21%, list=17%, signal=25% |
| 1318 | PEPTIDE\_RECEPTOR\_ACTIVITY(GO:0001653) |  | 153 | 0.26 | 0.99 | 0.449 | 0.714 | 1.000 | 5561 | tags=23%, list=17%, signal=28% |
| 1319 | THYMUS\_DEVELOPMENT(GO:0048538) |  | 21 | 0.30 | 0.99 | 0.385 | 0.714 | 1.000 | 8419 | tags=29%, list=26%, signal=39% |
| 1320 | GOLGI\_APPARATUS(GO:0005794) |  | 700 | 0.16 | 0.99 | 0.438 | 0.714 | 1.000 | 5217 | tags=12%, list=16%, signal=15% |
| 1321 | CELLULAR\_RESPONSE\_TO\_INTERFERON\_GAMMA(GO:0071346) |  | 28 | 0.32 | 0.99 | 0.462 | 0.714 | 1.000 | 5055 | tags=29%, list=16%, signal=34% |
| 1322 | REGULATION\_OF\_METABOLIC\_PROCESS(GO:0019222) |  | 3620 | 0.16 | 0.99 | 0.432 | 0.715 | 1.000 | 7127 | tags=18%, list=22%, signal=21% |
| 1323 | SEMICIRCULAR\_CANAL\_MORPHOGENESIS(GO:0048752) |  | 18 | 0.30 | 0.99 | 0.405 | 0.714 | 1.000 | 1196 | tags=6%, list=4%, signal=6% |
| 1324 | REGULATION\_OF\_CELLULAR\_METABOLIC\_PROCESS(GO:0031323) |  | 3403 | 0.16 | 0.99 | 0.420 | 0.714 | 1.000 | 7127 | tags=18%, list=22%, signal=21% |
| 1325 | POSITIVE\_REGULATION\_OF\_NEURON\_PROJECTION\_DEVELOPMENT(GO:0010976) |  | 58 | 0.23 | 0.99 | 0.510 | 0.714 | 1.000 | 3491 | tags=10%, list=11%, signal=12% |
| 1326 | MITOTIC\_CELL\_CYCLE\_CHECKPOINT(GO:0007093) |  | 51 | 0.25 | 0.99 | 0.283 | 0.715 | 1.000 | 1020 | tags=6%, list=3%, signal=6% |
| 1327 | REGULATION\_OF\_MITOTIC\_CELL\_CYCLE(GO:0007346) |  | 201 | 0.20 | 0.99 | 0.474 | 0.715 | 1.000 | 7434 | tags=22%, list=23%, signal=29% |
| 1328 | TRANSMEMBRANE\_RECEPTOR\_PROTEIN\_TYROSINE\_KINASE\_SIGNALING\_PATHWAY(GO:0007169) |  | 313 | 0.20 | 0.99 | 0.479 | 0.718 | 1.000 | 7642 | tags=24%, list=24%, signal=32% |
| 1329 | CLATHRIN\_COATED\_VESICLE\_MEMBRANE(GO:0030665) |  | 29 | 0.24 | 0.99 | 0.458 | 0.718 | 1.000 | 8709 | tags=31%, list=27%, signal=43% |
| 1330 | RESPONSE\_TO\_INTERFERON\_GAMMA(GO:0034341) |  | 29 | 0.31 | 0.99 | 0.469 | 0.718 | 1.000 | 5055 | tags=28%, list=16%, signal=33% |
| 1331 | SPHINGOLIPID\_CATABOLIC\_PROCESS(GO:0030149) |  | 25 | 0.30 | 0.99 | 0.439 | 0.718 | 1.000 | 5110 | tags=24%, list=16%, signal=29% |
| 1332 | MEMBRANE\_LIPID\_CATABOLIC\_PROCESS(GO:0046466) |  | 25 | 0.30 | 0.99 | 0.439 | 0.717 | 1.000 | 5110 | tags=24%, list=16%, signal=29% |
| 1333 | TRIGLYCERIDE\_METABOLIC\_PROCESS(GO:0006641) |  | 36 | 0.25 | 0.99 | 0.453 | 0.717 | 1.000 | 8835 | tags=31%, list=28%, signal=42% |
| 1334 | PATTERN\_SPECIFICATION\_PROCESS(GO:0007389) |  | 588 | 0.16 | 0.99 | 0.427 | 0.717 | 1.000 | 7470 | tags=19%, list=23%, signal=25% |
| 1335 | PHOSPHATIDYLINOSITOL\_BISPHOSPHATE\_PHOSPHATASE\_ACTIVITY(GO:0034593) |  | 22 | 0.33 | 0.98 | 0.517 | 0.719 | 1.000 | 4918 | tags=18%, list=15%, signal=21% |
| 1336 | DOUBLE\_STRANDED\_RNA\_BINDING(GO:0003725) |  | 30 | 0.27 | 0.98 | 0.451 | 0.719 | 1.000 | 5426 | tags=23%, list=17%, signal=28% |
| 1337 | ALTERNATIVE\_MRNA\_SPLICING\_\_VIA\_SPLICEOSOME(GO:0000380) |  | 62 | 0.20 | 0.98 | 0.418 | 0.721 | 1.000 | 7521 | tags=19%, list=24%, signal=25% |
| 1338 | EXIT\_FROM\_MITOSIS(GO:0010458) |  | 16 | 0.29 | 0.98 | 0.572 | 0.721 | 1.000 | 7143 | tags=25%, list=22%, signal=32% |
| 1339 | RESPONSE\_TO\_INTERLEUKIN\_1(GO:0070555) |  | 31 | 0.30 | 0.98 | 0.450 | 0.722 | 1.000 | 2466 | tags=16%, list=8%, signal=17% |
| 1340 | PHAGOCYTOSIS\_\_ENGULFMENT(GO:0006911) |  | 29 | 0.30 | 0.98 | 0.464 | 0.722 | 1.000 | 5211 | tags=21%, list=16%, signal=25% |
| 1341 | RIBOSOMAL\_LARGE\_SUBUNIT\_BIOGENESIS(GO:0042273) |  | 59 | 0.26 | 0.98 | 0.455 | 0.723 | 1.000 | 715 | tags=3%, list=2%, signal=3% |
| 1342 | REGULATION\_OF\_PRIMARY\_METABOLIC\_PROCESS(GO:0080090) |  | 3304 | 0.16 | 0.98 | 0.459 | 0.724 | 1.000 | 7127 | tags=18%, list=22%, signal=21% |
| 1343 | INTRACELLULAR\_VESICLE(GO:0097708) |  | 796 | 0.16 | 0.98 | 0.486 | 0.724 | 1.000 | 7243 | tags=18%, list=23%, signal=23% |
| 1344 | INORGANIC\_ANION\_TRANSPORT(GO:0015698) |  | 115 | 0.25 | 0.98 | 0.473 | 0.726 | 1.000 | 3992 | tags=13%, list=13%, signal=15% |
| 1345 | GENE\_SILENCING\_BY\_MIRNA(GO:0035195) |  | 57 | 0.27 | 0.98 | 0.505 | 0.725 | 1.000 | 6084 | tags=21%, list=19%, signal=26% |
| 1346 | REGULATION\_OF\_CELLULAR\_PH(GO:0030641) |  | 40 | 0.24 | 0.98 | 0.376 | 0.726 | 1.000 | 10367 | tags=40%, list=33%, signal=59% |
| 1347 | INTEGRAL\_COMPONENT\_OF\_PLASMA\_MEMBRANE(GO:0005887) |  | 1012 | 0.21 | 0.98 | 0.421 | 0.726 | 1.000 | 7008 | tags=22%, list=22%, signal=27% |
| 1348 | LIPID\_BINDING(GO:0008289) |  | 440 | 0.17 | 0.98 | 0.415 | 0.727 | 1.000 | 4877 | tags=12%, list=15%, signal=14% |
| 1349 | CYTOPLASMIC\_RIBONUCLEOPROTEIN\_GRANULE(GO:0036464) |  | 99 | 0.20 | 0.98 | 0.492 | 0.726 | 1.000 | 1628 | tags=5%, list=5%, signal=5% |
| 1350 | SYNAPTIC\_VESICLE(GO:0008021) |  | 111 | 0.21 | 0.98 | 0.496 | 0.726 | 1.000 | 6511 | tags=17%, list=20%, signal=21% |
| 1351 | ENDORIBONUCLEASE\_COMPLEX(GO:1902555) |  | 15 | 0.38 | 0.98 | 0.463 | 0.727 | 1.000 | 5016 | tags=27%, list=16%, signal=32% |
| 1352 | NEGATIVE\_REGULATION\_OF\_AXON\_EXTENSION\_INVOLVED\_IN\_AXON\_GUIDANCE(GO:0048843) |  | 30 | 0.31 | 0.98 | 0.486 | 0.727 | 1.000 | 2608 | tags=10%, list=8%, signal=11% |
| 1353 | NEGATIVE\_REGULATION\_OF\_AXON\_GUIDANCE(GO:1902668) |  | 30 | 0.31 | 0.98 | 0.486 | 0.727 | 1.000 | 2608 | tags=10%, list=8%, signal=11% |
| 1354 | MITOCHONDRIAL\_RESPIRATORY\_CHAIN(GO:0005746) |  | 64 | 0.26 | 0.98 | 0.444 | 0.727 | 1.000 | 1769 | tags=5%, list=6%, signal=5% |
| 1355 | POSITIVE\_REGULATION\_OF\_MRNA\_CATABOLIC\_PROCESS(GO:0061014) |  | 19 | 0.34 | 0.98 | 0.445 | 0.726 | 1.000 | 913 | tags=11%, list=3%, signal=11% |
| 1356 | FEMALE\_GAMETE\_GENERATION(GO:0007292) |  | 62 | 0.26 | 0.98 | 0.436 | 0.726 | 1.000 | 6606 | tags=21%, list=21%, signal=26% |
| 1357 | REGULATION\_OF\_PROTEIN\_DEPOLYMERIZATION(GO:1901879) |  | 53 | 0.22 | 0.98 | 0.523 | 0.725 | 1.000 | 3342 | tags=11%, list=10%, signal=13% |
| 1358 | MYELOID\_LEUKOCYTE\_ACTIVATION(GO:0002274) |  | 32 | 0.29 | 0.98 | 0.489 | 0.725 | 1.000 | 7532 | tags=34%, list=24%, signal=45% |
| 1359 | DOUBLE\_STRAND\_BREAK\_REPAIR\_VIA\_NONHOMOLOGOUS\_END\_JOINING(GO:0006303) |  | 23 | 0.29 | 0.98 | 0.475 | 0.725 | 1.000 | 3853 | tags=26%, list=12%, signal=30% |
| 1360 | NEGATIVE\_REGULATION\_OF\_CHEMOTAXIS(GO:0050922) |  | 34 | 0.31 | 0.98 | 0.498 | 0.725 | 1.000 | 11535 | tags=47%, list=36%, signal=74% |
| 1361 | REGULATION\_OF\_MRNA\_SPLICING\_\_VIA\_SPLICEOSOME(GO:0048024) |  | 73 | 0.19 | 0.98 | 0.547 | 0.725 | 1.000 | 7521 | tags=18%, list=24%, signal=23% |
| 1362 | CHROMATIN\_BINDING(GO:0003682) |  | 239 | 0.18 | 0.98 | 0.463 | 0.725 | 1.000 | 1863 | tags=4%, list=6%, signal=4% |
| 1363 | CELLULAR\_DETOXIFICATION(GO:1990748) |  | 16 | 0.36 | 0.97 | 0.557 | 0.726 | 1.000 | 148 | tags=6%, list=0%, signal=6% |
| 1364 | REGULATION\_OF\_AXON\_EXTENSION\_INVOLVED\_IN\_AXON\_GUIDANCE(GO:0048841) |  | 37 | 0.27 | 0.97 | 0.469 | 0.725 | 1.000 | 11535 | tags=43%, list=36%, signal=68% |
| 1365 | NEGATIVE\_REGULATION\_OF\_DEVELOPMENTAL\_PROCESS(GO:0051093) |  | 187 | 0.21 | 0.97 | 0.460 | 0.725 | 1.000 | 2608 | tags=7%, list=8%, signal=8% |
| 1366 | CATALYTIC\_STEP\_2\_SPLICEOSOME(GO:0071013) |  | 41 | 0.20 | 0.97 | 0.477 | 0.726 | 1.000 | 10963 | tags=37%, list=34%, signal=56% |
| 1367 | CYTOCHROME\_C\_OXIDASE\_ACTIVITY(GO:0004129) |  | 24 | 0.27 | 0.97 | 0.512 | 0.725 | 1.000 | 2518 | tags=8%, list=8%, signal=9% |
| 1368 | HEME\_COPPER\_TERMINAL\_OXIDASE\_ACTIVITY(GO:0015002) |  | 24 | 0.27 | 0.97 | 0.512 | 0.725 | 1.000 | 2518 | tags=8%, list=8%, signal=9% |
| 1369 | OXIDOREDUCTASE\_ACTIVITY\_\_ACTING\_ON\_A\_HEME\_GROUP\_OF\_DONORS(GO:0016675) |  | 24 | 0.27 | 0.97 | 0.512 | 0.724 | 1.000 | 2518 | tags=8%, list=8%, signal=9% |
| 1370 | OXIDOREDUCTASE\_ACTIVITY\_\_ACTING\_ON\_A\_HEME\_GROUP\_OF\_DONORS\_\_OXYGEN\_AS\_ACCEPTOR(GO:0016676) |  | 24 | 0.27 | 0.97 | 0.512 | 0.724 | 1.000 | 2518 | tags=8%, list=8%, signal=9% |
| 1371 | REGULATION\_OF\_NITROGEN\_COMPOUND\_METABOLIC\_PROCESS(GO:0051171) |  | 3231 | 0.16 | 0.97 | 0.512 | 0.723 | 1.000 | 7127 | tags=18%, list=22%, signal=21% |
| 1372 | INTEGRAL\_COMPONENT\_OF\_ENDOPLASMIC\_RETICULUM\_MEMBRANE(GO:0030176) |  | 103 | 0.22 | 0.97 | 0.374 | 0.723 | 1.000 | 5606 | tags=18%, list=18%, signal=22% |
| 1373 | AMINOGLYCAN\_METABOLIC\_PROCESS(GO:0006022) |  | 75 | 0.25 | 0.97 | 0.423 | 0.723 | 1.000 | 7006 | tags=31%, list=22%, signal=39% |
| 1374 | RNA\_METHYLATION(GO:0001510) |  | 51 | 0.23 | 0.97 | 0.413 | 0.725 | 1.000 | 1840 | tags=4%, list=6%, signal=4% |
| 1375 | REGULATION\_OF\_NEURON\_DIFFERENTIATION(GO:0045664) |  | 219 | 0.21 | 0.97 | 0.445 | 0.724 | 1.000 | 3713 | tags=9%, list=12%, signal=10% |
| 1376 | GASTRULATION(GO:0007369) |  | 246 | 0.18 | 0.97 | 0.547 | 0.726 | 1.000 | 4468 | tags=11%, list=14%, signal=13% |
| 1377 | REGULATION\_OF\_GTPASE\_ACTIVITY(GO:0043087) |  | 168 | 0.21 | 0.97 | 0.569 | 0.725 | 1.000 | 6791 | tags=19%, list=21%, signal=24% |
| 1378 | DEVELOPMENTAL\_PROCESS\_INVOLVED\_IN\_REPRODUCTION(GO:0003006) |  | 107 | 0.22 | 0.97 | 0.549 | 0.726 | 1.000 | 9804 | tags=36%, list=31%, signal=51% |
| 1379 | ERYTHROCYTE\_HOMEOSTASIS(GO:0034101) |  | 116 | 0.19 | 0.97 | 0.413 | 0.726 | 1.000 | 2287 | tags=7%, list=7%, signal=7% |
| 1380 | NEUROMAST\_PRIMORDIUM\_MIGRATION(GO:0048883) |  | 26 | 0.23 | 0.97 | 0.457 | 0.726 | 1.000 | 439 | tags=4%, list=1%, signal=4% |
| 1381 | NEGATIVE\_REGULATION\_OF\_CELL\_COMMUNICATION(GO:0010648) |  | 425 | 0.17 | 0.97 | 0.467 | 0.726 | 1.000 | 4470 | tags=12%, list=14%, signal=14% |
| 1382 | NEGATIVE\_REGULATION\_OF\_SIGNALING(GO:0023057) |  | 425 | 0.17 | 0.97 | 0.467 | 0.725 | 1.000 | 4470 | tags=12%, list=14%, signal=14% |
| 1383 | INTRINSIC\_COMPONENT\_OF\_PLASMA\_MEMBRANE(GO:0031226) |  | 1047 | 0.21 | 0.97 | 0.451 | 0.726 | 1.000 | 6721 | tags=20%, list=21%, signal=25% |
| 1384 | CHROMOSOMAL\_REGION(GO:0098687) |  | 105 | 0.18 | 0.97 | 0.402 | 0.726 | 1.000 | 7470 | tags=22%, list=23%, signal=29% |
| 1385 | SYNAPTIC\_VESICLE\_TRANSPORT(GO:0048489) |  | 68 | 0.25 | 0.97 | 0.475 | 0.726 | 1.000 | 2932 | tags=10%, list=9%, signal=11% |
| 1386 | SYNAPTIC\_VESICLE\_LOCALIZATION(GO:0097479) |  | 68 | 0.25 | 0.97 | 0.475 | 0.725 | 1.000 | 2932 | tags=10%, list=9%, signal=11% |
| 1387 | ESTABLISHMENT\_OF\_SYNAPTIC\_VESICLE\_LOCALIZATION(GO:0097480) |  | 68 | 0.25 | 0.97 | 0.475 | 0.725 | 1.000 | 2932 | tags=10%, list=9%, signal=11% |
| 1388 | ERYTHROCYTE\_DIFFERENTIATION(GO:0030218) |  | 115 | 0.19 | 0.97 | 0.413 | 0.725 | 1.000 | 2287 | tags=7%, list=7%, signal=7% |
| 1389 | INNATE\_IMMUNE\_RESPONSE(GO:0045087) |  | 198 | 0.21 | 0.97 | 0.535 | 0.725 | 1.000 | 7485 | tags=30%, list=23%, signal=39% |
| 1390 | DNA\_METHYLATION\_OR\_DEMETHYLATION(GO:0044728) |  | 34 | 0.29 | 0.97 | 0.602 | 0.725 | 1.000 | 7555 | tags=32%, list=24%, signal=42% |
| 1391 | STEROID\_HYDROXYLASE\_ACTIVITY(GO:0008395) |  | 60 | 0.30 | 0.97 | 0.446 | 0.726 | 1.000 | 6399 | tags=37%, list=20%, signal=46% |
| 1392 | COAGULATION(GO:0050817) |  | 64 | 0.26 | 0.97 | 0.435 | 0.728 | 1.000 | 3441 | tags=17%, list=11%, signal=19% |
| 1393 | ORGANIC\_HYDROXY\_COMPOUND\_TRANSPORT(GO:0015850) |  | 83 | 0.26 | 0.97 | 0.502 | 0.729 | 1.000 | 4752 | tags=17%, list=15%, signal=20% |
| 1394 | MEMBRANE\_PROTEIN\_PROTEOLYSIS(GO:0033619) |  | 22 | 0.27 | 0.97 | 0.533 | 0.729 | 1.000 | 6162 | tags=18%, list=19%, signal=23% |
| 1395 | SNRNA\_METABOLIC\_PROCESS(GO:0016073) |  | 38 | 0.24 | 0.97 | 0.523 | 0.729 | 1.000 | 6939 | tags=18%, list=22%, signal=24% |
| 1396 | CYCLIC\_NUCLEOTIDE\_MEDIATED\_SIGNALING(GO:0019935) |  | 128 | 0.24 | 0.97 | 0.479 | 0.729 | 1.000 | 5401 | tags=18%, list=17%, signal=22% |
| 1397 | ENTERIC\_NERVOUS\_SYSTEM\_DEVELOPMENT(GO:0048484) |  | 39 | 0.25 | 0.97 | 0.486 | 0.729 | 1.000 | 8658 | tags=31%, list=27%, signal=42% |
| 1398 | HEPARIN\_BINDING(GO:0008201) |  | 70 | 0.20 | 0.97 | 0.515 | 0.730 | 1.000 | 7427 | tags=23%, list=23%, signal=30% |
| 1399 | PYRIMIDINE\_CONTAINING\_COMPOUND\_METABOLIC\_PROCESS(GO:0072527) |  | 53 | 0.22 | 0.96 | 0.502 | 0.731 | 1.000 | 4461 | tags=13%, list=14%, signal=15% |
| 1400 | NUCLEOSIDE\_SALVAGE(GO:0043174) |  | 16 | 0.31 | 0.96 | 0.592 | 0.732 | 1.000 | 1678 | tags=13%, list=5%, signal=13% |
| 1401 | POSITIVE\_REGULATION\_OF\_CELL\_PROLIFERATION(GO:0008284) |  | 124 | 0.21 | 0.96 | 0.614 | 0.731 | 1.000 | 7105 | tags=26%, list=22%, signal=33% |
| 1402 | BLOOD\_COAGULATION(GO:0007596) |  | 61 | 0.27 | 0.96 | 0.435 | 0.733 | 1.000 | 3441 | tags=18%, list=11%, signal=20% |
| 1403 | HYPOTHALAMUS\_DEVELOPMENT(GO:0021854) |  | 25 | 0.31 | 0.96 | 0.523 | 0.733 | 1.000 | 3530 | tags=16%, list=11%, signal=18% |
| 1404 | COLLAGEN\_CATABOLIC\_PROCESS(GO:0030574) |  | 19 | 0.28 | 0.96 | 0.437 | 0.733 | 1.000 | 6316 | tags=37%, list=20%, signal=46% |
| 1405 | POSITIVE\_REGULATION\_OF\_CELLULAR\_CATABOLIC\_PROCESS(GO:0031331) |  | 76 | 0.20 | 0.96 | 0.466 | 0.734 | 1.000 | 3850 | tags=9%, list=12%, signal=10% |
| 1406 | SOLUTE:PROTON\_ANTIPORTER\_ACTIVITY(GO:0015299) |  | 18 | 0.28 | 0.96 | 0.366 | 0.734 | 1.000 | 6829 | tags=28%, list=21%, signal=35% |
| 1407 | PROTEIN\_KINASE\_ACTIVITY(GO:0004672) |  | 656 | 0.16 | 0.96 | 0.544 | 0.733 | 1.000 | 7681 | tags=21%, list=24%, signal=27% |
| 1408 | LONG\_CHAIN\_FATTY\_ACID\_TRANSPORT(GO:0015909) |  | 23 | 0.32 | 0.96 | 0.487 | 0.735 | 1.000 | 2057 | tags=17%, list=6%, signal=19% |
| 1409 | REGULATION\_OF\_CELL\_DEVELOPMENT(GO:0060284) |  | 329 | 0.20 | 0.96 | 0.494 | 0.735 | 1.000 | 3713 | tags=9%, list=12%, signal=10% |
| 1410 | CHEMOKINE\_BINDING(GO:0019956) |  | 24 | 0.31 | 0.96 | 0.414 | 0.735 | 1.000 | 4577 | tags=25%, list=14%, signal=29% |
| 1411 | REGULATION\_OF\_SYNAPSE\_ORGANIZATION(GO:0050807) |  | 37 | 0.28 | 0.96 | 0.544 | 0.735 | 1.000 | 953 | tags=5%, list=3%, signal=6% |
| 1412 | UNFOLDED\_PROTEIN\_BINDING(GO:0051082) |  | 76 | 0.21 | 0.96 | 0.541 | 0.739 | 1.000 | 3165 | tags=9%, list=10%, signal=10% |
| 1413 | REGULATION\_OF\_SYSTEMIC\_ARTERIAL\_BLOOD\_PRESSURE(GO:0003073) |  | 27 | 0.28 | 0.96 | 0.486 | 0.740 | 1.000 | 10070 | tags=44%, list=32%, signal=65% |
| 1414 | EXOCYTIC\_VESICLE(GO:0070382) |  | 131 | 0.21 | 0.96 | 0.501 | 0.740 | 1.000 | 6511 | tags=18%, list=20%, signal=22% |
| 1415 | NUCLEOTIDE\_SALVAGE(GO:0043173) |  | 21 | 0.28 | 0.96 | 0.518 | 0.741 | 1.000 | 4313 | tags=19%, list=14%, signal=22% |
| 1416 | MRNA\_METABOLIC\_PROCESS(GO:0016071) |  | 426 | 0.12 | 0.95 | 0.496 | 0.745 | 1.000 | 7585 | tags=15%, list=24%, signal=19% |
| 1417 | CATALYTIC\_ACTIVITY\_\_ACTING\_ON\_DNA(GO:0140097) |  | 156 | 0.18 | 0.95 | 0.555 | 0.745 | 1.000 | 7211 | tags=21%, list=23%, signal=26% |
| 1418 | AXONEME\_PART(GO:0044447) |  | 20 | 0.31 | 0.95 | 0.597 | 0.745 | 1.000 | 4880 | tags=25%, list=15%, signal=29% |
| 1419 | PLASMA\_MEMBRANE\_BOUNDED\_CELL\_PROJECTION\_CYTOPLASM(GO:0032838) |  | 58 | 0.26 | 0.95 | 0.523 | 0.745 | 1.000 | 6675 | tags=33%, list=21%, signal=41% |
| 1420 | LONG\_CHAIN\_FATTY\_ACYL\_COA\_METABOLIC\_PROCESS(GO:0035336) |  | 17 | 0.37 | 0.95 | 0.554 | 0.745 | 1.000 | 5835 | tags=35%, list=18%, signal=43% |
| 1421 | RIBONUCLEOTIDE\_CATABOLIC\_PROCESS(GO:0009261) |  | 19 | 0.34 | 0.95 | 0.526 | 0.744 | 1.000 | 9805 | tags=42%, list=31%, signal=61% |
| 1422 | DNA\_CONFORMATION\_CHANGE(GO:0071103) |  | 116 | 0.20 | 0.95 | 0.438 | 0.744 | 1.000 | 10671 | tags=34%, list=33%, signal=50% |
| 1423 | PHOSPHOTRANSFERASE\_ACTIVITY\_\_ALCOHOL\_GROUP\_AS\_ACCEPTOR(GO:0016773) |  | 766 | 0.16 | 0.95 | 0.619 | 0.744 | 1.000 | 7681 | tags=21%, list=24%, signal=27% |
| 1424 | CHROMOSOME\_SEPARATION(GO:0051304) |  | 43 | 0.27 | 0.95 | 0.487 | 0.744 | 1.000 | 4148 | tags=12%, list=13%, signal=13% |
| 1425 | DELAMINATION(GO:0060232) |  | 16 | 0.30 | 0.95 | 0.537 | 0.744 | 1.000 | 9734 | tags=44%, list=31%, signal=63% |
| 1426 | PEPTIDE\_BIOSYNTHETIC\_PROCESS(GO:0043043) |  | 437 | 0.18 | 0.95 | 0.494 | 0.749 | 1.000 | 2112 | tags=4%, list=7%, signal=5% |
| 1427 | TRANSLATION(GO:0006412) |  | 432 | 0.18 | 0.95 | 0.494 | 0.749 | 1.000 | 2112 | tags=4%, list=7%, signal=5% |
| 1428 | DETOXIFICATION(GO:0098754) |  | 25 | 0.27 | 0.95 | 0.498 | 0.749 | 1.000 | 148 | tags=4%, list=0%, signal=4% |
| 1429 | G\_PROTEIN\_COUPLED\_PEPTIDE\_RECEPTOR\_ACTIVITY(GO:0008528) |  | 144 | 0.25 | 0.95 | 0.626 | 0.749 | 1.000 | 5561 | tags=23%, list=17%, signal=28% |
| 1430 | REGULATION\_OF\_SKELETAL\_MUSCLE\_TISSUE\_DEVELOPMENT(GO:0048641) |  | 15 | 0.35 | 0.95 | 0.576 | 0.749 | 1.000 | 6856 | tags=33%, list=21%, signal=42% |
| 1431 | RESPONSE\_TO\_KETONE(GO:1901654) |  | 16 | 0.36 | 0.95 | 0.506 | 0.749 | 1.000 | 5036 | tags=25%, list=16%, signal=30% |
| 1432 | POSITIVE\_REGULATION\_OF\_ORGANELLE\_ORGANIZATION(GO:0010638) |  | 160 | 0.16 | 0.95 | 0.583 | 0.750 | 1.000 | 7020 | tags=17%, list=22%, signal=22% |
| 1433 | PROXIMAL\_PROMOTER\_SEQUENCE\_SPECIFIC\_DNA\_BINDING(GO:0000987) |  | 939 | 0.19 | 0.95 | 0.485 | 0.750 | 1.000 | 7415 | tags=22%, list=23%, signal=28% |
| 1434 | REGULATION\_OF\_MULTICELLULAR\_ORGANISMAL\_PROCESS(GO:0051239) |  | 943 | 0.17 | 0.95 | 0.604 | 0.750 | 1.000 | 8060 | tags=21%, list=25%, signal=28% |
| 1435 | ANCHORED\_COMPONENT\_OF\_MEMBRANE(GO:0031225) |  | 52 | 0.27 | 0.95 | 0.509 | 0.750 | 1.000 | 3632 | tags=12%, list=11%, signal=13% |
| 1436 | POSITIVE\_REGULATION\_OF\_PROTEOLYSIS\_INVOLVED\_IN\_CELLULAR\_PROTEIN\_CATABOLIC\_PROCESS(GO:1903052) |  | 32 | 0.22 | 0.95 | 0.517 | 0.750 | 1.000 | 5879 | tags=16%, list=18%, signal=19% |
| 1437 | POSITIVE\_REGULATION\_OF\_CELLULAR\_PROTEIN\_CATABOLIC\_PROCESS(GO:1903364) |  | 32 | 0.22 | 0.95 | 0.517 | 0.750 | 1.000 | 5879 | tags=16%, list=18%, signal=19% |
| 1438 | PHOSPHOTRANSFERASE\_ACTIVITY\_\_PHOSPHATE\_GROUP\_AS\_ACCEPTOR(GO:0016776) |  | 36 | 0.27 | 0.95 | 0.442 | 0.754 | 1.000 | 1388 | tags=6%, list=4%, signal=6% |
| 1439 | RNA\_POLYMERASE\_II\_PROXIMAL\_PROMOTER\_SEQUENCE\_SPECIFIC\_DNA\_BINDING(GO:0000978) |  | 929 | 0.19 | 0.95 | 0.505 | 0.753 | 1.000 | 7099 | tags=21%, list=22%, signal=26% |
| 1440 | TRNA\_MODIFICATION(GO:0006400) |  | 61 | 0.22 | 0.95 | 0.574 | 0.753 | 1.000 | 1840 | tags=5%, list=6%, signal=5% |
| 1441 | VERY\_LONG\_CHAIN\_FATTY\_ACID\_METABOLIC\_PROCESS(GO:0000038) |  | 28 | 0.30 | 0.95 | 0.493 | 0.753 | 1.000 | 4729 | tags=25%, list=15%, signal=29% |
| 1442 | CHROMOSOME\_\_CENTROMERIC\_REGION(GO:0000775) |  | 81 | 0.18 | 0.95 | 0.488 | 0.753 | 1.000 | 7470 | tags=22%, list=23%, signal=29% |
| 1443 | RIBONUCLEOPROTEIN\_GRANULE(GO:0035770) |  | 106 | 0.19 | 0.95 | 0.520 | 0.753 | 1.000 | 1628 | tags=5%, list=5%, signal=5% |
| 1444 | HISTONE\_DEACETYLASE\_COMPLEX(GO:0000118) |  | 38 | 0.28 | 0.94 | 0.528 | 0.754 | 1.000 | 10916 | tags=47%, list=34%, signal=72% |
| 1445 | DEVELOPMENTAL\_PROCESS(GO:0032502) |  | 4527 | 0.14 | 0.94 | 0.604 | 0.754 | 1.000 | 7328 | tags=18%, list=23%, signal=20% |
| 1446 | MATURATION\_OF\_5.8S\_RRNA\_FROM\_TRICISTRONIC\_RRNA\_TRANSCRIPT\_(SSU\_RRNA\_\_5.8S\_RRNA\_\_LSU\_RRNA)(GO:0000466) |  | 21 | 0.30 | 0.94 | 0.481 | 0.754 | 1.000 | 470 | tags=5%, list=1%, signal=5% |
| 1447 | AXONEME(GO:0005930) |  | 55 | 0.26 | 0.94 | 0.543 | 0.755 | 1.000 | 6675 | tags=33%, list=21%, signal=41% |
| 1448 | POSITIVE\_REGULATION\_OF\_UBIQUITIN\_DEPENDENT\_PROTEIN\_CATABOLIC\_PROCESS(GO:2000060) |  | 31 | 0.23 | 0.94 | 0.485 | 0.756 | 1.000 | 5879 | tags=16%, list=18%, signal=20% |
| 1449 | NUCLEOSOMAL\_DNA\_BINDING(GO:0031492) |  | 22 | 0.28 | 0.94 | 0.616 | 0.757 | 1.000 | 792 | tags=9%, list=2%, signal=9% |
| 1450 | REGULATION\_OF\_POLYSACCHARIDE\_METABOLIC\_PROCESS(GO:0032881) |  | 16 | 0.34 | 0.94 | 0.499 | 0.756 | 1.000 | 2515 | tags=25%, list=8%, signal=27% |
| 1451 | MORPHOGENESIS\_OF\_A\_BRANCHING\_STRUCTURE(GO:0001763) |  | 28 | 0.29 | 0.94 | 0.550 | 0.757 | 1.000 | 1089 | tags=7%, list=3%, signal=7% |
| 1452 | MULTICELLULAR\_ORGANISMAL\_PROCESS(GO:0032501) |  | 4770 | 0.15 | 0.94 | 0.623 | 0.757 | 1.000 | 7230 | tags=18%, list=23%, signal=20% |
| 1453 | CARTILAGE\_DEVELOPMENT(GO:0051216) |  | 155 | 0.17 | 0.94 | 0.563 | 0.758 | 1.000 | 4376 | tags=10%, list=14%, signal=12% |
| 1454 | AMIDE\_BINDING(GO:0033218) |  | 138 | 0.19 | 0.94 | 0.565 | 0.761 | 1.000 | 10429 | tags=38%, list=33%, signal=56% |
| 1455 | NUCLEAR\_UBIQUITIN\_LIGASE\_COMPLEX(GO:0000152) |  | 32 | 0.25 | 0.94 | 0.481 | 0.761 | 1.000 | 5024 | tags=13%, list=16%, signal=15% |
| 1456 | POSITIVE\_REGULATION\_OF\_SIGNAL\_TRANSDUCTION(GO:0009967) |  | 410 | 0.17 | 0.94 | 0.569 | 0.761 | 1.000 | 4398 | tags=13%, list=14%, signal=15% |
| 1457 | HEMATOPOIETIC\_PROGENITOR\_CELL\_DIFFERENTIATION(GO:0002244) |  | 124 | 0.17 | 0.94 | 0.632 | 0.760 | 1.000 | 4504 | tags=10%, list=14%, signal=12% |
| 1458 | ANATOMICAL\_STRUCTURE\_DEVELOPMENT(GO:0048856) |  | 4379 | 0.14 | 0.94 | 0.614 | 0.760 | 1.000 | 7323 | tags=18%, list=23%, signal=20% |
| 1459 | HEMOSTASIS(GO:0007599) |  | 62 | 0.27 | 0.94 | 0.508 | 0.761 | 1.000 | 3441 | tags=18%, list=11%, signal=20% |
| 1460 | MESODERMAL\_CELL\_DIFFERENTIATION(GO:0048333) |  | 28 | 0.28 | 0.94 | 0.484 | 0.761 | 1.000 | 8538 | tags=32%, list=27%, signal=44% |
| 1461 | GLYCOGEN\_BIOSYNTHETIC\_PROCESS(GO:0005978) |  | 27 | 0.26 | 0.94 | 0.517 | 0.762 | 1.000 | 2515 | tags=15%, list=8%, signal=16% |
| 1462 | GLUCAN\_BIOSYNTHETIC\_PROCESS(GO:0009250) |  | 27 | 0.26 | 0.94 | 0.517 | 0.762 | 1.000 | 2515 | tags=15%, list=8%, signal=16% |
| 1463 | PLASMA\_MEMBRANE\_PART(GO:0044459) |  | 1707 | 0.18 | 0.94 | 0.475 | 0.763 | 1.000 | 7066 | tags=21%, list=22%, signal=26% |
| 1464 | INDOLE\_CONTAINING\_COMPOUND\_METABOLIC\_PROCESS(GO:0042430) |  | 16 | 0.33 | 0.94 | 0.504 | 0.765 | 1.000 | 7790 | tags=44%, list=24%, signal=58% |
| 1465 | EXCITATORY\_EXTRACELLULAR\_LIGAND\_GATED\_ION\_CHANNEL\_ACTIVITY(GO:0005231) |  | 38 | 0.33 | 0.93 | 0.597 | 0.765 | 1.000 | 765 | tags=8%, list=2%, signal=8% |
| 1466 | ENDOPLASMIC\_RETICULUM\_GOLGI\_INTERMEDIATE\_COMPARTMENT\_MEMBRANE(GO:0033116) |  | 22 | 0.27 | 0.93 | 0.518 | 0.767 | 1.000 | 5187 | tags=23%, list=16%, signal=27% |
| 1467 | PHOSPHATIDYLINOSITOL\_PHOSPHATE\_KINASE\_ACTIVITY(GO:0016307) |  | 18 | 0.26 | 0.93 | 0.480 | 0.769 | 1.000 | 8828 | tags=22%, list=28%, signal=31% |
| 1468 | PEPTIDYL\_ARGININE\_MODIFICATION(GO:0018195) |  | 18 | 0.30 | 0.93 | 0.557 | 0.769 | 1.000 | 9653 | tags=44%, list=30%, signal=64% |
| 1469 | CARBOHYDRATE\_KINASE\_ACTIVITY(GO:0019200) |  | 26 | 0.31 | 0.93 | 0.577 | 0.768 | 1.000 | 4685 | tags=27%, list=15%, signal=32% |
| 1470 | LIPID\_OXIDATION(GO:0034440) |  | 49 | 0.27 | 0.93 | 0.514 | 0.768 | 1.000 | 6866 | tags=27%, list=22%, signal=34% |
| 1471 | NOTCH\_SIGNALING\_PATHWAY(GO:0007219) |  | 120 | 0.18 | 0.93 | 0.527 | 0.768 | 1.000 | 7242 | tags=20%, list=23%, signal=26% |
| 1472 | CLATHRIN\_VESICLE\_COAT(GO:0030125) |  | 24 | 0.26 | 0.93 | 0.680 | 0.769 | 1.000 | 8018 | tags=29%, list=25%, signal=39% |
| 1473 | ROSTROCAUDAL\_NEURAL\_TUBE\_PATTERNING(GO:0021903) |  | 46 | 0.22 | 0.93 | 0.523 | 0.768 | 1.000 | 4244 | tags=13%, list=13%, signal=15% |
| 1474 | PECTORAL\_FIN\_MORPHOGENESIS(GO:0035138) |  | 30 | 0.24 | 0.93 | 0.570 | 0.769 | 1.000 | 3713 | tags=13%, list=12%, signal=15% |
| 1475 | CILIARY\_PLASM(GO:0097014) |  | 56 | 0.26 | 0.93 | 0.581 | 0.772 | 1.000 | 6675 | tags=32%, list=21%, signal=41% |
| 1476 | G\_PROTEIN\_COUPLED\_CHEMOATTRACTANT\_RECEPTOR\_ACTIVITY(GO:0001637) |  | 27 | 0.34 | 0.93 | 0.510 | 0.772 | 1.000 | 4577 | tags=22%, list=14%, signal=26% |
| 1477 | CHEMOKINE\_RECEPTOR\_ACTIVITY(GO:0004950) |  | 27 | 0.34 | 0.93 | 0.510 | 0.772 | 1.000 | 4577 | tags=22%, list=14%, signal=26% |
| 1478 | BASE\_EXCISION\_REPAIR(GO:0006284) |  | 25 | 0.27 | 0.93 | 0.642 | 0.771 | 1.000 | 8509 | tags=32%, list=27%, signal=44% |
| 1479 | G\_PROTEIN\_COUPLED\_RECEPTOR\_BINDING(GO:0001664) |  | 155 | 0.22 | 0.93 | 0.574 | 0.773 | 1.000 | 4445 | tags=15%, list=14%, signal=17% |
| 1480 | NEGATIVE\_REGULATION\_OF\_CELLULAR\_COMPONENT\_ORGANIZATION(GO:0051129) |  | 193 | 0.18 | 0.93 | 0.645 | 0.773 | 1.000 | 3538 | tags=9%, list=11%, signal=10% |
| 1481 | REGULATION\_OF\_AXON\_EXTENSION(GO:0030516) |  | 68 | 0.23 | 0.93 | 0.537 | 0.774 | 1.000 | 8709 | tags=26%, list=27%, signal=36% |
| 1482 | PROTEIN\_DNA\_COMPLEX\_ASSEMBLY(GO:0065004) |  | 94 | 0.20 | 0.93 | 0.563 | 0.774 | 1.000 | 2232 | tags=7%, list=7%, signal=8% |
| 1483 | ANTIGEN\_RECEPTOR\_MEDIATED\_SIGNALING\_PATHWAY(GO:0050851) |  | 40 | 0.27 | 0.93 | 0.555 | 0.775 | 1.000 | 6666 | tags=35%, list=21%, signal=44% |
| 1484 | NUCLEOSIDE\_TRIPHOSPHATASE\_REGULATOR\_ACTIVITY(GO:0060589) |  | 238 | 0.17 | 0.93 | 0.626 | 0.776 | 1.000 | 2864 | tags=7%, list=9%, signal=7% |
| 1485 | POSITIVE\_REGULATION\_OF\_CELL\_CYCLE\_PROCESS(GO:0090068) |  | 44 | 0.21 | 0.93 | 0.539 | 0.775 | 1.000 | 7020 | tags=25%, list=22%, signal=32% |
| 1486 | POSITIVE\_REGULATION\_OF\_PROTEASOMAL\_PROTEIN\_CATABOLIC\_PROCESS(GO:1901800) |  | 31 | 0.22 | 0.92 | 0.565 | 0.776 | 1.000 | 5879 | tags=16%, list=18%, signal=20% |
| 1487 | REGULATION\_OF\_GLYCOGEN\_METABOLIC\_PROCESS(GO:0070873) |  | 15 | 0.35 | 0.92 | 0.499 | 0.777 | 1.000 | 2515 | tags=27%, list=8%, signal=29% |
| 1488 | SULFUR\_COMPOUND\_TRANSPORT(GO:0072348) |  | 25 | 0.30 | 0.92 | 0.458 | 0.777 | 1.000 | 8626 | tags=36%, list=27%, signal=49% |
| 1489 | S\_ADENOSYLMETHIONINE\_DEPENDENT\_METHYLTRANSFERASE\_ACTIVITY(GO:0008757) |  | 146 | 0.18 | 0.92 | 0.530 | 0.777 | 1.000 | 4299 | tags=11%, list=13%, signal=13% |
| 1490 | POSITIVE\_REGULATION\_OF\_PROTEASOMAL\_UBIQUITIN\_DEPENDENT\_PROTEIN\_CATABOLIC\_PROCESS(GO:0032436) |  | 30 | 0.23 | 0.92 | 0.524 | 0.777 | 1.000 | 5879 | tags=17%, list=18%, signal=20% |
| 1491 | UBIQUITIN\_CONJUGATING\_ENZYME\_ACTIVITY(GO:0061631) |  | 36 | 0.25 | 0.92 | 0.458 | 0.778 | 1.000 | 2994 | tags=8%, list=9%, signal=9% |
| 1492 | POSTSYNAPSE\_ORGANIZATION(GO:0099173) |  | 37 | 0.29 | 0.92 | 0.560 | 0.778 | 1.000 | 1043 | tags=5%, list=3%, signal=6% |
| 1493 | MYELOID\_CELL\_HOMEOSTASIS(GO:0002262) |  | 127 | 0.18 | 0.92 | 0.463 | 0.778 | 1.000 | 7146 | tags=19%, list=22%, signal=24% |
| 1494 | REGULATION\_OF\_RESPONSE\_TO\_DRUG(GO:2001023) |  | 27 | 0.29 | 0.92 | 0.512 | 0.777 | 1.000 | 1221 | tags=7%, list=4%, signal=8% |
| 1495 | TUBE\_MORPHOGENESIS(GO:0035239) |  | 620 | 0.16 | 0.92 | 0.602 | 0.779 | 1.000 | 3698 | tags=9%, list=12%, signal=9% |
| 1496 | MONOCARBOXYLIC\_ACID\_BIOSYNTHETIC\_PROCESS(GO:0072330) |  | 142 | 0.23 | 0.92 | 0.564 | 0.779 | 1.000 | 5292 | tags=18%, list=17%, signal=22% |
| 1497 | PEPTIDYL\_GLUTAMIC\_ACID\_MODIFICATION(GO:0018200) |  | 21 | 0.25 | 0.92 | 0.678 | 0.778 | 1.000 | 1971 | tags=10%, list=6%, signal=10% |
| 1498 | DORSAL\_VENTRAL\_AXIS\_SPECIFICATION(GO:0009950) |  | 22 | 0.30 | 0.92 | 0.564 | 0.778 | 1.000 | 4687 | tags=14%, list=15%, signal=16% |
| 1499 | CELLULAR\_MONOVALENT\_INORGANIC\_CATION\_HOMEOSTASIS(GO:0030004) |  | 55 | 0.21 | 0.92 | 0.509 | 0.778 | 1.000 | 7160 | tags=24%, list=22%, signal=30% |
| 1500 | PROTEIN\_LOCALIZATION\_TO\_POSTSYNAPTIC\_MEMBRANE(GO:1903539) |  | 15 | 0.30 | 0.92 | 0.574 | 0.778 | 1.000 | 3734 | tags=13%, list=12%, signal=15% |
| 1501 | MRNA\_SPLICE\_SITE\_SELECTION(GO:0006376) |  | 22 | 0.23 | 0.92 | 0.623 | 0.780 | 1.000 | 24639 | tags=100%, list=77%, signal=439% |
| 1502 | TUBE\_DEVELOPMENT(GO:0035295) |  | 785 | 0.15 | 0.92 | 0.602 | 0.780 | 1.000 | 3698 | tags=8%, list=12%, signal=9% |
| 1503 | GLYCOLIPID\_BIOSYNTHETIC\_PROCESS(GO:0009247) |  | 48 | 0.24 | 0.92 | 0.613 | 0.779 | 1.000 | 5007 | tags=15%, list=16%, signal=17% |
| 1504 | ORGANELLE\_TRANSPORT\_ALONG\_MICROTUBULE(GO:0072384) |  | 29 | 0.26 | 0.92 | 0.614 | 0.779 | 1.000 | 3299 | tags=10%, list=10%, signal=12% |
| 1505 | NEGATIVE\_REGULATION\_OF\_G\_PROTEIN\_COUPLED\_RECEPTOR\_PROTEIN\_SIGNALING\_PATHWAY(GO:0045744) |  | 15 | 0.39 | 0.92 | 0.659 | 0.782 | 1.000 | 2974 | tags=13%, list=9%, signal=15% |
| 1506 | REGULATION\_OF\_INTRINSIC\_APOPTOTIC\_SIGNALING\_PATHWAY(GO:2001242) |  | 32 | 0.22 | 0.92 | 0.541 | 0.785 | 1.000 | 9328 | tags=28%, list=29%, signal=40% |
| 1507 | FORMATION\_OF\_PRIMARY\_GERM\_LAYER(GO:0001704) |  | 66 | 0.23 | 0.92 | 0.573 | 0.785 | 1.000 | 7089 | tags=23%, list=22%, signal=29% |
| 1508 | WOUND\_HEALING(GO:0042060) |  | 224 | 0.18 | 0.91 | 0.532 | 0.786 | 1.000 | 7651 | tags=23%, list=24%, signal=30% |
| 1509 | REGULATION\_OF\_SYSTEMIC\_ARTERIAL\_BLOOD\_PRESSURE\_MEDIATED\_BY\_A\_CHEMICAL\_SIGNAL(GO:0003044) |  | 21 | 0.31 | 0.91 | 0.639 | 0.786 | 1.000 | 10070 | tags=48%, list=32%, signal=70% |
| 1510 | MRNA\_3'\_END\_PROCESSING(GO:0031124) |  | 29 | 0.23 | 0.91 | 0.474 | 0.786 | 1.000 | 944 | tags=3%, list=3%, signal=4% |
| 1511 | GROWTH\_FACTOR\_RECEPTOR\_BINDING(GO:0070851) |  | 57 | 0.26 | 0.91 | 0.516 | 0.788 | 1.000 | 7227 | tags=26%, list=23%, signal=34% |
| 1512 | REGULATION\_OF\_CHROMOSOME\_ORGANIZATION(GO:0033044) |  | 89 | 0.21 | 0.91 | 0.432 | 0.788 | 1.000 | 6220 | tags=16%, list=20%, signal=19% |
| 1513 | PLASMA\_MEMBRANE(GO:0005886) |  | 3166 | 0.17 | 0.91 | 0.614 | 0.788 | 1.000 | 7175 | tags=21%, list=22%, signal=24% |
| 1514 | DICARBOXYLIC\_ACID\_TRANSMEMBRANE\_TRANSPORTER\_ACTIVITY(GO:0005310) |  | 31 | 0.25 | 0.91 | 0.651 | 0.788 | 1.000 | 5783 | tags=19%, list=18%, signal=24% |
| 1515 | TELOMERIC\_DNA\_BINDING(GO:0042162) |  | 20 | 0.31 | 0.91 | 0.638 | 0.788 | 1.000 | 8735 | tags=30%, list=27%, signal=41% |
| 1516 | MITOTIC\_NUCLEAR\_DIVISION(GO:0140014) |  | 133 | 0.18 | 0.91 | 0.497 | 0.789 | 1.000 | 8998 | tags=26%, list=28%, signal=35% |
| 1517 | POSITIVE\_REGULATION\_OF\_PROTEIN\_TRANSPORT(GO:0051222) |  | 25 | 0.25 | 0.91 | 0.491 | 0.788 | 1.000 | 5859 | tags=24%, list=18%, signal=29% |
| 1518 | POSITIVE\_REGULATION\_OF\_ESTABLISHMENT\_OF\_PROTEIN\_LOCALIZATION(GO:1904951) |  | 25 | 0.25 | 0.91 | 0.491 | 0.788 | 1.000 | 5859 | tags=24%, list=18%, signal=29% |
| 1519 | SIGNAL\_SEQUENCE\_BINDING(GO:0005048) |  | 26 | 0.19 | 0.91 | 0.556 | 0.788 | 1.000 | 13495 | tags=54%, list=42%, signal=93% |
| 1520 | PROTEIN\_DNA\_COMPLEX\_SUBUNIT\_ORGANIZATION(GO:0071824) |  | 115 | 0.20 | 0.91 | 0.543 | 0.789 | 1.000 | 4047 | tags=10%, list=13%, signal=12% |
| 1521 | SOMATIC\_DIVERSIFICATION\_OF\_IMMUNE\_RECEPTORS(GO:0002200) |  | 16 | 0.34 | 0.91 | 0.540 | 0.789 | 1.000 | 12436 | tags=69%, list=39%, signal=113% |
| 1522 | TRNA\_PROCESSING(GO:0008033) |  | 97 | 0.19 | 0.91 | 0.558 | 0.789 | 1.000 | 5016 | tags=11%, list=16%, signal=13% |
| 1523 | HEPARAN\_SULFATE\_PROTEOGLYCAN\_BIOSYNTHETIC\_PROCESS(GO:0015012) |  | 24 | 0.25 | 0.91 | 0.554 | 0.789 | 1.000 | 4567 | tags=17%, list=14%, signal=19% |
| 1524 | GLUCOSIDASE\_ACTIVITY(GO:0015926) |  | 28 | 0.27 | 0.91 | 0.537 | 0.789 | 1.000 | 2333 | tags=7%, list=7%, signal=8% |
| 1525 | GLYCEROLIPID\_BIOSYNTHETIC\_PROCESS(GO:0045017) |  | 86 | 0.22 | 0.91 | 0.637 | 0.792 | 1.000 | 6267 | tags=17%, list=20%, signal=22% |
| 1526 | MONOVALENT\_INORGANIC\_ANION\_HOMEOSTASIS(GO:0055083) |  | 27 | 0.28 | 0.91 | 0.600 | 0.792 | 1.000 | 3992 | tags=15%, list=13%, signal=17% |
| 1527 | CHEMOREPELLENT\_ACTIVITY(GO:0045499) |  | 31 | 0.29 | 0.91 | 0.626 | 0.793 | 1.000 | 11535 | tags=48%, list=36%, signal=76% |
| 1528 | BRAIN\_MORPHOGENESIS(GO:0048854) |  | 28 | 0.28 | 0.91 | 0.601 | 0.793 | 1.000 | 10333 | tags=32%, list=32%, signal=48% |
| 1529 | ORGANIC\_HYDROXY\_COMPOUND\_TRANSMEMBRANE\_TRANSPORTER\_ACTIVITY(GO:1901618) |  | 30 | 0.31 | 0.91 | 0.551 | 0.793 | 1.000 | 6427 | tags=23%, list=20%, signal=29% |
| 1530 | CRANIAL\_SKELETAL\_SYSTEM\_DEVELOPMENT(GO:1904888) |  | 211 | 0.16 | 0.91 | 0.563 | 0.794 | 1.000 | 5643 | tags=11%, list=18%, signal=14% |
| 1531 | POSITIVE\_REGULATION\_OF\_APOPTOTIC\_SIGNALING\_PATHWAY(GO:2001235) |  | 17 | 0.30 | 0.91 | 0.560 | 0.793 | 1.000 | 3609 | tags=12%, list=11%, signal=13% |
| 1532 | TRNA\_METABOLIC\_PROCESS(GO:0006399) |  | 145 | 0.18 | 0.91 | 0.519 | 0.794 | 1.000 | 5032 | tags=11%, list=16%, signal=13% |
| 1533 | CELL\_CYCLE\_DNA\_REPLICATION(GO:0044786) |  | 18 | 0.29 | 0.91 | 0.595 | 0.794 | 1.000 | 9643 | tags=39%, list=30%, signal=56% |
| 1534 | LIPOPROTEIN\_METABOLIC\_PROCESS(GO:0042157) |  | 107 | 0.20 | 0.91 | 0.620 | 0.793 | 1.000 | 10057 | tags=36%, list=32%, signal=53% |
| 1535 | SULFATE\_TRANSPORT(GO:0008272) |  | 15 | 0.33 | 0.90 | 0.578 | 0.795 | 1.000 | 4677 | tags=27%, list=15%, signal=31% |
| 1536 | SULFATE\_TRANSMEMBRANE\_TRANSPORTER\_ACTIVITY(GO:0015116) |  | 15 | 0.33 | 0.90 | 0.578 | 0.794 | 1.000 | 4677 | tags=27%, list=15%, signal=31% |
| 1537 | INORGANIC\_MOLECULAR\_ENTITY\_TRANSMEMBRANE\_TRANSPORTER\_ACTIVITY(GO:0015318) |  | 780 | 0.19 | 0.90 | 0.622 | 0.795 | 1.000 | 6527 | tags=18%, list=20%, signal=22% |
| 1538 | REGULATION\_OF\_PLASMA\_MEMBRANE\_BOUNDED\_CELL\_PROJECTION\_ORGANIZATION(GO:0120035) |  | 227 | 0.19 | 0.90 | 0.560 | 0.797 | 1.000 | 3713 | tags=9%, list=12%, signal=10% |
| 1539 | LOCALIZATION\_WITHIN\_MEMBRANE(GO:0051668) |  | 63 | 0.22 | 0.90 | 0.534 | 0.797 | 1.000 | 5486 | tags=14%, list=17%, signal=17% |
| 1540 | IMPORT\_INTO\_NUCLEUS(GO:0051170) |  | 51 | 0.18 | 0.90 | 0.576 | 0.799 | 1.000 | 2896 | tags=6%, list=9%, signal=6% |
| 1541 | MORPHOGENESIS\_OF\_A\_BRANCHING\_EPITHELIUM(GO:0061138) |  | 23 | 0.29 | 0.90 | 0.602 | 0.799 | 1.000 | 1089 | tags=9%, list=3%, signal=9% |
| 1542 | DNA\_STRAND\_ELONGATION\_INVOLVED\_IN\_DNA\_REPLICATION(GO:0006271) |  | 17 | 0.33 | 0.90 | 0.506 | 0.799 | 1.000 | 11619 | tags=47%, list=36%, signal=74% |
| 1543 | RNA\_POLYMERASE\_II\_CORE\_BINDING(GO:0000993) |  | 23 | 0.18 | 0.90 | 0.665 | 0.800 | 1.000 | 8544 | tags=26%, list=27%, signal=36% |
| 1544 | FLOOR\_PLATE\_DEVELOPMENT(GO:0033504) |  | 17 | 0.28 | 0.90 | 0.643 | 0.800 | 1.000 | 8662 | tags=29%, list=27%, signal=40% |
| 1545 | PRE\_MRNA\_BINDING(GO:0036002) |  | 17 | 0.29 | 0.90 | 0.598 | 0.800 | 1.000 | 13420 | tags=65%, list=42%, signal=112% |
| 1546 | HISTONE\_LYSINE\_METHYLATION(GO:0034968) |  | 45 | 0.20 | 0.90 | 0.493 | 0.801 | 1.000 | 7114 | tags=16%, list=22%, signal=20% |
| 1547 | ANTIMICROBIAL\_HUMORAL\_RESPONSE(GO:0019730) |  | 15 | 0.30 | 0.90 | 0.684 | 0.801 | 1.000 | 6439 | tags=27%, list=20%, signal=33% |
| 1548 | ALCOHOL\_BINDING(GO:0043178) |  | 50 | 0.23 | 0.90 | 0.624 | 0.802 | 1.000 | 4688 | tags=18%, list=15%, signal=21% |
| 1549 | NEGATIVE\_REGULATION\_OF\_WNT\_SIGNALING\_PATHWAY(GO:0030178) |  | 87 | 0.21 | 0.90 | 0.653 | 0.806 | 1.000 | 7739 | tags=25%, list=24%, signal=33% |
| 1550 | PROTEIN\_TYROSINE\_SERINE\_THREONINE\_PHOSPHATASE\_ACTIVITY(GO:0008138) |  | 54 | 0.23 | 0.90 | 0.598 | 0.806 | 1.000 | 5451 | tags=20%, list=17%, signal=25% |
| 1551 | STEROID\_BINDING(GO:0005496) |  | 57 | 0.22 | 0.90 | 0.645 | 0.806 | 1.000 | 6468 | tags=23%, list=20%, signal=29% |
| 1552 | REGULATION\_OF\_CELL\_SUBSTRATE\_ADHESION(GO:0010810) |  | 22 | 0.24 | 0.89 | 0.513 | 0.808 | 1.000 | 4032 | tags=14%, list=13%, signal=16% |
| 1553 | REGULATION\_OF\_NERVOUS\_SYSTEM\_DEVELOPMENT(GO:0051960) |  | 326 | 0.20 | 0.89 | 0.597 | 0.808 | 1.000 | 3713 | tags=8%, list=12%, signal=9% |
| 1554 | LIMBIC\_SYSTEM\_DEVELOPMENT(GO:0021761) |  | 26 | 0.28 | 0.89 | 0.608 | 0.810 | 1.000 | 3530 | tags=15%, list=11%, signal=17% |
| 1555 | PROTEIN\_IMPORT\_INTO\_NUCLEUS(GO:0006606) |  | 50 | 0.18 | 0.89 | 0.558 | 0.811 | 1.000 | 2896 | tags=6%, list=9%, signal=7% |
| 1556 | FIBROBLAST\_GROWTH\_FACTOR\_RECEPTOR\_BINDING(GO:0005104) |  | 26 | 0.27 | 0.89 | 0.655 | 0.812 | 1.000 | 5163 | tags=19%, list=16%, signal=23% |
| 1557 | REGULATION\_OF\_CELL\_DIFFERENTIATION(GO:0045595) |  | 505 | 0.17 | 0.89 | 0.635 | 0.812 | 1.000 | 3838 | tags=9%, list=12%, signal=10% |
| 1558 | REGULATION\_OF\_CELL\_PROJECTION\_ORGANIZATION(GO:0031344) |  | 229 | 0.19 | 0.89 | 0.576 | 0.812 | 1.000 | 3713 | tags=9%, list=12%, signal=10% |
| 1559 | REGULATION\_OF\_RNA\_SPLICING(GO:0043484) |  | 105 | 0.16 | 0.89 | 0.656 | 0.811 | 1.000 | 8989 | tags=22%, list=28%, signal=30% |
| 1560 | CELL\_PERIPHERY(GO:0071944) |  | 3235 | 0.17 | 0.89 | 0.665 | 0.811 | 1.000 | 7337 | tags=21%, list=23%, signal=24% |
| 1561 | NEGATIVE\_REGULATION\_OF\_GENE\_EXPRESSION(GO:0010629) |  | 554 | 0.14 | 0.89 | 0.816 | 0.812 | 1.000 | 7125 | tags=16%, list=22%, signal=21% |
| 1562 | SIGNAL\_TRANSDUCER\_ACTIVITY(GO:0004871) |  | 1167 | 0.19 | 0.89 | 0.738 | 0.812 | 1.000 | 8040 | tags=25%, list=25%, signal=32% |
| 1563 | MULTI\_ORGANISM\_REPRODUCTIVE\_PROCESS(GO:0044703) |  | 180 | 0.20 | 0.89 | 0.714 | 0.813 | 1.000 | 4988 | tags=15%, list=16%, signal=18% |
| 1564 | NUCLEOTIDE\_SUGAR\_METABOLIC\_PROCESS(GO:0009225) |  | 23 | 0.29 | 0.89 | 0.779 | 0.813 | 1.000 | 9831 | tags=48%, list=31%, signal=69% |
| 1565 | RESPIRATORY\_CHAIN(GO:0070469) |  | 77 | 0.23 | 0.89 | 0.506 | 0.814 | 1.000 | 1769 | tags=4%, list=6%, signal=4% |
| 1566 | PLASMA\_MEMBRANE\_PROTEIN\_COMPLEX(GO:0098797) |  | 381 | 0.17 | 0.89 | 0.695 | 0.814 | 1.000 | 6200 | tags=17%, list=19%, signal=21% |
| 1567 | CLATHRIN\_ADAPTOR\_COMPLEX(GO:0030131) |  | 20 | 0.24 | 0.89 | 0.532 | 0.814 | 1.000 | 5189 | tags=20%, list=16%, signal=24% |
| 1568 | REGULATION\_OF\_CELLULAR\_CARBOHYDRATE\_METABOLIC\_PROCESS(GO:0010675) |  | 35 | 0.25 | 0.89 | 0.495 | 0.813 | 1.000 | 4470 | tags=23%, list=14%, signal=27% |
| 1569 | RNA\_MODIFICATION(GO:0009451) |  | 114 | 0.17 | 0.89 | 0.655 | 0.813 | 1.000 | 7638 | tags=18%, list=24%, signal=23% |
| 1570 | CELLULAR\_RESPONSE\_TO\_TOXIC\_SUBSTANCE(GO:0097237) |  | 24 | 0.26 | 0.89 | 0.605 | 0.814 | 1.000 | 148 | tags=4%, list=0%, signal=4% |
| 1571 | NEGATIVE\_REGULATION\_OF\_PROTEIN\_COMPLEX\_ASSEMBLY(GO:0031333) |  | 63 | 0.21 | 0.89 | 0.707 | 0.813 | 1.000 | 3538 | tags=13%, list=11%, signal=14% |
| 1572 | NEGATIVE\_REGULATION\_OF\_CELL\_GROWTH(GO:0030308) |  | 47 | 0.28 | 0.89 | 0.598 | 0.814 | 1.000 | 2608 | tags=9%, list=8%, signal=9% |
| 1573 | U2\_TYPE\_PRESPLICEOSOME(GO:0071004) |  | 16 | 0.26 | 0.89 | 0.650 | 0.814 | 1.000 | 2280 | tags=6%, list=7%, signal=7% |
| 1574 | PRESPLICEOSOME(GO:0071010) |  | 16 | 0.26 | 0.89 | 0.650 | 0.814 | 1.000 | 2280 | tags=6%, list=7%, signal=7% |
| 1575 | RETINA\_DEVELOPMENT\_IN\_CAMERA\_TYPE\_EYE(GO:0060041) |  | 265 | 0.15 | 0.89 | 0.638 | 0.815 | 1.000 | 7209 | tags=14%, list=23%, signal=18% |
| 1576 | DNA\_STRAND\_ELONGATION(GO:0022616) |  | 19 | 0.28 | 0.89 | 0.557 | 0.815 | 1.000 | 941 | tags=5%, list=3%, signal=5% |
| 1577 | GLYCEROPHOSPHOLIPID\_BIOSYNTHETIC\_PROCESS(GO:0046474) |  | 70 | 0.21 | 0.88 | 0.747 | 0.816 | 1.000 | 5442 | tags=16%, list=17%, signal=19% |
| 1578 | DETECTION\_OF\_LIGHT\_STIMULUS(GO:0009583) |  | 51 | 0.27 | 0.88 | 0.619 | 0.816 | 1.000 | 7936 | tags=25%, list=25%, signal=34% |
| 1579 | SENSORY\_ORGAN\_DEVELOPMENT(GO:0007423) |  | 684 | 0.15 | 0.88 | 0.676 | 0.816 | 1.000 | 7993 | tags=19%, list=25%, signal=25% |
| 1580 | PYRIMIDINE\_RIBONUCLEOTIDE\_METABOLIC\_PROCESS(GO:0009218) |  | 25 | 0.25 | 0.88 | 0.635 | 0.817 | 1.000 | 4461 | tags=16%, list=14%, signal=19% |
| 1581 | ION\_ANTIPORTER\_ACTIVITY(GO:0099516) |  | 50 | 0.25 | 0.88 | 0.634 | 0.817 | 1.000 | 6987 | tags=30%, list=22%, signal=38% |
| 1582 | PRIMITIVE\_HEMOPOIESIS(GO:0060215) |  | 60 | 0.22 | 0.88 | 0.648 | 0.818 | 1.000 | 7285 | tags=22%, list=23%, signal=28% |
| 1583 | ERYTHROCYTE\_DEVELOPMENT(GO:0048821) |  | 45 | 0.22 | 0.88 | 0.658 | 0.818 | 1.000 | 5996 | tags=20%, list=19%, signal=25% |
| 1584 | CELL\_DEVELOPMENT(GO:0048468) |  | 1453 | 0.15 | 0.88 | 0.653 | 0.819 | 1.000 | 7018 | tags=17%, list=22%, signal=20% |
| 1585 | DNA\_PACKAGING\_COMPLEX(GO:0044815) |  | 66 | 0.22 | 0.88 | 0.781 | 0.823 | 1.000 | 1209 | tags=5%, list=4%, signal=5% |
| 1586 | ANAPHASE\_PROMOTING\_COMPLEX(GO:0005680) |  | 19 | 0.29 | 0.88 | 0.601 | 0.822 | 1.000 | 5024 | tags=16%, list=16%, signal=19% |
| 1587 | ENDOTHELIAL\_CELL\_DIFFERENTIATION(GO:0045446) |  | 47 | 0.22 | 0.88 | 0.518 | 0.823 | 1.000 | 6618 | tags=21%, list=21%, signal=27% |
| 1588 | REGULATION\_OF\_INTRACELLULAR\_TRANSPORT(GO:0032386) |  | 52 | 0.22 | 0.88 | 0.587 | 0.823 | 1.000 | 4443 | tags=13%, list=14%, signal=16% |
| 1589 | EMBRYONIC\_APPENDAGE\_MORPHOGENESIS(GO:0035113) |  | 30 | 0.23 | 0.88 | 0.621 | 0.824 | 1.000 | 3713 | tags=13%, list=12%, signal=15% |
| 1590 | TRANSPORTER\_ACTIVITY(GO:0005215) |  | 1191 | 0.18 | 0.88 | 0.685 | 0.824 | 1.000 | 6576 | tags=18%, list=21%, signal=22% |
| 1591 | CATION:CATION\_ANTIPORTER\_ACTIVITY(GO:0015491) |  | 27 | 0.29 | 0.88 | 0.571 | 0.824 | 1.000 | 6987 | tags=37%, list=22%, signal=47% |
| 1592 | REGULATION\_OF\_NEURON\_PROJECTION\_DEVELOPMENT(GO:0010975) |  | 173 | 0.19 | 0.88 | 0.680 | 0.824 | 1.000 | 3713 | tags=9%, list=12%, signal=10% |
| 1593 | POSITIVE\_REGULATION\_OF\_CYTOSOLIC\_CALCIUM\_ION\_CONCENTRATION\_INVOLVED\_IN\_PHOSPHOLIPASE\_C\_ACTIVATING\_G\_PROTEIN\_COUPLED\_SIGNALING\_PATHWAY(GO:0051482) |  | 29 | 0.28 | 0.88 | 0.642 | 0.824 | 1.000 | 4374 | tags=21%, list=14%, signal=24% |
| 1594 | REGULATION\_OF\_NEUROGENESIS(GO:0050767) |  | 295 | 0.19 | 0.88 | 0.686 | 0.825 | 1.000 | 3713 | tags=8%, list=12%, signal=10% |
| 1595 | RUFFLE(GO:0001726) |  | 52 | 0.18 | 0.87 | 0.639 | 0.829 | 1.000 | 4764 | tags=13%, list=15%, signal=16% |
| 1596 | POSITIVE\_REGULATION\_OF\_MITOTIC\_CELL\_CYCLE(GO:0045931) |  | 29 | 0.24 | 0.87 | 0.603 | 0.829 | 1.000 | 7020 | tags=28%, list=22%, signal=35% |
| 1597 | DEOXYRIBONUCLEOTIDE\_METABOLIC\_PROCESS(GO:0009262) |  | 15 | 0.33 | 0.87 | 0.639 | 0.829 | 1.000 | 9136 | tags=53%, list=29%, signal=75% |
| 1598 | SH3\_DOMAIN\_BINDING(GO:0017124) |  | 36 | 0.25 | 0.87 | 0.576 | 0.829 | 1.000 | 2093 | tags=8%, list=7%, signal=9% |
| 1599 | LIPOPROTEIN\_BIOSYNTHETIC\_PROCESS(GO:0042158) |  | 80 | 0.19 | 0.87 | 0.621 | 0.829 | 1.000 | 9060 | tags=30%, list=28%, signal=42% |
| 1600 | INORGANIC\_ANION\_TRANSMEMBRANE\_TRANSPORT(GO:0098661) |  | 66 | 0.25 | 0.87 | 0.659 | 0.830 | 1.000 | 3992 | tags=11%, list=13%, signal=12% |
| 1601 | POST\_ANAL\_TAIL\_MORPHOGENESIS(GO:0036342) |  | 20 | 0.27 | 0.87 | 0.649 | 0.830 | 1.000 | 6554 | tags=25%, list=21%, signal=31% |
| 1602 | EXTRACELLULAR\_MATRIX\_STRUCTURAL\_CONSTITUENT(GO:0005201) |  | 81 | 0.25 | 0.87 | 0.586 | 0.829 | 1.000 | 4905 | tags=16%, list=15%, signal=19% |
| 1603 | KETONE\_BIOSYNTHETIC\_PROCESS(GO:0042181) |  | 24 | 0.27 | 0.87 | 0.616 | 0.830 | 1.000 | 2823 | tags=13%, list=9%, signal=14% |
| 1604 | SEXUAL\_REPRODUCTION(GO:0019953) |  | 177 | 0.20 | 0.87 | 0.707 | 0.830 | 1.000 | 4988 | tags=15%, list=16%, signal=17% |
| 1605 | UBIQUITIN\_LIKE\_PROTEIN\_CONJUGATING\_ENZYME\_ACTIVITY(GO:0061650) |  | 40 | 0.23 | 0.87 | 0.508 | 0.830 | 1.000 | 2994 | tags=8%, list=9%, signal=8% |
| 1606 | DETECTION\_OF\_MECHANICAL\_STIMULUS\_INVOLVED\_IN\_SENSORY\_PERCEPTION(GO:0050974) |  | 20 | 0.29 | 0.87 | 0.639 | 0.831 | 1.000 | 10779 | tags=50%, list=34%, signal=75% |
| 1607 | GASTRULATION\_WITH\_MOUTH\_FORMING\_SECOND(GO:0001702) |  | 58 | 0.22 | 0.87 | 0.632 | 0.831 | 1.000 | 439 | tags=3%, list=1%, signal=3% |
| 1608 | NEURON\_PROJECTION\_REGENERATION(GO:0031102) |  | 33 | 0.26 | 0.87 | 0.670 | 0.831 | 1.000 | 953 | tags=6%, list=3%, signal=6% |
| 1609 | NEGATIVE\_REGULATION\_OF\_BMP\_SIGNALING\_PATHWAY(GO:0030514) |  | 41 | 0.22 | 0.87 | 0.583 | 0.836 | 1.000 | 7427 | tags=22%, list=23%, signal=29% |
| 1610 | PROTEIN\_LIPIDATION(GO:0006497) |  | 78 | 0.19 | 0.87 | 0.645 | 0.836 | 1.000 | 9060 | tags=29%, list=28%, signal=41% |
| 1611 | REGULATION\_OF\_VASCULAR\_ENDOTHELIAL\_GROWTH\_FACTOR\_RECEPTOR\_SIGNALING\_PATHWAY(GO:0030947) |  | 17 | 0.27 | 0.87 | 0.578 | 0.837 | 1.000 | 13640 | tags=59%, list=43%, signal=103% |
| 1612 | ORGANIC\_CATION\_TRANSPORT(GO:0015695) |  | 15 | 0.29 | 0.87 | 0.676 | 0.837 | 1.000 | 858 | tags=7%, list=3%, signal=7% |
| 1613 | CELL\_CELL\_ADHESION\_VIA\_PLASMA\_MEMBRANE\_ADHESION\_MOLECULES(GO:0098742) |  | 176 | 0.19 | 0.86 | 0.845 | 0.839 | 1.000 | 7313 | tags=20%, list=23%, signal=26% |
| 1614 | RETINA\_MORPHOGENESIS\_IN\_CAMERA\_TYPE\_EYE(GO:0060042) |  | 114 | 0.17 | 0.86 | 0.714 | 0.839 | 1.000 | 4275 | tags=8%, list=13%, signal=9% |
| 1615 | PROTEIN\_ACETYLATION(GO:0006473) |  | 70 | 0.20 | 0.86 | 0.578 | 0.838 | 1.000 | 3115 | tags=7%, list=10%, signal=8% |
| 1616 | POSITIVE\_REGULATION\_OF\_SIGNALING(GO:0023056) |  | 456 | 0.16 | 0.86 | 0.811 | 0.842 | 1.000 | 7061 | tags=20%, list=22%, signal=25% |
| 1617 | REGULATION\_OF\_BLOOD\_CIRCULATION(GO:1903522) |  | 87 | 0.21 | 0.86 | 0.711 | 0.842 | 1.000 | 10375 | tags=39%, list=33%, signal=58% |
| 1618 | POSITIVE\_REGULATION\_OF\_CELL\_COMMUNICATION(GO:0010647) |  | 455 | 0.16 | 0.86 | 0.811 | 0.842 | 1.000 | 7061 | tags=20%, list=22%, signal=25% |
| 1619 | OUTWARD\_RECTIFIER\_POTASSIUM\_CHANNEL\_ACTIVITY(GO:0015271) |  | 15 | 0.29 | 0.86 | 0.691 | 0.843 | 1.000 | 8968 | tags=40%, list=28%, signal=56% |
| 1620 | REGULATION\_OF\_AXON\_GUIDANCE(GO:1902667) |  | 44 | 0.21 | 0.86 | 0.713 | 0.843 | 1.000 | 2608 | tags=7%, list=8%, signal=7% |
| 1621 | OOCYTE\_DIFFERENTIATION(GO:0009994) |  | 28 | 0.28 | 0.86 | 0.654 | 0.843 | 1.000 | 9734 | tags=36%, list=31%, signal=51% |
| 1622 | OOCYTE\_DEVELOPMENT(GO:0048599) |  | 28 | 0.28 | 0.86 | 0.654 | 0.842 | 1.000 | 9734 | tags=36%, list=31%, signal=51% |
| 1623 | SYNAPTIC\_VESICLE\_EXOCYTOSIS(GO:0016079) |  | 52 | 0.25 | 0.86 | 0.649 | 0.844 | 1.000 | 2932 | tags=10%, list=9%, signal=11% |
| 1624 | LYMPH\_VESSEL\_DEVELOPMENT(GO:0001945) |  | 69 | 0.21 | 0.86 | 0.575 | 0.844 | 1.000 | 11941 | tags=45%, list=37%, signal=72% |
| 1625 | REGENERATION(GO:0031099) |  | 192 | 0.16 | 0.86 | 0.668 | 0.843 | 1.000 | 7160 | tags=19%, list=22%, signal=25% |
| 1626 | MONOVALENT\_INORGANIC\_CATION\_TRANSMEMBRANE\_TRANSPORTER\_ACTIVITY(GO:0015077) |  | 335 | 0.18 | 0.86 | 0.674 | 0.843 | 1.000 | 6987 | tags=20%, list=22%, signal=25% |
| 1627 | VESICLE\_DOCKING\_INVOLVED\_IN\_EXOCYTOSIS(GO:0006904) |  | 27 | 0.24 | 0.86 | 0.621 | 0.844 | 1.000 | 7622 | tags=26%, list=24%, signal=34% |
| 1628 | REGULATION\_OF\_EPIDERMAL\_GROWTH\_FACTOR\_RECEPTOR\_SIGNALING\_PATHWAY(GO:0042058) |  | 21 | 0.27 | 0.86 | 0.617 | 0.847 | 1.000 | 4684 | tags=24%, list=15%, signal=28% |
| 1629 | PROTEIN\_LOCALIZATION\_TO\_SYNAPSE(GO:0035418) |  | 18 | 0.26 | 0.86 | 0.649 | 0.847 | 1.000 | 3734 | tags=11%, list=12%, signal=13% |
| 1630 | WATER\_TRANSMEMBRANE\_TRANSPORTER\_ACTIVITY(GO:0005372) |  | 15 | 0.31 | 0.86 | 0.566 | 0.847 | 1.000 | 1079 | tags=13%, list=3%, signal=14% |
| 1631 | DETECTION\_OF\_EXTERNAL\_STIMULUS(GO:0009581) |  | 81 | 0.23 | 0.85 | 0.611 | 0.848 | 1.000 | 9719 | tags=33%, list=30%, signal=48% |
| 1632 | DETECTION\_OF\_ABIOTIC\_STIMULUS(GO:0009582) |  | 81 | 0.23 | 0.85 | 0.611 | 0.847 | 1.000 | 9719 | tags=33%, list=30%, signal=48% |
| 1633 | PROTEOGLYCAN\_METABOLIC\_PROCESS(GO:0006029) |  | 59 | 0.19 | 0.85 | 0.659 | 0.847 | 1.000 | 7006 | tags=22%, list=22%, signal=28% |
| 1634 | HYDRO\_LYASE\_ACTIVITY(GO:0016836) |  | 55 | 0.23 | 0.85 | 0.513 | 0.847 | 1.000 | 4565 | tags=16%, list=14%, signal=19% |
| 1635 | ACETYLCHOLINE\_GATED\_CATION\_SELECTIVE\_CHANNEL\_ACTIVITY(GO:0022848) |  | 23 | 0.29 | 0.85 | 0.746 | 0.847 | 1.000 | 81 | tags=4%, list=0%, signal=4% |
| 1636 | REGULATION\_OF\_NUCLEOTIDE\_BIOSYNTHETIC\_PROCESS(GO:0030808) |  | 19 | 0.26 | 0.85 | 0.782 | 0.849 | 1.000 | 8023 | tags=37%, list=25%, signal=49% |
| 1637 | REGULATION\_OF\_PURINE\_NUCLEOTIDE\_BIOSYNTHETIC\_PROCESS(GO:1900371) |  | 19 | 0.26 | 0.85 | 0.782 | 0.848 | 1.000 | 8023 | tags=37%, list=25%, signal=49% |
| 1638 | SYNAPSE\_ORGANIZATION(GO:0050808) |  | 139 | 0.21 | 0.85 | 0.649 | 0.848 | 1.000 | 1563 | tags=5%, list=5%, signal=5% |
| 1639 | COLLAGEN\_BINDING(GO:0005518) |  | 23 | 0.23 | 0.85 | 0.597 | 0.848 | 1.000 | 4464 | tags=17%, list=14%, signal=20% |
| 1640 | PRONEPHRIC\_GLOMERULUS\_DEVELOPMENT(GO:0039021) |  | 26 | 0.23 | 0.85 | 0.854 | 0.848 | 1.000 | 5463 | tags=15%, list=17%, signal=19% |
| 1641 | CHROMOSOME\_\_TELOMERIC\_REGION(GO:0000781) |  | 23 | 0.25 | 0.85 | 0.635 | 0.849 | 1.000 | 10948 | tags=43%, list=34%, signal=66% |
| 1642 | SECONDARY\_ACTIVE\_MONOCARBOXYLATE\_TRANSMEMBRANE\_TRANSPORTER\_ACTIVITY(GO:0015355) |  | 17 | 0.31 | 0.85 | 0.612 | 0.850 | 1.000 | 5262 | tags=18%, list=16%, signal=21% |
| 1643 | SOMATIC\_DIVERSIFICATION\_OF\_IMMUNE\_RECEPTORS\_VIA\_GERMLINE\_RECOMBINATION\_WITHIN\_A\_SINGLE\_LOCUS(GO:0002562) |  | 15 | 0.32 | 0.85 | 0.593 | 0.849 | 1.000 | 12436 | tags=67%, list=39%, signal=109% |
| 1644 | SOMATIC\_CELL\_DNA\_RECOMBINATION(GO:0016444) |  | 15 | 0.32 | 0.85 | 0.593 | 0.849 | 1.000 | 12436 | tags=67%, list=39%, signal=109% |
| 1645 | EMBRYONIC\_CAMERA\_TYPE\_EYE\_DEVELOPMENT(GO:0031076) |  | 48 | 0.22 | 0.85 | 0.692 | 0.849 | 1.000 | 3761 | tags=10%, list=12%, signal=12% |
| 1646 | PROTEIN\_TRANSPORT\_WITHIN\_PLASMA\_MEMBRANE(GO:0099632) |  | 22 | 0.28 | 0.85 | 0.727 | 0.851 | 1.000 | 4426 | tags=18%, list=14%, signal=21% |
| 1647 | NEUROTRANSMITTER\_RECEPTOR\_TRANSPORT(GO:0099637) |  | 22 | 0.28 | 0.85 | 0.727 | 0.850 | 1.000 | 4426 | tags=18%, list=14%, signal=21% |
| 1648 | PROTEIN\_IMPORT(GO:0017038) |  | 89 | 0.15 | 0.85 | 0.569 | 0.850 | 1.000 | 3926 | tags=8%, list=12%, signal=9% |
| 1649 | NEGATIVE\_REGULATION\_OF\_TRANSFERASE\_ACTIVITY(GO:0051348) |  | 58 | 0.20 | 0.85 | 0.648 | 0.852 | 1.000 | 11069 | tags=40%, list=35%, signal=61% |
| 1650 | G\_PROTEIN\_COUPLED\_PHOTORECEPTOR\_ACTIVITY(GO:0008020) |  | 34 | 0.29 | 0.85 | 0.687 | 0.852 | 1.000 | 7936 | tags=32%, list=25%, signal=43% |
| 1651 | CENTRAL\_NERVOUS\_SYSTEM\_DEVELOPMENT(GO:0007417) |  | 662 | 0.16 | 0.85 | 0.695 | 0.854 | 1.000 | 7848 | tags=19%, list=25%, signal=25% |
| 1652 | REGULATION\_OF\_EXTENT\_OF\_CELL\_GROWTH(GO:0061387) |  | 82 | 0.20 | 0.85 | 0.626 | 0.853 | 1.000 | 7916 | tags=23%, list=25%, signal=31% |
| 1653 | TRNA\_WOBBLE\_URIDINE\_MODIFICATION(GO:0002098) |  | 15 | 0.27 | 0.85 | 0.575 | 0.853 | 1.000 | 23148 | tags=100%, list=73%, signal=365% |
| 1654 | PYRIMIDINE\_RIBONUCLEOTIDE\_BIOSYNTHETIC\_PROCESS(GO:0009220) |  | 24 | 0.24 | 0.84 | 0.677 | 0.856 | 1.000 | 1678 | tags=8%, list=5%, signal=9% |
| 1655 | PYRIMIDINE\_RIBONUCLEOSIDE\_BIOSYNTHETIC\_PROCESS(GO:0046132) |  | 24 | 0.24 | 0.84 | 0.677 | 0.855 | 1.000 | 1678 | tags=8%, list=5%, signal=9% |
| 1656 | PYRIMIDINE\_NUCLEOSIDE\_BIOSYNTHETIC\_PROCESS(GO:0046134) |  | 24 | 0.24 | 0.84 | 0.677 | 0.855 | 1.000 | 1678 | tags=8%, list=5%, signal=9% |
| 1657 | POSITIVE\_REGULATION\_OF\_NEUROGENESIS(GO:0050769) |  | 101 | 0.19 | 0.84 | 0.643 | 0.855 | 1.000 | 3491 | tags=9%, list=11%, signal=10% |
| 1658 | CANONICAL\_WNT\_SIGNALING\_PATHWAY(GO:0060070) |  | 174 | 0.18 | 0.84 | 0.665 | 0.857 | 1.000 | 7624 | tags=21%, list=24%, signal=27% |
| 1659 | CONNEXIN\_COMPLEX(GO:0005922) |  | 32 | 0.27 | 0.84 | 0.689 | 0.857 | 1.000 | 10308 | tags=44%, list=32%, signal=65% |
| 1660 | NEURAL\_RETINA\_DEVELOPMENT(GO:0003407) |  | 99 | 0.18 | 0.84 | 0.674 | 0.857 | 1.000 | 5170 | tags=10%, list=16%, signal=12% |
| 1661 | ENDONUCLEASE\_COMPLEX(GO:1905348) |  | 17 | 0.31 | 0.84 | 0.631 | 0.858 | 1.000 | 5016 | tags=24%, list=16%, signal=28% |
| 1662 | DETECTION\_OF\_STIMULUS(GO:0051606) |  | 151 | 0.22 | 0.84 | 0.744 | 0.858 | 1.000 | 10219 | tags=35%, list=32%, signal=51% |
| 1663 | PURINE\_RIBONUCLEOTIDE\_CATABOLIC\_PROCESS(GO:0009154) |  | 18 | 0.29 | 0.84 | 0.690 | 0.859 | 1.000 | 9805 | tags=39%, list=31%, signal=56% |
| 1664 | PERIPHERAL\_NERVOUS\_SYSTEM\_DEVELOPMENT(GO:0007422) |  | 68 | 0.18 | 0.84 | 0.746 | 0.860 | 1.000 | 3581 | tags=9%, list=11%, signal=10% |
| 1665 | PEPTIDE\_BINDING(GO:0042277) |  | 120 | 0.17 | 0.84 | 0.676 | 0.862 | 1.000 | 10588 | tags=37%, list=33%, signal=55% |
| 1666 | RNA\_METHYLTRANSFERASE\_ACTIVITY(GO:0008173) |  | 53 | 0.20 | 0.84 | 0.733 | 0.862 | 1.000 | 1840 | tags=4%, list=6%, signal=4% |
| 1667 | SISTER\_CHROMATID\_SEGREGATION(GO:0000819) |  | 97 | 0.18 | 0.84 | 0.519 | 0.863 | 1.000 | 8402 | tags=25%, list=26%, signal=33% |
| 1668 | NEGATIVE\_REGULATION\_OF\_CELL\_FATE\_COMMITMENT(GO:0010454) |  | 15 | 0.28 | 0.84 | 0.654 | 0.864 | 1.000 | 888 | tags=7%, list=3%, signal=7% |
| 1669 | PROTEIN\_KINASE\_BINDING(GO:0019901) |  | 146 | 0.15 | 0.84 | 0.740 | 0.864 | 1.000 | 7575 | tags=22%, list=24%, signal=29% |
| 1670 | CALCIUM\_ION\_REGULATED\_EXOCYTOSIS(GO:0017156) |  | 77 | 0.24 | 0.84 | 0.770 | 0.864 | 1.000 | 2932 | tags=9%, list=9%, signal=10% |
| 1671 | RESPONSE\_TO\_AMINO\_ACID(GO:0043200) |  | 26 | 0.25 | 0.84 | 0.725 | 0.864 | 1.000 | 4510 | tags=15%, list=14%, signal=18% |
| 1672 | REGULATION\_OF\_ERBB\_SIGNALING\_PATHWAY(GO:1901184) |  | 22 | 0.27 | 0.83 | 0.710 | 0.864 | 1.000 | 4684 | tags=23%, list=15%, signal=27% |
| 1673 | TRANSMEMBRANE\_TRANSPORTER\_ACTIVITY(GO:0022857) |  | 1114 | 0.17 | 0.83 | 0.796 | 0.864 | 1.000 | 6576 | tags=18%, list=21%, signal=22% |
| 1674 | LATE\_ENDOSOME\_TO\_VACUOLE\_TRANSPORT\_VIA\_MULTIVESICULAR\_BODY\_SORTING\_PATHWAY(GO:0032511) |  | 16 | 0.23 | 0.83 | 0.640 | 0.866 | 1.000 | 2217 | tags=6%, list=7%, signal=7% |
| 1675 | AXONAL\_TRANSPORT(GO:0098930) |  | 22 | 0.23 | 0.83 | 0.666 | 0.867 | 1.000 | 6854 | tags=27%, list=21%, signal=35% |
| 1676 | ADENYLATE\_CYCLASE\_MODULATING\_G\_PROTEIN\_COUPLED\_RECEPTOR\_SIGNALING\_PATHWAY(GO:0007188) |  | 159 | 0.21 | 0.83 | 0.745 | 0.868 | 1.000 | 5401 | tags=18%, list=17%, signal=21% |
| 1677 | MIDBRAIN\_HINDBRAIN\_BOUNDARY\_DEVELOPMENT(GO:0030917) |  | 40 | 0.21 | 0.83 | 0.783 | 0.869 | 1.000 | 4244 | tags=13%, list=13%, signal=14% |
| 1678 | GENE\_SILENCING(GO:0016458) |  | 99 | 0.19 | 0.83 | 0.802 | 0.870 | 1.000 | 6084 | tags=17%, list=19%, signal=21% |
| 1679 | REGULATION\_OF\_MULTICELLULAR\_ORGANISMAL\_DEVELOPMENT(GO:2000026) |  | 698 | 0.15 | 0.83 | 0.727 | 0.871 | 1.000 | 7931 | tags=19%, list=25%, signal=25% |
| 1680 | POSITIVE\_REGULATION\_OF\_FERTILIZATION(GO:1905516) |  | 15 | 0.31 | 0.83 | 0.798 | 0.871 | 1.000 | 4988 | tags=27%, list=16%, signal=32% |
| 1681 | AXON\_REGENERATION(GO:0031103) |  | 28 | 0.27 | 0.83 | 0.708 | 0.872 | 1.000 | 953 | tags=7%, list=3%, signal=7% |
| 1682 | AXON\_EXTENSION\_INVOLVED\_IN\_AXON\_GUIDANCE(GO:0048846) |  | 44 | 0.22 | 0.83 | 0.795 | 0.872 | 1.000 | 11535 | tags=43%, list=36%, signal=68% |
| 1683 | NEURON\_PROJECTION\_EXTENSION\_INVOLVED\_IN\_NEURON\_PROJECTION\_GUIDANCE(GO:1902284) |  | 44 | 0.22 | 0.83 | 0.795 | 0.871 | 1.000 | 11535 | tags=43%, list=36%, signal=68% |
| 1684 | PROTEIN\_IMPORT\_INTO\_MITOCHONDRIAL\_MATRIX(GO:0030150) |  | 19 | 0.26 | 0.83 | 0.650 | 0.873 | 1.000 | 3926 | tags=16%, list=12%, signal=18% |
| 1685 | PEPTIDE\_CATABOLIC\_PROCESS(GO:0043171) |  | 20 | 0.32 | 0.82 | 0.632 | 0.877 | 1.000 | 7082 | tags=30%, list=22%, signal=39% |
| 1686 | MAP\_KINASE\_PHOSPHATASE\_ACTIVITY(GO:0033549) |  | 21 | 0.29 | 0.82 | 0.714 | 0.877 | 1.000 | 4422 | tags=24%, list=14%, signal=28% |
| 1687 | AUTONOMIC\_NERVOUS\_SYSTEM\_DEVELOPMENT(GO:0048483) |  | 52 | 0.19 | 0.82 | 0.654 | 0.877 | 1.000 | 8662 | tags=27%, list=27%, signal=37% |
| 1688 | EMBRYONIC\_ORGAN\_DEVELOPMENT(GO:0048568) |  | 587 | 0.14 | 0.82 | 0.861 | 0.877 | 1.000 | 6941 | tags=15%, list=22%, signal=19% |
| 1689 | ANION:ANION\_ANTIPORTER\_ACTIVITY(GO:0015301) |  | 23 | 0.27 | 0.82 | 0.628 | 0.876 | 1.000 | 8626 | tags=35%, list=27%, signal=48% |
| 1690 | INNER\_MITOCHONDRIAL\_MEMBRANE\_PROTEIN\_COMPLEX(GO:0098800) |  | 118 | 0.20 | 0.82 | 0.650 | 0.877 | 1.000 | 3926 | tags=9%, list=12%, signal=11% |
| 1691 | NEURAL\_TUBE\_PATTERNING(GO:0021532) |  | 55 | 0.18 | 0.82 | 0.715 | 0.877 | 1.000 | 4244 | tags=11%, list=13%, signal=13% |
| 1692 | PROTEIN\_BINDING\_INVOLVED\_IN\_PROTEIN\_FOLDING(GO:0044183) |  | 16 | 0.28 | 0.82 | 0.651 | 0.879 | 1.000 | 2952 | tags=19%, list=9%, signal=21% |
| 1693 | ION\_TRANSPORT(GO:0006811) |  | 1066 | 0.17 | 0.82 | 0.796 | 0.879 | 1.000 | 6527 | tags=18%, list=20%, signal=22% |
| 1694 | OXIDOREDUCTASE\_ACTIVITY\_\_ACTING\_ON\_PAIRED\_DONORS\_\_WITH\_INCORPORATION\_OR\_REDUCTION\_OF\_MOLECULAR\_OXYGEN\_\_NAD(P)H\_AS\_ONE\_DONOR\_\_AND\_INCORPORATION\_OF\_ONE\_ATOM\_OF\_OXYGEN(GO:0016709) |  | 31 | 0.25 | 0.82 | 0.753 | 0.880 | 1.000 | 6660 | tags=26%, list=21%, signal=33% |
| 1695 | REGULATED\_EXOCYTOSIS(GO:0045055) |  | 89 | 0.22 | 0.82 | 0.775 | 0.880 | 1.000 | 3394 | tags=9%, list=11%, signal=10% |
| 1696 | HOMOPHILIC\_CELL\_ADHESION\_VIA\_PLASMA\_MEMBRANE\_ADHESION\_MOLECULES(GO:0007156) |  | 148 | 0.19 | 0.82 | 0.928 | 0.880 | 1.000 | 7313 | tags=22%, list=23%, signal=28% |
| 1697 | DENDRITE\_DEVELOPMENT(GO:0016358) |  | 54 | 0.21 | 0.82 | 0.756 | 0.880 | 1.000 | 4687 | tags=13%, list=15%, signal=15% |
| 1698 | POSITIVE\_REGULATION\_OF\_PROTEIN\_KINASE\_ACTIVITY(GO:0045860) |  | 47 | 0.20 | 0.82 | 0.875 | 0.880 | 1.000 | 8693 | tags=36%, list=27%, signal=50% |
| 1699 | AMINOPEPTIDASE\_ACTIVITY(GO:0004177) |  | 36 | 0.24 | 0.82 | 0.685 | 0.880 | 1.000 | 156 | tags=3%, list=0%, signal=3% |
| 1700 | SCAVENGER\_RECEPTOR\_ACTIVITY(GO:0005044) |  | 40 | 0.25 | 0.82 | 0.701 | 0.881 | 1.000 | 5517 | tags=28%, list=17%, signal=33% |
| 1701 | NEURON\_FATE\_COMMITMENT(GO:0048663) |  | 40 | 0.26 | 0.82 | 0.667 | 0.883 | 1.000 | 8662 | tags=33%, list=27%, signal=45% |
| 1702 | NEGATIVE\_REGULATION\_OF\_KINASE\_ACTIVITY(GO:0033673) |  | 54 | 0.19 | 0.82 | 0.700 | 0.883 | 1.000 | 11069 | tags=39%, list=35%, signal=59% |
| 1703 | PROTON\_TRANSPORTING\_TWO\_SECTOR\_ATPASE\_COMPLEX\_\_CATALYTIC\_DOMAIN(GO:0033178) |  | 21 | 0.29 | 0.82 | 0.692 | 0.883 | 1.000 | 2778 | tags=10%, list=9%, signal=10% |
| 1704 | CELL\_CYCLE\_G2\_M\_PHASE\_TRANSITION(GO:0044839) |  | 40 | 0.20 | 0.82 | 0.686 | 0.883 | 1.000 | 4217 | tags=15%, list=13%, signal=17% |
| 1705 | REGULATION\_OF\_CALCIUM\_ION\_DEPENDENT\_EXOCYTOSIS(GO:0017158) |  | 38 | 0.26 | 0.82 | 0.731 | 0.882 | 1.000 | 2932 | tags=11%, list=9%, signal=12% |
| 1706 | GLUCURONOSYLTRANSFERASE\_ACTIVITY(GO:0015020) |  | 40 | 0.30 | 0.81 | 0.679 | 0.884 | 1.000 | 8092 | tags=33%, list=25%, signal=43% |
| 1707 | REGULATION\_OF\_FERTILIZATION(GO:0080154) |  | 16 | 0.31 | 0.81 | 0.857 | 0.883 | 1.000 | 4988 | tags=25%, list=16%, signal=30% |
| 1708 | VASOCONSTRICTION(GO:0042310) |  | 36 | 0.26 | 0.81 | 0.779 | 0.886 | 1.000 | 10070 | tags=44%, list=32%, signal=65% |
| 1709 | NEGATIVE\_REGULATION\_OF\_BLOOD\_VESSEL\_DIAMETER(GO:0097756) |  | 36 | 0.26 | 0.81 | 0.779 | 0.885 | 1.000 | 10070 | tags=44%, list=32%, signal=65% |
| 1710 | CELL\_MATURATION(GO:0048469) |  | 66 | 0.19 | 0.81 | 0.793 | 0.888 | 1.000 | 9734 | tags=33%, list=31%, signal=48% |
| 1711 | SENSORY\_PERCEPTION\_OF\_CHEMICAL\_STIMULUS(GO:0007606) |  | 81 | 0.23 | 0.81 | 0.758 | 0.889 | 1.000 | 4590 | tags=17%, list=14%, signal=20% |
| 1712 | PEROXISOME(GO:0005777) |  | 83 | 0.18 | 0.81 | 0.709 | 0.889 | 1.000 | 9617 | tags=36%, list=30%, signal=52% |
| 1713 | MICROBODY(GO:0042579) |  | 83 | 0.18 | 0.81 | 0.709 | 0.888 | 1.000 | 9617 | tags=36%, list=30%, signal=52% |
| 1714 | ION\_TRANSMEMBRANE\_TRANSPORTER\_ACTIVITY(GO:0015075) |  | 825 | 0.17 | 0.81 | 0.828 | 0.888 | 1.000 | 6576 | tags=17%, list=21%, signal=21% |
| 1715 | MITOCHONDRIAL\_MEMBRANE\_PART(GO:0044455) |  | 187 | 0.18 | 0.81 | 0.640 | 0.888 | 1.000 | 3242 | tags=8%, list=10%, signal=9% |
| 1716 | GLUTAMATE\_METABOLIC\_PROCESS(GO:0006536) |  | 17 | 0.28 | 0.81 | 0.696 | 0.888 | 1.000 | 2628 | tags=12%, list=8%, signal=13% |
| 1717 | NEGATIVE\_REGULATION\_OF\_PROTEIN\_KINASE\_ACTIVITY(GO:0006469) |  | 51 | 0.19 | 0.81 | 0.694 | 0.888 | 1.000 | 11069 | tags=39%, list=35%, signal=60% |
| 1718 | REGULATION\_OF\_LEUKOCYTE\_MEDIATED\_IMMUNITY(GO:0002703) |  | 16 | 0.27 | 0.81 | 0.686 | 0.888 | 1.000 | 9019 | tags=44%, list=28%, signal=61% |
| 1719 | CELLULAR\_PROCESS\_INVOLVED\_IN\_REPRODUCTION\_IN\_MULTICELLULAR\_ORGANISM(GO:0022412) |  | 107 | 0.20 | 0.81 | 0.872 | 0.890 | 1.000 | 9804 | tags=33%, list=31%, signal=47% |
| 1720 | REGULATION\_OF\_ENDOTHELIAL\_CELL\_PROLIFERATION(GO:0001936) |  | 19 | 0.25 | 0.81 | 0.644 | 0.892 | 1.000 | 9254 | tags=37%, list=29%, signal=52% |
| 1721 | CHLORIDE\_CHANNEL\_ACTIVITY(GO:0005254) |  | 63 | 0.23 | 0.80 | 0.637 | 0.892 | 1.000 | 3752 | tags=11%, list=12%, signal=13% |
| 1722 | NOTOCHORD\_DEVELOPMENT(GO:0030903) |  | 67 | 0.18 | 0.80 | 0.686 | 0.893 | 1.000 | 6270 | tags=15%, list=20%, signal=19% |
| 1723 | ENDOPLASMIC\_RETICULUM\_GOLGI\_INTERMEDIATE\_COMPARTMENT(GO:0005793) |  | 43 | 0.23 | 0.80 | 0.699 | 0.892 | 1.000 | 9157 | tags=35%, list=29%, signal=49% |
| 1724 | ANION\_HOMEOSTASIS(GO:0055081) |  | 34 | 0.24 | 0.80 | 0.772 | 0.893 | 1.000 | 4545 | tags=18%, list=14%, signal=21% |
| 1725 | NUCLEAR\_DNA\_REPLICATION(GO:0033260) |  | 16 | 0.29 | 0.80 | 0.746 | 0.893 | 1.000 | 855 | tags=6%, list=3%, signal=6% |
| 1726 | DETECTION\_OF\_MECHANICAL\_STIMULUS(GO:0050982) |  | 27 | 0.23 | 0.80 | 0.742 | 0.895 | 1.000 | 10779 | tags=48%, list=34%, signal=73% |
| 1727 | VENTRAL\_SPINAL\_CORD\_INTERNEURON\_DIFFERENTIATION(GO:0021514) |  | 24 | 0.26 | 0.80 | 0.810 | 0.895 | 1.000 | 8662 | tags=29%, list=27%, signal=40% |
| 1728 | SYNAPSIS(GO:0007129) |  | 18 | 0.27 | 0.80 | 0.757 | 0.895 | 1.000 | 3321 | tags=11%, list=10%, signal=12% |
| 1729 | U1\_SNRNP(GO:0005685) |  | 17 | 0.24 | 0.80 | 0.728 | 0.894 | 1.000 | 9999 | tags=24%, list=31%, signal=34% |
| 1730 | GENE\_SILENCING\_BY\_RNA(GO:0031047) |  | 88 | 0.19 | 0.80 | 0.842 | 0.895 | 1.000 | 6084 | tags=18%, list=19%, signal=22% |
| 1731 | MRNA\_3'\_UTR\_BINDING(GO:0003730) |  | 58 | 0.16 | 0.80 | 0.818 | 0.895 | 1.000 | 6833 | tags=17%, list=21%, signal=22% |
| 1732 | RESPONSE\_TO\_AXON\_INJURY(GO:0048678) |  | 30 | 0.26 | 0.80 | 0.757 | 0.896 | 1.000 | 953 | tags=7%, list=3%, signal=7% |
| 1733 | PECTORAL\_FIN\_DEVELOPMENT(GO:0033339) |  | 63 | 0.20 | 0.80 | 0.724 | 0.896 | 1.000 | 3713 | tags=11%, list=12%, signal=13% |
| 1734 | REGULATION\_OF\_BMP\_SIGNALING\_PATHWAY(GO:0030510) |  | 81 | 0.17 | 0.80 | 0.778 | 0.897 | 1.000 | 9244 | tags=27%, list=29%, signal=38% |
| 1735 | POSITIVE\_REGULATION\_OF\_CYTOSKELETON\_ORGANIZATION(GO:0051495) |  | 98 | 0.16 | 0.80 | 0.841 | 0.897 | 1.000 | 6848 | tags=18%, list=21%, signal=23% |
| 1736 | NEUROTRANSMITTER\_SECRETION(GO:0007269) |  | 72 | 0.22 | 0.80 | 0.754 | 0.897 | 1.000 | 2932 | tags=8%, list=9%, signal=9% |
| 1737 | SIGNAL\_RELEASE\_FROM\_SYNAPSE(GO:0099643) |  | 72 | 0.22 | 0.80 | 0.754 | 0.897 | 1.000 | 2932 | tags=8%, list=9%, signal=9% |
| 1738 | RESPONSE\_TO\_ZINC\_ION(GO:0010043) |  | 15 | 0.28 | 0.80 | 0.794 | 0.896 | 1.000 | 3765 | tags=13%, list=12%, signal=15% |
| 1739 | POSITIVE\_REGULATION\_OF\_RAS\_PROTEIN\_SIGNAL\_TRANSDUCTION(GO:0046579) |  | 33 | 0.24 | 0.80 | 0.807 | 0.896 | 1.000 | 4374 | tags=21%, list=14%, signal=25% |
| 1740 | POSITIVE\_REGULATION\_OF\_SMALL\_GTPASE\_MEDIATED\_SIGNAL\_TRANSDUCTION(GO:0051057) |  | 33 | 0.24 | 0.80 | 0.807 | 0.895 | 1.000 | 4374 | tags=21%, list=14%, signal=25% |
| 1741 | CHLORIDE\_TRANSMEMBRANE\_TRANSPORTER\_ACTIVITY(GO:0015108) |  | 89 | 0.21 | 0.80 | 0.778 | 0.897 | 1.000 | 6400 | tags=18%, list=20%, signal=22% |
| 1742 | CILIUM\_OR\_FLAGELLUM\_DEPENDENT\_CELL\_MOTILITY(GO:0001539) |  | 19 | 0.25 | 0.80 | 0.747 | 0.897 | 1.000 | 6134 | tags=21%, list=19%, signal=26% |
| 1743 | CILIUM\_DEPENDENT\_CELL\_MOTILITY(GO:0060285) |  | 19 | 0.25 | 0.80 | 0.747 | 0.896 | 1.000 | 6134 | tags=21%, list=19%, signal=26% |
| 1744 | MICROTUBULE(GO:0005874) |  | 238 | 0.15 | 0.79 | 0.884 | 0.897 | 1.000 | 7859 | tags=18%, list=25%, signal=24% |
| 1745 | NUCLEOSOME\_POSITIONING(GO:0016584) |  | 18 | 0.24 | 0.79 | 0.783 | 0.896 | 1.000 | 792 | tags=6%, list=2%, signal=6% |
| 1746 | NEUROTRANSMITTER\_BINDING(GO:0042165) |  | 24 | 0.32 | 0.79 | 0.714 | 0.896 | 1.000 | 8330 | tags=33%, list=26%, signal=45% |
| 1747 | SPLICEOSOMAL\_SNRNP\_ASSEMBLY(GO:0000387) |  | 23 | 0.23 | 0.79 | 0.631 | 0.896 | 1.000 | 7096 | tags=17%, list=22%, signal=22% |
| 1748 | G\_PROTEIN\_COUPLED\_RECEPTOR\_SIGNALING\_PATHWAY\_\_COUPLED\_TO\_CYCLIC\_NUCLEOTIDE\_SECOND\_MESSENGER(GO:0007187) |  | 182 | 0.20 | 0.79 | 0.862 | 0.896 | 1.000 | 5401 | tags=18%, list=17%, signal=21% |
| 1749 | METAL\_ION\_TRANSMEMBRANE\_TRANSPORTER\_ACTIVITY(GO:0046873) |  | 392 | 0.17 | 0.79 | 0.831 | 0.898 | 1.000 | 6527 | tags=19%, list=20%, signal=23% |
| 1750 | NCRNA\_TRANSCRIPTION(GO:0098781) |  | 18 | 0.24 | 0.79 | 0.706 | 0.898 | 1.000 | 6939 | tags=22%, list=22%, signal=28% |
| 1751 | CELL\_JUNCTION(GO:0030054) |  | 564 | 0.15 | 0.79 | 0.864 | 0.898 | 1.000 | 6576 | tags=15%, list=21%, signal=19% |
| 1752 | NUCLEAR\_LOCALIZATION\_SEQUENCE\_BINDING(GO:0008139) |  | 15 | 0.25 | 0.79 | 0.642 | 0.901 | 1.000 | 13495 | tags=53%, list=42%, signal=92% |
| 1753 | POSTSYNAPTIC\_SPECIALIZATION\_MEMBRANE(GO:0099634) |  | 42 | 0.25 | 0.79 | 0.701 | 0.901 | 1.000 | 1043 | tags=5%, list=3%, signal=5% |
| 1754 | EMBRYONIC\_PECTORAL\_FIN\_MORPHOGENESIS(GO:0035118) |  | 24 | 0.22 | 0.79 | 0.746 | 0.901 | 1.000 | 3713 | tags=13%, list=12%, signal=14% |
| 1755 | REGULATION\_OF\_SYNAPSE\_STRUCTURE\_OR\_ACTIVITY(GO:0050803) |  | 44 | 0.23 | 0.79 | 0.783 | 0.901 | 1.000 | 953 | tags=5%, list=3%, signal=5% |
| 1756 | LYMPHANGIOGENESIS(GO:0001946) |  | 46 | 0.21 | 0.79 | 0.712 | 0.901 | 1.000 | 12247 | tags=48%, list=38%, signal=78% |
| 1757 | FRIZZLED\_BINDING(GO:0005109) |  | 28 | 0.22 | 0.79 | 0.764 | 0.901 | 1.000 | 7041 | tags=25%, list=22%, signal=32% |
| 1758 | MITOTIC\_SISTER\_CHROMATID\_SEGREGATION(GO:0000070) |  | 81 | 0.17 | 0.79 | 0.710 | 0.902 | 1.000 | 8402 | tags=22%, list=26%, signal=30% |
| 1759 | INORGANIC\_CATION\_TRANSMEMBRANE\_TRANSPORTER\_ACTIVITY(GO:0022890) |  | 551 | 0.16 | 0.79 | 0.913 | 0.902 | 1.000 | 6527 | tags=17%, list=20%, signal=22% |
| 1760 | O\_ACYLTRANSFERASE\_ACTIVITY(GO:0008374) |  | 40 | 0.20 | 0.79 | 0.635 | 0.901 | 1.000 | 11876 | tags=45%, list=37%, signal=72% |
| 1761 | MESODERM\_DEVELOPMENT(GO:0007498) |  | 98 | 0.17 | 0.79 | 0.917 | 0.903 | 1.000 | 4882 | tags=10%, list=15%, signal=12% |
| 1762 | NEGATIVE\_REGULATION\_OF\_ION\_TRANSMEMBRANE\_TRANSPORT(GO:0034766) |  | 17 | 0.29 | 0.78 | 0.717 | 0.904 | 1.000 | 9315 | tags=41%, list=29%, signal=58% |
| 1763 | NEGATIVE\_REGULATION\_OF\_CATION\_TRANSMEMBRANE\_TRANSPORT(GO:1904063) |  | 17 | 0.29 | 0.78 | 0.717 | 0.903 | 1.000 | 9315 | tags=41%, list=29%, signal=58% |
| 1764 | CARDIAC\_MUSCLE\_TISSUE\_MORPHOGENESIS(GO:0055008) |  | 16 | 0.27 | 0.78 | 0.692 | 0.903 | 1.000 | 888 | tags=6%, list=3%, signal=6% |
| 1765 | REGULATION\_OF\_SIGNAL\_TRANSDUCTION\_BY\_P53\_CLASS\_MEDIATOR(GO:1901796) |  | 16 | 0.26 | 0.78 | 0.694 | 0.903 | 1.000 | 2107 | tags=13%, list=7%, signal=13% |
| 1766 | SIGNALING\_RECEPTOR\_ACTIVITY(GO:0038023) |  | 1023 | 0.17 | 0.78 | 0.898 | 0.905 | 1.000 | 8040 | tags=24%, list=25%, signal=32% |
| 1767 | MOLECULAR\_TRANSDUCER\_ACTIVITY(GO:0060089) |  | 1023 | 0.17 | 0.78 | 0.898 | 0.904 | 1.000 | 8040 | tags=24%, list=25%, signal=32% |
| 1768 | GLUTAMINE\_METABOLIC\_PROCESS(GO:0006541) |  | 20 | 0.24 | 0.78 | 0.751 | 0.904 | 1.000 | 5012 | tags=25%, list=16%, signal=30% |
| 1769 | POSITIVE\_REGULATION\_OF\_MITOTIC\_NUCLEAR\_DIVISION(GO:0045840) |  | 16 | 0.21 | 0.78 | 0.802 | 0.904 | 1.000 | 13112 | tags=63%, list=41%, signal=106% |
| 1770 | POSITIVE\_REGULATION\_OF\_NUCLEAR\_DIVISION(GO:0051785) |  | 16 | 0.21 | 0.78 | 0.802 | 0.904 | 1.000 | 13112 | tags=63%, list=41%, signal=106% |
| 1771 | SEMAPHORIN\_RECEPTOR\_BINDING(GO:0030215) |  | 33 | 0.23 | 0.78 | 0.726 | 0.905 | 1.000 | 2608 | tags=9%, list=8%, signal=10% |
| 1772 | CALMODULIN\_BINDING(GO:0005516) |  | 110 | 0.18 | 0.78 | 0.651 | 0.906 | 1.000 | 7206 | tags=20%, list=23%, signal=26% |
| 1773 | G\_PROTEIN\_COUPLED\_RECEPTOR\_ACTIVITY(GO:0004930) |  | 563 | 0.18 | 0.78 | 0.916 | 0.906 | 1.000 | 8036 | tags=26%, list=25%, signal=34% |
| 1774 | PERIKARYON(GO:0043204) |  | 30 | 0.20 | 0.78 | 0.793 | 0.906 | 1.000 | 8709 | tags=37%, list=27%, signal=50% |
| 1775 | PRESYNAPTIC\_PROCESS\_INVOLVED\_IN\_CHEMICAL\_SYNAPTIC\_TRANSMISSION(GO:0099531) |  | 74 | 0.22 | 0.78 | 0.812 | 0.905 | 1.000 | 2932 | tags=8%, list=9%, signal=9% |
| 1776 | MICROTUBULE\_END(GO:1990752) |  | 20 | 0.22 | 0.78 | 0.888 | 0.908 | 1.000 | 8022 | tags=25%, list=25%, signal=33% |
| 1777 | CELL\_SURFACE\_RECEPTOR\_SIGNALING\_PATHWAY\_INVOLVED\_IN\_CELL\_CELL\_SIGNALING(GO:1905114) |  | 276 | 0.15 | 0.78 | 0.747 | 0.909 | 1.000 | 7739 | tags=20%, list=24%, signal=26% |
| 1778 | ENDOTHELIAL\_CELL\_MIGRATION(GO:0043542) |  | 48 | 0.21 | 0.77 | 0.755 | 0.911 | 1.000 | 7020 | tags=23%, list=22%, signal=29% |
| 1779 | SULFUR\_COMPOUND\_TRANSMEMBRANE\_TRANSPORTER\_ACTIVITY(GO:1901682) |  | 28 | 0.25 | 0.77 | 0.739 | 0.914 | 1.000 | 9216 | tags=39%, list=29%, signal=55% |
| 1780 | TRANSMEMBRANE\_SIGNALING\_RECEPTOR\_ACTIVITY(GO:0004888) |  | 913 | 0.17 | 0.77 | 0.952 | 0.914 | 1.000 | 8040 | tags=24%, list=25%, signal=32% |
| 1781 | RESPONSE\_TO\_HYDROGEN\_PEROXIDE(GO:0042542) |  | 15 | 0.26 | 0.77 | 0.713 | 0.916 | 1.000 | 9766 | tags=60%, list=31%, signal=86% |
| 1782 | DNA\_ALKYLATION(GO:0006305) |  | 23 | 0.22 | 0.77 | 0.810 | 0.918 | 1.000 | 7114 | tags=26%, list=22%, signal=34% |
| 1783 | DNA\_METHYLATION(GO:0006306) |  | 23 | 0.22 | 0.77 | 0.810 | 0.917 | 1.000 | 7114 | tags=26%, list=22%, signal=34% |
| 1784 | PHOSPHORIC\_DIESTER\_HYDROLASE\_ACTIVITY(GO:0008081) |  | 92 | 0.18 | 0.77 | 0.833 | 0.917 | 1.000 | 7073 | tags=23%, list=22%, signal=29% |
| 1785 | REGULATION\_OF\_REGULATED\_SECRETORY\_PATHWAY(GO:1903305) |  | 43 | 0.23 | 0.77 | 0.754 | 0.918 | 1.000 | 2932 | tags=9%, list=9%, signal=10% |
| 1786 | POSITIVE\_REGULATION\_OF\_SUPRAMOLECULAR\_FIBER\_ORGANIZATION(GO:1902905) |  | 95 | 0.16 | 0.77 | 0.891 | 0.918 | 1.000 | 6848 | tags=18%, list=21%, signal=23% |
| 1787 | HISTONE\_ACETYLATION(GO:0016573) |  | 56 | 0.19 | 0.77 | 0.632 | 0.917 | 1.000 | 3115 | tags=7%, list=10%, signal=8% |
| 1788 | HINDBRAIN\_DEVELOPMENT(GO:0030902) |  | 127 | 0.15 | 0.77 | 0.706 | 0.917 | 1.000 | 8784 | tags=22%, list=28%, signal=30% |
| 1789 | PROTON\_TRANSMEMBRANE\_TRANSPORT(GO:1902600) |  | 80 | 0.17 | 0.77 | 0.784 | 0.917 | 1.000 | 7161 | tags=20%, list=22%, signal=26% |
| 1790 | PURINERGIC\_NUCLEOTIDE\_RECEPTOR\_ACTIVITY(GO:0001614) |  | 17 | 0.28 | 0.77 | 0.770 | 0.917 | 1.000 | 652 | tags=6%, list=2%, signal=6% |
| 1791 | NUCLEOTIDE\_RECEPTOR\_ACTIVITY(GO:0016502) |  | 17 | 0.28 | 0.77 | 0.770 | 0.916 | 1.000 | 652 | tags=6%, list=2%, signal=6% |
| 1792 | LIGAND\_GATED\_ANION\_CHANNEL\_ACTIVITY(GO:0099095) |  | 17 | 0.33 | 0.77 | 0.718 | 0.917 | 1.000 | 6131 | tags=24%, list=19%, signal=29% |
| 1793 | NEUROGENESIS(GO:0022008) |  | 1109 | 0.14 | 0.77 | 0.809 | 0.916 | 1.000 | 7981 | tags=20%, list=25%, signal=25% |
| 1794 | DIGESTIVE\_SYSTEM\_DEVELOPMENT(GO:0055123) |  | 133 | 0.14 | 0.76 | 0.767 | 0.917 | 1.000 | 3529 | tags=8%, list=11%, signal=9% |
| 1795 | SMOOTHENED\_SIGNALING\_PATHWAY(GO:0007224) |  | 73 | 0.16 | 0.76 | 0.783 | 0.917 | 1.000 | 10080 | tags=30%, list=32%, signal=44% |
| 1796 | ENERGY\_COUPLED\_PROTON\_TRANSPORT\_\_DOWN\_ELECTROCHEMICAL\_GRADIENT(GO:0015985) |  | 25 | 0.23 | 0.76 | 0.701 | 0.917 | 1.000 | 7852 | tags=24%, list=25%, signal=32% |
| 1797 | ATP\_SYNTHESIS\_COUPLED\_PROTON\_TRANSPORT(GO:0015986) |  | 25 | 0.23 | 0.76 | 0.701 | 0.917 | 1.000 | 7852 | tags=24%, list=25%, signal=32% |
| 1798 | REGULATION\_OF\_CANONICAL\_WNT\_SIGNALING\_PATHWAY(GO:0060828) |  | 118 | 0.18 | 0.76 | 0.754 | 0.917 | 1.000 | 7624 | tags=20%, list=24%, signal=27% |
| 1799 | ACTIN\_MONOMER\_BINDING(GO:0003785) |  | 17 | 0.27 | 0.76 | 0.749 | 0.917 | 1.000 | 4764 | tags=24%, list=15%, signal=28% |
| 1800 | PIGMENT\_BIOSYNTHETIC\_PROCESS(GO:0046148) |  | 38 | 0.22 | 0.76 | 0.828 | 0.917 | 1.000 | 4639 | tags=11%, list=15%, signal=12% |
| 1801 | PROTEIN\_LOCALIZATION\_TO\_LYSOSOME(GO:0061462) |  | 27 | 0.22 | 0.76 | 0.804 | 0.920 | 1.000 | 5789 | tags=15%, list=18%, signal=18% |
| 1802 | RESPONSE\_TO\_REACTIVE\_OXYGEN\_SPECIES(GO:0000302) |  | 30 | 0.20 | 0.76 | 0.600 | 0.920 | 1.000 | 9766 | tags=37%, list=31%, signal=53% |
| 1803 | INTRACELLULAR\_LIGAND\_GATED\_ION\_CHANNEL\_ACTIVITY(GO:0005217) |  | 22 | 0.26 | 0.76 | 0.804 | 0.920 | 1.000 | 11736 | tags=55%, list=37%, signal=86% |
| 1804 | ION\_CHANNEL\_BINDING(GO:0044325) |  | 37 | 0.21 | 0.75 | 0.772 | 0.925 | 1.000 | 5917 | tags=19%, list=19%, signal=23% |
| 1805 | SIGNAL\_TRANSDUCER\_\_DOWNSTREAM\_OF\_RECEPTOR\_\_WITH\_SERINE\_THREONINE\_KINASE\_ACTIVITY(GO:0004702) |  | 42 | 0.20 | 0.75 | 0.804 | 0.926 | 1.000 | 7276 | tags=21%, list=23%, signal=28% |
| 1806 | SIGNAL\_TRANSDUCER\_ACTIVITY\_\_DOWNSTREAM\_OF\_RECEPTOR(GO:0005057) |  | 42 | 0.20 | 0.75 | 0.804 | 0.926 | 1.000 | 7276 | tags=21%, list=23%, signal=28% |
| 1807 | VOLTAGE\_GATED\_POTASSIUM\_CHANNEL\_ACTIVITY(GO:0005249) |  | 100 | 0.19 | 0.75 | 0.948 | 0.926 | 1.000 | 6527 | tags=21%, list=20%, signal=26% |
| 1808 | POTASSIUM\_CHANNEL\_ACTIVITY(GO:0005267) |  | 127 | 0.18 | 0.75 | 0.929 | 0.926 | 1.000 | 7900 | tags=25%, list=25%, signal=33% |
| 1809 | PYRIMIDINE\_NUCLEOTIDE\_METABOLIC\_PROCESS(GO:0006220) |  | 31 | 0.22 | 0.75 | 0.842 | 0.926 | 1.000 | 8427 | tags=29%, list=26%, signal=39% |
| 1810 | CATION\_TRANSMEMBRANE\_TRANSPORTER\_ACTIVITY(GO:0008324) |  | 602 | 0.15 | 0.75 | 0.914 | 0.927 | 1.000 | 6527 | tags=17%, list=20%, signal=21% |
| 1811 | SPINAL\_CORD\_MOTOR\_NEURON\_DIFFERENTIATION(GO:0021522) |  | 30 | 0.26 | 0.75 | 0.805 | 0.927 | 1.000 | 6936 | tags=23%, list=22%, signal=30% |
| 1812 | POSITIVE\_REGULATION\_OF\_NERVOUS\_SYSTEM\_DEVELOPMENT(GO:0051962) |  | 111 | 0.18 | 0.75 | 0.770 | 0.928 | 1.000 | 3491 | tags=8%, list=11%, signal=9% |
| 1813 | NEGATIVE\_REGULATION\_OF\_DNA\_RECOMBINATION(GO:0045910) |  | 28 | 0.20 | 0.75 | 0.795 | 0.928 | 1.000 | 792 | tags=4%, list=2%, signal=4% |
| 1814 | CELLULAR\_RESPONSE\_TO\_LIGHT\_STIMULUS(GO:0071482) |  | 49 | 0.21 | 0.75 | 0.865 | 0.928 | 1.000 | 7936 | tags=27%, list=25%, signal=35% |
| 1815 | CARDIAC\_VENTRICLE\_DEVELOPMENT(GO:0003231) |  | 26 | 0.24 | 0.75 | 0.759 | 0.930 | 1.000 | 1347 | tags=8%, list=4%, signal=8% |
| 1816 | PROTEIN\_DNA\_COMPLEX(GO:0032993) |  | 98 | 0.15 | 0.75 | 0.956 | 0.931 | 1.000 | 4047 | tags=9%, list=13%, signal=10% |
| 1817 | MAP\_KINASE\_TYROSINE\_SERINE\_THREONINE\_PHOSPHATASE\_ACTIVITY(GO:0017017) |  | 15 | 0.29 | 0.75 | 0.787 | 0.930 | 1.000 | 4422 | tags=27%, list=14%, signal=31% |
| 1818 | MITOCHONDRIAL\_INTERMEMBRANE\_SPACE(GO:0005758) |  | 35 | 0.19 | 0.75 | 0.758 | 0.930 | 1.000 | 8006 | tags=26%, list=25%, signal=34% |
| 1819 | ORGANELLE\_ENVELOPE\_LUMEN(GO:0031970) |  | 35 | 0.19 | 0.75 | 0.758 | 0.930 | 1.000 | 8006 | tags=26%, list=25%, signal=34% |
| 1820 | VENTRAL\_SPINAL\_CORD\_DEVELOPMENT(GO:0021517) |  | 54 | 0.21 | 0.74 | 0.758 | 0.931 | 1.000 | 8662 | tags=26%, list=27%, signal=36% |
| 1821 | ENDOCRINE\_PROCESS(GO:0050886) |  | 17 | 0.25 | 0.74 | 0.780 | 0.931 | 1.000 | 10070 | tags=47%, list=32%, signal=69% |
| 1822 | EPHRIN\_RECEPTOR\_SIGNALING\_PATHWAY(GO:0048013) |  | 18 | 0.26 | 0.74 | 0.774 | 0.931 | 1.000 | 7745 | tags=22%, list=24%, signal=29% |
| 1823 | PYRIMIDINE\_NUCLEOTIDE\_BIOSYNTHETIC\_PROCESS(GO:0006221) |  | 29 | 0.21 | 0.74 | 0.742 | 0.931 | 1.000 | 8427 | tags=28%, list=26%, signal=37% |
| 1824 | NEURAL\_PLATE\_PATTERN\_SPECIFICATION(GO:0060896) |  | 17 | 0.27 | 0.74 | 0.759 | 0.930 | 1.000 | 10482 | tags=29%, list=33%, signal=44% |
| 1825 | TRNA\_WOBBLE\_BASE\_MODIFICATION(GO:0002097) |  | 17 | 0.24 | 0.74 | 0.658 | 0.930 | 1.000 | 24229 | tags=100%, list=76%, signal=416% |
| 1826 | CHROMATIN\_ASSEMBLY(GO:0031497) |  | 65 | 0.20 | 0.74 | 0.858 | 0.930 | 1.000 | 941 | tags=5%, list=3%, signal=5% |
| 1827 | LIGAND\_GATED\_CATION\_CHANNEL\_ACTIVITY(GO:0099094) |  | 108 | 0.20 | 0.74 | 0.874 | 0.930 | 1.000 | 4087 | tags=11%, list=13%, signal=13% |
| 1828 | EYE\_DEVELOPMENT(GO:0001654) |  | 513 | 0.14 | 0.74 | 0.905 | 0.930 | 1.000 | 7281 | tags=16%, list=23%, signal=20% |
| 1829 | RECEPTOR\_INTERNALIZATION(GO:0031623) |  | 33 | 0.24 | 0.74 | 0.759 | 0.930 | 1.000 | 6320 | tags=18%, list=20%, signal=23% |
| 1830 | DEMETHYLATION(GO:0070988) |  | 34 | 0.20 | 0.74 | 0.730 | 0.930 | 1.000 | 10574 | tags=35%, list=33%, signal=53% |
| 1831 | NEURAL\_PLATE\_REGIONALIZATION(GO:0060897) |  | 16 | 0.27 | 0.74 | 0.759 | 0.933 | 1.000 | 10482 | tags=31%, list=33%, signal=47% |
| 1832 | BRANCHED\_CHAIN\_AMINO\_ACID\_METABOLIC\_PROCESS(GO:0009081) |  | 17 | 0.23 | 0.74 | 0.658 | 0.932 | 1.000 | 5080 | tags=18%, list=16%, signal=21% |
| 1833 | MOTILE\_CILIUM(GO:0031514) |  | 50 | 0.21 | 0.74 | 0.770 | 0.932 | 1.000 | 5208 | tags=16%, list=16%, signal=19% |
| 1834 | TRANSMITTER\_GATED\_ION\_CHANNEL\_ACTIVITY(GO:0022824) |  | 68 | 0.23 | 0.74 | 0.820 | 0.932 | 1.000 | 9450 | tags=31%, list=30%, signal=44% |
| 1835 | TRANSMITTER\_GATED\_CHANNEL\_ACTIVITY(GO:0022835) |  | 68 | 0.23 | 0.74 | 0.820 | 0.932 | 1.000 | 9450 | tags=31%, list=30%, signal=44% |
| 1836 | LYMPH\_VESSEL\_MORPHOGENESIS(GO:0036303) |  | 50 | 0.19 | 0.73 | 0.736 | 0.934 | 1.000 | 11587 | tags=42%, list=36%, signal=66% |
| 1837 | WNT\_SIGNALING\_PATHWAY(GO:0016055) |  | 266 | 0.14 | 0.73 | 0.823 | 0.935 | 1.000 | 7739 | tags=19%, list=24%, signal=25% |
| 1838 | CELL\_CELL\_SIGNALING\_BY\_WNT(GO:0198738) |  | 266 | 0.14 | 0.73 | 0.823 | 0.935 | 1.000 | 7739 | tags=19%, list=24%, signal=25% |
| 1839 | INTERNAL\_PROTEIN\_AMINO\_ACID\_ACETYLATION(GO:0006475) |  | 58 | 0.18 | 0.73 | 0.744 | 0.938 | 1.000 | 3115 | tags=7%, list=10%, signal=8% |
| 1840 | INTERNAL\_PEPTIDYL\_LYSINE\_ACETYLATION(GO:0018393) |  | 58 | 0.18 | 0.73 | 0.744 | 0.937 | 1.000 | 3115 | tags=7%, list=10%, signal=8% |
| 1841 | PALMITOYL\_(PROTEIN)\_HYDROLASE\_ACTIVITY(GO:0008474) |  | 20 | 0.21 | 0.73 | 0.953 | 0.940 | 1.000 | 8468 | tags=30%, list=27%, signal=41% |
| 1842 | PALMITOYL\_HYDROLASE\_ACTIVITY(GO:0098599) |  | 20 | 0.21 | 0.73 | 0.953 | 0.939 | 1.000 | 8468 | tags=30%, list=27%, signal=41% |
| 1843 | CHANNEL\_ACTIVITY(GO:0015267) |  | 469 | 0.16 | 0.73 | 0.878 | 0.939 | 1.000 | 7525 | tags=22%, list=24%, signal=28% |
| 1844 | PASSIVE\_TRANSMEMBRANE\_TRANSPORTER\_ACTIVITY(GO:0022803) |  | 469 | 0.16 | 0.73 | 0.878 | 0.939 | 1.000 | 7525 | tags=22%, list=24%, signal=28% |
| 1845 | ANION\_CHANNEL\_ACTIVITY(GO:0005253) |  | 69 | 0.20 | 0.73 | 0.740 | 0.938 | 1.000 | 3752 | tags=10%, list=12%, signal=11% |
| 1846 | PROTEIN\_K63\_LINKED\_DEUBIQUITINATION(GO:0070536) |  | 19 | 0.23 | 0.73 | 0.765 | 0.938 | 1.000 | 6935 | tags=21%, list=22%, signal=27% |
| 1847 | NEURON\_MIGRATION(GO:0001764) |  | 44 | 0.20 | 0.73 | 0.779 | 0.939 | 1.000 | 184 | tags=2%, list=1%, signal=2% |
| 1848 | REGULATION\_OF\_CELL\_CYCLE\_G2\_M\_PHASE\_TRANSITION(GO:1902749) |  | 32 | 0.20 | 0.73 | 0.757 | 0.940 | 1.000 | 6892 | tags=22%, list=22%, signal=28% |
| 1849 | MACROMOLECULE\_METHYLATION(GO:0043414) |  | 148 | 0.13 | 0.72 | 0.875 | 0.943 | 1.000 | 4474 | tags=8%, list=14%, signal=9% |
| 1850 | PEPTIDYL\_LYSINE\_ACETYLATION(GO:0018394) |  | 59 | 0.18 | 0.72 | 0.744 | 0.943 | 1.000 | 3115 | tags=7%, list=10%, signal=7% |
| 1851 | RRNA\_BINDING(GO:0019843) |  | 38 | 0.20 | 0.72 | 0.727 | 0.945 | 1.000 | 637 | tags=3%, list=2%, signal=3% |
| 1852 | PRONEPHRIC\_NEPHRON\_DEVELOPMENT(GO:0039019) |  | 41 | 0.19 | 0.72 | 0.829 | 0.945 | 1.000 | 5623 | tags=17%, list=18%, signal=21% |
| 1853 | EMBRYONIC\_HEMOPOIESIS(GO:0035162) |  | 91 | 0.16 | 0.72 | 0.951 | 0.945 | 1.000 | 6815 | tags=19%, list=21%, signal=24% |
| 1854 | BRAIN\_DEVELOPMENT(GO:0007420) |  | 462 | 0.13 | 0.72 | 0.830 | 0.946 | 1.000 | 5811 | tags=12%, list=18%, signal=14% |
| 1855 | ATP\_DEPENDENT\_MICROTUBULE\_MOTOR\_ACTIVITY\_\_PLUS\_END\_DIRECTED(GO:0008574) |  | 16 | 0.19 | 0.72 | 0.761 | 0.947 | 1.000 | 6005 | tags=19%, list=19%, signal=23% |
| 1856 | ARTERY\_MORPHOGENESIS(GO:0048844) |  | 19 | 0.21 | 0.72 | 0.919 | 0.947 | 1.000 | 6651 | tags=21%, list=21%, signal=27% |
| 1857 | ATRIOVENTRICULAR\_CANAL\_DEVELOPMENT(GO:0036302) |  | 17 | 0.23 | 0.72 | 0.862 | 0.947 | 1.000 | 5757 | tags=18%, list=18%, signal=22% |
| 1858 | CELLULAR\_RESPONSE\_TO\_VASCULAR\_ENDOTHELIAL\_GROWTH\_FACTOR\_STIMULUS(GO:0035924) |  | 26 | 0.21 | 0.71 | 0.803 | 0.948 | 1.000 | 11941 | tags=50%, list=37%, signal=80% |
| 1859 | HEAD\_DEVELOPMENT(GO:0060322) |  | 480 | 0.13 | 0.71 | 0.852 | 0.950 | 1.000 | 5811 | tags=11%, list=18%, signal=14% |
| 1860 | ION\_GATED\_CHANNEL\_ACTIVITY(GO:0022839) |  | 331 | 0.17 | 0.71 | 0.947 | 0.950 | 1.000 | 6576 | tags=18%, list=21%, signal=22% |
| 1861 | MYELINATION(GO:0042552) |  | 69 | 0.15 | 0.71 | 0.917 | 0.952 | 1.000 | 8021 | tags=23%, list=25%, signal=31% |
| 1862 | NEGATIVE\_REGULATION\_OF\_CANONICAL\_WNT\_SIGNALING\_PATHWAY(GO:0090090) |  | 59 | 0.19 | 0.71 | 0.808 | 0.951 | 1.000 | 7624 | tags=22%, list=24%, signal=29% |
| 1863 | REGULATION\_OF\_CELL\_SIZE(GO:0008361) |  | 104 | 0.17 | 0.71 | 0.894 | 0.951 | 1.000 | 8709 | tags=24%, list=27%, signal=33% |
| 1864 | GAMETE\_GENERATION(GO:0007276) |  | 152 | 0.16 | 0.71 | 0.956 | 0.952 | 1.000 | 7160 | tags=20%, list=22%, signal=25% |
| 1865 | REGULATION\_OF\_DENDRITE\_MORPHOGENESIS(GO:0048814) |  | 19 | 0.25 | 0.71 | 0.845 | 0.952 | 1.000 | 9563 | tags=37%, list=30%, signal=53% |
| 1866 | MULTICELLULAR\_ORGANISMAL\_REPRODUCTIVE\_PROCESS(GO:0048609) |  | 155 | 0.16 | 0.71 | 0.956 | 0.952 | 1.000 | 7160 | tags=19%, list=22%, signal=25% |
| 1867 | NUCLEAR\_ENVELOPE\_ORGANIZATION(GO:0006998) |  | 21 | 0.22 | 0.70 | 0.727 | 0.955 | 1.000 | 6426 | tags=14%, list=20%, signal=18% |
| 1868 | VISUAL\_PERCEPTION(GO:0007601) |  | 150 | 0.20 | 0.70 | 0.889 | 0.955 | 1.000 | 8071 | tags=23%, list=25%, signal=31% |
| 1869 | PHOTOTRANSDUCTION(GO:0007602) |  | 41 | 0.22 | 0.70 | 0.892 | 0.956 | 1.000 | 7936 | tags=29%, list=25%, signal=39% |
| 1870 | PROTEIN\_K48\_LINKED\_UBIQUITINATION(GO:0070936) |  | 24 | 0.16 | 0.70 | 0.795 | 0.956 | 1.000 | 26852 | tags=100%, list=84%, signal=632% |
| 1871 | POSITIVE\_REGULATION\_OF\_INTRACELLULAR\_TRANSPORT(GO:0032388) |  | 19 | 0.23 | 0.70 | 0.790 | 0.956 | 1.000 | 6384 | tags=21%, list=20%, signal=26% |
| 1872 | SENSORY\_EPITHELIUM\_REGENERATION(GO:0070654) |  | 20 | 0.22 | 0.70 | 0.837 | 0.956 | 1.000 | 7125 | tags=25%, list=22%, signal=32% |
| 1873 | EPITHELIUM\_REGENERATION(GO:1990399) |  | 20 | 0.22 | 0.70 | 0.837 | 0.955 | 1.000 | 7125 | tags=25%, list=22%, signal=32% |
| 1874 | MULTICELLULAR\_ORGANISM\_REPRODUCTION(GO:0032504) |  | 156 | 0.15 | 0.70 | 0.956 | 0.955 | 1.000 | 7160 | tags=19%, list=22%, signal=25% |
| 1875 | SEGMENTATION(GO:0035282) |  | 108 | 0.13 | 0.70 | 0.978 | 0.957 | 1.000 | 10984 | tags=33%, list=34%, signal=51% |
| 1876 | GAP\_JUNCTION(GO:0005921) |  | 39 | 0.22 | 0.70 | 0.811 | 0.956 | 1.000 | 10308 | tags=41%, list=32%, signal=61% |
| 1877 | ECTODERMAL\_PLACODE\_FORMATION(GO:0060788) |  | 26 | 0.20 | 0.70 | 0.806 | 0.956 | 1.000 | 8798 | tags=27%, list=28%, signal=37% |
| 1878 | SUBSTRATE\_SPECIFIC\_CHANNEL\_ACTIVITY(GO:0022838) |  | 419 | 0.16 | 0.70 | 0.946 | 0.956 | 1.000 | 7525 | tags=21%, list=24%, signal=27% |
| 1879 | REGULATION\_OF\_BLOOD\_VESSEL\_DIAMETER(GO:0097746) |  | 53 | 0.20 | 0.70 | 0.905 | 0.956 | 1.000 | 10070 | tags=36%, list=32%, signal=52% |
| 1880 | REGULATION\_OF\_TUBE\_SIZE(GO:0035150) |  | 53 | 0.20 | 0.70 | 0.905 | 0.955 | 1.000 | 10070 | tags=36%, list=32%, signal=52% |
| 1881 | REGULATION\_OF\_TUBE\_DIAMETER(GO:0035296) |  | 53 | 0.20 | 0.70 | 0.905 | 0.955 | 1.000 | 10070 | tags=36%, list=32%, signal=52% |
| 1882 | REGULATION\_OF\_BLOOD\_VESSEL\_SIZE(GO:0050880) |  | 53 | 0.20 | 0.70 | 0.905 | 0.954 | 1.000 | 10070 | tags=36%, list=32%, signal=52% |
| 1883 | REGULATION\_OF\_DOPAMINE\_SECRETION(GO:0014059) |  | 24 | 0.23 | 0.69 | 0.864 | 0.954 | 1.000 | 500 | tags=4%, list=2%, signal=4% |
| 1884 | DOPAMINE\_SECRETION(GO:0014046) |  | 24 | 0.23 | 0.69 | 0.864 | 0.954 | 1.000 | 500 | tags=4%, list=2%, signal=4% |
| 1885 | CENTRAL\_NERVOUS\_SYSTEM\_NEURON\_DIFFERENTIATION(GO:0021953) |  | 114 | 0.16 | 0.69 | 0.896 | 0.954 | 1.000 | 8830 | tags=29%, list=28%, signal=40% |
| 1886 | PHOTORECEPTOR\_ACTIVITY(GO:0009881) |  | 39 | 0.22 | 0.69 | 0.886 | 0.955 | 1.000 | 7936 | tags=28%, list=25%, signal=38% |
| 1887 | CATION\_CHANNEL\_COMPLEX(GO:0034703) |  | 148 | 0.16 | 0.69 | 0.911 | 0.955 | 1.000 | 6142 | tags=16%, list=19%, signal=20% |
| 1888 | SOMITOGENESIS(GO:0001756) |  | 92 | 0.13 | 0.69 | 0.951 | 0.955 | 1.000 | 10984 | tags=34%, list=34%, signal=51% |
| 1889 | CHLORIDE\_TRANSPORT(GO:0006821) |  | 86 | 0.19 | 0.69 | 0.905 | 0.955 | 1.000 | 6400 | tags=16%, list=20%, signal=20% |
| 1890 | DOPAMINE\_TRANSPORT(GO:0015872) |  | 26 | 0.22 | 0.69 | 0.833 | 0.956 | 1.000 | 500 | tags=4%, list=2%, signal=4% |
| 1891 | THYROID\_GLAND\_DEVELOPMENT(GO:0030878) |  | 17 | 0.25 | 0.69 | 0.789 | 0.955 | 1.000 | 8736 | tags=35%, list=27%, signal=49% |
| 1892 | DETECTION\_OF\_CHEMICAL\_STIMULUS\_INVOLVED\_IN\_SENSORY\_PERCEPTION(GO:0050907) |  | 62 | 0.21 | 0.69 | 0.892 | 0.955 | 1.000 | 5799 | tags=19%, list=18%, signal=24% |
| 1893 | OLIGODENDROCYTE\_DIFFERENTIATION(GO:0048709) |  | 53 | 0.18 | 0.69 | 0.858 | 0.955 | 1.000 | 8146 | tags=25%, list=26%, signal=33% |
| 1894 | EYE\_PHOTORECEPTOR\_CELL\_DEVELOPMENT(GO:0042462) |  | 40 | 0.22 | 0.69 | 0.770 | 0.956 | 1.000 | 6934 | tags=20%, list=22%, signal=26% |
| 1895 | PROTEIN\_SERINE\_THREONINE\_KINASE\_ACTIVATOR\_ACTIVITY(GO:0043539) |  | 27 | 0.20 | 0.69 | 0.814 | 0.956 | 1.000 | 10725 | tags=48%, list=34%, signal=72% |
| 1896 | POSITIVE\_REGULATION\_OF\_AXON\_EXTENSION(GO:0045773) |  | 15 | 0.20 | 0.69 | 0.895 | 0.956 | 1.000 | 8709 | tags=33%, list=27%, signal=46% |
| 1897 | NEURON\_FATE\_SPECIFICATION(GO:0048665) |  | 28 | 0.24 | 0.69 | 0.890 | 0.956 | 1.000 | 8662 | tags=32%, list=27%, signal=44% |
| 1898 | PURINERGIC\_RECEPTOR\_ACTIVITY(GO:0035586) |  | 23 | 0.22 | 0.68 | 0.902 | 0.960 | 1.000 | 652 | tags=4%, list=2%, signal=4% |
| 1899 | DETECTION\_OF\_CHEMICAL\_STIMULUS(GO:0009593) |  | 64 | 0.20 | 0.68 | 0.942 | 0.960 | 1.000 | 5799 | tags=19%, list=18%, signal=23% |
| 1900 | HISTONE\_MONOUBIQUITINATION(GO:0010390) |  | 15 | 0.23 | 0.68 | 0.900 | 0.961 | 1.000 | 10971 | tags=33%, list=34%, signal=51% |
| 1901 | PRIMITIVE\_ERYTHROCYTE\_DIFFERENTIATION(GO:0060319) |  | 23 | 0.22 | 0.68 | 0.729 | 0.961 | 1.000 | 740 | tags=4%, list=2%, signal=4% |
| 1902 | GATED\_CHANNEL\_ACTIVITY(GO:0022836) |  | 343 | 0.16 | 0.68 | 0.948 | 0.961 | 1.000 | 6576 | tags=17%, list=21%, signal=22% |
| 1903 | REGULATION\_OF\_MUSCLE\_ORGAN\_DEVELOPMENT(GO:0048634) |  | 26 | 0.22 | 0.68 | 0.847 | 0.960 | 1.000 | 10309 | tags=38%, list=32%, signal=57% |
| 1904 | SYNAPTIC\_MEMBRANE(GO:0097060) |  | 204 | 0.19 | 0.68 | 0.883 | 0.960 | 1.000 | 7548 | tags=19%, list=24%, signal=24% |
| 1905 | AMINO\_ACID\_BINDING(GO:0016597) |  | 24 | 0.22 | 0.68 | 0.893 | 0.960 | 1.000 | 3179 | tags=8%, list=10%, signal=9% |
| 1906 | PRESYNAPSE(GO:0098793) |  | 192 | 0.16 | 0.67 | 0.937 | 0.963 | 1.000 | 7519 | tags=18%, list=24%, signal=23% |
| 1907 | CATECHOLAMINE\_SECRETION(GO:0050432) |  | 26 | 0.22 | 0.67 | 0.860 | 0.963 | 1.000 | 500 | tags=4%, list=2%, signal=4% |
| 1908 | REGULATION\_OF\_CATECHOLAMINE\_SECRETION(GO:0050433) |  | 26 | 0.22 | 0.67 | 0.860 | 0.963 | 1.000 | 500 | tags=4%, list=2%, signal=4% |
| 1909 | G\_PROTEIN\_COUPLED\_SEROTONIN\_RECEPTOR\_ACTIVITY(GO:0004993) |  | 39 | 0.23 | 0.67 | 0.958 | 0.963 | 1.000 | 6258 | tags=28%, list=20%, signal=35% |
| 1910 | SEROTONIN\_RECEPTOR\_ACTIVITY(GO:0099589) |  | 39 | 0.23 | 0.67 | 0.958 | 0.963 | 1.000 | 6258 | tags=28%, list=20%, signal=35% |
| 1911 | SODIUM\_CHANNEL\_COMPLEX(GO:0034706) |  | 21 | 0.20 | 0.67 | 0.926 | 0.963 | 1.000 | 3663 | tags=14%, list=11%, signal=16% |
| 1912 | CAMERA\_TYPE\_EYE\_DEVELOPMENT(GO:0043010) |  | 441 | 0.13 | 0.67 | 0.941 | 0.963 | 1.000 | 7281 | tags=16%, list=23%, signal=20% |
| 1913 | POSITIVE\_REGULATION\_OF\_CANONICAL\_WNT\_SIGNALING\_PATHWAY(GO:0090263) |  | 41 | 0.17 | 0.67 | 0.868 | 0.964 | 1.000 | 6954 | tags=20%, list=22%, signal=25% |
| 1914 | VOLTAGE\_GATED\_SODIUM\_CHANNEL\_COMPLEX(GO:0001518) |  | 19 | 0.22 | 0.67 | 0.839 | 0.964 | 1.000 | 3663 | tags=16%, list=11%, signal=18% |
| 1915 | CELL\_FATE\_COMMITMENT(GO:0045165) |  | 172 | 0.14 | 0.66 | 0.924 | 0.965 | 1.000 | 10020 | tags=30%, list=31%, signal=43% |
| 1916 | ANTERIOR\_POSTERIOR\_PATTERN\_SPECIFICATION(GO:0009952) |  | 227 | 0.12 | 0.66 | 0.938 | 0.965 | 1.000 | 4468 | tags=9%, list=14%, signal=10% |
| 1917 | REGULATION\_OF\_CELL\_FATE\_SPECIFICATION(GO:0042659) |  | 21 | 0.21 | 0.66 | 0.916 | 0.965 | 1.000 | 888 | tags=5%, list=3%, signal=5% |
| 1918 | ION\_CHANNEL\_ACTIVITY(GO:0005216) |  | 405 | 0.15 | 0.66 | 0.946 | 0.965 | 1.000 | 7525 | tags=20%, list=24%, signal=26% |
| 1919 | GAP\_JUNCTION\_CHANNEL\_ACTIVITY(GO:0005243) |  | 34 | 0.21 | 0.66 | 0.921 | 0.965 | 1.000 | 6141 | tags=24%, list=19%, signal=29% |
| 1920 | ECTODERMAL\_PLACODE\_MORPHOGENESIS(GO:0071697) |  | 31 | 0.17 | 0.66 | 0.879 | 0.965 | 1.000 | 8933 | tags=29%, list=28%, signal=40% |
| 1921 | NEGATIVE\_REGULATION\_OF\_PROTEIN\_SERINE\_THREONINE\_KINASE\_ACTIVITY(GO:0071901) |  | 25 | 0.20 | 0.66 | 0.857 | 0.965 | 1.000 | 4879 | tags=16%, list=15%, signal=19% |
| 1922 | NEUROTRANSMITTER\_RECEPTOR\_ACTIVITY\_INVOLVED\_IN\_REGULATION\_OF\_POSTSYNAPTIC\_MEMBRANE\_POTENTIAL(GO:0099529) |  | 40 | 0.22 | 0.66 | 0.927 | 0.965 | 1.000 | 81 | tags=3%, list=0%, signal=3% |
| 1923 | TRANSMITTER\_GATED\_ION\_CHANNEL\_ACTIVITY\_INVOLVED\_IN\_REGULATION\_OF\_POSTSYNAPTIC\_MEMBRANE\_POTENTIAL(GO:1904315) |  | 40 | 0.22 | 0.66 | 0.927 | 0.965 | 1.000 | 81 | tags=3%, list=0%, signal=3% |
| 1924 | PHOSPHATIDYLINOSITOL\_3\_PHOSPHATASE\_ACTIVITY(GO:0004438) |  | 17 | 0.19 | 0.66 | 0.924 | 0.964 | 1.000 | 4918 | tags=12%, list=15%, signal=14% |
| 1925 | PHOSPHATIDYLINOSITOL\_MONOPHOSPHATE\_PHOSPHATASE\_ACTIVITY(GO:0052744) |  | 17 | 0.19 | 0.66 | 0.924 | 0.964 | 1.000 | 4918 | tags=12%, list=15%, signal=14% |
| 1926 | EXTRACELLULAR\_LIGAND\_GATED\_ION\_CHANNEL\_ACTIVITY(GO:0005230) |  | 89 | 0.21 | 0.66 | 0.859 | 0.965 | 1.000 | 1189 | tags=4%, list=4%, signal=5% |
| 1927 | RECEPTOR\_METABOLIC\_PROCESS(GO:0043112) |  | 42 | 0.21 | 0.66 | 0.935 | 0.964 | 1.000 | 2974 | tags=7%, list=9%, signal=8% |
| 1928 | POSTSYNAPTIC\_NEUROTRANSMITTER\_RECEPTOR\_ACTIVITY(GO:0098960) |  | 42 | 0.21 | 0.66 | 0.926 | 0.964 | 1.000 | 81 | tags=2%, list=0%, signal=2% |
| 1929 | SENSORY\_PERCEPTION\_OF\_LIGHT\_STIMULUS(GO:0050953) |  | 156 | 0.18 | 0.66 | 0.924 | 0.963 | 1.000 | 8071 | tags=22%, list=25%, signal=30% |
| 1930 | ADENYLATE\_CYCLASE\_INHIBITING\_G\_PROTEIN\_COUPLED\_RECEPTOR\_SIGNALING\_PATHWAY(GO:0007193) |  | 36 | 0.21 | 0.66 | 0.931 | 0.964 | 1.000 | 6919 | tags=22%, list=22%, signal=28% |
| 1931 | NUCLEOSOME\_ORGANIZATION(GO:0034728) |  | 79 | 0.17 | 0.65 | 0.921 | 0.965 | 1.000 | 941 | tags=4%, list=3%, signal=4% |
| 1932 | LIGAND\_GATED\_ION\_CHANNEL\_ACTIVITY(GO:0015276) |  | 148 | 0.19 | 0.65 | 0.924 | 0.965 | 1.000 | 3731 | tags=9%, list=12%, signal=11% |
| 1933 | LIGAND\_GATED\_CHANNEL\_ACTIVITY(GO:0022834) |  | 148 | 0.19 | 0.65 | 0.924 | 0.964 | 1.000 | 3731 | tags=9%, list=12%, signal=11% |
| 1934 | NEURAL\_PRECURSOR\_CELL\_PROLIFERATION(GO:0061351) |  | 36 | 0.15 | 0.65 | 0.912 | 0.971 | 1.000 | 8146 | tags=25%, list=26%, signal=34% |
| 1935 | NEGATIVE\_REGULATION\_OF\_TRANSPORTER\_ACTIVITY(GO:0032410) |  | 16 | 0.24 | 0.64 | 0.897 | 0.970 | 1.000 | 9315 | tags=38%, list=29%, signal=53% |
| 1936 | NEGATIVE\_REGULATION\_OF\_ION\_TRANSMEMBRANE\_TRANSPORTER\_ACTIVITY(GO:0032413) |  | 16 | 0.24 | 0.64 | 0.897 | 0.970 | 1.000 | 9315 | tags=38%, list=29%, signal=53% |
| 1937 | POTASSIUM\_ION\_ANTIPORTER\_ACTIVITY(GO:0022821) |  | 15 | 0.21 | 0.64 | 0.919 | 0.970 | 1.000 | 10367 | tags=53%, list=33%, signal=79% |
| 1938 | HOMOLOGOUS\_CHROMOSOME\_SEGREGATION(GO:0045143) |  | 22 | 0.20 | 0.64 | 0.925 | 0.970 | 1.000 | 8939 | tags=32%, list=28%, signal=44% |
| 1939 | NEUROEPITHELIAL\_CELL\_DIFFERENTIATION(GO:0060563) |  | 27 | 0.18 | 0.64 | 0.905 | 0.970 | 1.000 | 26248 | tags=100%, list=82%, signal=565% |
| 1940 | MITOTIC\_CYTOKINESIS(GO:0000281) |  | 37 | 0.16 | 0.64 | 0.867 | 0.970 | 1.000 | 13441 | tags=49%, list=42%, signal=84% |
| 1941 | REGULATION\_OF\_STRIATED\_MUSCLE\_TISSUE\_DEVELOPMENT(GO:0016202) |  | 20 | 0.22 | 0.64 | 0.892 | 0.969 | 1.000 | 6856 | tags=25%, list=21%, signal=32% |
| 1942 | EMBRYONIC\_CAMERA\_TYPE\_EYE\_MORPHOGENESIS(GO:0048596) |  | 40 | 0.17 | 0.64 | 0.921 | 0.970 | 1.000 | 7137 | tags=18%, list=22%, signal=23% |
| 1943 | WIDE\_PORE\_CHANNEL\_ACTIVITY(GO:0022829) |  | 41 | 0.19 | 0.64 | 0.947 | 0.972 | 1.000 | 7440 | tags=27%, list=23%, signal=35% |
| 1944 | CGMP\_METABOLIC\_PROCESS(GO:0046068) |  | 23 | 0.20 | 0.64 | 0.932 | 0.971 | 1.000 | 10854 | tags=35%, list=34%, signal=53% |
| 1945 | REGULATION\_OF\_MUSCLE\_TISSUE\_DEVELOPMENT(GO:1901861) |  | 21 | 0.22 | 0.64 | 0.898 | 0.971 | 1.000 | 6856 | tags=24%, list=21%, signal=30% |
| 1946 | LARVAL\_LOCOMOTORY\_BEHAVIOR(GO:0008345) |  | 16 | 0.23 | 0.64 | 0.896 | 0.971 | 1.000 | 6322 | tags=19%, list=20%, signal=23% |
| 1947 | LARVAL\_BEHAVIOR(GO:0030537) |  | 16 | 0.23 | 0.64 | 0.896 | 0.970 | 1.000 | 6322 | tags=19%, list=20%, signal=23% |
| 1948 | INORGANIC\_ANION\_TRANSMEMBRANE\_TRANSPORTER\_ACTIVITY(GO:0015103) |  | 128 | 0.16 | 0.64 | 0.959 | 0.970 | 1.000 | 6400 | tags=16%, list=20%, signal=20% |
| 1949 | SENSORY\_PERCEPTION(GO:0007600) |  | 308 | 0.16 | 0.63 | 0.977 | 0.972 | 1.000 | 8071 | tags=23%, list=25%, signal=30% |
| 1950 | DEOXYRIBONUCLEASE\_ACTIVITY(GO:0004536) |  | 40 | 0.17 | 0.63 | 0.903 | 0.973 | 1.000 | 7133 | tags=20%, list=22%, signal=26% |
| 1951 | REGULATION\_OF\_ORGAN\_MORPHOGENESIS(GO:2000027) |  | 49 | 0.15 | 0.63 | 0.964 | 0.974 | 1.000 | 8146 | tags=18%, list=26%, signal=25% |
| 1952 | CGMP\_BIOSYNTHETIC\_PROCESS(GO:0006182) |  | 21 | 0.21 | 0.63 | 0.979 | 0.973 | 1.000 | 10854 | tags=38%, list=34%, signal=58% |
| 1953 | INNER\_EAR\_AUDITORY\_RECEPTOR\_CELL\_DIFFERENTIATION(GO:0042491) |  | 26 | 0.18 | 0.62 | 0.897 | 0.974 | 1.000 | 26248 | tags=100%, list=82%, signal=565% |
| 1954 | GAMMA\_AMINOBUTYRIC\_ACID\_SIGNALING\_PATHWAY(GO:0007214) |  | 16 | 0.25 | 0.62 | 0.905 | 0.974 | 1.000 | 9557 | tags=31%, list=30%, signal=45% |
| 1955 | REGULATION\_OF\_MYELOID\_LEUKOCYTE\_DIFFERENTIATION(GO:0002761) |  | 16 | 0.22 | 0.62 | 0.978 | 0.974 | 1.000 | 5609 | tags=19%, list=18%, signal=23% |
| 1956 | NEUROPEPTIDE\_RECEPTOR\_BINDING(GO:0071855) |  | 29 | 0.22 | 0.62 | 0.942 | 0.973 | 1.000 | 9866 | tags=38%, list=31%, signal=55% |
| 1957 | DETECTION\_OF\_CHEMICAL\_STIMULUS\_INVOLVED\_IN\_SENSORY\_PERCEPTION\_OF\_SMELL(GO:0050911) |  | 60 | 0.19 | 0.62 | 0.978 | 0.973 | 1.000 | 9226 | tags=30%, list=29%, signal=42% |
| 1958 | U2\_TYPE\_SPLICEOSOMAL\_COMPLEX(GO:0005684) |  | 48 | 0.13 | 0.61 | 0.886 | 0.978 | 1.000 | 2280 | tags=4%, list=7%, signal=4% |
| 1959 | SARCOPLASMIC\_RETICULUM(GO:0016529) |  | 28 | 0.18 | 0.61 | 0.891 | 0.978 | 1.000 | 6865 | tags=18%, list=22%, signal=23% |
| 1960 | DETERMINATION\_OF\_DORSAL\_VENTRAL\_ASYMMETRY(GO:0048262) |  | 33 | 0.13 | 0.61 | 0.979 | 0.978 | 1.000 | 9065 | tags=21%, list=28%, signal=30% |
| 1961 | ODORANT\_BINDING(GO:0005549) |  | 61 | 0.19 | 0.61 | 0.978 | 0.979 | 1.000 | 9226 | tags=30%, list=29%, signal=41% |
| 1962 | PROTEIN\_SERINE\_THREONINE\_PHOSPHATASE\_ACTIVITY(GO:0004722) |  | 51 | 0.14 | 0.61 | 0.923 | 0.979 | 1.000 | 3535 | tags=8%, list=11%, signal=9% |
| 1963 | REGULATION\_OF\_VASOCONSTRICTION(GO:0019229) |  | 23 | 0.21 | 0.61 | 0.924 | 0.979 | 1.000 | 10070 | tags=39%, list=32%, signal=57% |
| 1964 | BETA\_CATENIN\_BINDING(GO:0008013) |  | 30 | 0.16 | 0.61 | 0.851 | 0.979 | 1.000 | 12977 | tags=57%, list=41%, signal=95% |
| 1965 | CATENIN\_COMPLEX(GO:0016342) |  | 23 | 0.18 | 0.60 | 0.950 | 0.979 | 1.000 | 26291 | tags=100%, list=82%, signal=569% |
| 1966 | VOLTAGE\_GATED\_SODIUM\_CHANNEL\_ACTIVITY(GO:0005248) |  | 17 | 0.24 | 0.60 | 0.856 | 0.980 | 1.000 | 3663 | tags=18%, list=11%, signal=20% |
| 1967 | IONOTROPIC\_GLUTAMATE\_RECEPTOR\_ACTIVITY(GO:0004970) |  | 27 | 0.22 | 0.60 | 0.949 | 0.981 | 1.000 | 9450 | tags=33%, list=30%, signal=47% |
| 1968 | EMBRYONIC\_AXIS\_SPECIFICATION(GO:0000578) |  | 19 | 0.20 | 0.60 | 0.954 | 0.981 | 1.000 | 4328 | tags=16%, list=14%, signal=18% |
| 1969 | INNER\_EAR\_RECEPTOR\_CELL\_DIFFERENTIATION(GO:0060113) |  | 43 | 0.14 | 0.59 | 0.886 | 0.984 | 1.000 | 27581 | tags=100%, list=86%, signal=739% |
| 1970 | CELL\_ACTIVATION\_INVOLVED\_IN\_IMMUNE\_RESPONSE(GO:0002263) |  | 36 | 0.16 | 0.59 | 0.976 | 0.985 | 1.000 | 7314 | tags=22%, list=23%, signal=29% |
| 1971 | DETECTION\_OF\_STIMULUS\_INVOLVED\_IN\_SENSORY\_PERCEPTION(GO:0050906) |  | 90 | 0.16 | 0.58 | 0.978 | 0.985 | 1.000 | 10219 | tags=31%, list=32%, signal=46% |
| 1972 | ACTIVIN\_RECEPTOR\_SIGNALING\_PATHWAY(GO:0032924) |  | 26 | 0.17 | 0.58 | 0.897 | 0.985 | 1.000 | 2580 | tags=8%, list=8%, signal=8% |
| 1973 | NOTOCHORD\_MORPHOGENESIS(GO:0048570) |  | 19 | 0.18 | 0.58 | 0.848 | 0.987 | 1.000 | 3791 | tags=11%, list=12%, signal=12% |
| 1974 | REGULATION\_OF\_BLOOD\_COAGULATION(GO:0030193) |  | 16 | 0.21 | 0.57 | 0.978 | 0.987 | 1.000 | 8291 | tags=38%, list=26%, signal=51% |
| 1975 | REGULATION\_OF\_HEMOSTASIS(GO:1900046) |  | 16 | 0.21 | 0.57 | 0.978 | 0.986 | 1.000 | 8291 | tags=38%, list=26%, signal=51% |
| 1976 | EYE\_PHOTORECEPTOR\_CELL\_DIFFERENTIATION(GO:0001754) |  | 59 | 0.16 | 0.57 | 0.925 | 0.988 | 1.000 | 6934 | tags=15%, list=22%, signal=19% |
| 1977 | SENSORY\_PERCEPTION\_OF\_SMELL(GO:0007608) |  | 69 | 0.17 | 0.57 | 0.977 | 0.988 | 1.000 | 9226 | tags=29%, list=29%, signal=41% |
| 1978 | AUDITORY\_RECEPTOR\_CELL\_DEVELOPMENT(GO:0060117) |  | 19 | 0.18 | 0.55 | 0.977 | 0.991 | 1.000 | 26248 | tags=100%, list=82%, signal=565% |
| 1979 | REGULATION\_OF\_GLIOGENESIS(GO:0014013) |  | 19 | 0.17 | 0.55 | 0.952 | 0.991 | 1.000 | 26627 | tags=100%, list=83%, signal=605% |
| 1980 | NEUROTRANSMITTER\_TRANSPORTER\_ACTIVITY(GO:0005326) |  | 38 | 0.14 | 0.54 | 0.977 | 0.994 | 1.000 | 8018 | tags=21%, list=25%, signal=28% |
| 1981 | PURINE\_NUCLEOBASE\_METABOLIC\_PROCESS(GO:0006144) |  | 20 | 0.16 | 0.53 | 0.948 | 0.997 | 1.000 | 7572 | tags=20%, list=24%, signal=26% |
| 1982 | INNER\_EAR\_RECEPTOR\_CELL\_STEREOCILIUM\_ORGANIZATION(GO:0060122) |  | 15 | 0.18 | 0.52 | 0.947 | 0.997 | 1.000 | 26248 | tags=100%, list=82%, signal=565% |
| 1983 | CHLORIDE\_TRANSMEMBRANE\_TRANSPORT(GO:1902476) |  | 50 | 0.16 | 0.52 | 0.978 | 0.998 | 1.000 | 6186 | tags=14%, list=19%, signal=17% |
| 1984 | SODIUM\_CHANNEL\_ACTIVITY(GO:0005272) |  | 31 | 0.15 | 0.51 | 0.979 | 0.999 | 1.000 | 3663 | tags=10%, list=11%, signal=11% |
| 1985 | OLFACTORY\_RECEPTOR\_ACTIVITY(GO:0004984) |  | 69 | 0.15 | 0.50 | 0.978 | 0.999 | 1.000 | 9226 | tags=28%, list=29%, signal=39% |
| 1986 | REGULATION\_OF\_PATHWAY\_RESTRICTED\_SMAD\_PROTEIN\_PHOSPHORYLATION(GO:0060393) |  | 35 | 0.15 | 0.50 | 0.954 | 0.999 | 1.000 | 11849 | tags=43%, list=37%, signal=68% |
| 1987 | EPHRIN\_RECEPTOR\_BINDING(GO:0046875) |  | 15 | 0.17 | 0.50 | 0.978 | 0.999 | 1.000 | 9687 | tags=27%, list=30%, signal=38% |
| 1988 | STRUCTURAL\_CONSTITUENT\_OF\_EYE\_LENS(GO:0005212) |  | 58 | 0.19 | 0.50 | 0.940 | 0.998 | 1.000 | 8071 | tags=24%, list=25%, signal=32% |
| 1989 | POSTSYNAPTIC\_MEMBRANE(GO:0045211) |  | 164 | 0.14 | 0.49 | 0.979 | 0.999 | 1.000 | 1189 | tags=3%, list=4%, signal=3% |
| 1990 | ANCHORED\_COMPONENT\_OF\_PLASMA\_MEMBRANE(GO:0046658) |  | 27 | 0.16 | 0.49 | 0.954 | 0.998 | 1.000 | 3632 | tags=7%, list=11%, signal=8% |
| 1991 | STABILIZATION\_OF\_MEMBRANE\_POTENTIAL(GO:0030322) |  | 19 | 0.17 | 0.49 | 0.978 | 0.998 | 1.000 | 8968 | tags=32%, list=28%, signal=44% |
| 1992 | HISTONE\_H3\_K4\_METHYLATION(GO:0051568) |  | 17 | 0.13 | 0.47 | 0.944 | 0.999 | 1.000 | 27720 | tags=100%, list=87%, signal=764% |
| 1993 | HISTONE\_EXCHANGE(GO:0043486) |  | 17 | 0.16 | 0.45 | 0.977 | 1.000 | 1.000 | 22646 | tags=94%, list=71%, signal=324% |
| 1994 | ATP\_DEPENDENT\_CHROMATIN\_REMODELING(GO:0043044) |  | 17 | 0.16 | 0.45 | 0.977 | 1.000 | 1.000 | 22646 | tags=94%, list=71%, signal=324% |
| 1995 | SMAD\_PROTEIN\_SIGNAL\_TRANSDUCTION(GO:0060395) |  | 41 | 0.13 | 0.45 | 0.979 | 0.999 | 1.000 | 11759 | tags=37%, list=37%, signal=58% |
| 1996 | POSITIVE\_REGULATION\_OF\_PATHWAY\_RESTRICTED\_SMAD\_PROTEIN\_PHOSPHORYLATION(GO:0010862) |  | 31 | 0.14 | 0.44 | 0.979 | 1.000 | 1.000 | 11537 | tags=39%, list=36%, signal=61% |
| 1997 | CHLORIDE\_CHANNEL\_COMPLEX(GO:0034707) |  | 41 | 0.14 | 0.41 | 0.948 | 1.000 | 1.000 | 3752 | tags=7%, list=12%, signal=8% |
| 1998 | GABA\_A\_RECEPTOR\_COMPLEX(GO:1902711) |  | 20 | 0.16 | 0.37 | 0.978 | 1.000 | 1.000 | 10370 | tags=30%, list=33%, signal=44% |
| 1999 | GABA\_RECEPTOR\_COMPLEX(GO:1902710) |  | 20 | 0.16 | 0.37 | 0.978 | 1.000 | 1.000 | 10370 | tags=30%, list=33%, signal=44% |
| 2000 | GABA\_A\_RECEPTOR\_ACTIVITY(GO:0004890) |  | 21 | 0.15 | 0.35 | 0.979 | 1.000 | 1.000 | 11198 | tags=33%, list=35%, signal=51% |
Table: Gene sets enriched in phenotype
 **wfs1KO (4 samples)
 **[plain text format]****
